# Enhanced glycerol assimilation and lipid production in *Rhodotorula toruloides* CBS14 upon addition of hemicellulose primarily correlates with early transcription of energy-metabolism-related genes

**DOI:** 10.1186/s13068-023-02294-3

**Published:** 2023-03-10

**Authors:** Giselle C. Martín-Hernández, Mikołaj Chmielarz, Bettina Müller, Christian Brandt, Adrian Viehweger, Martin Hölzer, Volkmar Passoth

**Affiliations:** 1 Department of Molecular Sciences, BioCenter, Swedish University of Agricultural Sciences, Box 7015, 75007 Uppsala, Sweden; 2 Institute for Infectious Diseases and Infection Control, Jena University Hospital, Jena, Germany; 3 Institute of Medical Microbiology and Virology, University Hospital Leipzig, 04103 Leipzig, Germany; 4 Method Development and Research Infrastructure, Bioinformatics and Systems Biology, Robert Koch Institute, 13353 Berlin, Germany

**Keywords:** *Rhodotorula toruloides*, Transcriptomics, Lignocellulose, Glycerol, Biofuels

## Abstract

**Background:**

Lipid formation from glycerol was previously found to be activated in *Rhodotorula toruloides* when the yeast was cultivated in a mixture of crude glycerol (CG) and hemicellulose hydrolysate (CGHH) compared to CG as the only carbon source. RNA samples from *R. toruloides* CBS14 cell cultures grown on either CG or CGHH were collected at different timepoints of cultivation, and a differential gene expression analysis was performed between cells grown at a similar physiological situation.

**Results:**

We observed enhanced transcription of genes involved in oxidative phosphorylation and enzymes localized in mitochondria in CGHH compared to CG. Genes involved in protein turnover, including those encoding ribosomal proteins, translation elongation factors, and genes involved in building the proteasome also showed an enhanced transcription in CGHH compared to CG. At 10 h cultivation, another group of activated genes in CGHH was involved in β-oxidation, handling oxidative stress and degradation of xylose and aromatic compounds. Potential bypasses of the standard *GUT1* and *GUT2*-glycerol assimilation pathway were also expressed and upregulated in CGHH 10 h. When the additional carbon sources from HH were completely consumed, at CGHH 36 h, their transcription decreased and NAD^+^-dependent glycerol-3-phosphate dehydrogenase was upregulated compared to CG 60 h, generating NADH instead of NADPH with glycerol catabolism. *TPI1* was upregulated in CGHH compared to cells grown on CG in all physiological situations, potentially channeling the DHAP formed through glycerol catabolism into glycolysis. The highest number of upregulated genes encoding glycolytic enzymes was found after 36 h in CGHH, when all additional carbon sources were already consumed.

**Conclusions:**

We suspect that the physiological reason for the accelerated glycerol assimilation and faster lipid production, was primarily the activation of enzymes that provide energy.

**Supplementary Information:**

The online version contains supplementary material available at 10.1186/s13068-023-02294-3.

## Introduction

One of the world's fastest-growing food commodities is vegetable oils (VO) [1]. VO are also used as the main feedstock for biodiesel production, a renewable energy source and alternative to fossil fuels [2]. VO consumption in Sweden and the European Union (EU) is much higher than production. Thus, a significant proportion of the VO used for biodiesel production is imported [3]. Furthermore, a high greenhouse gas potential is associated with producing many VO, such as palm-, soybean- and peanut oil, whose emissions are reported to exceed 2000 kg CO_2_ equivalents per ton produced [4, 5]. Substantial rainforest clearing due to land use is also associated with producing these VO [4, 6].

Microbial oils have the potential to replace VO in the production of biodiesel and feed and food additives [7, 8]. Oleaginous yeasts are known to be among the fastest lipid producers on earth [9–11]. Among these, *Rhodotorula* species, which are basidiomycetes oleaginous yeasts, can convert a variety of industrial low-value residues, such as lignocellulose hydrolysates, including hemicellulose from pulp-and-paper industry or crude glycerol, a residue from biodiesel production, into lipids and carotenoids [11–17]. In a recent study, we could show that lipid production rate was increased in *Rhodotorula* species when cultured on crude glycerol and hemicellulose hydrolysate (CGHH), compared to crude glycerol (CG) as the sole carbon source [13]. The physiological basis of this phenomenon remains to be elucidated.

The increasing availability of *R. toruloides* genome assemblies has enabled differential gene expression studies, which both contribute to the understanding of carbon metabolism and allow the construction of metabolic models at the genome level for this species [18–25]. Recently, we assembled the genome of *R. toruloides* CBS14 at the chromosomal level, with the highest number of annotated transcripts published so far for *R. toruloide*s [26]. This study aimed to compare gene transcription of *R. toruloides* CBS14 cultivated in either CGHH or CG, to get an insight into the metabolic pathways that are activated due to the presence of HH and enhance glycerol turnover.

## Materials and methods

### Bioreactor cultivation and sampling

Cultivations were performed as described in [13]. Briefly, bioreactors (Multifors, Infors HT, Bottmingen, Switzerland) with 500 mL working volume were used for growing *R. toruloides* CBS14 containing either CGHH (50% CG, 10% HH) or only CG (50% CG) as carbon sources as well as 0.75 g/L yeast extract (BactoTM Yeast Extract, BD, France), 1 g/L MgCl_2_ 6xH_2_O (Merck KGaA, Germany), 2 g/L (NH_4_)_2_HPO_4_ (≥ 98%, Sigma-Aldrich, USA) and 1.7 g/L YNB without amino acids and ammonium sulphate (DifcoTM, Becton Dickinson, France). To inoculate the bioreactors, cells from a −80 °C stock culture were streaked on YPD-agar (glucose 20 g/L, yeast extract 10 g/L, peptone 20 g/L) and incubated for 3 days. From the plates, cells were inoculated into 100 mL YPD in 500 mL baffled Erlenmeyer flasks. After incubation on an orbital shaker at 125 rpm and 25 °C for 72 h, the cells were transferred to 50 mL-Falcon tubes, washed twice with NaCl-solution (9 g/L) and re-suspended with NaCl-solution. Cultures were inoculated with the cell suspension, to reach an initial OD of 1 in the cultivation. Cultivations were performed in triplicate at 25 °C, pH 6, and an oxygen tension of 21%.

CG was obtained from Perstorp Holding AB, Sweden. The glycerol concentration was specified as 80%; however, there were differences from batch to batch. For the cultivations described here, the same batch was used as in the bioreactor experiments in [13]. HH was generated from wheat straw, after steam explosion and enzymatic digestion. Pretreatment was performed at Lund University, Sweden, as described previously [16]. Briefly, the straw was soaked with 1% acetic acid overnight, and then steam exploded at 190 °C. The liquid fraction, representing the solubilised hemicellulose, was removed from the solid fraction by pressing and used in the experiments. HH contained 26.2 g/L xylose, 7 g/L glucose, 6.6 g/L acetic acid and small amounts of arabinose (< 0.5 g/L). The nitrogen content was 0.6 g/l [27]. The pH was set to 6 by addition of appropriate amounts of 5 M NaOH [13]. The C/N-ratios were 32.5 for CGHH and 30.7 for CG.

Samples for RNA-isolation and determination of the concentrations of biomass, carbon sources and lipids were isolated from *R. toruloides* CBS14 cultures grown at different growth conditions: they were taken from CG cultures after 10 h, 30 h, and 60 h and from CGHH cultures after 10 h, 36 h, and 60 h. Cell dry weight was determined gravimetrically, and glucose, xylose, acetic acid and arabinose were determined by HPLC [17]. Lipid content was measured using FT-NIR, as described previously [28]. Cell samples for RNA isolation (3 mL) were rapidly collected in Falcon tubes and placed in ice to decrease sample temperature.

### RNA extraction and sequencing

Total RNA was immediately extracted in triplicates from each sample following a previously described protocol [26]. rRNA-depleted samples were sequenced on the Illumina MiSeq system using the reagent kit v3, and paired-end RNA sequencing reads were obtained (2 × 75 bp). The base-calling pipeline included MiSeq Control Software v2.6.2.1, RTA v1.18.54 and bcl2fastq v2.20.0.422.

### RNA-Seq data analysis

The RNAflow differential gene expression pipeline v1.1.0 [29] was used for transcriptome analysis. This includes read quality control, count normalization, reference-based mapping, gene quantification, differential expression, and visualization. Samples were quality-checked with FastQC, and raw reads were trimmed with fastp to remove low-value bases and adapter contamination [30]. After quality-trimming, the transcriptomics data had an average coverage of 1.6 M (0.8–3.8 M) reads per sample, with an expected read depth of 28X. For read depth estimation, the whole transcriptome size was calculated to be 8,484,192 bp, based on 5958 average number of transcribed genes (TPM > 0) per sample, and an average transcript length of 1424 bp. The remaining rRNA was detected and removed using SortMeRNA [31]. Then, reads were splice-aware aligned with HISAT2 [32] to the *R. toruloides* assembly as previously reported [26]. FeatureCounts was used for gene-level expression quantification [33] only considering uniquely mapped reads and using the annotation file from [26]. To identify and remove low-expressed genes, the TPM values (transcripts per million kilobases) were determined using RNAflow [29]. The TPM value was determined for each sample and each gene. Furthermore, the mean TPM value per condition was calculated over all biological replicates. A gene had to have a particular mean TPM value above a defined threshold to be considered in the subsequent analyses. We performed all calculations using a TPM 5 as the default threshold. Finally, differential gene expression analysis was performed using DESeq2 [34] to identify significantly (adjusted *p* value < 0.05) differentially expressed genes. We adjusted the p values attained by the Wald test in DESeq2 [34] for multiple testing using the Benjamini and Hochberg method, implemented as a default in DESeq2's results() function. As recommended, we used these adjusted *p* values to identify genes with significantly different expressions. Corresponding R packages were used to conduct principal component analysis and generate expression heatmaps and box plots. Details of the tool versions, R packages, and custom scripts used can be found at https://github.com/hoelzer-lab/rnaflow.

Visualization of gene expression density in the genome of *R. toruloides* CBS14 was performed using Circa (http://omgenomics.com/circa; accessed on May 2022). The respective KEGG orthology (KO) numbers were assigned to those annotated proteins encoded by differentially expressed genes. Subsequently, metabolic pathways and cellular processes were determined using KofamKOALA [35]. Gene ontology (GO) terms from differentially expressed genes that occurred at least 10 times were visualized using REVIGO [36] in semantic similarity-based scatterplots. Blast homology search (v 2.13.0+) was performed to identify genes and proteins belonging to central metabolic pathways annotated with a similar function in CBS14 [37, 38].

## Results

### RNA sampling points were selected according to the dynamics of glycerol consumption in CG or CGHH

**Fig. 1:**
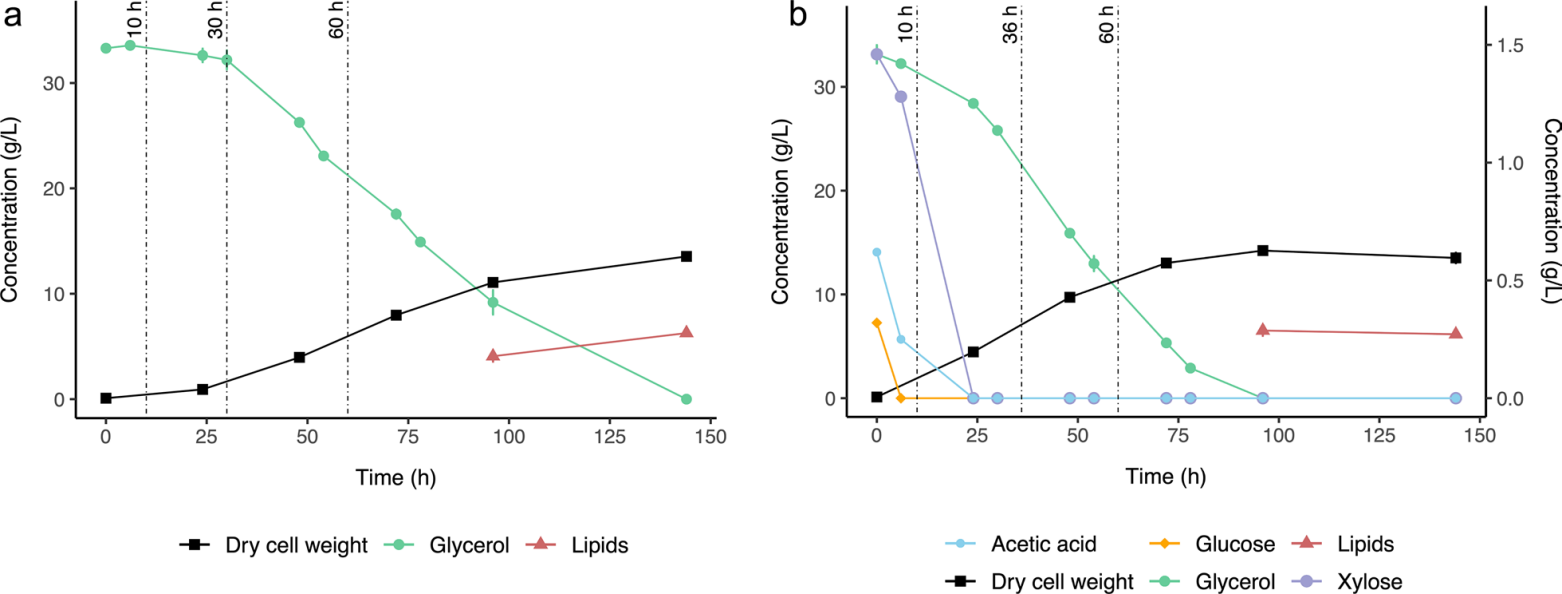
Bioreactor cultivation of *Rhodotorula toruloides* CBS14 using as carbon source: **a** crude glycerol (CG), or **b** mixture of CG and hemicellulose hydrolysate (CGHH). The reconstruction of the cultivation curves was performed using results from [13]. Independent cultures, different from those whose values are the basis of the shown growth curves, were performed for RNA-isolation. At each sampling, concentrations of biomass, lipids and substrate were determined, to ensure the cells were in the same growth phase as indicated in the figure. Glycerol concentrations are given on the primary y axis. The secondary y axis indicates the concentrations of xylose, glucose and acetic acid in the hemicellulose hydrolysate. Vertical dashed lines represent sampling points for RNA extraction

To identify genes and metabolic pathways active during glycerol turnover in *R. toruloides* CBS14, cells were cultivated under different growth conditions. Differential gene expression analysis was performed by bulk RNA-sequencing (RNA-seq) at different timepoints as explained below: as mentioned above, *R. toruloides* CBS14 showed different growth rates in cultivation media using either CG or CGHH as the main carbon source [13]. Faster growth, faster initial glycerol consumption, and more rapid lipid formation were observed in CGHH compared to CG [13]. Thus, sampling timing was selected based on the observed dynamics of glycerol consumption. RNA isolation was done in three independent cultivations for each culture medium (sampling points are illustrated in Fig. 1). The first sampling was performed after 10 h to allow the cultures to adapt to the cultivation conditions. In CGHH, the consumption of glycerol was visible after 10 h. However, a physiologically comparable situation was reached in the CG culture after about 30 h, so a further sample was taken from the CG culture at this time. Another sampling point was chosen after 36 h in CGHH and 60 h in CG. In CGHH, about 20 g/L of glycerol was left at this timepoint and the additional carbon sources from HH were consumed entirely. This culture condition was thus similar to the CG-culture after 60 h, where about 20 g/L was also left. In the CGHH culture, another sample was taken after 60 h. At this point, glycerol was still present, but only half as much after 36 h. Thus, the expression profile of this sample may reflect physiological responses to different glycerol concentrations.

### Global gene expression patterns differ clearly between timepoints and carbon sources

**Fig. 2:**
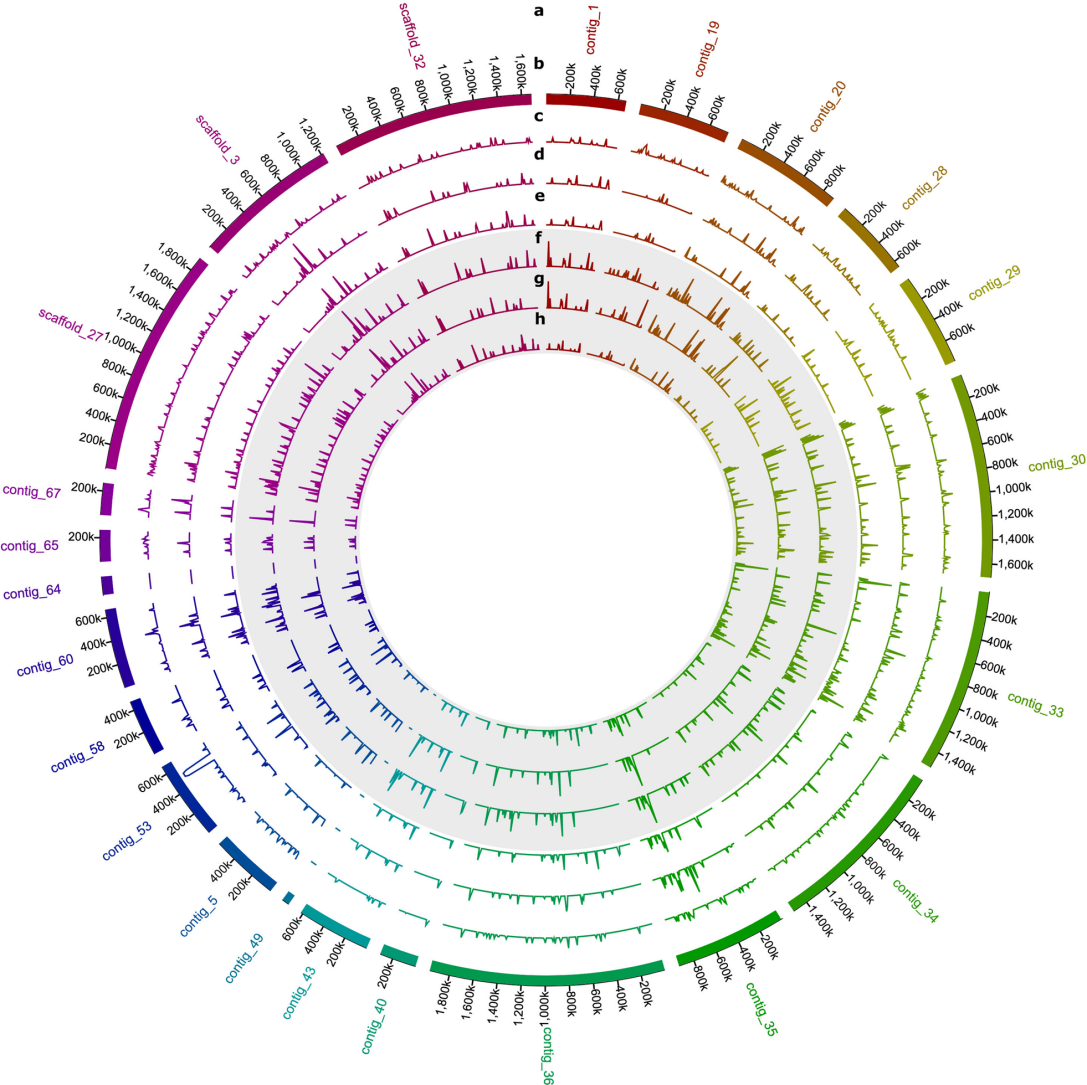
Map of genes expressed over the mean level of TPM in *Rhodotorula toruloides* CBS14. Gene densities when grown on each of the two different carbon sources are indicated in concentric circles. From outside to inside: **a** *R. toruloides* CBS14 contig names; **b** sizes; and in 10 kb windows, density of genes expressed over the mean TPM level in CBS14 when grown in CGHH as main carbon source at **c** 10 h **d** 36 h and **e** 60 h; and in CG at **f** 10 h, **g** 30 h and **h** 60 h. The circles representing samples from cells grown in CG are also indicated in gray. Only nuclear encoded genes are included in this graph. CG, crude glycerol; CGHH, mixture of CG and hemicellulose hydrolysate; TPM, transcripts per million kilobases

Prior to differential expression analysis with DESeq2, the quality of the transcriptome reads was checked. Passed reads were mapped and quantified using the annotated genome of *R. toruloides* CBS14 [26]. The number of TPM was calculated per gene and sample. The expression levels of each condition were thus normalized against gene length and sequencing depth. Weakly expressed genes were filtered out. The density of highly expressed genes within contigs and scaffolds from CBS14 genome assembly is shown in Fig. 2. An expression level of at least 105 TPM is evenly distributed throughout the genome, except for contigs 49 (length 62 kbp) and 64 (length 151 kbp), from which no transcripts were recovered. Differences in the expression profile can be spotted between different timepoints and media. In addition, gene expression density and transcription levels in the mitochondrial genome were much higher than in the rest of the genome (results not shown). We conducted PCA to analyze differences in the clustering of biological replicates and global gene expression patterns between the samples (Additional file 1: Figs. S1–S4). The sampling time (reflected by PC1) explains 86.6% of the variance, and 8% variance is explained by medium composition (reflected by PC2) (Additional file 1: Fig. S1). The genes with annotated function which contributed most to the differences between conditions were in decreasing order: RHOT147219 (encoding NADH-ubiquinone oxidoreductase chain 1), RHOT147222 (cytochrome c oxidase subunit 1), RHOT142646 (sulfated surface glycoprotein 185), RHOT149100 (putative protein TPRXL) and RHOT149239 (elongation of fatty acids protein 3).

Transcription levels of genes were first compared pairwise between the sampling points of the same growth condition (CG or CGHH). More specifically, we compared each of the two later sampling points with the first after 10 h of growth and assigned the identified differentially expressed genes to the KEGG metabolic pathways and cellular processes (Additional file 1: Fig. S5). The accounted genes summarized in Additional file 1: Fig. S5 showed a significant up- or downregulation (*p* < 0.05) with a log_2_Fold change > 1.5 or < − 1.5, which is also in line with the high variance in gene expression shown by the principal component 1 (PC1 86.6%, see Additional file 1: Fig. S1). In both CGHH and CG, more genes per KEGG pathway and process were higher transcribed at the 10 h samplings compared to later samplings. In CGHH, glucose was exhausted at this timepoint (Fig. 1b), and thus, the significantly higher gene expression at 10 h compared to later timepoints is probably related to the transition to a broader spectrum of metabolic activities to assimilate other carbon sources [39]. In comparison, the number of differentially expressed genes in CG with no additional carbon sources remained close to zero between 10 and 30 h of growth for most pathways and processes. This is also indicated by the low expression variance between these samples (Additional file 1: Fig. S1).

**Fig. 3:**
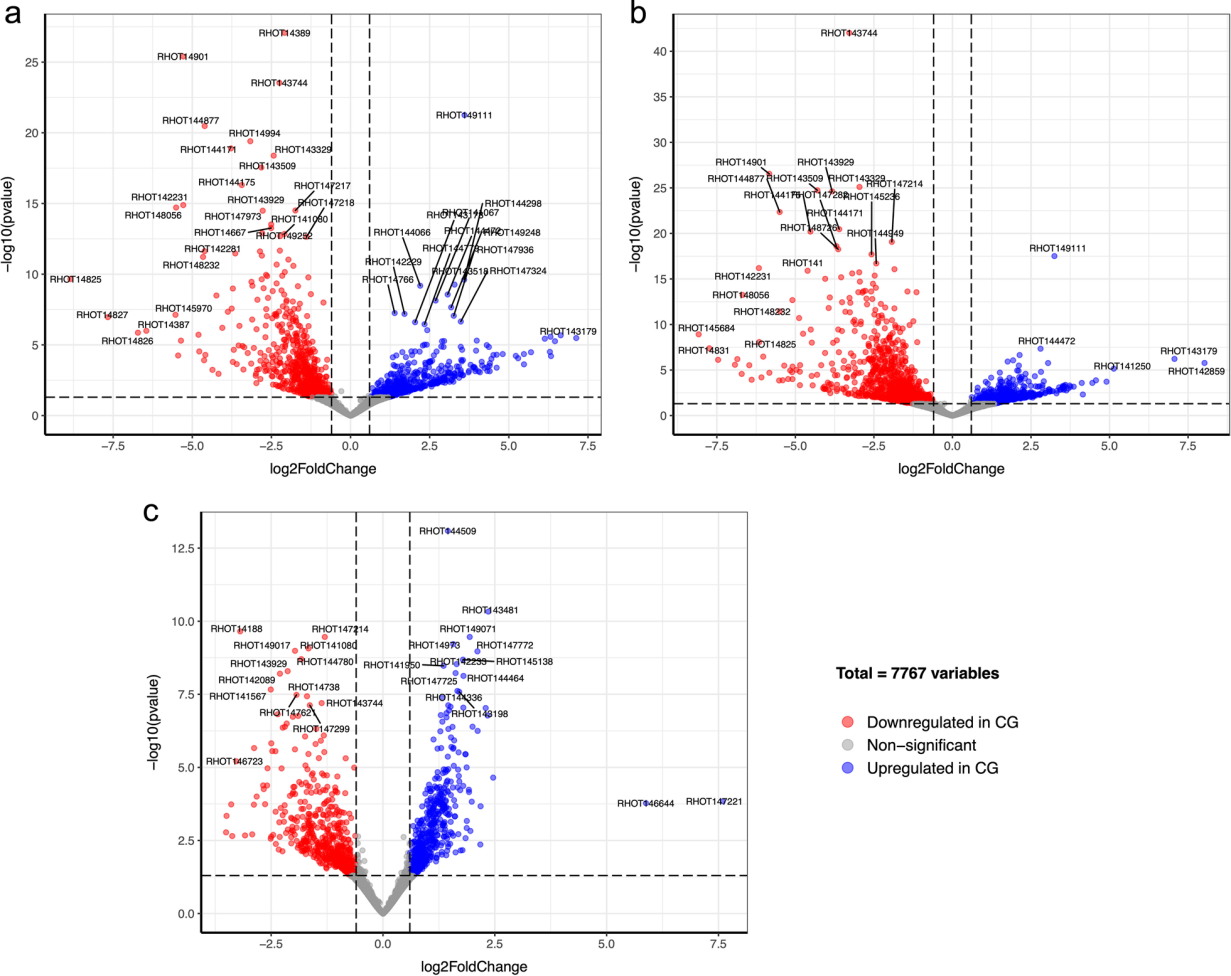
Top differentially expressed genes when comparing the cultures at timepoints with similar physiological situations (for detailed explanation, see section “RNA sampling points were selected according to the dynamics of glycerol consumption in CG or CGHH”): **a** CGHH 10 h versus CG 10 h; **b** CGHH 10 h versus CG 30 h; **c** CGHH 36 h versus CG 60 h. Upregulated genes (adjusted *p* value < 0.05 and log_2_Fold change > 0.6) in CG are indicated in blue while downregulated genes (adjusted *p* value < 0.05 and log_2_Fold change < − 0.6) in red. Genes that are not significantly differentially expressed are in gray. The higher in the y axis, the more significant, and the further to the left, the more downregulated in CG. For example, the gene encoding Peroxisomal multifunctional enzyme type 2 (RHOT14901) is significantly downregulated in CG 10 h and CG 30 h compared to CGHH 10 h. CG, crude glycerol; CGHH, mixture of CG and hemicellulose hydrolysate

Changes in transcript abundance were further evaluated to identify differentially expressed genes (adjusted *p* value < 0.05) between *R. toruloides* cell cultures grown on different carbon sources at a similar physiological situation, as illustrated in Volcano plots (Fig. 3; Additional file 1: Figs. S2–S4, and Additional file 1: Tables S1–S3). They correspond to samples after adapting to cultivation conditions in each medium (CGHH 10 h vs. CG 10 h), when glycerol consumption became visible (CGHH 10 h vs. CG 30 h), and when there is about 20 g/l of glycerol left, and the additional carbon sources from HH were completely consumed (CGHH 36 h vs. CG 60 h). GO term enrichment analysis revealed that these differentially expressed genes are involved in the biological processes illustrated in Additional file 1: Fig. S6.

### Increased protein turnover and energy metabolism in CGHH after 10 h of cultivation

**Fig. 4:**
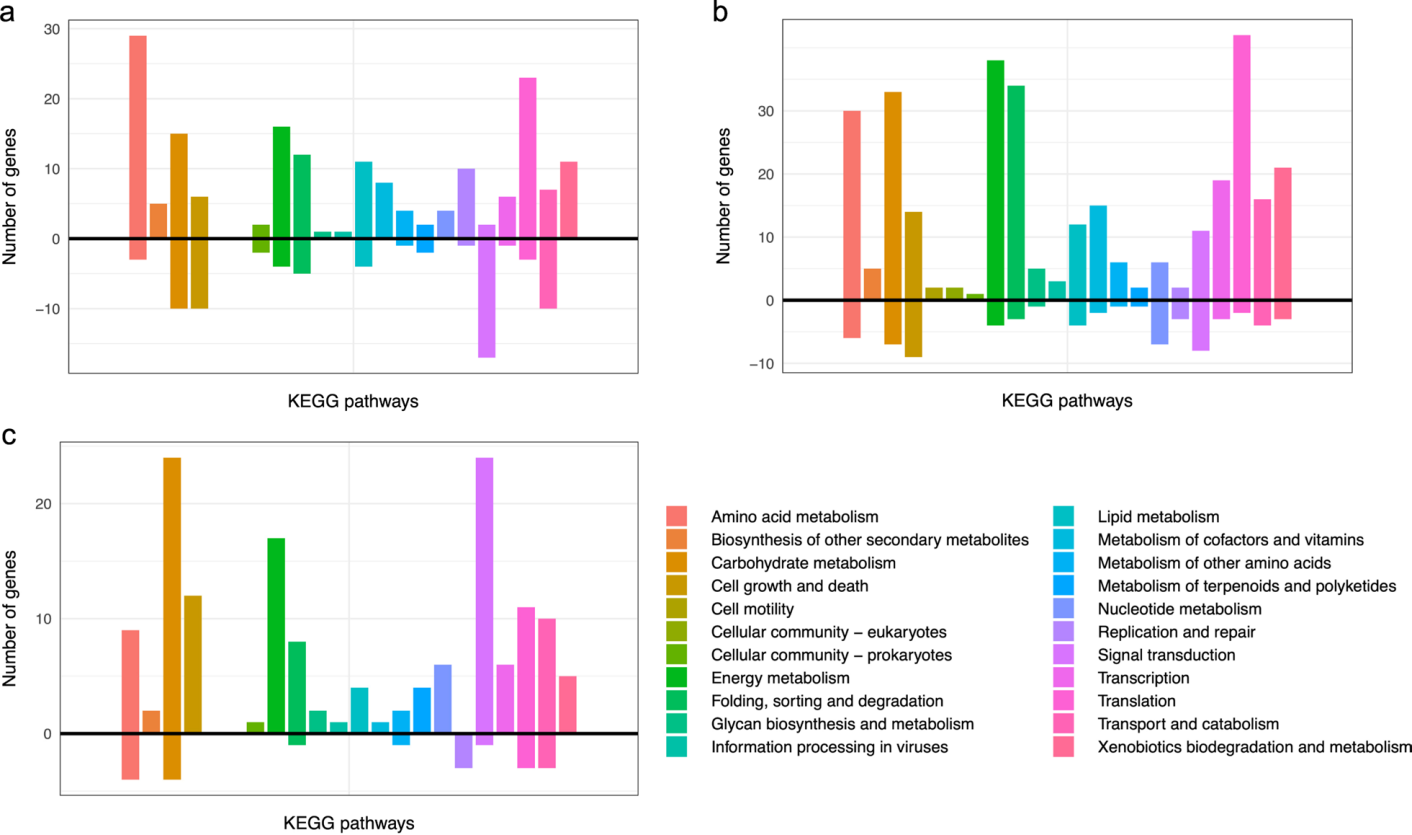
Number of differentially expressed genes in CG compared to CGHH per KEGG pathway and cellular process: **a** 10 h in CGHH versus 10 h in CG, **b** 10 h in CGHH versus 30 h in CG, and **c** 36 h in CGHH versus 60 h in CG. The accounted genes showed significant (*p* < 0.05) upregulation (+*y* axis) or downregulation (−*y* axis) in CGHH with an absolute log_2_Fold Change higher than 1.5. Bar colors indicate different KEGG pathways. CG, crude glycerol; CGHH, mixture of CG and hemicellulose hydrolysate

Of 634 differentially expressed genes, 396 were significantly higher transcribed (*p* < 0.05, log_2_Fold change < 0) in CGHH 10 h than in CG 10 h (Fig. 3a; Additional file 1: Table S1). Many of these genes were generally higher expressed also when compared to the other sampling points, both in CGHH and CG. Genes encoding enzymes involved in metabolic pathways were most differentially expressed within the assigned KEGG orthologs. An exceptionally high proportion of these genes are involved in amino acid metabolism and were upregulated mainly in cells grown in CGHH as carbon source (Fig. 4a). While genes involved in signal transduction had the highest number of upregulated genes among cellular processes in CG 10 h, genes involved in translation was highest in CGHH 10 h (Fig. 4a).

27 ribosomal protein genes were upregulated in CGHH 10 h, including both cytoplasmic and mitochondrial ribosomes (Additional file 1: Fig. S7b). Genes involved in ribosome biogenesis and spliceosome formation, as well as translation initiation factors and components of all three DNA-dependent RNA-polymerases were also higher expressed in CGHH. Apart from genes involved in protein synthesis, protein degradation also appeared activated in CGHH 10 h. Transcription of 13 proteasome-related genes was upregulated in CGHH (Additional file 1: Fig. S7c), while none were downregulated. Compared to all other measuring points, in both CGHH and CG, the TPM values of most of these proteasome-related genes were about 2–3-fold higher in CGHH 10 h.

**Fig. 5:**
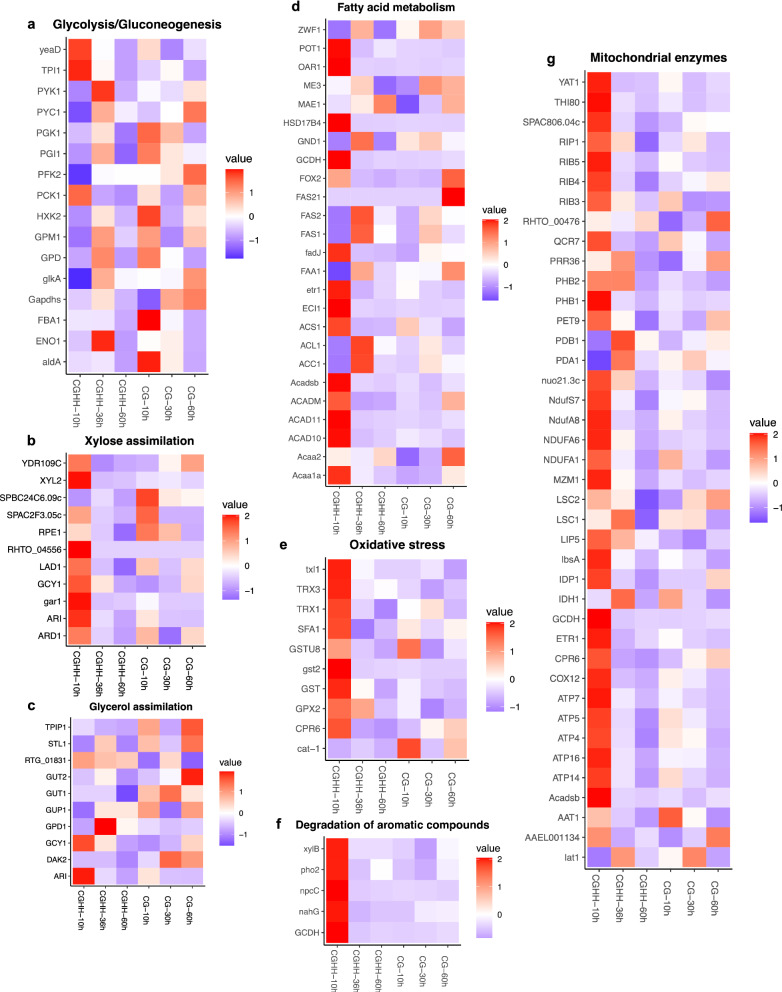
Gene expression in central metabolic pathways. Scaled TPM values from genes encoding enzymes involved in **a** glycolysis and gluconeogenesis, **b** xylose assimilation, **c** glycerol assimilation, **d** fatty acid metabolism and NADPH generation, **e** handling oxidative stress, **f** degradation of aromatic compounds and **g** mitochondrial enzymes involved in respiration. Color key stands for the z-scores obtained for each gene (normalized for all cultivation conditions, separately performed for each of the metabolic pathways)

Besides gene expression, protein synthesis, and protein degradation through proteasome, a high proportion of the upregulated genes in CGHH 10 h compared to CG 10 h were associated with energy metabolism (Fig. 4a). This includes especially genes encoding proteins for oxidative phosphorylation and other mitochondrial enzymes. 21 genes involved in the mitochondrial electron transport chain-complex I (NADH ubiquinone oxidoreductase), III (cytochrome c reductase), IV (cytochrome c oxidase), and F-type ATPase were significantly regulated (*p* < 0.05) in CGHH 10 h compared to CG 10 h. They included 20 genes that were upregulated in CGHH 10 h and one that was downregulated (Additional file 1: Fig. S7a). Two prohibitin genes (RHOT148333 and RHOT147096), involved in the formation of respiratory supercomplexes [40], were regulated similarly. A variety of genes encoding mitochondrial enzymes or accessory components followed the same pattern: they were upregulated in CGHH 10 h compared to CG 10 h, had the highest TPM values in CGHH 10 h, and were at 36 h lower, but still at a similar level as in CG 10 h (Fig. 5g and Additional file 1: Table S4). This includes genes encoding enzymes involved in the tricarboxylic acid (TCA) cycle, such as NADP^+^-specific isocitrate dehydrogenase (ICDH), succinate-CoA ligase, and fumarate hydratase (RHOT145845, -7009 and -5604, respectively), or in the synthesis of cofactors of mitochondrial enzymes, such as riboflavin (RHOT149252, 2556 and 2045), lipoic acid (by lipoyl synthase, RHOT145711) and thiamine pyrophosphate (TPP, by thiamine pyrophosphokinase, RHOT149040). Lipoic acid and TPP are cofactors of pyruvate dehydrogenase and α-keto glutarate dehydrogenase [41].

On the other hand, transcription of most genes involved in glycolytic reactions and regulation were not significantly different between CG 10 h and CGHH 10 h, when this medium still had xylose and acetic acid as additional carbon sources (Fig. 1b). The exceptions were the genes encoding a phosphofructokinase (*PFK2*, RHOT143173), which was downregulated in CGHH 10 h, phosphoglyceromutase (*GPM1*, RHOT146772) and triosephosphate isomerase (*TPI1*, RHOT141080), which were upregulated in CGHH 10 h. Other glycolytic genes were expressed constitutively. Phosphoglyceromutase (PGM) has been identified as the only glycolytic enzyme significantly differentially expressed in *R. toruloides* cells depending on the carbon source, glucose, or xylose [42]. It was reported for a recombinant *S. cerevisiae* strain that the overexpression of a *PGM*-gene enhanced xylose fermentation [43]. Furthermore, *PFK2* was also downregulated on YP (yeast/peptone) medium supplemented with either xylose or glucose when compared to the expression on YP only [22]. In the same study, *TPI1* was overexpressed when YP medium was supplemented with acetate [22].

Some of the suggested genes that are involved in xylose assimilation in *R. toruloides* are NAD(P)H-dependent xylose reductase (XR), NADH-dependent xylitol dehydrogenase (XDH), xylulose kinase (XK), and phosphoketolase [42]. By performing homology analyses, we identified the gene RHOT147125 (annotated as *ARI* in *R. toruloides* CBS14, homolog to Rhto_03963 in *R. toruloides* NP11 [25]) as XR. Its transcription was more than doubled in CGHH (Fig. 5b and Additional file 1: Table S4), although significant upregulation (*p* < 0.05) was only found compared to CG 30 h and to the later CGHH sample points. In both media, RHOT147125 expression decreased with time. Besides XR, Tiukova et al. [42] identified three aldo/keto reductases that were potentially involved in xylose assimilation. They are annotated in CBS14 as galacturonate reductase (RHOT148868), glycerol 2-dehydrogenase (NADP^+^) (RHOT14370), and uncharacterized oxidoreductase C2F3.05c (RHOT143299). RHOT148868 and RHOT14370 were upregulated (*p* < 0.05) in CGHH 10 h compared to CG 10 h. The gene encoding XDH (RHOT149007) was significantly higher transcribed (*p* < 0.05) at CGHH 10 h and strongly decreased between 10 and 36 h. (Fig. 5b; Additional file 1: Table S4). In CG, XDH was equally low expressed at all sample points. According to homology analysis encoded by RHOT149455, XK was poorly expressed (Additional file 1: Table S4) at all sample points in both media without any significant differential expression, as observed in a previous study [22]. Contradictory, a phosphoketolase gene (RHOT147705) was significantly higher transcribed (*p* < 0.05) in CG 10 h than in CGHH 10 h. Tiukova et al. [42] proposed that with excess carbon, the reaction catalyzed by phosphoketolase might be unnecessary, resulting in most xylulose-5-P entering the pentose phosphate pathway (PPP). However, RHOT146580, encoding ribulose-phosphate 3-epimerase, was also expressed but with no significant differences between media and sample points. Jagtap et al. [22, 44] suggested that *R. toruloides* might use an alternative route for xylose utilization, in which xylulose is converted to D-arabitol rather than to xylulose-5-phosphate by the activity of XK. An enzyme catalyzing this alternative reaction is D-arabinitol 4-dehydrogenase. The genome harbors a gene coding for arabinitol 4-dehydrogenase (*LAD1*, RHOT144154). It was transcribed highest in CGHH 10 h, and the expression decreased with time (Fig. 5b; Additional file 1: Table S4). The differences were also significant between CGHH 10 h and all the timepoints in CG. However, whether it can act on D- and L-arabinitol remains unclear. *ARD1* (RHOT146692), which encodes D-arabinitol 2-dehydrogenase, was also higher transcribed in CGHH 10 h compared to CG 10 h but without statistical significance. Its expression decreased with time in CGHH. *ARD1* could be involved in the formation of D-ribulose from D-arabinitol. These results agree with the proposition from Jagtap et al. [22, 44], though Ribulokinase (RHOT145356) was expressed at an even lower level and not significantly different.

Five genes that may be important in the degradation of aromatic compounds were clearly expressed in CGHH 10 h, while there was only weak expression in CG (Fig. 5f; Additional file 1: Table S4). Aromatic monomers could originate from the lignin and thus reside in the hemicellulose portion of CGHH.

The highest number of upregulated genes involved in lipid metabolism was found at 10 h of cultivation, with higher levels in CGHH (Fig. 4a; Additional file 1: Fig. S5). The gene encoding acetyl-CoA synthetase (ACS, RHOT148257) was upregulated in CGHH 10 h compared to CG 10 h. RHOT148257 was also upregulated in CGHH 10 h compared to all other measuring points, in both CGHH and CG. This acetate-converting enzyme is part of the acetate assimilation pathway and was likely upregulated, since cells at this point were consuming the acetic acid present in the HH (Fig. 1b) [13, 45]. At later timepoints, when acetate was also no longer detected in the medium, the transcription of this gene was downregulated compared to 10 h. Interestingly, its expression in CG 10 h was relatively high, about half that in CGHH 10 h, even though no acetate was present in the cultivation medium. Here, too, the gene was downregulated with increasing cultivation time. Acetate could originate as a secondary metabolite from other metabolic pathways associated with glycerol assimilation. ATP-dependent citrate lyase (ACL, RHOT147175), thought to be the main producer of acetyl-CoA in FA synthesis [46, 47], was downregulated in CGHH 10 h compared to CG 10 h. High levels of cytoplasmic acetyl CoA produced by ACS could affect the expression level of ACL. A previous study also showed reduced expression of *ACL1* in *R. toruloides* grown on YP supplemented with acetate compared to when supplemented with glucose [22]. At later timepoints, higher transcription levels were found in CGHH (Fig. 5d; Additional file 1: Table S4). The expression of acetyl CoA carboxylase (RHOT148968) showed no significant differences between media or timepoints. The FA synthase genes *RtFAS1* (RHOT148939) and *RtFAS2* (RHOT146383) were transcribed at low levels in both substrates, particularly in CGHH, upon 10 h of cultivation, but without significant differences between the media. In contrast, two genes involved in fatty acid (FA) biosynthesis, 3-ketoacyl-acyl carrier protein reductase (FabG, RHOT148056) and enoyl carrier protein reductase (RHOT148822), were significantly upregulated in CGHH 10 h compared to CG 10 h. Transcription of these genes declined at later timepoints in CGHH down to levels comparable to those in CG (Fig. 5d; Additional file 1: Table S4). A diglyceride acyltransferase encoding gene (RHOT149017), involved in triacylglycerol biosynthesis, was also upregulated in CGHH 10 h compared to CG 10 h, and its expression significantly decreased with cultivation time in CGHH. Upregulation of this enzyme on acetate-containing medium has previously been observed elsewhere [22]. RHOT147182, which codes for a putative acyl-CoA desaturase, had very low TPM values in CGHH 10 h compared to the other conditions and was downregulated in CGHH compared to CG 10 h. In addition, ten genes involved in FA degradation, about half of the genes being mitochondrial and the other half being peroxisomal, were significantly upregulated in CGHH 10 h compared to CG 10 h. FA accumulation is considered higher at later timepoints when there is nitrogen or phosphate limitation but a surplus of carbon [21, 27]. FA degradation at earlier growth stages could be related to an increase in released FA through autophagy processes triggered by glucose depletion [48]. Mitochondrial NADP^+^-specific ICDH was the only enzyme coding gene involved in NADPH-generation whose transcription differed significantly between media at 10 h.

The catabolic L-glycerol 3-phosphate (G3P) pathway, involving glycerol kinase (*GUT1*) and FAD-dependent glycerol-3-phosphate dehydrogenase (*GUT2*), is used by *Saccharomyces cerevisiae* as the main assimilation pathway for glycerol as demonstrated by deletion studies targeting *GUT1* and *GUT2* [49, 50]. Another proposed pathway in yeast is the catabolic dihydroxyacetone (DHA) pathway. It is performed by glycerol dehydrogenase (GDH) and DHA kinase (DAK) [51, 52]. A third pathway, termed the catabolic glyceraldehyde (GA) pathway, has been proposed for *Neurospora crassa*. Here, the glycerol is first oxidized by an NADP^+^-dependent GDH to GA, which is then either phosphorylated by a GA kinase to GA-3-P or reduced by an aldehyde dehydrogenase to D-glycerate. A glycerate 3-kinase then converts the D-glycerate to 3-P-D-glycerate [51, 53, 54].

At 10 h of cultivation, transcripts of two putative glycerol transporters (*STL1*, RHOT147915, and *GPU1,* RHOT144353) were more abundant in CG than in CGHH. Enzymes belonging to the catabolic G3P pathway were transcribed under all conditions without significant differences. Enzymes belonging to the catabolic DHA pathway were also expressed, indicating the presence and expression of alternative pathways of glycerol assimilation in *R. toruloides*. The genome harbors two NADP^+^-dependent glycerol dehydrogenase genes (*GCY1*-homologs), RHOT14370 and RHOT144361, that convert glycerol to DHA. They were highest expressed in CGHH 10 h. Transcription levels decreased with time, except for RHOT14370 in CG, where the level increased. This enzyme was previously described as involved mostly in glycerol anabolic reactions [51]. The genome also encodes a DHA kinase 2 homolog, alternative name glycerone kinase 2 (*DAK2*, RHOT142321), which phosphorylates both DHA and GA, indicating that it may also be involved in the GA pathway in addition to the catabolic DHA pathway. *DAK2* expression decreased in the 60 h samples in both media compared to the earlier timepoints. However, there were no significant differences between the conditions and further investigations are required to confirm this tendency. The genome harbors an Alcohol dehydrogenase [NADP(+)] gene (*ARI*, RHOT147125), whose encoding protein was found to have 54% sequence identity to NADP^+^-dependent GDH from *Trichoderma reesei* (ABD83952.1), besides 100% identity to XR from *R. toruloides* NP11. *ARI* could have mediated the conversion of glycerol to GA, which represents the first step of the catabolic GA pathway, in addition to its role in xylose metabolism [51]. *ARI* was transcribed under all conditions, and the transcription levels decreased with time (Fig. 5c). It was higher transcribed in CGHH 10 h than in CG 10 h. However, the significance of these differences could not be proven, thus, further investigations are required to confirm this tendency. A variety of aldehyde dehydrogenases were expressed under both experimental conditions; however, differences in their expression could not be shown. We identified RHOT146637 as D-glycerate 3-kinase (*RTG_01831*) by performing homology analyses. It was transcribed under all conditions but without significant differences. Summarized, this suggests that *R. toruloides* can utilize all three glycerol assimilation pathways described in fungi [51]. The expression level of enzymes belonging to the DHA catabolic pathway could account for the differences in glycerol assimilation between cells grown in CGHH and CG after 10 h.

Several genes involved in handling oxidative stress were upregulated in CGHH at 10 h, including three thioredoxin genes (RHOT143685, 7078, and 3176). However, some stress-related genes were also upregulated in CG, including a catalase gene (RHOT141031) (Fig. 5e; Additional file 1: Table S4).

**Fig. 6:**
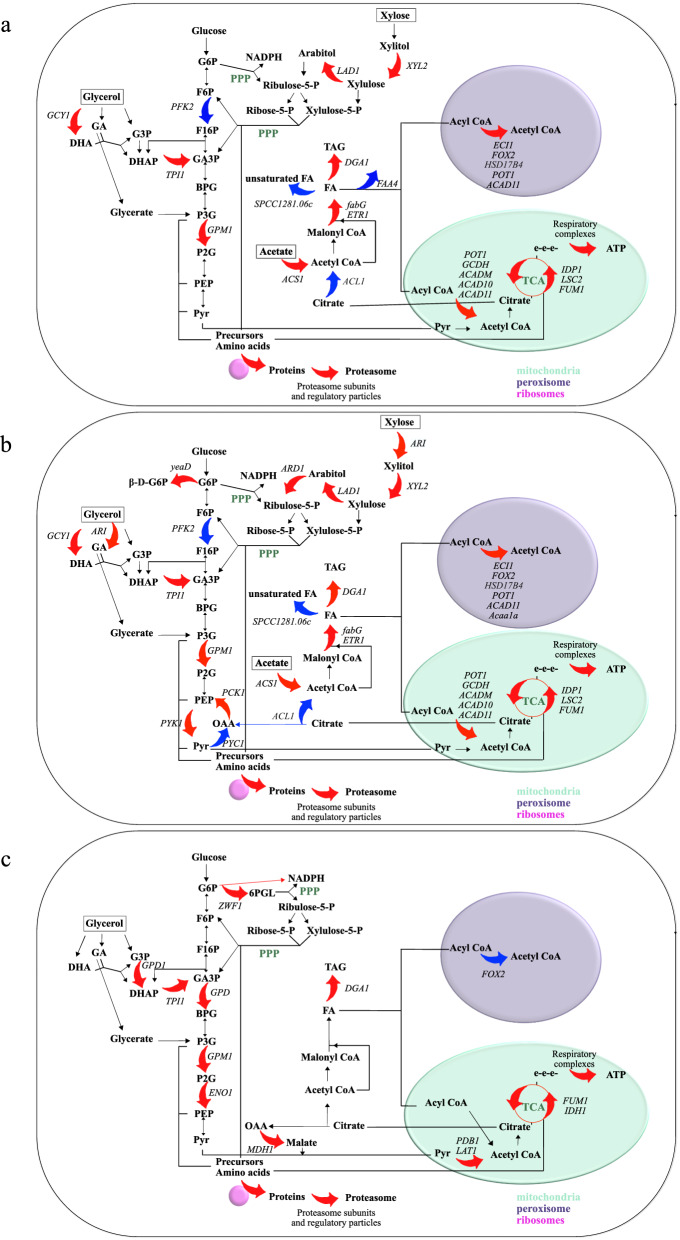
Differentially regulated central metabolic pathways in *R. toruloides* CBS14. Genes expressed in cells grown on a mixture of crude glycerol (CG) and hemicellulose hydrolysate (CGHH) were compared to cells grown in CG as only carbon source: **a** 10 h versus 10 h **b** 10 versus 30 h and **c** 36 h versus 60 h, respectively. Red arrows indicate upregulated genes (adjusted *p* value < 0.05) in CGHH, while blue arrows indicate downregulated genes (adjusted *p* value < 0.05). Genes that are expressed without significant differences are represented with black arrows

Central metabolic pathways that were differentially regulated at the 10 h sampling point are represented in Fig. 6a.

### The number of differentially expressed genes was highest when glycerol consumption became visible

Of the 787 differentially expressed genes in CGHH 10 h, 585 were upregulated in CG 30 h (Fig. 3b; Additional file 1: Table S2). Central metabolic pathways that were differentially regulated are shown in Fig. 6b. The pathways with a higher number of differentially expressed genes were energy, carbohydrate, and amino acid metabolism, in descending order (Fig. 4b). These mainly were upregulated in CGHH 10 h, similar as when compared to the earlier timepoint in CG.

27 genes associated with oxidative phosphorylation were upregulated in CGHH 10 h (Additional file 1: Fig. S8a) compared to CG 30 h. In addition, the upregulated genes at 10 h cultivations which encoded mitochondrial enzymes or components, such as prohibitin, NADP^+^-specific ICDH, succinate-CoA ligase, fumarate hydratase, riboflavin synthase, lipoyl synthase, and thiamine pyrophosphokinase, were still upregulated in CGHH 10 h when compared to CG 30 h.

Differences in the transcription of glycolytic genes included the downregulation of *PFK2* and upregulation of *GPM1* and *TPI1* in CGHH, similarly as described at 10 h comparison. A gene encoding pyruvate kinase (RHOT144157) was upregulated in CGHH. Phosphoenolpyruvate carboxykinase gene (*PCK1*, RHOT144519), which can be linked with gluconeogenic reactions, was also upregulated in CGHH, while pyruvate carboxylase gene (*PYC1*, RHOT149202) was downregulated. *PCK1* was previously found to be upregulated during growth of *R. toruloides* on acetate [22]. Another upregulated gene involved in carbohydrate metabolic processes encodes a putative glucose-6-phosphate 1-epimerase (RHOT149194). Of the described differentially expressed genes involved in xylose assimilation in *R. toruloides*, *ARI*, the aldo/keto reductases RHOT148868 and RHOT14370, *XYL2*, *LAD1,* and *ARD1* were upregulated in CGHH (Fig. 6b), which suggests an increased flux via arabitol.

The processes of folding, sorting and degradation, and translation also had a high number of upregulated genes in CGHH 10 h compared to CG 30 h (Fig. 4b). Transcription of 46 ribosomal proteins (Additional file 1: Fig. S8b), five RNA polymerases, and 20 genes associated with the spliceosome was upregulated in CGHH. There were no downregulated genes whose functions are involved in transcription. In addition, the transcription of genes involved in protein degradation was activated in CGHH. Transcription of 19 genes involved in proteasome assembly was upregulated (Additional file 1: Fig. S8c), while none was downregulated in CGHH.

The highest number of upregulated genes involved in lipid metabolism was also found on the carbon source CGHH at these timepoints (Fig. 4b). In a similar pattern to that described in the comparison of the 10 h sample points, transcription of the genes encoding for ACS, FabG, enoyl carrier protein reductase, and diglyceride acyltransferase was upregulated in CGHH. ACL and probable acyl-CoA desaturase were downregulated, and there were no significant differences between media for *ACC1*, *RtFAS1,* and *RtFAS2.* Twelve genes involved in FA degradation were significantly upregulated in CGHH, about six of which were peroxisomal. Transcription of genes encoding enzymes involved in cytosolic NADPH-generation did not differ significantly between media when glycerol consumption became visible.

The genes involved in glycerol assimilation which were differentially expressed when comparing CGHH 10 h with CG30 h were two *GCY1* genes and *ARI.* They might be associated with the catabolic pathways via DHA and GA, respectively.

Several genes related to oxidative stress management were also upregulated in CGHH at 10 h compared to CG 30 h, including the three thioredoxin genes described above and a glutathione-S transferase (RHOT149349).

### The depletion of additional carbon sources induces changes in glycerol utilization and carbohydrate pathways

311 of 632 differentially expressed genes were higher transcribed in CGHH 36 h than in CG 60 h (Fig. 3c; Additional file 1: Table S3). Central metabolic pathways that were differentially expressed are shown in Fig. 6c. The cellular process with the highest number of upregulated genes was signal transduction in CGHH (Fig. 4c). Carbohydrate metabolism was the pathway with a higher number of differentially expressed genes, with a more prominent expression level on the CGHH carbon source.

Transcription of seven genes associated with Glycolysis and Gluconeogenesis was upregulated in CGHH, while none was downregulated. Besides *TPI1* and *GPM1,* the other upregulated genes were an aldehyde dehydrogenase (RHOT148569), glyceraldehyde 3-phosphate dehydrogenase (RHOT147990), pyruvate dehydrogenase E1 component subunit beta (RHOT14206), pyruvate dehydrogenase complex component E2 (RHOT146289), and enolase (RHOT142969). Another upregulated gene involved in carbohydrate metabolic processes was that for glucose-6-phosphate 1-dehydrogenase (RHOT146681), which provides NADPH and pentose phosphates for the synthesis of FA and nucleic acids.

There were few significant differences in gene expression related to lipid metabolism between CGHH 36 h and CG 60 h. The exceptions were diglyceride acyltransferase, involved in triacylglycerol biosynthesis, and the peroxisomal multifunctional beta-oxidation protein (RHOT144031), involved in FA degradation. They were upregulated in CGHH and CG, respectively. Interestingly, *RtFAS21* (RHOT146384), which forms an antisense RNA [26], was expressed in CG 60 h.

25 genes associated with oxidative phosphorylation were upregulated in CGHH 36 h (Additional file 1: Fig. S9a) compared to CG 60 h. Other genes encoding mitochondrial enzymes or associated components still upregulated in CGHH when glycerol was the sole carbon source were prohibitin-2, NAD^+^-specific ICDH (RHOT14435), fumarate hydratase, DHBP synthase, and lipoyl synthase. A cytoplasmic malate dehydrogenase (RHOT147988) was also upregulated in CGHH.

There were two RNA polymerase genes (DNA-directed RNA polymerase I subunit rpa1 and DNA-directed RNA polymerase II subunit rpb1) that were downregulated and none upregulated in cultivations with CGHH as carbon source. However, the transcription of 22 ribosomal proteins (Additional file 1: Fig. S9b) was upregulated. In addition, the transcription of genes involved in protein degradation was activated in CGHH, including seven genes involved in proteasome assembly (Additional file 1: Fig. S9c).

At these timepoints, *GCY1* and *ARI* transcription, possibly involved in glycerol and xylose metabolism, were no longer significantly different between cultivations. However, expression of an NAD^+^-dependent glycerol-3-phosphate dehydrogenase (RHOT141674) was upregulated in CGHH, generating NADH along with glycerol catabolism. NADH could then be transported to the mitochondria by the malate dehydrogenase shuttle or *GUT2.*

## Discussion

A considerable transcriptional upregulation of genes involved in oxidative phosphorylation and of several mitochondrial enzymes was observed in CGHH 10 h cultures compared to CG 10 h and CG 30 h (Fig. 4a, b). For some genes, this effect was still visible even after longer cultivation, when the additional carbon sources originating from the HH were already consumed, and the remaining glycerol concentrations were similar, as especially shown for CGHH 36 h compared to CG 60 h (Figs. 4, 5, 6). Energy metabolism could thus have been activated in response to the HH addition. High availability of ATP is of central importance for forming biomass and lipids, as well as for efficient uptake of limiting resources, such as nitrogen and for inhibitor tolerance [55].

Among the mitochondrial enzymes, lower transcription of the NADP^+^-specific ICDH and succinate-CoA ligase genes was observed in CGHH 36 h compared to CG 60 h, although significance could not be demonstrated. Further studies are required to confirm this tendency. Nevertheless, this would be consistent with the specific increase in lipid production observed during the growth on glycerol supplemented with HH [13]: the inhibition of ICDH is a central regulatory element in lipid accumulation [56] in oleaginous yeasts and succinate-CoA ligase catalyzes the subsequent reaction in the TCA-cycle. On the other hand, we found an upregulated NAD^+^-specific ICDH in CGHH 36 h compared to CG 60 h. Which cofactor, NAD^+^ or NADP^+^ is used by the ICDH may have different physiological consequences for the cell [57]. During the early growth phase in CGHH, the generated NADPH might provide biosynthetic reducing power needed to promote cell growth and stress responses. In the later growth phase, around 36 h, the ICDH may have primarily provided an energy source in the form of NADH.

Transcription of genes involved in protein turnover was largely upregulated in the CGHH culture, especially at the beginning of growth (Fig. 4a, b). Probably, this might be due to the temporary presence of various carbon sources and other compounds present in HH, which require establishment and later inactivation of metabolic pathways.

Total glycerol consumption took about 48 h less in the culture with HH than in the cultures without HH (Fig. 1). No upregulation of genes involved in glycerol assimilation via the catabolic G3P pathway was observed in CGHH when sampled after 10 h. A potential bypass of the standard *GUT1* and *GUT2* pathway, as described by Klein et al. [51], was expressed, similar to the DHA pathway demonstrated in *S. pombe* [52]. We found two upregulated GDH genes in CGHH that were annotated as NADP^+^-dependent. These were initially upregulated in CGHH-cultivated cells (Fig. 6). They might represent additional pathways to direct glycerol into main metabolic pathways while providing NADPH to biosynthetic pathways, including FA synthesis. Moreover, genes belonging to the catabolic GA pathway proposed in *N. crassa* [51, 53, 54] were also expressed. To our knowledge, this is the first indication that the catabolic GA pathway for glycerol assimilation was identified in a basidiomycete and a yeast. However, further investigations are required to confirm the existence of this pathway in *R. toruloides*.

In addition, *TPI1* was upregulated in CGHH compared to cells grown in CG in the same physiological situation (i.e., CGHH 10 h and CG 10 h, CGHH 10 h and CG 30 h, and CGHH 36 h and CG 60 h), possibly directing the DHAP formed by glycerol catabolism to glycolysis. At 10 h cultivations, the expression level of genes encoding glycolytic enzymes was similar under both growth conditions, i.e., with or without the addition of HH, except for *PFK2* and *GPM1*. This might be due to the fact that glucose was rapidly depleted in CGHH. After 36 h in CGHH, the number of upregulated glycolytic genes increased compared to CG 60 h.

When the cells grew in CGHH, they simultaneously assimilated acetic acid and glucose, which supplied the cell with additional amounts of acetyl-CoA for FA synthesis [58, 59]. ACS was significantly more highly transcribed after 10 h in CGHH compared to CGHH 60 h and CG 60 h. This points to a higher acetyl-CoA production required for the slightly enhanced lipid synthesis observed in CGHH [13]. Both acetate assimilation and alternative glycerol degradation pathway may provide the additional acetyl-CoA and NADPH, respectively, that would have been required for the enhanced lipid synthesis seen in CGHH [13].

However, cells harvested after 10 h in CGHH showed higher transcription of genes involved in lipid degradation and lower transcription of genes involved in FA production compared to later timepoints in CGHH and CG. Activation of genes involved in β-oxidation has previously been observed upon cultivation on xylose and under nitrogen-limiting and lipid-accumulating conditions [42]. In the closely related species *R. glutinis*, we even observed a breakdown of the cell's intracellular lipids with simultaneous xylose assimilation [27].

During xylose assimilation, *XYL2* and *LAD1* were upregulated in CGHH 10 h compared to CG 10 h and CG 30 h, suggesting an increased flux via arabitol. These expression differences disappeared when xylose was exhausted after 36 h in CGHH. It has been shown that the growth on xylose appears to correlate with the induction of several genes involved in oxidative stress response [42]. Likewise, in this study, xylose appears to trigger stress responses, particularly in CGHH, after 10 h of cultivation, although the reason remains unclear. However, the presence of acetic acid in our cultivation might also have contributed to the activation of the oxidative stress response. The addition of acetic acid led to the production of reactive oxygen species in *S. cerevisiae* [60].

Activation of energy-yielding enzymes, meaning those enzymes involved in oxidative phosphorylation and mitochondrial function, appears to be the primary physiological cause of accelerated glycerol assimilation and lipid production.

In addition to providing additional carbon sources, the substrate itself could also have led to the induction of respiratory genes as a response to stress (for example, due to the presence of aromatic compounds in the HH). In this context, we have observed the activation of genes involved in the degradation of aromatic compounds. Aromatic compounds can be metabolized by *Rhodotorula* yeasts [61] as a source of carbon, but they are also toxic at the same time. Due to the pre-treatment of the lignocellulose, soluble aromatic compounds may have been present in small amounts in the HH fraction [62] and thus have been involved in stress response. In the presence of HH, the energy demand of the cells could be higher, which could explain the activation of the β-oxidation in CGHH 10 h compared to CG at 10 and 30 h (Fig. 6). Once the substrate has been detoxified, the cells switch to glycerol metabolism and lipid accumulation suggested by the upregulation of genes (*p* < 0.05) involved in these pathways in CGHH (Fig. 6). These transcriptional changes could have been enabled by the cells having a sufficiently high ATP level and, therefore, sufficient energy, since a high number of respiratory genes were upregulated (*p* < 0.05) in CGHH compared to CG in the three similar physiological situations. This could be further supported by the HH-induced increased protein synthesis and turnover, which enabled the cells to adapt efficiently to the changing carbon source.

## Conclusions

There is no direct proportionality between transcription level and enzyme activity. The transcriptome is only one part of a cell's information chain, and such changes do not necessarily equate to metabolic pathways and enzyme activities [63, 64]. Nevertheless, the observation of the transcription of a large number of genes does allow some valuable conclusions to be drawn about metabolic and signaling pathways. In this case, it provides hypotheses about the molecular mechanisms triggered by a small amount of HH added to CG medium, which lead to the faster initial glycerol consumption and higher lipid accumulation.

In this study, we observed enhanced transcription of genes involved in oxidative phosphorylation and enzymes localised in mitochondria in CGHH compared to CG. Genes involved in protein turnover, including those encoding ribosomal proteins, translation elongation factors and genes involved in building the proteasome also showed an enhanced transcription in CGHH. In the presence of the HH, β-oxidation was activated. We suspect that the physiological reason for the accelerated glycerol assimilation and faster lipid production, was primarily the activation of enzymes involved in providing energy to the cells.

Our study paves the way for further detailed investigations of such underlying mechanisms. It also helps to identify new targets to obtain strains that can more rapidly accumulate lipids from residual, low-value substrates.

## Supplementary Information

**Additional file 1:** **Table S1.** Differentially expressed genes between growth on different media at 10 h. **Table S2.** Differentially expressed genes between growth on different media when glycerol consumption became visible. **Table S3.** Differentially expressed genes between growth on different media when glycerol concentration is lower. **Table S4.** Gene expression in TPM within central metabolic pathways. **Figure S1.** Principal Component Analysis based on expression level from the 500 highest expressed genes in each of the cultivation media and RNA sampling points. **Figure S2.** Overview of differential expression analysis between Rhodotorula toruloides CBS 14 cultivated on crude glycerol and a mixture of crude glycerol and hemicellulose hydrolysate, at 10 h cultivation. **Figure S3.** Overview of differential expression analysis between Rhodotorula toruloides CBS 14 grown on different media when glycerol consumption became visible. **Figure S4.** Overview of differential expression analysis between Rhodotorula toruloides CBS 14 grown on different media when about 20 g/l of glycerol were left. **Figure S5.** Differentially expressed genes in Rhodotorula toruloides CBS 14 within sampling time points in each of the growth media. **Figure S6.** Examples of upregulated genes in Rhodotorula toruloides CBS 14 grown on a mixture of crude glycerol (CG) and hemicellulose hydrolysate (CGHH) for 10 h of cultivation, whose expression is higher than CG 10 h. **Figure S7.** Examples of upregulated genes in Rhodotorula toruloides CBS 14 grown on a mixture of crude glycerol (CG) and hemicellulose hydrolysate (CGHH) for 10 h of cultivation, whose expression is higher than CG 30 h. **Figure S8.** Examples of upregulated genes in Rhodotorula toruloides CBS 14 grown on a mixture of crude glycerol (CG) and hemicellulose hydrolysate (CGHH) for 36 h of cultivation, whose expression is higher than CG 60 h. **Figure S9.** Distribution of shared ortholog clusters within differentially expressed genes in each of the three differential expression analyzes. The diagram was generated using OrthoVenn2.

## Data Availability

The genome assembly and annotation of *R. toruloides* CBS14, as well as BAM mapping files and transcript counts, are available at https://osf.io/yzqn5. Raw RNA reads are available in ENA under the project PRJEB40807.
