# Supplementary material for: Enhanced glycerol assimilation and lipid production in Rhodotorula toruloides CBS14 upon addition of hemicellulose primarily correlates with early transcription of energy-metabolism-related genes
Source: Biotechnol Biofuels Bioprod. 2023 Mar 10;16:42. doi: 10.1186/s13068-023-02294-3 (PMC9999650; doi:10.1186/s13068-023-02294-3)
Supplement: Supplementary file 1 — Additional file 1: Table S1. Differentially expressed genes between growth on different media at 10 h. Table S2. Differentially expressed genes between growth on different media when glycerol consumption became visible. Table S3. Differentially expressed genes between growth on different media when glycerol concentration is lower. Table S4. Gene expression in TPM within central metabolic pathways. Figure S1. Principal Component Analysis based on expression level from the 500 highest expressed genes in each of the cultivation media and RNA sampling points. Figure S2. Overview of differential expression analysis between Rhodotorula toruloides CBS 14 cultivated on crude glycerol and a mixture of crude glycerol and hemicellulose hydrolysate, at 10 h cultivation. Figure S3. Overview of differential expression analysis between Rhodotorula toruloides CBS 14 grown on different media when glycerol consumption became visible. Figure S4. Overview of differential expression analysis between Rhodotorula toruloides CBS 14 grown on different media when about 20 g/l of glycerol were left. Figure S5. Differentially expressed genes in Rhodotorula toruloides CBS 14 within sampling time points in each of the growth media. Figure S6. Examples of upregulated genes in Rhodotorula toruloides CBS 14 grown on a mixture of crude glycerol (CG) and hemicellulose hydrolysate (CGHH) for 10 h of cultivation, whose expression is higher than CG 10 h. Figure S7. Examples of upregulated genes in Rhodotorula toruloides CBS 14 grown on a mixture of crude glycerol (CG) and hemicellulose hydrolysate (CGHH) for 10 h of cultivation, whose expression is higher than CG 30 h. Figure S8. Examples of upregulated genes in Rhodotorula toruloides CBS 14 grown on a mixture of crude glycerol (CG) and hemicellulose hydrolysate (CGHH) for 36 h of cultivation, whose expression is higher than CG 60 h. Figure S9. Distribution of shared ortholog clusters within differentially expressed genes in each of the three differenti [file 13068_2023_2294_MOESM1_ESM.docx]

**Supplementary information**

Enhanced glycerol assimilation and lipid production in *Rhodotorula toruloides* CBS14 upon addition of hemicellulose primarily correlates with early transcription of energy-metabolism related genes

Giselle C. Martín-Hernandez^a,†^, Mikołaj Chmielarz^a,†^, Bettina Müller^a^, Christian Brandt^b^, Adrian Viehweger^c^, Martin Hölzer^d^, Volkmar Passoth^a,*^

^a^Swedish University of Agricultural Sciences, Department of Molecular Sciences, BioCenter, Box 7015, 75007 Uppsala, Sweden

^b^Institute for Infectious Diseases and Infection Control, Jena University Hospital, Jena, Germany

^c^Institute of Medical Microbiology and Virology, University Hospital Leipzig, 04103 Leipzig, Germany

^d^Method Development and Research Infrastructure, Bioinformatics and Systems Biology, Robert Koch Institute, 13353 Berlin, Germany

*Corresponding author: [Volkmar.passoth@slu.se](mailto:Volkmar.passoth@slu.se)

†These authors contributed equally to this work.

Table S1: Differentially expressed genes between growth on different media at 10 h

Table S2: Differentially expressed genes between growth on different media when glycerol consumption became visible

Table S3: Differentially expressed genes between growth on different media when glycerol concentration is lower

Table S4: Gene expression in TPM within central metabolic pathways

Figure S1: Principal Component Analysis based on expression level from the 500 highest expressed genes in each of the cultivation media and RNA sampling points

Figure S2: Overview of differential expression analysis between *Rhodotorula toruloides* CBS 14 cultivated on crude glycerol and a mixture of crude glycerol and hemicellulose hydrolysate, at 10 h cultivation

Figure S3: Overview of differential expression analysis between *Rhodotorula toruloides* CBS 14 grown on different media when glycerol consumption became visible

Figure S4: Overview of differential expression analysis between *Rhodotorula toruloides* CBS 14 grown on different media when about 20 g/l of glycerol were left

Figure S5: Differentially expressed genes in *Rhodotorula toruloides* CBS 14 within sampling time points in each of the growth media.

Figure S6: Gene Ontology term summaries belonging to the GO topic: biological processes

Figure S7: Examples of upregulated genes in *Rhodotorula toruloides* CBS 14 grown on a mixture of crude glycerol (CG) and hemicellulose hydrolysate (CGHH) for 10 h of cultivation, whose expression is higher than CG 10 h.

Figure S8: Examples of upregulated genes in *Rhodotorula toruloides* CBS 14 grown on a mixture of crude glycerol (CG) and hemicellulose hydrolysate (CGHH) for 10 h of cultivation, whose expression is higher than CG 30 h.

Figure S9: Examples of upregulated genes in *Rhodotorula toruloides* CBS 14 grown on a mixture of crude glycerol (CG) and hemicellulose hydrolysate (CGHH) for 36 h of cultivation, whose expression is higher than CG 60 h.

Table S1: Differentially expressed genes between growth on different media at 10 h

| **Gene ID** | **baseMean** | **log2FoldChange** | **lfcSE** | **pvalue** | **padj** |
| --- | --- | --- | --- | --- | --- |
| NANOZOOG825 | 11,1852881 | -8,8491887 | 2,83844349 | 2,2255E-10 | 2,9281E-08 |
| NANOZOOG827 | 5,87716147 | -7,6658954 | 2,7541767 | 1,0892E-07 | 6,0429E-06 |
| NANOZOOG7104 | 13,6400338 | 7,12779809 | 2,44614387 | 3,2874E-06 | 0,00011735 |
| NANOZOOG826 | 4,07082745 | -6,7162258 | 2,61794704 | 1,374E-06 | 5,5995E-05 |
| NANOZOOG3179 | 104,649965 | 6,63689911 | 2,20457253 | 2,0977E-06 | 8,1862E-05 |
| NANOZOOG5335 | 5,73694102 | 6,45620136 | 2,45609974 | 5,4984E-06 | 0,00018086 |
| NANOZOOG387 | 4,10252047 | -6,4507305 | 2,52940868 | 1,0116E-06 | 4,2348E-05 |
| NANOZOOG2859 | 43,548459 | 6,33003419 | 2,54553348 | 6,1932E-05 | 0,00130825 |
| NANOZOOG5806 | 5,60716221 | 6,31493248 | 2,27059816 | 2,6561E-06 | 9,7849E-05 |
| NANOZOOG4366 | 6,0826476 | 6,29510152 | 2,49897375 | 3,1203E-05 | 0,00075276 |
| NANOZOOG350 | 6,79355868 | 6,13551289 | 2,21640659 | 3,6739E-06 | 0,00013014 |
| NANOZOOG8116 | 5,31130146 | 5,69893599 | 2,29674538 | 3,3107E-05 | 0,00078587 |
| NANOZOOG5970 | 11,455005 | -5,5296689 | 1,02915813 | 7,4605E-08 | 4,5205E-06 |
| NANOZOOG8056 | 22,7808221 | -5,5102891 | 0,70464777 | 1,9316E-15 | 7,4124E-13 |
| NANOZOOG1031 | 5,04532831 | 5,47672572 | 2,54669373 | 0,00023812 | 0,00391617 |
| NANOZOOG7955 | 5,28554133 | 5,47520873 | 2,26612024 | 4,4583E-05 | 0,00101353 |
| NANOZOOG370 | 4,01663461 | -5,445739 | 2,50834372 | 5,6382E-05 | 0,00120762 |
| NANOZOOG831 | 5,05141496 | -5,3567154 | 1,3862419 | 5,0278E-06 | 0,00017024 |
| NANOZOOG8947 | 4,15694062 | 5,29273734 | 2,29917743 | 8,8096E-05 | 0,00177154 |
| NANOZOOG901 | 33,4395467 | -5,2850799 | 0,50416163 | 3,977E-26 | 9,157E-23 |
| NANOZOOG2231 | 37,8220243 | -5,2843948 | 0,6901195 | 1,2961E-15 | 5,4261E-13 |
| NANOZOOG2307 | 5,60395817 | 5,2198646 | 2,21860536 | 5,9932E-05 | 0,00127182 |
| NANOZOOG7869 | 11,1022052 | 4,80492945 | 1,99180715 | 4,4679E-05 | 0,00101353 |
| NANOZOOG56 | 12,6994121 | -4,8033361 | 1,02564396 | 1,9172E-06 | 7,5459E-05 |
| NANOZOOG3041 | 9,77639713 | -4,7781444 | 1,36075949 | 2,916E-05 | 0,00072332 |
| NANOZOOG8232 | 14,5412779 | -4,659355 | 0,69673349 | 6,0009E-12 | 1,0629E-09 |
| NANOZOOG8742 | 3,95562238 | -4,6255295 | 1,51315869 | 0,00012197 | 0,00231143 |
| NANOZOOG451 | 14,4194683 | 4,61495529 | 2,29012717 | 0,00041181 | 0,00581709 |
| NANOZOOG3971 | 6,34084497 | 4,61395518 | 1,34953684 | 4,968E-05 | 0,0011052 |
| NANOZOOG2281 | 16,5904486 | -4,6119585 | 0,68053501 | 2,3533E-12 | 4,875E-10 |
| NANOZOOG4877 | 33,2818588 | -4,6074378 | 0,49745043 | 3,3232E-21 | 3,0607E-18 |
| NANOZOOG1824 | 3,68107888 | -4,5861893 | 1,36420947 | 5,105E-05 | 0,00113022 |
| NANOZOOG1250 | 26,8430478 | 4,51114425 | 1,87400849 | 5,2199E-05 | 0,00114574 |
| NANOZOOG2399 | 10,4418958 | 4,44744683 | 1,95173179 | 9,6912E-05 | 0,00191377 |
| NANOZOOG7027 | 53,9811206 | 4,4017743 | 1,54947418 | 0,00019781 | 0,00333668 |
| NANOZOOG2341 | 7,93235008 | 4,39541525 | 2,35027507 | 0,00073068 | 0,00899679 |
| NANOZOOG1254 | 3,09792307 | 4,39310924 | 2,16775952 | 0,00029702 | 0,00462082 |
| NANOZOOG5007 | 15,9697811 | 4,36949357 | 1,03100329 | 1,4103E-05 | 0,00039361 |
| NANOZOOG908 | 3,49896372 | -4,3619559 | 1,76919969 | 0,00058161 | 0,0076547 |
| NANOZOOG5985 | 9,41102942 | 4,3277887 | 1,60728506 | 0,00036931 | 0,00536643 |
| NANOZOOG1159 | 7,71933027 | 4,31979728 | 2,30169465 | 0,00070283 | 0,00874744 |
| NANOZOOG7440 | 11,2774384 | 4,30200308 | 0,98810831 | 1,0423E-05 | 0,00030966 |
| NANOZOOG7323 | 10,5015246 | -4,2370137 | 0,74211423 | 3,215E-09 | 2,903E-07 |
| NANOZOOG5227 | 6,42646066 | 4,23252801 | 1,59622277 | 0,00043107 | 0,00601537 |
| NANOZOOG2057 | 7,20086197 | -4,1712267 | 2,5102151 | 0,00111134 | 0,01239158 |
| NANOZOOG8241 | 6,65944979 | 4,15723833 | 1,43536038 | 0,00028802 | 0,00449611 |
| NANOZOOG5831 | 7,91352116 | 4,14192607 | 1,3194805 | 0,00015717 | 0,00283924 |
| NANOZOOG5617 | 3,39047344 | 4,08657699 | 2,24469179 | 0,00086632 | 0,01028197 |
| NANOZOOG8336 | 7,71842985 | -4,0499473 | 0,87692025 | 6,2117E-07 | 2,7243E-05 |
| NANOZOOG2343 | 4,04856484 | 4,03993237 | 2,18196557 | 0,00073885 | 0,00904891 |
| NANOZOOG303 | 8,40449023 | 3,98590451 | 0,91975009 | 5,0003E-06 | 0,00017024 |
| NANOZOOG3288 | 17,1981762 | 3,96475662 | 1,09253421 | 2,5619E-05 | 0,00066278 |
| NANOZOOG4967 | 6,5617505 | 3,9432107 | 1,48792623 | 0,00049441 | 0,00677608 |
| NANOZOOG6040 | 3,43837025 | 3,94037553 | 2,13149044 | 0,0007272 | 0,00899679 |
| NANOZOOG2220 | 13,7061724 | 3,9114336 | 1,41168638 | 0,00036942 | 0,00536643 |
| NANOZOOG5490 | 20,0118333 | 3,87813482 | 1,3395417 | 0,00040062 | 0,00570086 |
| NANOZOOG4305 | 8,80256756 | 3,8779555 | 1,38448017 | 0,00036234 | 0,00532692 |
| NANOZOOG4484 | 9,30730228 | -3,8755834 | 0,74376186 | 4,5551E-08 | 3,0463E-06 |
| NANOZOOG3771 | 9,24167644 | 3,79325502 | 1,25798902 | 0,00025584 | 0,00410499 |
| NANOZOOG4171 | 30,0756713 | -3,7758145 | 0,42823496 | 1,2677E-19 | 8,3395E-17 |
| NANOZOOG5684 | 8,45646196 | -3,7543637 | 0,70150396 | 2,7061E-08 | 1,978E-06 |
| NANOZOOG5991 | 5,35086811 | 3,73852012 | 2,00971755 | 0,00069672 | 0,00870596 |
| NANOZOOG6010 | 4,4046335 | 3,72640202 | 1,47866343 | 0,00087606 | 0,01029153 |
| NANOZOOG1345 | 4,53427256 | -3,717655 | 1,20369277 | 0,00016141 | 0,00289213 |
| NANOZOOG52 | 22,3607474 | -3,7175592 | 0,65246512 | 1,0306E-09 | 1,0547E-07 |
| NANOZOOG9451 | 5,97305545 | -3,6878962 | 0,95852248 | 2,2347E-05 | 0,00059484 |
| NANOZOOG4483 | 18,1789856 | -3,6415133 | 0,54775907 | 3,4059E-12 | 6,5351E-10 |
| NANOZOOG9111 | 181,909701 | 3,59795881 | 0,37858887 | 5,5495E-22 | 6,3889E-19 |
| NANOZOOG6137 | 5,61077259 | -3,5952857 | 1,07738373 | 0,00022867 | 0,00380153 |
| NANOZOOG4298 | 26,3973025 | 3,58906883 | 0,58209779 | 2,445E-10 | 3,043E-08 |
| NANOZOOG7925 | 3,19684054 | 3,5553398 | 1,50182588 | 0,00139229 | 0,01487591 |
| NANOZOOG5209 | 24,1999875 | 3,55020931 | 1,1524511 | 0,00011893 | 0,00227245 |
| NANOZOOG1128 | 7,00362487 | 3,53899054 | 2,10581521 | 0,0013977 | 0,0148991 |
| NANOZOOG7037 | 5,57069085 | -3,5362905 | 0,89093512 | 1,1071E-05 | 0,0003268 |
| NANOZOOG2835 | 34,1463742 | 3,52272591 | 0,96851158 | 2,2219E-05 | 0,00059484 |
| NANOZOOG7247 | 23,7754943 | 3,51370704 | 1,09298153 | 0,00014835 | 0,00271662 |
| NANOZOOG4610 | 9,66274566 | -3,510153 | 0,75742709 | 4,5136E-07 | 2,0785E-05 |
| NANOZOOG6880 | 25,5543312 | 3,49452219 | 1,09050223 | 0,00011739 | 0,00225234 |
| NANOZOOG4921 | 9,97315954 | 3,48607081 | 1,29885006 | 0,00087549 | 0,01029153 |
| NANOZOOG7324 | 128,578775 | 3,48168333 | 0,75256444 | 2,233E-07 | 1,13E-05 |
| NANOZOOG6139 | 5,3060325 | 3,48147902 | 2,01883043 | 0,0012117 | 0,01322244 |
| NANOZOOG1136 | 28,9894185 | 3,48015582 | 0,93565009 | 1,361E-05 | 0,00038858 |
| NANOZOOG4496 | 14,5242335 | 3,47910863 | 1,28722958 | 0,00061879 | 0,00800816 |
| NANOZOOG7781 | 6,12350139 | 3,47485145 | 2,02299826 | 0,0012257 | 0,01334363 |
| NANOZOOG4175 | 31,2366973 | -3,4352473 | 0,42157036 | 4,931E-17 | 2,2707E-14 |
| NANOZOOG5984 | 11,0806502 | 3,42819698 | 1,55380781 | 0,00213617 | 0,02070964 |
| NANOZOOG7907 | 16,4348716 | 3,41992726 | 1,17157673 | 0,00034661 | 0,00521608 |
| NANOZOOG6006 | 8,41460271 | -3,4144464 | 0,84539922 | 1,1934E-05 | 0,00034784 |
| NANOZOOG8818 | 3,52151501 | -3,3838055 | 1,1560654 | 0,00055409 | 0,00741737 |
| NANOZOOG3297 | 23,0113738 | 3,37677922 | 1,33293785 | 0,00065199 | 0,00834009 |
| NANOZOOG9037 | 6,25749743 | -3,3566719 | 0,84463248 | 1,4677E-05 | 0,00040472 |
| NANOZOOG4489 | 37,630092 | 3,35620942 | 1,22528671 | 0,00033075 | 0,00502673 |
| NANOZOOG3928 | 5,4772569 | 3,30218515 | 1,4269604 | 0,00147419 | 0,01549918 |
| NANOZOOG3518 | 15,696387 | 3,28366673 | 0,55546615 | 5,4304E-10 | 6,2518E-08 |
| NANOZOOG4508 | 11,1320364 | 3,28355779 | 1,22285701 | 0,00101494 | 0,01154023 |
| NANOZOOG6753 | 5,41694698 | 3,28312391 | 1,31220106 | 0,00112659 | 0,01253129 |
| NANOZOOG8649 | 5,15572143 | 3,28134581 | 2,03679759 | 0,00176726 | 0,01776902 |
| NANOZOOG7936 | 10,1059002 | 3,25836509 | 0,65434814 | 8,7728E-08 | 5,1793E-06 |
| NANOZOOG5774 | 7,33724706 | 3,24884156 | 2,5588492 | 0,00401727 | 0,03339266 |
| NANOZOOG5154 | 7,0747515 | -3,2472043 | 0,89233962 | 3,763E-05 | 0,00087519 |
| NANOZOOG8467 | 7,38476691 | 3,24087322 | 1,99705697 | 0,001689 | 0,01716967 |
| NANOZOOG6292 | 8,53547606 | 3,23587236 | 1,97903854 | 0,00162839 | 0,01670096 |
| NANOZOOG1365 | 3,55407649 | 3,19633125 | 2,23522725 | 0,00314136 | 0,02798061 |
| NANOZOOG8519 | 7,02329187 | 3,18297566 | 1,78804445 | 0,00304374 | 0,02748319 |
| NANOZOOG9248 | 35,7151791 | 3,17652898 | 0,59241682 | 2,2104E-08 | 1,6686E-06 |
| NANOZOOG994 | 62,3213066 | -3,1671409 | 0,35447413 | 3,9388E-20 | 3,023E-17 |
| NANOZOOG1049 | 207,189312 | 3,15450489 | 1,26238447 | 0,00044043 | 0,00610903 |
| NANOZOOG7704 | 10,0474018 | 3,14822297 | 1,10690937 | 0,00100452 | 0,01147845 |
| NANOZOOG5838 | 4,99100354 | -3,1383262 | 0,96580067 | 0,00021062 | 0,00352691 |
| NANOZOOG3450 | 3,10821944 | 3,13059031 | 2,36886919 | 0,00408857 | 0,03384656 |
| NANOZOOG2923 | 19,9575495 | -3,1289363 | 0,52549517 | 3,4794E-10 | 4,2165E-08 |
| NANOZOOG4562 | 29,9400609 | 3,11393825 | 0,92122999 | 0,00015469 | 0,00281569 |
| NANOZOOG4086 | 6,84513299 | 3,11269376 | 1,07932415 | 0,00089793 | 0,01046827 |
| NANOZOOG50 | 8,96269717 | -3,1070078 | 0,63760021 | 1,6153E-07 | 8,4528E-06 |
| NANOZOOG9007 | 11,511231 | -3,101161 | 0,52685336 | 6,5821E-10 | 7,2168E-08 |
| NANOZOOG2297 | 25,1954911 | 3,09510995 | 1,10424879 | 0,00102696 | 0,01164821 |
| NANOZOOG6879 | 8,04267782 | 3,08101637 | 1,15370265 | 0,0008848 | 0,01036768 |
| NANOZOOG3699 | 7,83621211 | 3,07125244 | 1,01630765 | 0,00027936 | 0,00439069 |
| NANOZOOG4472 | 37,413628 | 3,06736791 | 0,55115159 | 2,7264E-09 | 2,5353E-07 |
| NANOZOOG4798 | 3,15167477 | 3,06145395 | 1,59397056 | 0,00330645 | 0,02900232 |
| NANOZOOG4462 | 12,5806857 | 3,05778504 | 1,25941952 | 0,00156644 | 0,01628007 |
| NANOZOOG9079 | 6,04701576 | -3,0539052 | 0,79822782 | 1,6508E-05 | 0,00044718 |
| NANOZOOG4135 | 7,28360182 | 2,99771411 | 1,00674492 | 0,00043259 | 0,00601837 |
| NANOZOOG4261 | 5,11781988 | -2,9944247 | 1,13230923 | 0,00153021 | 0,01601502 |
| NANOZOOG6866 | 3,38698148 | 2,99284141 | 2,08287523 | 0,0030842 | 0,0276857 |
| NANOZOOG173 | 6,8295275 | 2,98797271 | 1,02609145 | 0,00104768 | 0,01185403 |
| NANOZOOG4516 | 5,72216416 | -2,9849535 | 1,02931966 | 0,00108622 | 0,01217039 |
| NANOZOOG3457 | 3,8866416 | 2,98151974 | 2,38698288 | 0,00476269 | 0,03787945 |
| NANOZOOG9335 | 3,29075786 | -2,9742872 | 1,95658245 | 0,00504915 | 0,03947595 |
| NANOZOOG3586 | 10,4018158 | -2,9701796 | 0,64835067 | 7,7999E-07 | 3,3885E-05 |
| NANOZOOG2356 | 3,76821898 | 2,96908375 | 1,38393759 | 0,0028193 | 0,02575967 |
| NANOZOOG8694 | 11,5129247 | 2,96625799 | 1,24538 | 0,00182637 | 0,01800953 |
| NANOZOOG6302 | 4,88365455 | 2,96436363 | 1,28906226 | 0,00217447 | 0,02103667 |
| NANOZOOG3603 | 3,97264257 | 2,96173402 | 2,07616698 | 0,00318646 | 0,02827288 |
| NANOZOOG1757 | 13,5208293 | 2,95408716 | 0,70531876 | 5,3392E-06 | 0,00017688 |
| NANOZOOG5022 | 3,56226191 | -2,9524924 | 1,0243196 | 0,00058249 | 0,0076547 |
| NANOZOOG5127 | 16,7907299 | -2,9421459 | 0,53705902 | 6,387E-09 | 5,5495E-07 |
| NANOZOOG1355 | 23,6332725 | 2,93627588 | 0,74510327 | 1,3905E-05 | 0,000392 |
| NANOZOOG197 | 11,7250559 | 2,92577235 | 1,35641024 | 0,00207003 | 0,02019113 |
| NANOZOOG6878 | 21,1081842 | 2,91847529 | 1,91080418 | 0,00228829 | 0,02183604 |
| NANOZOOG4566 | 7,88599152 | 2,91097587 | 2,24568374 | 0,00434177 | 0,03538732 |
| NANOZOOG3437 | 6,82919287 | -2,8901996 | 0,96000594 | 0,00051849 | 0,0070019 |
| NANOZOOG9296 | 31,7672599 | 2,88882921 | 0,67657007 | 5,559E-06 | 0,00018156 |
| NANOZOOG4670 | 16,8289723 | 2,86764705 | 0,89382706 | 0,00029891 | 0,0046346 |
| NANOZOOG7480 | 5,33055482 | 2,86605202 | 1,36707966 | 0,00328499 | 0,02886906 |
| NANOZOOG1422 | 11,3312125 | 2,86484215 | 0,7410046 | 1,3072E-05 | 0,000378 |
| NANOZOOG468 | 10,2614176 | 2,86250836 | 1,01252335 | 0,00068099 | 0,00861522 |
| NANOZOOG1220 | 952,831026 | -2,8610905 | 0,43204547 | 2,4349E-12 | 4,875E-10 |
| NANOZOOG9299 | 3,32423773 | -2,8477073 | 1,19395041 | 0,00259326 | 0,02402807 |
| NANOZOOG9040 | 4,95491646 | -2,8475194 | 0,87772473 | 0,00013199 | 0,00248089 |
| NANOZOOG1872 | 10,8556682 | -2,8466326 | 0,77734455 | 3,6265E-05 | 0,00084771 |
| NANOZOOG7175 | 111,962567 | 2,82295518 | 1,59360994 | 0,00240348 | 0,02258784 |
| NANOZOOG1272 | 5,88442772 | 2,81907594 | 2,10685386 | 0,00399167 | 0,03339266 |
| NANOZOOG3509 | 35,5787406 | -2,8182444 | 0,33281117 | 2,8461E-18 | 1,4563E-15 |
| NANOZOOG4755 | 16,4187158 | -2,8051667 | 0,46764458 | 2,2978E-10 | 2,9393E-08 |
| NANOZOOG2000 | 25,825969 | -2,8009291 | 0,4249976 | 4,8425E-12 | 8,9199E-10 |
| NANOZOOG680 | 5,05794085 | 2,79607945 | 1,12820023 | 0,00141324 | 0,01499533 |
| NANOZOOG7182 | 35,8335813 | 2,79512757 | 1,45590986 | 0,0041239 | 0,03403326 |
| NANOZOOG2738 | 11,6453349 | 2,79466499 | 1,01169732 | 0,00049895 | 0,00681802 |
| NANOZOOG9252 | 25,4456035 | -2,7914476 | 0,39078758 | 1,2638E-13 | 3,4234E-11 |
| NANOZOOG2696 | 4,36959571 | 2,7849483 | 1,36427973 | 0,00370969 | 0,0317427 |
| NANOZOOG3929 | 157,426197 | -2,7745533 | 0,36712373 | 3,2256E-15 | 1,061E-12 |
| NANOZOOG2643 | 11,7726012 | 2,76428506 | 1,28942213 | 0,0029733 | 0,02700602 |
| NANOZOOG1300 | 3,91393691 | 2,74664452 | 1,54352718 | 0,00531396 | 0,04092104 |
| NANOZOOG9406 | 37,3888683 | -2,7461357 | 0,48082296 | 9,813E-10 | 1,027E-07 |
| NANOZOOG8846 | 3,5332491 | 2,73628358 | 1,49790095 | 0,00487872 | 0,03853605 |
| NANOZOOG3816 | 5,64253235 | -2,7362398 | 0,85701216 | 0,00024457 | 0,00395171 |
| NANOZOOG6103 | 2,9270702 | 2,73130887 | 1,5149748 | 0,00508175 | 0,03966348 |
| NANOZOOG7746 | 6,17723086 | -2,7189407 | 0,76276139 | 6,2612E-05 | 0,00131657 |
| NANOZOOG6535 | 3,93181041 | -2,7178628 | 0,900639 | 0,00036124 | 0,00532692 |
| NANOZOOG1935 | 29,7560599 | 2,71583293 | 0,74724051 | 2,9382E-05 | 0,00072355 |
| NANOZOOG7375 | 8,17314818 | 2,70415233 | 1,14092831 | 0,00178898 | 0,01790751 |
| NANOZOOG2031 | 5,64221924 | -2,6940986 | 0,93995276 | 0,00071615 | 0,00888916 |
| NANOZOOG3957 | 8,84741716 | 2,6919233 | 1,42766319 | 0,00428223 | 0,03508837 |
| NANOZOOG8737 | 20,1857249 | -2,6892784 | 0,6290593 | 2,8757E-06 | 0,0001051 |
| NANOZOOG1067 | 119,474499 | 2,6866633 | 0,50155313 | 7,3423E-09 | 6,1475E-07 |
| NANOZOOG7812 | 19,028802 | -2,6796168 | 0,57885882 | 4,3499E-07 | 2,0233E-05 |
| NANOZOOG1182 | 24,2154792 | -2,6676225 | 0,43865295 | 1,1154E-10 | 1,657E-08 |
| NANOZOOG3002 | 9,9863811 | 2,66683764 | 1,44382445 | 0,00537267 | 0,04096215 |
| NANOZOOG3057 | 16,0389307 | 2,66622712 | 2,30585906 | 0,00532646 | 0,04094885 |
| NANOZOOG5278 | 10,2701675 | -2,6653719 | 0,77848096 | 0,00014092 | 0,00261663 |
| NANOZOOG6202 | 14,3806575 | -2,6633191 | 0,5156806 | 2,9748E-08 | 2,0756E-06 |
| NANOZOOG3114 | 7,50495214 | 2,65205957 | 1,29698524 | 0,00386051 | 0,0328001 |
| NANOZOOG4986 | 11,6465966 | -2,647092 | 0,87603111 | 0,00062618 | 0,00807717 |
| NANOZOOG3033 | 15,0686661 | -2,6425403 | 0,53397678 | 9,3043E-08 | 5,2897E-06 |
| NANOZOOG2337 | 5,18036299 | 2,63974636 | 1,54708885 | 0,00566535 | 0,04270614 |
| NANOZOOG2512 | 8,73059628 | 2,63649402 | 1,05461819 | 0,00284156 | 0,0259117 |
| NANOZOOG4327 | 18,2657437 | -2,6339883 | 0,43034808 | 1,373E-10 | 1,9159E-08 |
| NANOZOOG6449 | 26,5563875 | 2,6291282 | 0,71741745 | 5,5189E-05 | 0,00120447 |
| NANOZOOG3444 | 16,0913381 | 2,6221992 | 0,90528353 | 0,00077282 | 0,00936531 |
| NANOZOOG648 | 11,0714378 | 2,61437726 | 1,28921074 | 0,00397151 | 0,03339266 |
| NANOZOOG2713 | 42,2113882 | -2,6071991 | 0,48107147 | 4,7611E-09 | 4,2163E-07 |
| NANOZOOG9174 | 6,88462928 | -2,5888855 | 0,80853391 | 0,00025871 | 0,00413671 |
| NANOZOOG3605 | 7,60163965 | -2,5822965 | 0,69824132 | 2,365E-05 | 0,0006153 |
| NANOZOOG9260 | 13,783931 | 2,5792353 | 0,7831479 | 0,0001337 | 0,00250284 |
| NANOZOOG6716 | 5,42060943 | 2,57916324 | 1,32470564 | 0,00462906 | 0,03707271 |
| NANOZOOG6217 | 5,96896804 | 2,57337335 | 1,55268461 | 0,0058099 | 0,04357428 |
| NANOZOOG7071 | 8,98002991 | -2,5552328 | 0,90944981 | 0,00068092 | 0,00861522 |
| NANOZOOG2987 | 9,57469465 | 2,54716068 | 1,54677929 | 0,00616287 | 0,04555377 |
| NANOZOOG6874 | 31,584261 | 2,53971641 | 1,26283113 | 0,00209506 | 0,02035388 |
| NANOZOOG3159 | 5,90295388 | 2,5362714 | 1,48565125 | 0,00578816 | 0,04348203 |
| NANOZOOG888 | 81,634109 | 2,52192295 | 0,79284995 | 0,00017055 | 0,00297489 |
| NANOZOOG9058 | 10,7662841 | 2,5209696 | 0,76482019 | 0,00013984 | 0,00260716 |
| NANOZOOG3030 | 6,22407086 | 2,51738547 | 2,06669086 | 0,0055351 | 0,0420613 |
| NANOZOOG667 | 45,2717667 | -2,5088981 | 0,34740741 | 5,4586E-14 | 1,571E-11 |
| NANOZOOG7973 | 49,5229523 | -2,5069482 | 0,34388819 | 3,0275E-14 | 9,2944E-12 |
| NANOZOOG6610 | 41,6105119 | 2,49456492 | 0,8017889 | 0,00016078 | 0,0028921 |
| NANOZOOG2157 | 8,25430055 | 2,48898832 | 1,29328485 | 0,00512629 | 0,03987597 |
| NANOZOOG3822 | 10,2914716 | -2,4886985 | 0,68783829 | 5,6843E-05 | 0,00121186 |
| NANOZOOG8335 | 6,01591881 | -2,4877521 | 0,96114979 | 0,00160074 | 0,01645403 |
| NANOZOOG7779 | 6,30473606 | 2,47705016 | 0,9722824 | 0,00174118 | 0,01758367 |
| NANOZOOG9033 | 10,7882718 | 2,47620893 | 0,83667881 | 0,00038817 | 0,00558607 |
| NANOZOOG2726 | 19,2943875 | -2,4746795 | 0,50539987 | 1,3025E-07 | 6,9746E-06 |
| NANOZOOG6510 | 9,23198052 | 2,46853672 | 0,75186649 | 0,00015722 | 0,00283924 |
| NANOZOOG8459 | 7,79578632 | -2,466989 | 0,74145602 | 0,00016675 | 0,00294215 |
| NANOZOOG6038 | 5,03878331 | -2,4630302 | 0,95768551 | 0,00170094 | 0,01725288 |
| NANOZOOG3241 | 33,0702194 | -2,4586054 | 0,43334441 | 1,5608E-09 | 1,5292E-07 |
| NANOZOOG2579 | 7,57870551 | -2,4533622 | 0,72978687 | 7,8323E-05 | 0,0015889 |
| NANOZOOG2460 | 4,93766239 | -2,4484699 | 1,33955093 | 0,00643801 | 0,04720868 |
| NANOZOOG2591 | 10,3288342 | -2,44429 | 0,87076711 | 0,0004833 | 0,00664356 |
| NANOZOOG7140 | 11,2141426 | 2,43800968 | 0,93069789 | 0,00156525 | 0,01628007 |
| NANOZOOG3329 | 64,4655368 | -2,4283297 | 0,27978854 | 4,1134E-19 | 2,3678E-16 |
| NANOZOOG2290 | 12,473745 | -2,4258517 | 0,6168347 | 8,9703E-06 | 0,00027723 |
| NANOZOOG4968 | 19,1028576 | 2,42103902 | 0,52872663 | 9,1178E-07 | 3,8897E-05 |
| NANOZOOG4060 | 12,7362619 | 2,40541483 | 0,60195104 | 9,7033E-06 | 0,00029437 |
| NANOZOOG3087 | 7,79312718 | -2,4013749 | 0,80232799 | 0,00058145 | 0,0076547 |
| NANOZOOG3062 | 20,4673878 | -2,4000102 | 0,47638594 | 6,8384E-08 | 4,1988E-06 |
| NANOZOOG2683 | 20,2728066 | 2,37001727 | 0,82661157 | 0,00042255 | 0,00591448 |
| NANOZOOG5958 | 11,7679965 | 2,36805778 | 0,72955216 | 0,00024054 | 0,00392018 |
| NANOZOOG8667 | 30,7308764 | -2,364772 | 0,47521608 | 8,0311E-08 | 4,803E-06 |
| NANOZOOG7705 | 16,4808555 | 2,3602482 | 0,75768202 | 0,00018373 | 0,00316888 |
| NANOZOOG1051 | 2,93502421 | -2,3564907 | 1,18483664 | 0,00661014 | 0,04816409 |
| NANOZOOG1722 | 18,8435701 | 2,35559244 | 0,95133055 | 0,0022903 | 0,02183604 |
| NANOZOOG8868 | 8,73504862 | -2,3532473 | 0,93134429 | 0,00075048 | 0,00911859 |
| NANOZOOG3330 | 14,5706288 | 2,35015807 | 0,86527072 | 0,00066857 | 0,00850486 |
| NANOZOOG1415 | 25,2324094 | -2,3499488 | 0,46566403 | 6,344E-08 | 4,0575E-06 |
| NANOZOOG2324 | 8,13830373 | -2,3478904 | 0,71679241 | 0,00013141 | 0,00248002 |
| NANOZOOG1494 | 10,2749456 | -2,3410735 | 0,65329083 | 4,8292E-05 | 0,00109011 |
| NANOZOOG5502 | 9,04167772 | 2,34102236 | 0,914202 | 0,00143187 | 0,01515811 |
| NANOZOOG2236 | 27,0982402 | -2,339726 | 0,47825206 | 9,2374E-08 | 5,2897E-06 |
| NANOZOOG4779 | 54,0184453 | 2,33648643 | 0,49915709 | 3,4623E-07 | 1,6961E-05 |
| NANOZOOG6790 | 27,2594694 | -2,3360002 | 0,394172 | 4,4901E-10 | 5,3018E-08 |
| NANOZOOG2191 | 13,5306591 | 2,33498348 | 0,58556434 | 9,5725E-06 | 0,00029387 |
| NANOZOOG8726 | 30,5702933 | -2,3341182 | 0,36673462 | 2,0836E-11 | 3,4267E-09 |
| NANOZOOG5177 | 8,31812342 | -2,3257641 | 0,81609785 | 0,00081033 | 0,00974295 |
| NANOZOOG639 | 8,71051667 | -2,3230894 | 0,60596803 | 1,629E-05 | 0,00044652 |
| NANOZOOG6809 | 26,9593259 | 2,32250556 | 0,87382823 | 0,00164945 | 0,01684192 |
| NANOZOOG6425 | 291,158372 | 2,32235493 | 1,02483045 | 0,00129736 | 0,01405732 |
| NANOZOOG5462 | 5,58938254 | 2,31526635 | 0,87007698 | 0,00094216 | 0,01090117 |
| NANOZOOG9099 | 10,1439239 | -2,3114067 | 0,7407536 | 0,00027529 | 0,00435033 |
| NANOZOOG505 | 7,79746033 | 2,30771771 | 1,10189815 | 0,00409393 | 0,03384656 |
| NANOZOOG4368 | 179,243697 | -2,2973748 | 0,50095807 | 3,9186E-07 | 1,8603E-05 |
| NANOZOOG3195 | 33,0595944 | 2,28322723 | 0,59173807 | 1,1193E-05 | 0,00032829 |
| NANOZOOG6667 | 6,97930741 | -2,2659739 | 0,90201215 | 0,00165709 | 0,01688256 |
| NANOZOOG4230 | 4,49638855 | 2,26540833 | 1,09964692 | 0,00513567 | 0,03988157 |
| NANOZOOG5110 | 42,2354385 | -2,2610415 | 0,44749867 | 4,392E-08 | 3,0187E-06 |
| NANOZOOG3634 | 9,17524668 | -2,260011 | 0,60257427 | 3,1222E-05 | 0,00075276 |
| NANOZOOG3744 | 99,9214103 | -2,2484101 | 0,22641077 | 2,9081E-24 | 4,4639E-21 |
| NANOZOOG7736 | 7,7140137 | -2,2451176 | 0,68863045 | 0,00019403 | 0,00330929 |
| NANOZOOG2795 | 35,6566274 | -2,2394981 | 0,49113085 | 5,1119E-07 | 2,2854E-05 |
| NANOZOOG6225 | 12,2587265 | -2,2347984 | 0,69068875 | 0,00018586 | 0,00319362 |
| NANOZOOG8101 | 32,699018 | -2,2328377 | 0,32944084 | 2,1507E-12 | 4,7163E-10 |
| NANOZOOG5824 | 11,2873541 | -2,2322319 | 0,65239044 | 9,0039E-05 | 0,00180274 |
| NANOZOOG7299 | 96,8537927 | -2,2235349 | 0,31559045 | 2,0349E-13 | 4,932E-11 |
| NANOZOOG2619 | 26,6243649 | 2,21143251 | 1,06067401 | 0,00448894 | 0,03639361 |
| NANOZOOG8218 | 7,91078152 | -2,209057 | 0,684148 | 0,00018666 | 0,00319547 |
| NANOZOOG5027 | 6,40972693 | -2,2066314 | 0,90422352 | 0,00203189 | 0,01986594 |
| NANOZOOG797 | 18,5021964 | 2,20015996 | 0,75463083 | 0,00061666 | 0,00800816 |
| NANOZOOG4066 | 46,0855194 | 2,19970063 | 0,3745289 | 6,519E-10 | 7,2168E-08 |
| NANOZOOG6243 | 8,02569664 | -2,1940738 | 0,89420929 | 0,00178489 | 0,01790719 |
| NANOZOOG3468 | 15,0129618 | 2,18127753 | 0,58370474 | 2,9898E-05 | 0,00073234 |
| NANOZOOG694 | 29,3016347 | -2,1808772 | 0,38181587 | 1,3988E-09 | 1,4003E-07 |
| NANOZOOG9188 | 3,49792852 | 2,17358904 | 1,04026587 | 0,00424123 | 0,03481435 |
| NANOZOOG721 | 163,131818 | -2,1703256 | 0,49661053 | 1,1992E-06 | 4,9308E-05 |
| NANOZOOG7117 | 17,4982969 | -2,163269 | 0,45516083 | 3,5371E-07 | 1,7138E-05 |
| NANOZOOG2972 | 18,2977989 | -2,1628382 | 0,48348823 | 9,1223E-07 | 3,8897E-05 |
| NANOZOOG5522 | 29,8190569 | -2,1590809 | 0,35236877 | 1,2896E-10 | 1,8558E-08 |
| NANOZOOG4031 | 39,2449135 | -2,1515611 | 0,56278297 | 1,3134E-05 | 0,000378 |
| NANOZOOG6199 | 18,0383189 | -2,1409721 | 0,77780438 | 0,00082922 | 0,00986704 |
| NANOZOOG2998 | 3,99042636 | -2,1404002 | 0,99474567 | 0,00500086 | 0,03917064 |
| NANOZOOG1320 | 16,3360673 | -2,1381644 | 0,40456142 | 1,8839E-08 | 1,4459E-06 |
| NANOZOOG3970 | 11,6356274 | 2,12291999 | 0,81253098 | 0,00101021 | 0,01151491 |
| NANOZOOG1024 | 9,83477282 | 2,11812497 | 0,80859066 | 0,00093646 | 0,01086248 |
| NANOZOOG3078 | 6,76594295 | -2,1073342 | 0,94645864 | 0,00348099 | 0,03024525 |
| NANOZOOG1185 | 14,3867878 | -2,1058267 | 0,50968856 | 4,3343E-06 | 0,00015121 |
| NANOZOOG1053 | 12,5007213 | 2,09781245 | 0,86493265 | 0,00230522 | 0,02188769 |
| NANOZOOG2514 | 94,1041735 | -2,0963311 | 0,37598583 | 2,7528E-09 | 2,5353E-07 |
| NANOZOOG4354 | 13,1724708 | -2,09269 | 0,55069823 | 2,6193E-05 | 0,00067384 |
| NANOZOOG1080 | 42,8041903 | -2,0874426 | 0,29398586 | 1,4213E-13 | 3,6362E-11 |
| NANOZOOG7462 | 13,0534301 | -2,0811506 | 0,49262333 | 4,249E-06 | 0,00014936 |
| NANOZOOG389 | 1754,182 | -2,0802666 | 0,19362383 | 8,4889E-28 | 3,9091E-24 |
| NANOZOOG4862 | 11,2732095 | -2,0792433 | 0,72435231 | 0,00069761 | 0,00870596 |
| NANOZOOG6214 | 4,35690975 | -2,0790916 | 1,03626918 | 0,00631643 | 0,04653946 |
| NANOZOOG9353 | 416,265554 | -2,0777208 | 0,3361274 | 7,0839E-11 | 1,0874E-08 |
| NANOZOOG4291 | 7,9476483 | -2,0695886 | 0,76518632 | 0,00079452 | 0,00957789 |
| NANOZOOG3643 | 8,35097494 | -2,0693763 | 0,68902573 | 0,00031015 | 0,0047608 |
| NANOZOOG4377 | 27,8739357 | -2,0673918 | 0,41783831 | 9,7267E-08 | 5,4624E-06 |
| NANOZOOG8663 | 12,4604318 | 2,06694844 | 0,76983252 | 0,00079451 | 0,00957789 |
| NANOZOOG6135 | 13,7776098 | -2,0661258 | 0,52906995 | 1,367E-05 | 0,00038858 |
| NANOZOOG39 | 13,4721378 | -2,0606671 | 0,52114155 | 1,0036E-05 | 0,00030207 |
| NANOZOOG1206 | 13,118621 | 2,05343805 | 1,0144187 | 0,00398648 | 0,03339266 |
| NANOZOOG3584 | 6,5523407 | -2,0459472 | 0,93721038 | 0,00510402 | 0,03976988 |
| NANOZOOG3173 | 68,1794329 | 2,03786601 | 0,42422073 | 2,49E-07 | 1,2329E-05 |
| NANOZOOG2305 | 27,6098171 | 2,03657236 | 0,81859201 | 0,00134784 | 0,01452741 |
| NANOZOOG6948 | 13,0370624 | 2,03533928 | 0,54045943 | 2,8496E-05 | 0,00072101 |
| NANOZOOG6467 | 18,9985646 | -2,0288345 | 0,4979216 | 5,8258E-06 | 0,00018761 |
| NANOZOOG3460 | 77,9679154 | -2,0287507 | 0,37678068 | 8,9625E-09 | 7,2407E-07 |
| NANOZOOG1 | 23,0651078 | -2,0284711 | 0,40390949 | 6,1628E-08 | 3,9971E-06 |
| NANOZOOG5058 | 154,16404 | -2,0254362 | 0,39103375 | 2,5851E-08 | 1,92E-06 |
| NANOZOOG6327 | 27,5378976 | -2,0179375 | 0,37058744 | 6,9644E-09 | 5,9391E-07 |
| NANOZOOG2790 | 12,0523228 | 2,01662637 | 0,8570983 | 0,00313869 | 0,02798061 |
| NANOZOOG4064 | 26,1749132 | 2,01095356 | 0,62790916 | 0,00017545 | 0,00304893 |
| NANOZOOG3054 | 9,54574205 | 2,00841765 | 0,74006061 | 0,00097719 | 0,0112218 |
| NANOZOOG124 | 3,34000107 | -2,004682 | 0,89471271 | 0,00358912 | 0,03085099 |
| NANOZOOG6224 | 28,3655625 | -1,9992453 | 0,45638705 | 1,4045E-06 | 5,6733E-05 |
| NANOZOOG4781 | 12,2953674 | 1,99817094 | 0,75382081 | 0,00137405 | 0,0147151 |
| NANOZOOG1965 | 17,301247 | -1,9940986 | 0,63797881 | 0,00019577 | 0,00332663 |
| NANOZOOG2397 | 11,6690891 | 1,9874018 | 0,89762515 | 0,00359873 | 0,0308606 |
| NANOZOOG801 | 15,1880003 | -1,9681662 | 0,45462069 | 2,3662E-06 | 8,8588E-05 |
| NANOZOOG8721 | 81,8155264 | -1,9643586 | 0,67781905 | 0,00033556 | 0,00508313 |
| NANOZOOG5236 | 308,655894 | -1,9629649 | 0,30160776 | 9,293E-12 | 1,585E-09 |
| NANOZOOG6300 | 10,0645397 | -1,9470421 | 0,5635272 | 7,4068E-05 | 0,00153027 |
| NANOZOOG3284 | 36,9930721 | 1,94176933 | 0,53646458 | 5,2249E-05 | 0,00114574 |
| NANOZOOG1271 | 13,9944934 | -1,9386907 | 0,45365054 | 2,9938E-06 | 0,00010856 |
| NANOZOOG8934 | 24,8239014 | -1,9274533 | 0,40943776 | 3,5728E-07 | 1,7138E-05 |
| NANOZOOG7018 | 8,83682688 | -1,9271951 | 0,69985286 | 0,00069611 | 0,00870596 |
| NANOZOOG2150 | 206,475206 | -1,9247741 | 0,39275484 | 1,2237E-07 | 6,6296E-06 |
| NANOZOOG7185 | 8,81165965 | -1,9228667 | 0,56811425 | 9,493E-05 | 0,00188429 |
| NANOZOOG4134 | 39,6497043 | 1,92086505 | 0,52025678 | 2,8684E-05 | 0,00072181 |
| NANOZOOG3548 | 121,478873 | -1,9040089 | 0,37442392 | 4,5645E-08 | 3,0463E-06 |
| NANOZOOG227 | 9,23489798 | 1,89664612 | 0,80627906 | 0,00265973 | 0,02444725 |
| NANOZOOG8686 | 9,16346926 | 1,89463742 | 0,80619213 | 0,003119 | 0,02789795 |
| NANOZOOG4848 | 41,0205573 | -1,8902104 | 0,41199713 | 5,7381E-07 | 2,5408E-05 |
| NANOZOOG8296 | 12,4569947 | -1,8900219 | 0,66702003 | 0,00073584 | 0,00903611 |
| NANOZOOG1014 | 15,6823519 | -1,8876978 | 0,63856106 | 0,0004011 | 0,00570086 |
| NANOZOOG2314 | 16,9664846 | -1,881007 | 0,59200394 | 0,00023996 | 0,00392018 |
| NANOZOOG9172 | 3,69119507 | -1,8755841 | 0,88821323 | 0,00536086 | 0,04096215 |
| NANOZOOG9082 | 12,2801621 | 1,85715973 | 0,83500084 | 0,00459191 | 0,03690353 |
| NANOZOOG5656 | 6,81342101 | -1,849946 | 0,69120714 | 0,00116553 | 0,01280965 |
| NANOZOOG8333 | 32,1066768 | -1,8461532 | 0,36692073 | 6,5569E-08 | 4,0804E-06 |
| NANOZOOG983 | 6,99107955 | -1,8442388 | 0,78127914 | 0,00319421 | 0,02828722 |
| NANOZOOG5423 | 6,26984574 | -1,8369297 | 0,77119531 | 0,00181227 | 0,0179473 |
| NANOZOOG3851 | 5,45088097 | -1,8364112 | 0,89000848 | 0,00615481 | 0,04555377 |
| NANOZOOG8767 | 25,3503334 | 1,83613531 | 0,56509998 | 0,00020094 | 0,00337717 |
| NANOZOOG3215 | 14,6573566 | -1,8300004 | 0,67907497 | 0,00086914 | 0,01028896 |
| NANOZOOG4680 | 23,2243998 | 1,8297549 | 0,67695433 | 0,00136501 | 0,01465235 |
| NANOZOOG2954 | 8,60585892 | 1,82878953 | 0,62170911 | 0,00056299 | 0,0075147 |
| NANOZOOG2860 | 14,3470338 | -1,8286393 | 0,55660412 | 0,00016409 | 0,00292202 |
| NANOZOOG7619 | 10,7206614 | -1,8264104 | 0,61622388 | 0,00056653 | 0,0075401 |
| NANOZOOG740 | 32,2520998 | -1,8252661 | 0,44364787 | 5,0019E-06 | 0,00017024 |
| NANOZOOG7443 | 20,8187836 | -1,8250913 | 0,43751395 | 4,5394E-06 | 0,00015717 |
| NANOZOOG9085 | 41,6038065 | -1,8242372 | 0,37388137 | 1,5102E-07 | 7,9936E-06 |
| NANOZOOG1570 | 27,6216752 | -1,8228572 | 0,3338892 | 8,5912E-09 | 7,0647E-07 |
| NANOZOOG407 | 11,9520836 | -1,8161332 | 0,48232037 | 3,0081E-05 | 0,00073294 |
| NANOZOOG3592 | 28,0029299 | -1,8091016 | 0,3718824 | 2,0712E-07 | 1,0597E-05 |
| NANOZOOG4949 | 49,6504217 | -1,8066711 | 0,28301411 | 2,5596E-11 | 4,0645E-09 |
| NANOZOOG3392 | 17,866845 | -1,8026806 | 0,45126328 | 9,7164E-06 | 0,00029437 |
| NANOZOOG8369 | 40,8808978 | 1,79910452 | 0,58030193 | 0,00026994 | 0,00428654 |
| NANOZOOG309 | 9,42737227 | -1,792448 | 0,57544189 | 0,00028774 | 0,00449611 |
| NANOZOOG3287 | 21,246448 | 1,78998179 | 0,63636937 | 0,00081607 | 0,00978641 |
| NANOZOOG4251 | 17,7173747 | -1,7880897 | 0,53771111 | 0,00017034 | 0,00297489 |
| NANOZOOG2269 | 5,2723466 | -1,7859412 | 0,77047542 | 0,00299643 | 0,02710912 |
| NANOZOOG3861 | 42,2750459 | -1,7838517 | 0,3431774 | 2,8703E-08 | 2,0335E-06 |
| NANOZOOG6118 | 9,2851198 | -1,782069 | 0,74466543 | 0,00226962 | 0,02172886 |
| NANOZOOG4155 | 6,04275426 | -1,7719383 | 0,72739309 | 0,00229939 | 0,02187743 |
| NANOZOOG4281 | 15,3802095 | 1,77174825 | 0,80410352 | 0,00515439 | 0,03995956 |
| NANOZOOG3574 | 5,05974515 | -1,7703135 | 0,79563398 | 0,00306531 | 0,02762375 |
| NANOZOOG1046 | 16,7289934 | -1,7667619 | 0,63910582 | 0,00069406 | 0,00870596 |
| NANOZOOG8470 | 7,52522337 | -1,7622338 | 0,68096069 | 0,00164143 | 0,01679732 |
| NANOZOOG3512 | 13,5045079 | 1,75782998 | 0,75598551 | 0,00355308 | 0,03064029 |
| NANOZOOG6728 | 10,8129728 | -1,7496895 | 0,60634211 | 0,00064444 | 0,00828947 |
| NANOZOOG6635 | 26,1844126 | 1,74836497 | 0,49479103 | 7,4104E-05 | 0,00153027 |
| NANOZOOG3843 | 24,304072 | -1,7481627 | 0,39496755 | 1,5942E-06 | 6,3286E-05 |
| NANOZOOG8822 | 13,9026056 | -1,7454494 | 0,45665029 | 2,3295E-05 | 0,00061139 |
| NANOZOOG5271 | 15,7964407 | -1,7452574 | 0,59785241 | 0,00064784 | 0,00831002 |
| NANOZOOG2872 | 14,724585 | -1,7439793 | 0,52684721 | 0,00016434 | 0,00292202 |
| NANOZOOG867 | 8,68952028 | -1,7435513 | 0,68189114 | 0,00157084 | 0,01628007 |
| NANOZOOG7217 | 6717,66455 | -1,7434998 | 0,22804314 | 3,1142E-15 | 1,061E-12 |
| NANOZOOG4787 | 342,78408 | -1,7365998 | 0,35213279 | 1,143E-07 | 6,266E-06 |
| NANOZOOG3149 | 22,3078066 | -1,731242 | 0,56699903 | 0,00040068 | 0,00570086 |
| NANOZOOG6045 | 68,8618212 | -1,725385 | 0,36069854 | 2,3384E-07 | 1,1705E-05 |
| NANOZOOG6990 | 50,2147725 | -1,7228599 | 0,35595659 | 1,8705E-07 | 9,6783E-06 |
| NANOZOOG5999 | 21,6560344 | -1,7226265 | 0,39647777 | 2,3172E-06 | 8,8189E-05 |
| NANOZOOG3339 | 7,79305911 | 1,72039996 | 0,79465916 | 0,00536191 | 0,04096215 |
| NANOZOOG6308 | 9,54202025 | -1,7199523 | 0,56567995 | 0,00037835 | 0,00547888 |
| NANOZOOG7172 | 5,01193631 | -1,7158841 | 0,66045043 | 0,00150284 | 0,01576446 |
| NANOZOOG1991 | 20,5466405 | -1,715549 | 0,55232549 | 0,00024248 | 0,00393178 |
| NANOZOOG1273 | 9,92322028 | -1,7108334 | 0,57540798 | 0,00039186 | 0,00562161 |
| NANOZOOG8709 | 8,46521919 | -1,7092515 | 0,71692095 | 0,0025122 | 0,02341834 |
| NANOZOOG2556 | 35,2035694 | -1,7055742 | 0,39058656 | 2,1495E-06 | 8,3182E-05 |
| NANOZOOG2229 | 72,6663268 | 1,70328734 | 0,3345955 | 6,4486E-08 | 4,0679E-06 |
| NANOZOOG8643 | 15,7212462 | -1,7005655 | 0,53546924 | 0,00024091 | 0,00392018 |
| NANOZOOG1673 | 22,6032221 | -1,7001537 | 0,405402 | 5,1976E-06 | 0,00017471 |
| NANOZOOG1780 | 9,76302063 | 1,69878761 | 0,70179365 | 0,00262425 | 0,02422442 |
| NANOZOOG3895 | 17,4709292 | -1,6934261 | 0,46068631 | 4,2545E-05 | 0,0009796 |
| NANOZOOG1149 | 420,336594 | -1,6918615 | 0,38439094 | 1,5002E-06 | 6,0074E-05 |
| NANOZOOG8982 | 14,3149071 | 1,68774079 | 0,84346651 | 0,00664737 | 0,04835885 |
| NANOZOOG653 | 129,104847 | 1,68713371 | 0,77535098 | 0,00308127 | 0,0276857 |
| NANOZOOG6400 | 25,1299151 | -1,6859027 | 0,3926707 | 3,0567E-06 | 0,00010997 |
| NANOZOOG542 | 12,8748462 | -1,685574 | 0,46564626 | 5,6133E-05 | 0,00120762 |
| NANOZOOG972 | 8,80954723 | -1,6811416 | 0,62082502 | 0,00120909 | 0,01322244 |
| NANOZOOG9235 | 7,86131647 | 1,68098007 | 0,82010318 | 0,00577687 | 0,0434681 |
| NANOZOOG6661 | 5,61793969 | -1,6804046 | 0,7737928 | 0,00480724 | 0,03810216 |
| NANOZOOG41 | 15,1906121 | -1,6799821 | 0,49115146 | 9,277E-05 | 0,00184937 |
| NANOZOOG1340 | 10,8461963 | -1,6799619 | 0,60736874 | 0,0008728 | 0,01029153 |
| NANOZOOG3802 | 16,9069139 | -1,6794626 | 0,4116979 | 7,1559E-06 | 0,00022571 |
| NANOZOOG3685 | 10,1791164 | -1,6774001 | 0,72134381 | 0,0026363 | 0,02428034 |
| NANOZOOG6626 | 18,7439234 | -1,6763709 | 0,63057438 | 0,00144495 | 0,01526147 |
| NANOZOOG7977 | 16,0350328 | 1,67124283 | 0,73600065 | 0,00359091 | 0,03085099 |
| NANOZOOG7811 | 19,0225576 | -1,6697703 | 0,54771179 | 0,00036449 | 0,00532853 |
| NANOZOOG1990 | 23,7330418 | -1,6680156 | 0,41702362 | 1,0397E-05 | 0,00030966 |
| NANOZOOG3227 | 20,9403826 | -1,6667198 | 0,50431843 | 0,00014463 | 0,00267474 |
| NANOZOOG4856 | 10,1735512 | -1,6660648 | 0,53778451 | 0,00035044 | 0,00525647 |
| NANOZOOG4873 | 17,5527432 | 1,66591631 | 0,65196927 | 0,00172169 | 0,01742497 |
| NANOZOOG6597 | 9,82082784 | -1,6641341 | 0,67029329 | 0,00184483 | 0,01815261 |
| NANOZOOG6354 | 697,014764 | -1,6622411 | 0,28344421 | 7,0889E-10 | 7,5918E-08 |
| NANOZOOG4304 | 33,1623604 | 1,65866679 | 0,77377083 | 0,00450802 | 0,03645159 |
| NANOZOOG7381 | 10,9054695 | -1,6578903 | 0,51603243 | 0,00023304 | 0,00384645 |
| NANOZOOG8772 | 43,433399 | -1,6488902 | 0,31522152 | 2,7529E-08 | 1,9808E-06 |
| NANOZOOG312 | 10,289908 | 1,64560232 | 0,63770305 | 0,00180648 | 0,01792852 |
| NANOZOOG3647 | 21,9213102 | 1,64488301 | 0,54045742 | 0,00046072 | 0,00637118 |
| NANOZOOG6309 | 10,6120411 | -1,6436813 | 0,61066315 | 0,00107345 | 0,01207122 |
| NANOZOOG5845 | 30,8392388 | -1,64312 | 0,34788364 | 4,0993E-07 | 1,9263E-05 |
| NANOZOOG6600 | 17,6465462 | -1,6431033 | 0,44563267 | 3,975E-05 | 0,00091985 |
| NANOZOOG7010 | 11,0337397 | -1,6396373 | 0,6680899 | 0,00249992 | 0,02335114 |
| NANOZOOG4003 | 53,484035 | 1,63538906 | 0,43470413 | 3,2614E-05 | 0,00077818 |
| NANOZOOG299 | 21,1897823 | -1,6352826 | 0,39372418 | 5,5994E-06 | 0,00018159 |
| NANOZOOG6844 | 13,6118857 | -1,6340281 | 0,45724701 | 6,3258E-05 | 0,0013241 |
| NANOZOOG1200 | 12,5242793 | 1,62254271 | 0,7174249 | 0,00400536 | 0,03339266 |
| NANOZOOG1188 | 12,5243284 | -1,6183658 | 0,58465277 | 0,00097669 | 0,0112218 |
| NANOZOOG3727 | 14,268174 | -1,615417 | 0,47439333 | 0,00011697 | 0,00225234 |
| NANOZOOG5888 | 14,1724147 | 1,6152401 | 0,58277929 | 0,00107475 | 0,01207122 |
| NANOZOOG6853 | 30,3327136 | 1,61377986 | 0,69775205 | 0,00271293 | 0,0248865 |
| NANOZOOG7898 | 29,1494947 | 1,60947874 | 0,5099308 | 0,00030425 | 0,00468593 |
| NANOZOOG231 | 61,3815248 | -1,6012821 | 0,29733361 | 1,2229E-08 | 9,5452E-07 |
| NANOZOOG6772 | 42,0164785 | -1,6005397 | 0,46070263 | 7,7011E-05 | 0,00156919 |
| NANOZOOG3211 | 21,8019284 | -1,5959412 | 0,41936487 | 2,6543E-05 | 0,00067905 |
| NANOZOOG6481 | 9,99080222 | -1,5958697 | 0,70501072 | 0,00466339 | 0,03728285 |
| NANOZOOG6298 | 30,0925843 | -1,5937933 | 0,34023307 | 4,7937E-07 | 2,1642E-05 |
| NANOZOOG2569 | 27,9826588 | 1,58906373 | 0,6595264 | 0,00234099 | 0,0221816 |
| NANOZOOG9001 | 14,8622587 | -1,5886916 | 0,53761357 | 0,00058345 | 0,0076547 |
| NANOZOOG5947 | 24,2760321 | -1,5837592 | 0,40628732 | 1,6426E-05 | 0,00044718 |
| NANOZOOG5195 | 8,90188757 | -1,5789234 | 0,76816506 | 0,00546614 | 0,0416059 |
| NANOZOOG4969 | 23,5299895 | 1,57390576 | 0,53342599 | 0,00061909 | 0,00800816 |
| NANOZOOG2533 | 15,5562625 | -1,5710748 | 0,51307835 | 0,00035157 | 0,00525647 |
| NANOZOOG3579 | 10,9738758 | 1,5618885 | 0,71861989 | 0,00555077 | 0,0421109 |
| NANOZOOG4103 | 19,6187156 | -1,5612787 | 0,39269457 | 1,3961E-05 | 0,000392 |
| NANOZOOG2495 | 17,8624124 | -1,5569889 | 0,47925207 | 0,00023084 | 0,00382378 |
| NANOZOOG4977 | 9,47251228 | -1,5482056 | 0,67825849 | 0,00380766 | 0,03247089 |
| NANOZOOG2383 | 15,2610811 | -1,5469461 | 0,4337157 | 6,6853E-05 | 0,00139302 |
| NANOZOOG2503 | 29,3830145 | 1,54688553 | 0,54654712 | 0,00089739 | 0,01046827 |
| NANOZOOG1208 | 25,2566822 | 1,54647941 | 0,6975855 | 0,00413962 | 0,0341019 |
| NANOZOOG3545 | 13,6848401 | -1,5403025 | 0,62705779 | 0,00256824 | 0,02384426 |
| NANOZOOG2045 | 10,1578296 | -1,5391472 | 0,53341778 | 0,00072993 | 0,00899679 |
| NANOZOOG99 | 20,5461316 | -1,5370133 | 0,49330414 | 0,00035924 | 0,00532692 |
| NANOZOOG2032 | 19,9240556 | 1,53564186 | 0,68548999 | 0,0047905 | 0,0380349 |
| NANOZOOG8418 | 143,574957 | -1,5353715 | 0,4207236 | 4,3583E-05 | 0,0009985 |
| NANOZOOG5683 | 28,6303938 | -1,534521 | 0,45066724 | 0,00010494 | 0,00203899 |
| NANOZOOG8693 | 15,4699491 | -1,5336934 | 0,53880719 | 0,00096108 | 0,01109212 |
| NANOZOOG9017 | 32,2806043 | -1,5334878 | 0,33452323 | 9,2766E-07 | 3,9192E-05 |
| NANOZOOG1941 | 10,6647384 | 1,5310479 | 0,73350929 | 0,00562594 | 0,04254098 |
| NANOZOOG8653 | 18,4342099 | -1,5261812 | 0,4809007 | 0,00031709 | 0,00485116 |
| NANOZOOG4254 | 11,5596912 | -1,5183794 | 0,71465198 | 0,00657625 | 0,04799305 |
| NANOZOOG3869 | 7,9985734 | -1,5126129 | 0,66875165 | 0,00352372 | 0,03044416 |
| NANOZOOG6101 | 22,5799359 | 1,50318732 | 0,60368755 | 0,00225355 | 0,02162 |
| NANOZOOG4306 | 22,4435291 | -1,502845 | 0,39673917 | 2,8435E-05 | 0,00072101 |
| NANOZOOG5238 | 65,4679197 | -1,4977124 | 0,34355995 | 2,3625E-06 | 8,8588E-05 |
| NANOZOOG6128 | 17,4834849 | -1,4966213 | 0,42828293 | 9,7247E-05 | 0,00191377 |
| NANOZOOG1895 | 154,815101 | -1,4914725 | 0,41498294 | 5,6196E-05 | 0,00120762 |
| NANOZOOG8603 | 22,4299428 | -1,4838551 | 0,44671423 | 0,00016789 | 0,00295082 |
| NANOZOOG3501 | 17,5170384 | -1,4792122 | 0,5126674 | 0,00074871 | 0,00911859 |
| NANOZOOG1201 | 9,74133808 | -1,4736923 | 0,6695072 | 0,0053614 | 0,04096215 |
| NANOZOOG4674 | 33,4247481 | 1,47334916 | 0,46848105 | 0,00033973 | 0,00512936 |
| NANOZOOG7205 | 30,0567405 | 1,46867764 | 0,53551143 | 0,00113636 | 0,01257916 |
| NANOZOOG2528 | 12,7017581 | 1,46713529 | 0,62446656 | 0,00371538 | 0,0317427 |
| NANOZOOG6550 | 181,802627 | -1,4662541 | 0,50950508 | 0,00066658 | 0,00850303 |
| NANOZOOG8188 | 556,979637 | -1,4658547 | 0,33446087 | 2,2093E-06 | 8,4781E-05 |
| NANOZOOG8636 | 19,5225892 | -1,4645075 | 0,443403 | 0,00018172 | 0,00314591 |
| NANOZOOG4167 | 45,1953936 | -1,4638488 | 0,34522453 | 5,255E-06 | 0,00017536 |
| NANOZOOG4285 | 20,2392175 | -1,4623449 | 0,72013672 | 0,00676238 | 0,04911791 |
| NANOZOOG5491 | 10,9476591 | 1,45770385 | 0,60934439 | 0,00311997 | 0,02789795 |
| NANOZOOG4657 | 15,1546475 | 1,45559724 | 0,67867848 | 0,00629529 | 0,046458 |
| NANOZOOG1744 | 18,2764723 | 1,45118445 | 0,68277886 | 0,00520397 | 0,04027613 |
| NANOZOOG1224 | 37,7245723 | -1,4508575 | 0,35158053 | 7,3922E-06 | 0,00023157 |
| NANOZOOG5006 | 20,3511365 | 1,45050877 | 0,62905762 | 0,00451192 | 0,03645159 |
| NANOZOOG9202 | 95,8964907 | 1,44616145 | 0,39946276 | 5,5781E-05 | 0,00120762 |
| NANOZOOG5378 | 20,9608867 | -1,444614 | 0,43689038 | 0,00019673 | 0,0033307 |
| NANOZOOG4262 | 18,9656078 | -1,4430466 | 0,48689085 | 0,00069277 | 0,00870596 |
| NANOZOOG3555 | 12,5666483 | -1,4419807 | 0,52012928 | 0,00114513 | 0,01264583 |
| NANOZOOG1192 | 19,0849628 | 1,4376585 | 0,65281031 | 0,00527899 | 0,04078816 |
| NANOZOOG8535 | 91,850603 | -1,4354182 | 0,31456694 | 1,0335E-06 | 4,2876E-05 |
| NANOZOOG191 | 16,531987 | -1,4352722 | 0,56261502 | 0,00192438 | 0,01889505 |
| NANOZOOG3126 | 25,0406053 | -1,434255 | 0,46509873 | 0,00041723 | 0,00587561 |
| NANOZOOG7240 | 127,611873 | -1,432217 | 0,42758793 | 0,00014652 | 0,00269895 |
| NANOZOOG2632 | 5,69773691 | -1,4304444 | 0,63574779 | 0,0049424 | 0,03889564 |
| NANOZOOG9148 | 34,4266517 | -1,4298014 | 0,39934134 | 7,5875E-05 | 0,0015529 |
| NANOZOOG2523 | 7,41295261 | -1,4246405 | 0,62966052 | 0,00500159 | 0,03917064 |
| NANOZOOG2162 | 10,9211327 | -1,4232267 | 0,51260764 | 0,00114806 | 0,01264792 |
| NANOZOOG2650 | 21,2434079 | 1,42111981 | 0,53707278 | 0,00182396 | 0,01800953 |
| NANOZOOG2247 | 3282,50365 | -1,4197366 | 0,35882655 | 1,4413E-05 | 0,00039984 |
| NANOZOOG4180 | 30,5618396 | -1,414352 | 0,36948103 | 2,9087E-05 | 0,00072332 |
| NANOZOOG678 | 24,5419671 | -1,4081858 | 0,41706386 | 0,00014866 | 0,00271662 |
| NANOZOOG2042 | 10,1887903 | -1,4058168 | 0,59507919 | 0,00399774 | 0,03339266 |
| NANOZOOG8398 | 13,8016654 | -1,4057879 | 0,54049083 | 0,00179658 | 0,01790751 |
| NANOZOOG8172 | 617,912454 | -1,4056475 | 0,44371866 | 0,00030131 | 0,00465611 |
| NANOZOOG1932 | 17,9773575 | -1,4049096 | 0,4530789 | 0,00036323 | 0,00532692 |
| NANOZOOG497 | 82,8808621 | -1,4032821 | 0,3834401 | 4,9635E-05 | 0,0011052 |
| NANOZOOG4780 | 40,926465 | 1,40199229 | 0,4408571 | 0,00032018 | 0,00488218 |
| NANOZOOG7015 | 12,0850088 | -1,4003226 | 0,61041306 | 0,00392561 | 0,03316963 |
| NANOZOOG5711 | 14,260105 | -1,3954372 | 0,44428529 | 0,00040428 | 0,00572833 |
| NANOZOOG766 | 40,6115685 | 1,39288116 | 0,26961286 | 5,6016E-08 | 3,685E-06 |
| NANOZOOG2335 | 19,2077367 | 1,39103255 | 0,54610534 | 0,00238867 | 0,02249451 |
| NANOZOOG7218 | 1279,94332 | -1,3909119 | 0,19517627 | 2,2559E-13 | 5,1943E-11 |
| NANOZOOG8966 | 11,6288459 | -1,3905781 | 0,58507264 | 0,00351825 | 0,03044416 |
| NANOZOOG5527 | 99,2323211 | -1,384473 | 0,31655353 | 2,5986E-06 | 9,6506E-05 |
| NANOZOOG7978 | 41,921181 | -1,3799084 | 0,33030939 | 6,5084E-06 | 0,0002067 |
| NANOZOOG9380 | 14,1436182 | 1,37260595 | 0,58268885 | 0,00401609 | 0,03339266 |
| NANOZOOG5476 | 287,222275 | -1,371488 | 0,27166623 | 9,1232E-08 | 5,2897E-06 |
| NANOZOOG9169 | 28,5313041 | -1,3705547 | 0,42533065 | 0,00024576 | 0,00395715 |
| NANOZOOG2513 | 15,279076 | 1,36694692 | 0,62861667 | 0,0055948 | 0,04237506 |
| NANOZOOG1264 | 27,8543982 | -1,3654749 | 0,42731191 | 0,00027585 | 0,00435033 |
| NANOZOOG1253 | 14,3425646 | -1,3652351 | 0,55226632 | 0,0023722 | 0,02243114 |
| NANOZOOG9162 | 15,715671 | -1,3614809 | 0,50138948 | 0,00141015 | 0,01499533 |
| NANOZOOG4584 | 32,8586823 | 1,3603831 | 0,48641194 | 0,00113588 | 0,01257916 |
| NANOZOOG6722 | 25,1003389 | 1,3584078 | 0,41933891 | 0,00026259 | 0,00418415 |
| NANOZOOG8673 | 12,4990099 | -1,3578851 | 0,53791053 | 0,00275345 | 0,02520806 |
| NANOZOOG7353 | 22,206953 | -1,3575713 | 0,44740033 | 0,00051046 | 0,00693406 |
| NANOZOOG8339 | 14,0287731 | -1,349985 | 0,44278309 | 0,00050982 | 0,00693406 |
| NANOZOOG8674 | 26,5356347 | -1,3409089 | 0,3764785 | 8,1118E-05 | 0,00163838 |
| NANOZOOG4005 | 47,0384078 | 1,33586354 | 0,36898337 | 7,5671E-05 | 0,0015529 |
| NANOZOOG2819 | 12,1463768 | -1,3358079 | 0,47259898 | 0,00109186 | 0,01220389 |
| NANOZOOG6773 | 104,535517 | -1,3342299 | 0,34377416 | 2,3357E-05 | 0,00061139 |
| NANOZOOG6987 | 12,1107146 | -1,3338521 | 0,546933 | 0,00299032 | 0,02710715 |
| NANOZOOG7214 | 2522,45957 | -1,3333878 | 0,21645591 | 1,6812E-10 | 2,2771E-08 |
| NANOZOOG2117 | 11,0425799 | -1,3268513 | 0,56728854 | 0,00395707 | 0,03337418 |
| NANOZOOG8484 | 58,6979507 | -1,3259891 | 0,27985313 | 4,7285E-07 | 2,1559E-05 |
| NANOZOOG7607 | 56,4046615 | -1,3250502 | 0,35010069 | 3,333E-05 | 0,00078711 |
| NANOZOOG3400 | 29,1425395 | -1,3218694 | 0,44842467 | 0,00082846 | 0,00986704 |
| NANOZOOG8933 | 22,1465828 | -1,3213694 | 0,44048773 | 0,00061141 | 0,00799866 |
| NANOZOOG2447 | 10,823816 | -1,3087207 | 0,50569121 | 0,00207392 | 0,02019113 |
| NANOZOOG30 | 28,0404458 | -1,3073797 | 0,31483404 | 8,1906E-06 | 0,00025485 |
| NANOZOOG1695 | 19,5958836 | -1,3072397 | 0,45420612 | 0,00099908 | 0,01144471 |
| NANOZOOG8207 | 14,6180791 | -1,304281 | 0,45285287 | 0,00090674 | 0,01054432 |
| NANOZOOG1011 | 10,6596042 | -1,2880124 | 0,57563608 | 0,00598085 | 0,04461859 |
| NANOZOOG1567 | 149,708367 | -1,2823198 | 0,46428638 | 0,00118287 | 0,01296938 |
| NANOZOOG8257 | 25,5301412 | -1,2817385 | 0,33199147 | 2,3367E-05 | 0,00061139 |
| NANOZOOG7760 | 15,7436501 | 1,27964726 | 0,44760073 | 0,00106584 | 0,01202988 |
| NANOZOOG1495 | 30,5766311 | -1,2761595 | 0,33448166 | 3,588E-05 | 0,00084299 |
| NANOZOOG79 | 15,758676 | -1,2740516 | 0,573474 | 0,00646869 | 0,04735821 |
| NANOZOOG1498 | 18,0751642 | -1,2733331 | 0,48069231 | 0,00202097 | 0,01980121 |
| NANOZOOG8654 | 11,0243472 | -1,2694599 | 0,53971907 | 0,00460118 | 0,03691363 |
| NANOZOOG7884 | 15,5591625 | -1,2692635 | 0,51749332 | 0,00290283 | 0,02641807 |
| NANOZOOG9083 | 64,5352375 | 1,26495687 | 0,45540513 | 0,00125035 | 0,01357989 |
| NANOZOOG3671 | 20,2388803 | 1,26482272 | 0,57051401 | 0,00607342 | 0,04510982 |
| NANOZOOG1692 | 50,4930618 | -1,2648209 | 0,50679602 | 0,0025643 | 0,02384426 |
| NANOZOOG9124 | 23,3447121 | -1,2580449 | 0,39364249 | 0,00035314 | 0,00526282 |
| NANOZOOG131 | 36,2054342 | -1,2533905 | 0,3280026 | 3,2113E-05 | 0,00077021 |
| NANOZOOG7226 | 5750,32271 | -1,2530413 | 0,21870939 | 2,5778E-09 | 2,4731E-07 |
| NANOZOOG8291 | 21,9078428 | -1,2508684 | 0,45419297 | 0,00135021 | 0,01452741 |
| NANOZOOG7078 | 8,43210382 | -1,245973 | 0,57082254 | 0,00654319 | 0,04782763 |
| NANOZOOG417 | 24,9549634 | 1,24539778 | 0,52108584 | 0,00421582 | 0,03466756 |
| NANOZOOG7238 | 24,9546703 | -1,2447873 | 0,39483817 | 0,00042217 | 0,00591448 |
| NANOZOOG1610 | 10,7561727 | -1,240264 | 0,55086094 | 0,0059879 | 0,04461859 |
| NANOZOOG3935 | 20,8992705 | -1,2402069 | 0,39735189 | 0,00046628 | 0,00642881 |
| NANOZOOG6423 | 30,3310807 | -1,2399158 | 0,33228802 | 4,9309E-05 | 0,0011052 |
| NANOZOOG8109 | 14,7780065 | -1,2319109 | 0,53002219 | 0,0045273 | 0,03646324 |
| NANOZOOG6126 | 17,5655218 | -1,223006 | 0,50047208 | 0,00336548 | 0,02945001 |
| NANOZOOG3995 | 12,7690948 | -1,2207931 | 0,50731664 | 0,00386938 | 0,03281493 |
| NANOZOOG74 | 19,4526118 | -1,2186922 | 0,52848578 | 0,00468862 | 0,03741959 |
| NANOZOOG5508 | 13,4016071 | -1,2169874 | 0,49587938 | 0,00322923 | 0,02848774 |
| NANOZOOG3691 | 21,3709909 | -1,2147291 | 0,44928947 | 0,00180242 | 0,01792684 |
| NANOZOOG8670 | 21,2520019 | 1,21269529 | 0,48870836 | 0,00350578 | 0,03040322 |
| NANOZOOG9428 | 44,7839882 | -1,2032559 | 0,28392094 | 5,9029E-06 | 0,00018877 |
| NANOZOOG6241 | 25,0388457 | -1,2015506 | 0,47029852 | 0,0024962 | 0,02335114 |
| NANOZOOG7743 | 40,8630416 | -1,1915881 | 0,45959143 | 0,00224423 | 0,02157556 |
| NANOZOOG6714 | 22,3031579 | 1,18721956 | 0,53274876 | 0,00634807 | 0,04669788 |
| NANOZOOG2372 | 57,0028491 | 1,185642 | 0,49902366 | 0,00430014 | 0,03517252 |
| NANOZOOG3248 | 36,8813606 | 1,18347815 | 0,48883414 | 0,00399675 | 0,03339266 |
| NANOZOOG5864 | 16,0172033 | -1,1807807 | 0,44887151 | 0,0024522 | 0,02299874 |
| NANOZOOG3327 | 107,000692 | -1,179606 | 0,29648361 | 2,0285E-05 | 0,00054627 |
| NANOZOOG8739 | 21,3710524 | -1,1739001 | 0,39190577 | 0,00074501 | 0,00910024 |
| NANOZOOG3308 | 22,4650698 | 1,17247606 | 0,48158294 | 0,00406902 | 0,0337619 |
| NANOZOOG7567 | 18,1959514 | -1,1675491 | 0,49744319 | 0,00444627 | 0,03611121 |
| NANOZOOG498 | 478,853077 | -1,1657921 | 0,37744352 | 0,00052085 | 0,00701321 |
| NANOZOOG3254 | 31,9440895 | 1,15943882 | 0,41695211 | 0,00157711 | 0,01628382 |
| NANOZOOG7009 | 25,7465831 | -1,158807 | 0,37567868 | 0,00061385 | 0,00800787 |
| NANOZOOG4351 | 9,81411027 | -1,1586646 | 0,5049139 | 0,00605002 | 0,04500864 |
| NANOZOOG1603 | 27,6085539 | -1,1561019 | 0,32281286 | 0,00010265 | 0,00200302 |
| NANOZOOG7931 | 29,1579173 | -1,1542119 | 0,34322071 | 0,000221 | 0,00368734 |
| NANOZOOG1509 | 32,7813829 | -1,1497547 | 0,47282053 | 0,0038305 | 0,03260529 |
| NANOZOOG3325 | 14,7071672 | -1,1488644 | 0,47030243 | 0,00391314 | 0,03312504 |
| NANOZOOG6042 | 15,3136702 | -1,1484648 | 0,48947996 | 0,00529662 | 0,04085581 |
| NANOZOOG5923 | 86,5906967 | -1,1445813 | 0,48683587 | 0,0045292 | 0,03646324 |
| NANOZOOG3046 | 31,2916822 | -1,1395661 | 0,38539213 | 0,00082913 | 0,00986704 |
| NANOZOOG1460 | 31,8335811 | -1,1352067 | 0,32900245 | 0,00016663 | 0,00294215 |
| NANOZOOG7038 | 23,8969797 | -1,1332723 | 0,36283324 | 0,00051312 | 0,00694982 |
| NANOZOOG5583 | 24,0544357 | -1,1317822 | 0,34826836 | 0,00036249 | 0,00532692 |
| NANOZOOG1808 | 173,562425 | -1,1254527 | 0,28953066 | 2,9216E-05 | 0,00072332 |
| NANOZOOG334 | 25,9292429 | -1,1212558 | 0,39385267 | 0,00130759 | 0,01413484 |
| NANOZOOG5541 | 39,396568 | -1,1210246 | 0,3139188 | 0,00010242 | 0,00200302 |
| NANOZOOG4810 | 24,7396756 | -1,117384 | 0,46757087 | 0,00441383 | 0,03591112 |
| NANOZOOG3176 | 20,9260153 | -1,1097567 | 0,39911918 | 0,00157321 | 0,01628007 |
| NANOZOOG8741 | 28,704774 | -1,1040126 | 0,43948717 | 0,00342749 | 0,02983665 |
| NANOZOOG8634 | 15,7716973 | -1,0993259 | 0,47027482 | 0,00536959 | 0,04096215 |
| NANOZOOG6556 | 25,3044418 | -1,0992603 | 0,4200308 | 0,00262497 | 0,02422442 |
| NANOZOOG1794 | 19,0725323 | -1,0923038 | 0,41032212 | 0,00223471 | 0,02155178 |
| NANOZOOG5019 | 24,8251059 | 1,08652192 | 0,4676522 | 0,00593827 | 0,04439244 |
| NANOZOOG7096 | 19,7154562 | -1,0810562 | 0,45200985 | 0,00490942 | 0,03871214 |
| NANOZOOG3569 | 13,8692435 | -1,0527692 | 0,41948674 | 0,00337974 | 0,02947667 |
| NANOZOOG7904 | 1697,36501 | -1,0428598 | 0,29474114 | 0,00012068 | 0,00229636 |
| NANOZOOG738 | 41,0503112 | -1,0344172 | 0,3269542 | 0,00053835 | 0,00722765 |
| NANOZOOG2910 | 25,4457077 | -1,0229967 | 0,3958094 | 0,00316356 | 0,0281239 |
| NANOZOOG2163 | 24,2316025 | -1,0169643 | 0,35577214 | 0,00145975 | 0,01538246 |
| NANOZOOG476 | 24,6642193 | -0,9988963 | 0,3841667 | 0,0032061 | 0,02833798 |
| NANOZOOG799 | 24,2234808 | -0,9970504 | 0,36647469 | 0,00237956 | 0,02245467 |
| NANOZOOG5604 | 22,514905 | -0,981784 | 0,40155876 | 0,00494959 | 0,03889564 |
| NANOZOOG8187 | 614,960848 | -0,9725194 | 0,34246004 | 0,00156729 | 0,01628007 |
| NANOZOOG328 | 23,0091935 | -0,9703642 | 0,33953082 | 0,00159268 | 0,01640781 |
| NANOZOOG6648 | 43,9861944 | 0,96693565 | 0,36841941 | 0,00326959 | 0,02878868 |
| NANOZOOG3197 | 36,3278615 | -0,952211 | 0,37636562 | 0,00432093 | 0,03527997 |
| NANOZOOG4645 | 39,3667043 | -0,9187881 | 0,28792686 | 0,0005685 | 0,00754455 |
| NANOZOOG7030 | 21,5246938 | -0,8912641 | 0,3542531 | 0,0048549 | 0,0384138 |
| NANOZOOG516 | 43,2329563 | -0,8821883 | 0,36064929 | 0,00611097 | 0,04531563 |
| NANOZOOG2409 | 31,9922885 | 0,88143199 | 0,34793428 | 0,00472029 | 0,03760716 |
| NANOZOOG1312 | 37,2121983 | -0,8635567 | 0,30789834 | 0,00223708 | 0,02155178 |
| NANOZOOG7222 | 20001,2538 | -0,8557012 | 0,1528734 | 1,0119E-08 | 8,0339E-07 |
| NANOZOOG3194 | 31,2641465 | 0,85008739 | 0,33997294 | 0,00566633 | 0,04270614 |
| NANOZOOG4154 | 20,0602045 | -0,8106901 | 0,32383433 | 0,00582891 | 0,04364575 |
| NANOZOOG4885 | 709,774022 | -0,8049158 | 0,29949942 | 0,00337028 | 0,02945001 |
| NANOZOOG122 | 1831,64942 | -0,7847761 | 0,23429852 | 0,00038718 | 0,00558607 |
| NANOZOOG3246 | 93,2281128 | -0,7817352 | 0,26858927 | 0,00179467 | 0,01790751 |
| NANOZOOG7220 | 3551,93305 | -0,7574853 | 0,25724178 | 0,00175123 | 0,01764643 |
| NANOZOOG7219 | 250825,212 | -0,7390034 | 0,19938043 | 0,00011266 | 0,00217984 |
| NANOZOOG568 | 412,600457 | -0,6885889 | 0,2697607 | 0,00636764 | 0,04676714 |

Mean of normalized counts, fold-change, fold-change standard error, and p-values in genes that were differentially expressed between the samples CGHH 10 h and CG 10 h. CGHH 10 h and CG 10 h samples belong to three independent cultivations in culture medium containing as carbon source either CGHH (a mixture of hemicellulose hydrolysate and crude glycerol) or CG (crude glycerol), respectively at 10 h. Data was obtained using RNA-Seq.

Table S2: Differentially expressed genes between growth on different media when glycerol consumption became visible

| **Gene ID** | **baseMean** | **log2FoldChange** | **lfcSE** | **pvalue** | **padj** |
| --- | --- | --- | --- | --- | --- |
| NANOZOOG5684 | 8,45646196 | -8,0779929 | 2,70776938 | 1,2049E-09 | 8,1703E-08 |
| NANOZOOG2859 | 43,548459 | 8,02302956 | 2,54721368 | 1,6803E-06 | 4,8135E-05 |
| NANOZOOG831 | 5,05141496 | -7,7396865 | 2,73314058 | 4,1421E-08 | 1,9636E-06 |
| NANOZOOG1345 | 4,53427256 | -7,4593826 | 2,79647726 | 7,6182E-07 | 2,4947E-05 |
| NANOZOOG3179 | 104,649965 | 7,06654138 | 2,21570751 | 6,0172E-07 | 2,0796E-05 |
| NANOZOOG8742 | 3,95562238 | -6,9233835 | 2,72204597 | 2,6389E-06 | 7,2496E-05 |
| NANOZOOG1824 | 3,68107888 | -6,8471187 | 2,61680957 | 6,3316E-07 | 2,1604E-05 |
| NANOZOOG908 | 3,49896372 | -6,7088297 | 2,86268832 | 2,67E-05 | 0,00052201 |
| NANOZOOG8056 | 22,7808221 | -6,680394 | 0,89313137 | 5,7459E-14 | 9,3275E-12 |
| NANOZOOG8818 | 3,52151501 | -6,4673722 | 2,59436176 | 2,9827E-06 | 7,9494E-05 |
| NANOZOOG9335 | 3,29075786 | -6,3917474 | 2,96682162 | 0,00011647 | 0,00191971 |
| NANOZOOG2231 | 37,8220243 | -6,1623702 | 0,74800752 | 6,3913E-17 | 2,2825E-14 |
| NANOZOOG825 | 11,1852881 | -6,1531326 | 1,0806821 | 8,4834E-09 | 4,7837E-07 |
| NANOZOOG4618 | 2,50390674 | -6,079872 | 2,75497092 | 6,4044E-05 | 0,00114361 |
| NANOZOOG8336 | 7,71842985 | -6,0245128 | 1,36056649 | 3,3842E-07 | 1,2532E-05 |
| NANOZOOG901 | 33,4395467 | -5,8322871 | 0,54257627 | 2,8649E-27 | 7,6736E-24 |
| NANOZOOG9299 | 3,32423773 | -5,8266291 | 2,52905925 | 2,0019E-05 | 0,00040322 |
| NANOZOOG5876 | 3,67104235 | -5,5173441 | 2,64617565 | 0,00014041 | 0,00224533 |
| NANOZOOG4877 | 33,2818588 | -5,4989581 | 0,56773769 | 4,4799E-23 | 3,9998E-20 |
| NANOZOOG8232 | 14,5412779 | -5,4926941 | 0,79871053 | 3,607E-12 | 4,1112E-10 |
| NANOZOOG9349 | 4,3139847 | -5,4044954 | 2,59088544 | 0,00013255 | 0,00213877 |
| NANOZOOG6006 | 8,41460271 | -5,3539721 | 1,32832741 | 3,2855E-06 | 8,6276E-05 |
| NANOZOOG9075 | 2,63027565 | -5,230995 | 2,48061408 | 9,7295E-05 | 0,00165463 |
| NANOZOOG9040 | 4,95491646 | -5,1659454 | 1,38132511 | 1,1119E-05 | 0,00024922 |
| NANOZOOG1250 | 26,8430478 | 5,13582401 | 1,88593103 | 7,0413E-06 | 0,00016839 |
| NANOZOOG826 | 4,07082745 | -5,1041426 | 1,40869624 | 1,7353E-05 | 0,00035854 |
| NANOZOOG999 | 2,9920704 | -5,0937312 | 2,43336443 | 9,875E-05 | 0,00166878 |
| NANOZOOG2281 | 16,5904486 | -5,0933615 | 0,71311798 | 2,1064E-13 | 2,9695E-11 |
| NANOZOOG827 | 5,87716147 | -5,0906525 | 1,18512423 | 4,2201E-06 | 0,00010756 |
| NANOZOOG7746 | 6,17723086 | -4,9637345 | 1,30299791 | 9,5149E-06 | 0,00021876 |
| NANOZOOG2947 | 2,93676794 | 4,90375344 | 2,25553458 | 0,00019002 | 0,00285972 |
| NANOZOOG52 | 22,3607474 | -4,8820569 | 0,75529564 | 1,9688E-11 | 1,9531E-09 |
| NANOZOOG387 | 4,10252047 | -4,857098 | 1,31528998 | 1,5194E-05 | 0,00032157 |
| NANOZOOG4308 | 2,91931265 | -4,8562789 | 2,59143935 | 0,00046318 | 0,00606659 |
| NANOZOOG8868 | 8,73504862 | -4,8498052 | 1,13103565 | 2,8919E-06 | 7,8242E-05 |
| NANOZOOG1185 | 14,3867878 | -4,7542896 | 0,8005894 | 1,0341E-09 | 7,1024E-08 |
| NANOZOOG1 | 23,0651078 | -4,6110509 | 0,56777714 | 1,2543E-16 | 3,9525E-14 |
| NANOZOOG222 | 5,4943853 | 4,56694866 | 2,05720018 | 0,0001357 | 0,00218294 |
| NANOZOOG4175 | 31,2366973 | -4,5217943 | 0,49071793 | 6,3117E-21 | 4,2265E-18 |
| NANOZOOG7323 | 10,5015246 | -4,5094056 | 0,74216693 | 3,6231E-10 | 2,6957E-08 |
| NANOZOOG8470 | 7,52522337 | -4,480742 | 1,29133855 | 4,4683E-05 | 0,00083114 |
| NANOZOOG4484 | 9,30730228 | -4,4769533 | 0,80120234 | 9,7861E-09 | 5,3494E-07 |
| NANOZOOG1254 | 3,09792307 | 4,45294708 | 2,15635632 | 0,00025963 | 0,00371884 |
| NANOZOOG1161 | 3,68358528 | -4,4493414 | 2,40712154 | 0,00048068 | 0,00621981 |
| NANOZOOG4247 | 8,90049846 | -4,3080836 | 1,11788476 | 3,3206E-05 | 0,00063759 |
| NANOZOOG3509 | 35,5787406 | -4,2982968 | 0,41843169 | 1,8519E-25 | 2,4801E-22 |
| NANOZOOG7037 | 5,57069085 | -4,177359 | 0,9653655 | 2,9334E-06 | 7,8965E-05 |
| NANOZOOG2857 | 7,21868624 | 4,14989174 | 4,20422492 | 0,00484831 | 0,03780555 |
| NANOZOOG3743 | 2,501203 | -4,1159613 | 2,40321372 | 0,00096136 | 0,01095742 |
| NANOZOOG7182 | 35,8335813 | 4,1155971 | 1,35378944 | 0,00026226 | 0,00372656 |
| NANOZOOG2284 | 2,14762296 | -4,0873763 | 2,45508153 | 0,0012398 | 0,01330987 |
| NANOZOOG5926 | 5,36357943 | -4,0815451 | 2,28996273 | 0,00065856 | 0,00796373 |
| NANOZOOG4664 | 12,8881063 | -4,0555441 | 0,80872685 | 1,3298E-07 | 5,356E-06 |
| NANOZOOG2774 | 2,11359358 | -4,0549835 | 2,41626089 | 0,00111949 | 0,01231434 |
| NANOZOOG2713 | 42,2113882 | -4,0511971 | 0,52722448 | 9,6099E-16 | 2,34E-13 |
| NANOZOOG9230 | 2,06559299 | -4,0267202 | 2,41495721 | 0,00120579 | 0,0131289 |
| NANOZOOG124 | 3,34000107 | -3,9671395 | 1,33723419 | 0,0002412 | 0,00349222 |
| NANOZOOG270 | 2,07130042 | -3,9405884 | 2,44733268 | 0,00155802 | 0,01577751 |
| NANOZOOG7443 | 20,8187836 | -3,940058 | 0,5796774 | 1,548E-12 | 1,8846E-10 |
| NANOZOOG3552 | 5,1514999 | -3,9229481 | 1,37449737 | 0,00036198 | 0,00493423 |
| NANOZOOG4483 | 18,1789856 | -3,9040573 | 0,54782053 | 1,0745E-13 | 1,693E-11 |
| NANOZOOG3745 | 1,93409213 | -3,8885686 | 2,85691058 | 0,00342869 | 0,02957726 |
| NANOZOOG9172 | 3,69119507 | -3,8680027 | 1,33507824 | 0,00034668 | 0,004762 |
| NANOZOOG1272 | 5,88442772 | 3,85995843 | 2,033825 | 0,00067247 | 0,00811361 |
| NANOZOOG2579 | 7,57870551 | -3,8350654 | 0,88234798 | 1,9466E-06 | 5,4883E-05 |
| NANOZOOG3929 | 157,426197 | -3,8313 | 0,37758331 | 2,4126E-25 | 2,5849E-22 |
| NANOZOOG3431 | 3,50698555 | -3,8267406 | 1,5863601 | 0,0008131 | 0,00948973 |
| NANOZOOG50 | 8,96269717 | -3,798542 | 0,69480643 | 1,0248E-08 | 5,5453E-07 |
| NANOZOOG5423 | 6,26984574 | -3,7956601 | 0,94128052 | 1,032E-05 | 0,00023427 |
| NANOZOOG8393 | 5,43994606 | 3,75831505 | 1,99736738 | 0,00071194 | 0,00853216 |
| NANOZOOG9194 | 7,21389191 | -3,7386385 | 1,00764428 | 3,883E-05 | 0,00073243 |
| NANOZOOG3957 | 8,84741716 | 3,73220709 | 1,36422028 | 0,00050473 | 0,00648399 |
| NANOZOOG7282 | 26,3175213 | -3,6945239 | 0,4201912 | 2,4865E-19 | 1,332E-16 |
| NANOZOOG1128 | 7,00362487 | 3,68337259 | 2,06901218 | 0,00107482 | 0,01194568 |
| NANOZOOG6692 | 2,3319665 | -3,6832521 | 2,39390672 | 0,00204176 | 0,01963686 |
| NANOZOOG39 | 13,4721378 | -3,6814991 | 0,6620832 | 5,5017E-09 | 3,2388E-07 |
| NANOZOOG2230 | 6,98858248 | -3,6750797 | 1,2329666 | 0,00041173 | 0,00555576 |
| NANOZOOG4598 | 2,36301772 | -3,669336 | 1,56845039 | 0,00127683 | 0,0135714 |
| NANOZOOG3485 | 7,43183846 | -3,664327 | 0,99013988 | 3,6352E-05 | 0,00069057 |
| NANOZOOG8726 | 30,5702933 | -3,637432 | 0,42266948 | 5,5133E-19 | 2,685E-16 |
| NANOZOOG5970 | 11,455005 | -3,6362455 | 0,61587374 | 3,5752E-10 | 2,6957E-08 |
| NANOZOOG1365 | 3,55407649 | 3,62706138 | 2,17263484 | 0,00172691 | 0,01729167 |
| NANOZOOG7185 | 8,81165965 | -3,6157272 | 0,72048629 | 1,1059E-07 | 4,5572E-06 |
| NANOZOOG4171 | 30,0756713 | -3,6038783 | 0,39041669 | 3,4368E-21 | 2,6301E-18 |
| NANOZOOG4462 | 12,5806857 | 3,59302923 | 1,2306485 | 0,00042204 | 0,00562406 |
| NANOZOOG7175 | 111,962567 | 3,56469748 | 1,47017693 | 0,00056381 | 0,00710671 |
| NANOZOOG8421 | 12,6828744 | 3,54982619 | 1,30292879 | 0,00061522 | 0,00750739 |
| NANOZOOG1091 | 4,68627116 | 3,54980667 | 2,00130202 | 0,00112584 | 0,01233354 |
| NANOZOOG2000 | 25,825969 | -3,5423515 | 0,45311533 | 6,5296E-16 | 1,6657E-13 |
| NANOZOOG6834 | 2,80765906 | 3,54184118 | 2,08114942 | 0,00150981 | 0,01537654 |
| NANOZOOG3335 | 2,18590606 | -3,5187365 | 1,47605677 | 0,001097 | 0,01209185 |
| NANOZOOG3033 | 15,0686661 | -3,5182759 | 0,59101796 | 5,1403E-10 | 3,7373E-08 |
| NANOZOOG4755 | 16,4187158 | -3,5139843 | 0,50635014 | 3,5794E-13 | 4,9167E-11 |
| NANOZOOG6878 | 21,1081842 | 3,51013694 | 1,86385279 | 0,00074008 | 0,00881025 |
| NANOZOOG1956 | 11,282317 | 3,50926498 | 1,25330357 | 0,00056532 | 0,00710896 |
| NANOZOOG2399 | 10,4418958 | 3,50615222 | 1,91586164 | 0,00091683 | 0,01051341 |
| NANOZOOG3869 | 7,9985734 | -3,4682468 | 0,84105332 | 5,8512E-06 | 0,00014313 |
| NANOZOOG1976 | 4,88404986 | -3,4655064 | 1,43029752 | 0,00131111 | 0,01390819 |
| NANOZOOG3605 | 7,60163965 | -3,4531678 | 0,76639323 | 9,8362E-07 | 3,0996E-05 |
| NANOZOOG7027 | 53,9811206 | 3,44357253 | 1,58319793 | 0,00144326 | 0,0149546 |
| NANOZOOG3946 | 4,57725143 | 3,42647513 | 2,01223479 | 0,00145853 | 0,01501419 |
| NANOZOOG3925 | 6,42259336 | -3,4102075 | 2,34995499 | 0,00300755 | 0,02680777 |
| NANOZOOG3802 | 16,9069139 | -3,4090748 | 0,5100193 | 4,0036E-12 | 4,4682E-10 |
| NANOZOOG4510 | 8,79491967 | 3,39504141 | 1,19960773 | 0,0008047 | 0,00943281 |
| NANOZOOG179 | 2,76006567 | -3,3848467 | 1,23209652 | 0,00080739 | 0,0094436 |
| NANOZOOG6199 | 18,0383189 | -3,3675874 | 0,93924304 | 7,5417E-05 | 0,00131599 |
| NANOZOOG3816 | 5,64253235 | -3,3590074 | 0,93115512 | 6,1518E-05 | 0,00110588 |
| NANOZOOG9246 | 5,58339478 | 3,32942432 | 2,0399679 | 0,00189497 | 0,01851612 |
| NANOZOOG9106 | 2,25328124 | -3,3134054 | 1,8049513 | 0,00360285 | 0,03056549 |
| NANOZOOG4340 | 4,45634167 | -3,3132165 | 2,56364412 | 0,00443789 | 0,03563911 |
| NANOZOOG9451 | 5,97305545 | -3,3129602 | 0,83000507 | 8,8668E-06 | 0,00020586 |
| NANOZOOG2008 | 2,82783492 | 3,31195553 | 2,30095827 | 0,00361807 | 0,03057094 |
| NANOZOOG9037 | 6,25749743 | -3,3078338 | 0,78946853 | 4,3203E-06 | 0,00010866 |
| NANOZOOG3744 | 99,9214103 | -3,2855594 | 0,24352343 | 9,474E-43 | 5,0752E-39 |
| NANOZOOG4327 | 18,2657437 | -3,2783866 | 0,45832838 | 1,2022E-13 | 1,84E-11 |
| NANOZOOG721 | 163,131818 | -3,2650556 | 0,49926658 | 4,5998E-12 | 5,0288E-10 |
| NANOZOOG5110 | 42,2354385 | -3,2512709 | 0,46962739 | 4,1228E-13 | 5,5215E-11 |
| NANOZOOG9111 | 181,909701 | 3,24540043 | 0,37928798 | 3,0959E-18 | 1,2757E-15 |
| NANOZOOG1273 | 9,92322028 | -3,2316055 | 0,66351242 | 1,4918E-07 | 5,876E-06 |
| NANOZOOG3244 | 3,56279067 | -3,2150609 | 2,31329589 | 0,0035959 | 0,03056549 |
| NANOZOOG9252 | 25,4456035 | -3,1921544 | 0,40040476 | 2,4752E-16 | 6,9789E-14 |
| NANOZOOG3030 | 6,22407086 | 3,1880789 | 1,98294176 | 0,00208545 | 0,01987861 |
| NANOZOOG9007 | 11,511231 | -3,1567031 | 0,50030972 | 4,9732E-11 | 4,3674E-09 |
| NANOZOOG6144 | 14,5013254 | -3,1543168 | 0,66687445 | 2,8542E-07 | 1,0768E-05 |
| NANOZOOG6398 | 8,42230008 | -3,1518881 | 0,87928024 | 5,1105E-05 | 0,0009312 |
| NANOZOOG309 | 9,42737227 | -3,1456943 | 0,70788982 | 1,5426E-06 | 4,4913E-05 |
| NANOZOOG7869 | 11,1022052 | 3,13604669 | 1,95027145 | 0,00207245 | 0,0197899 |
| NANOZOOG7172 | 5,01193631 | -3,132337 | 0,82769288 | 3,5328E-05 | 0,00067349 |
| NANOZOOG2236 | 27,0982402 | -3,1174941 | 0,49693978 | 3,1733E-11 | 3,0908E-09 |
| NANOZOOG7962 | 5,25335823 | -3,1124559 | 0,96174418 | 0,00022748 | 0,00332947 |
| NANOZOOG3569 | 13,8692435 | -3,1104161 | 0,52927121 | 5,1626E-10 | 3,7373E-08 |
| NANOZOOG370 | 4,01663461 | -3,1087765 | 1,21267987 | 0,00149196 | 0,01525272 |
| NANOZOOG9265 | 2,35910373 | -3,1079863 | 2,50831557 | 0,00549865 | 0,04114003 |
| NANOZOOG9079 | 6,04701576 | -3,1060819 | 0,76734817 | 5,9502E-06 | 0,00014489 |
| NANOZOOG2702 | 10,2813408 | 3,08949975 | 1,9214556 | 0,00217098 | 0,02047326 |
| NANOZOOG2795 | 35,6566274 | -3,0885861 | 0,50936753 | 9,5248E-11 | 7,9725E-09 |
| NANOZOOG3635 | 9,19352346 | 3,08783135 | 1,96352729 | 0,00236538 | 0,02202311 |
| NANOZOOG8694 | 11,5129247 | 3,08005149 | 1,22094914 | 0,00142687 | 0,0148135 |
| NANOZOOG2345 | 23,684122 | 3,07326443 | 1,14549737 | 0,00127136 | 0,0135714 |
| NANOZOOG3685 | 10,1791164 | -3,0704267 | 0,81230869 | 2,0299E-05 | 0,00040727 |
| NANOZOOG4082 | 4,33004604 | -3,0557027 | 2,53315317 | 0,00617409 | 0,04493832 |
| NANOZOOG9296 | 31,7672599 | 3,04311924 | 0,665653 | 1,7076E-06 | 4,8659E-05 |
| NANOZOOG109 | 4,46949426 | -2,998687 | 1,42413117 | 0,00309421 | 0,02739786 |
| NANOZOOG694 | 29,3016347 | -2,9960786 | 0,41068318 | 2,8547E-14 | 4,9394E-12 |
| NANOZOOG5154 | 7,0747515 | -2,995942 | 0,80259911 | 2,2085E-05 | 0,00043982 |
| NANOZOOG648 | 11,0714378 | 2,98052948 | 1,25171275 | 0,00188549 | 0,01846541 |
| NANOZOOG8889 | 2,00654914 | -2,9804787 | 2,85095982 | 0,0073395 | 0,04995894 |
| NANOZOOG4216 | 3,85030227 | -2,9755713 | 1,4285614 | 0,003203 | 0,02817484 |
| NANOZOOG3861 | 42,2750459 | -2,9675673 | 0,37379297 | 1,8542E-16 | 5,5183E-14 |
| NANOZOOG8100 | 5,77183822 | 2,96500221 | 1,98772924 | 0,00311172 | 0,02750741 |
| NANOZOOG3329 | 64,4655368 | -2,9600377 | 0,28832413 | 7,9054E-26 | 1,4116E-22 |
| NANOZOOG3548 | 121,478873 | -2,946923 | 0,38545496 | 1,9576E-15 | 4,5594E-13 |
| NANOZOOG7252 | 14,0752447 | -2,9418244 | 0,68165222 | 1,2326E-06 | 3,658E-05 |
| NANOZOOG6866 | 3,38698148 | 2,93959734 | 2,02596355 | 0,00357852 | 0,03052568 |
| NANOZOOG4519 | 7,03798722 | -2,937414 | 1,37402943 | 0,00375244 | 0,03136011 |
| NANOZOOG1567 | 149,708367 | -2,9200981 | 0,47911322 | 9,1019E-11 | 7,7395E-09 |
| NANOZOOG740 | 32,2520998 | -2,9157676 | 0,47140231 | 4,8131E-11 | 4,2973E-09 |
| NANOZOOG6809 | 26,9593259 | 2,90697351 | 0,8379587 | 0,00017283 | 0,00264526 |
| NANOZOOG7018 | 8,83682688 | -2,9015558 | 0,76323016 | 1,8838E-05 | 0,00038665 |
| NANOZOOG8598 | 2,60055592 | -2,8984972 | 2,51837793 | 0,00687899 | 0,04823395 |
| NANOZOOG4825 | 4,29135395 | -2,8934118 | 1,12856035 | 0,00197658 | 0,01921693 |
| NANOZOOG7125 | 13,5137557 | -2,8720314 | 1,30161646 | 0,00138953 | 0,01456693 |
| NANOZOOG5058 | 154,16404 | -2,8662309 | 0,39453057 | 3,1608E-14 | 5,2913E-12 |
| NANOZOOG7850 | 9,10065199 | 2,86589542 | 0,95218999 | 0,00046534 | 0,00607789 |
| NANOZOOG3968 | 4,47658407 | -2,8649516 | 1,0731577 | 0,00104331 | 0,01166813 |
| NANOZOOG2619 | 26,6243649 | 2,84922001 | 1,01816929 | 0,00077953 | 0,00919816 |
| NANOZOOG4823 | 18,5821301 | 2,83895565 | 1,465356 | 0,0030518 | 0,02706707 |
| NANOZOOG8257 | 25,5301412 | -2,8368565 | 0,38579276 | 2,2442E-14 | 4,3998E-12 |
| NANOZOOG6877 | 6,0142644 | -2,8305863 | 0,90248682 | 0,00041956 | 0,00561889 |
| NANOZOOG8597 | 1,91975941 | -2,8304726 | 1,89808045 | 0,00699223 | 0,04870921 |
| NANOZOOG1935 | 29,7560599 | 2,8181518 | 0,73506395 | 1,5882E-05 | 0,00033364 |
| NANOZOOG4610 | 9,66274566 | -2,8141361 | 0,64504569 | 1,2473E-06 | 3,6713E-05 |
| NANOZOOG5127 | 16,7907299 | -2,8052848 | 0,4974362 | 2,2469E-09 | 1,4679E-07 |
| NANOZOOG4472 | 37,413628 | 2,8039208 | 0,55152717 | 4,5695E-08 | 2,1286E-06 |
| NANOZOOG4706 | 28,7168902 | 2,80111445 | 1,38446431 | 0,00462073 | 0,03657928 |
| NANOZOOG3066 | 8,47581688 | -2,785039 | 1,01396881 | 0,00069744 | 0,00837706 |
| NANOZOOG4566 | 7,88599152 | 2,78271561 | 2,19359948 | 0,00544924 | 0,04097368 |
| NANOZOOG393 | 4,13647544 | 2,77537861 | 2,04991012 | 0,00478197 | 0,03740963 |
| NANOZOOG8594 | 2,76040573 | -2,7751136 | 1,30823637 | 0,00241221 | 0,02235677 |
| NANOZOOG6535 | 3,93181041 | -2,7712729 | 0,84459857 | 0,000165 | 0,00256947 |
| NANOZOOG6990 | 50,2147725 | -2,7711379 | 0,37706604 | 2,0112E-14 | 4,1438E-12 |
| NANOZOOG1722 | 18,8435701 | 2,76947246 | 0,9199567 | 0,00060664 | 0,00743521 |
| NANOZOOG5287 | 21,7280131 | 2,76699096 | 2,09451158 | 0,00478357 | 0,03740963 |
| NANOZOOG4737 | 11,5623122 | -2,7636798 | 1,31451566 | 0,00394652 | 0,03263642 |
| NANOZOOG8207 | 14,6180791 | -2,7635006 | 0,55678439 | 1,1021E-07 | 4,5572E-06 |
| NANOZOOG3769 | 1,89392755 | -2,7594001 | 1,57069144 | 0,00584829 | 0,04327252 |
| NANOZOOG9176 | 2,62886232 | -2,758168 | 1,40898259 | 0,00535986 | 0,04067503 |
| NANOZOOG2134 | 6,80318106 | -2,7496783 | 0,77976847 | 7,7944E-05 | 0,00135128 |
| NANOZOOG7657 | 4,03197367 | 2,74313246 | 1,35782293 | 0,00444511 | 0,03563911 |
| NANOZOOG3460 | 77,9679154 | -2,7373042 | 0,38702589 | 1,4233E-13 | 2,1179E-11 |
| NANOZOOG4156 | 3,42026242 | -2,7201104 | 1,36068512 | 0,00462277 | 0,03657928 |
| NANOZOOG2150 | 206,475206 | -2,7188911 | 0,39663863 | 6,1623E-13 | 7,8598E-11 |
| NANOZOOG1981 | 3,62636698 | -2,7184978 | 1,50830607 | 0,00594243 | 0,0437876 |
| NANOZOOG3350 | 7,20093982 | -2,7182092 | 0,87657692 | 0,00024859 | 0,00357054 |
| NANOZOOG4562 | 29,9400609 | 2,71590909 | 0,92202209 | 0,00068726 | 0,00827342 |
| NANOZOOG6541 | 6,10420952 | -2,684766 | 1,26911071 | 0,00451512 | 0,03593985 |
| NANOZOOG6180 | 7,77520469 | -2,6802195 | 0,88598995 | 0,00034103 | 0,00469646 |
| NANOZOOG56 | 12,6994121 | -2,6782471 | 0,58755146 | 6,1889E-07 | 2,1252E-05 |
| NANOZOOG1932 | 17,9773575 | -2,6699628 | 0,50597137 | 1,3185E-08 | 6,934E-07 |
| NANOZOOG6550 | 181,802627 | -2,6681586 | 0,51526359 | 2,1307E-08 | 1,0768E-06 |
| NANOZOOG6924 | 5,62550157 | -2,6651677 | 0,94596084 | 0,00097627 | 0,01105685 |
| NANOZOOG1153 | 11,3741699 | -2,6623466 | 0,80743567 | 0,00015386 | 0,00242425 |
| NANOZOOG5303 | 4,85580777 | 2,65944249 | 2,11118466 | 0,0061581 | 0,0448829 |
| NANOZOOG3586 | 10,4018158 | -2,652654 | 0,58132618 | 6,5883E-07 | 2,2197E-05 |
| NANOZOOG6870 | 22,2073953 | -2,6493797 | 0,51397122 | 2,5056E-08 | 1,2428E-06 |
| NANOZOOG7826 | 14,4277573 | 2,64843858 | 0,87753397 | 0,00060792 | 0,00743521 |
| NANOZOOG2272 | 6,41044532 | 2,64341926 | 1,96467612 | 0,00495722 | 0,03848671 |
| NANOZOOG8010 | 60,9779655 | -2,6393406 | 0,57753179 | 5,1834E-07 | 1,8171E-05 |
| NANOZOOG2075 | 3,88112672 | -2,626325 | 1,2595007 | 0,00320909 | 0,02818215 |
| NANOZOOG3166 | 43,5967538 | -2,6243737 | 0,84178847 | 0,00015038 | 0,00237633 |
| NANOZOOG7324 | 128,578775 | 2,62210808 | 0,76240759 | 4,7409E-05 | 0,00087576 |
| NANOZOOG6391 | 14,2809983 | -2,6207412 | 0,80319357 | 0,00014193 | 0,00225616 |
| NANOZOOG3574 | 5,05974515 | -2,6119886 | 0,85459425 | 0,00028887 | 0,00404044 |
| NANOZOOG3643 | 8,35097494 | -2,6093809 | 0,7039171 | 3,124E-05 | 0,00060353 |
| NANOZOOG1241 | 40,2623149 | -2,6081081 | 0,59639462 | 9,5705E-07 | 3,0337E-05 |
| NANOZOOG6040 | 3,43837025 | 2,60284643 | 2,11388556 | 0,00692195 | 0,0484085 |
| NANOZOOG2943 | 2,67968448 | -2,5970102 | 1,73978551 | 0,00686603 | 0,0482062 |
| NANOZOOG6840 | 4,72186998 | -2,5954763 | 0,90212123 | 0,00056956 | 0,00713655 |
| NANOZOOG1610 | 10,7561727 | -2,5905997 | 0,6954895 | 3,7918E-05 | 0,00071777 |
| NANOZOOG4787 | 342,78408 | -2,5903996 | 0,35376686 | 2,3818E-14 | 4,3998E-12 |
| NANOZOOG4278 | 3,24611363 | -2,5850971 | 1,15456402 | 0,00439003 | 0,03541779 |
| NANOZOOG4172 | 2,54935841 | 2,58157037 | 2,11791102 | 0,0070768 | 0,0490432 |
| NANOZOOG8820 | 5,50631122 | 2,58155226 | 1,03510341 | 0,00349459 | 0,03004898 |
| NANOZOOG7884 | 15,5591625 | -2,5806668 | 0,57812168 | 8,8931E-07 | 2,8527E-05 |
| NANOZOOG5236 | 308,655894 | -2,576112 | 0,3035756 | 1,9903E-18 | 8,8851E-16 |
| NANOZOOG5922 | 7,00134917 | -2,5746146 | 0,68691067 | 3,08E-05 | 0,00059781 |
| NANOZOOG2192 | 3,25885383 | -2,5737718 | 1,44503961 | 0,00693554 | 0,0484403 |
| NANOZOOG8224 | 6,90999857 | -2,5737028 | 0,93510843 | 0,00123205 | 0,0132799 |
| NANOZOOG2923 | 19,9575495 | -2,5727695 | 0,46160571 | 3,0467E-09 | 1,8978E-07 |
| NANOZOOG9155 | 4,04200725 | 2,57136807 | 2,03270494 | 0,00625851 | 0,04526607 |
| NANOZOOG3159 | 5,90295388 | 2,56953563 | 1,44936778 | 0,0060109 | 0,04404979 |
| NANOZOOG5564 | 6,84686803 | -2,5654666 | 0,8098552 | 0,00024115 | 0,00349222 |
| NANOZOOG4496 | 14,5242335 | 2,56396603 | 1,30003832 | 0,00470913 | 0,03704375 |
| NANOZOOG4031 | 39,2449135 | -2,5611532 | 0,56507279 | 5,8368E-07 | 2,0304E-05 |
| NANOZOOG5690 | 16,0600813 | -2,5518363 | 0,58906959 | 1,5817E-06 | 4,5801E-05 |
| NANOZOOG5238 | 65,4679197 | -2,5503336 | 0,36090887 | 1,6012E-13 | 2,3184E-11 |
| NANOZOOG8574 | 5,05225693 | 2,55019368 | 1,98090131 | 0,00596368 | 0,0438273 |
| NANOZOOG7654 | 19,6567899 | -2,5371566 | 0,55919881 | 6,9836E-07 | 2,3237E-05 |
| NANOZOOG1046 | 16,7289934 | -2,5370401 | 0,66831207 | 1,3622E-05 | 0,00029425 |
| NANOZOOG188 | 71,2275968 | -2,5331162 | 0,56519735 | 7,6372E-07 | 2,4947E-05 |
| NANOZOOG2598 | 10,2384527 | 2,53234832 | 1,02545475 | 0,0036077 | 0,03056549 |
| NANOZOOG7973 | 49,5229523 | -2,5303444 | 0,33159811 | 3,3481E-15 | 7,4732E-13 |
| NANOZOOG4488 | 9,73040215 | 2,52750761 | 1,01809507 | 0,00356482 | 0,03050566 |
| NANOZOOG1340 | 10,8461963 | -2,5267056 | 0,65904737 | 1,9448E-05 | 0,00039614 |
| NANOZOOG1965 | 17,301247 | -2,5219851 | 0,64739975 | 1,1532E-05 | 0,00025633 |
| NANOZOOG7781 | 6,12350139 | 2,52006773 | 1,98831477 | 0,00626756 | 0,04526607 |
| NANOZOOG9168 | 5,4353898 | -2,5188378 | 0,89843761 | 0,00076555 | 0,00905314 |
| NANOZOOG8822 | 13,9026056 | -2,5168095 | 0,5010614 | 7,8655E-08 | 3,4257E-06 |
| NANOZOOG1253 | 14,3425646 | -2,5119595 | 0,60520611 | 3,9318E-06 | 0,00010078 |
| NANOZOOG2632 | 5,69773691 | -2,5047073 | 0,75776882 | 0,00016633 | 0,00258116 |
| NANOZOOG1049 | 207,189312 | 2,50397973 | 1,29496871 | 0,00251965 | 0,02323191 |
| NANOZOOG4810 | 24,7396756 | -2,5036231 | 0,53028448 | 2,777E-07 | 1,0551E-05 |
| NANOZOOG1494 | 10,2749456 | -2,5010605 | 0,63018201 | 1,1999E-05 | 0,00026562 |
| NANOZOOG1018 | 4,37249052 | -2,4949587 | 1,10536516 | 0,0049884 | 0,03867273 |
| NANOZOOG1053 | 12,5007213 | 2,49283947 | 0,83318327 | 0,00053167 | 0,0067813 |
| NANOZOOG3843 | 24,304072 | -2,4878038 | 0,42334675 | 5,4073E-10 | 3,8623E-08 |
| NANOZOOG6327 | 27,5378976 | -2,4869767 | 0,38071247 | 8,0033E-12 | 8,5748E-10 |
| NANOZOOG2643 | 11,7726012 | 2,4863925 | 1,27250354 | 0,00553787 | 0,04137566 |
| NANOZOOG7004 | 10,2490233 | 2,48582136 | 1,24515069 | 0,0053814 | 0,04071775 |
| NANOZOOG4440 | 6,82740591 | 2,47945911 | 1,29114917 | 0,00613663 | 0,04478737 |
| NANOZOOG7785 | 4,23085564 | -2,4769268 | 1,198492 | 0,00648588 | 0,04626481 |
| NANOZOOG2169 | 8,19131867 | -2,4763291 | 0,74141226 | 9,8659E-05 | 0,00166878 |
| NANOZOOG7299 | 96,8537927 | -2,4756959 | 0,31502418 | 4,2836E-16 | 1,1474E-13 |
| NANOZOOG1531 | 18,6015567 | -2,4729688 | 0,6144642 | 6,188E-06 | 0,00014932 |
| NANOZOOG7247 | 23,7754943 | 2,47029909 | 1,11976527 | 0,00322355 | 0,02826281 |
| NANOZOOG9162 | 15,715671 | -2,4670656 | 0,56745481 | 2,1459E-06 | 6,0187E-05 |
| NANOZOOG2998 | 3,99042636 | -2,4574889 | 0,98491239 | 0,0021852 | 0,02053701 |
| NANOZOOG191 | 16,531987 | -2,4560684 | 0,61887172 | 8,8247E-06 | 0,00020586 |
| NANOZOOG6045 | 68,8618212 | -2,4551407 | 0,36744157 | 2,5479E-12 | 2,9672E-10 |
| NANOZOOG1692 | 50,4930618 | -2,4535005 | 0,5346629 | 4,5681E-07 | 1,6314E-05 |
| NANOZOOG4309 | 3,43096866 | -2,4532123 | 1,12189937 | 0,00516311 | 0,0397968 |
| NANOZOOG4261 | 5,11781988 | -2,4522628 | 0,93607438 | 0,00126648 | 0,01356912 |
| NANOZOOG7863 | 81,1573194 | -2,4512678 | 0,59322412 | 3,3772E-06 | 8,8252E-05 |
| NANOZOOG5807 | 22,5706831 | -2,4485144 | 0,4948814 | 9,5219E-08 | 4,0483E-06 |
| NANOZOOG6300 | 10,0645397 | -2,442108 | 0,58226544 | 3,4629E-06 | 9,0053E-05 |
| NANOZOOG994 | 62,3213066 | -2,4385708 | 0,32787189 | 9,1858E-15 | 1,9683E-12 |
| NANOZOOG2674 | 4,76514409 | -2,4372906 | 1,1067638 | 0,00390274 | 0,03236372 |
| NANOZOOG4009 | 5,13981775 | -2,4318017 | 0,76945296 | 0,00021895 | 0,00322223 |
| NANOZOOG7005 | 26,3098141 | 2,42800357 | 0,72963668 | 0,00018208 | 0,00275543 |
| NANOZOOG4103 | 19,6187156 | -2,4255057 | 0,43837718 | 5,4778E-09 | 3,2388E-07 |
| NANOZOOG4949 | 49,6504217 | -2,4249352 | 0,29540995 | 1,977E-17 | 7,565E-15 |
| NANOZOOG7240 | 127,611873 | -2,4221049 | 0,43853283 | 3,5375E-09 | 2,1782E-07 |
| NANOZOOG5028 | 11,7035219 | 2,42205544 | 1,02938673 | 0,00468656 | 0,03692047 |
| NANOZOOG3771 | 9,24167644 | 2,41041766 | 1,27410333 | 0,00636014 | 0,04562802 |
| NANOZOOG4234 | 5,41756619 | -2,4101 | 1,02466772 | 0,00294132 | 0,02639306 |
| NANOZOOG2447 | 10,823816 | -2,4100475 | 0,58097592 | 5,5873E-06 | 0,00013793 |
| NANOZOOG3062 | 20,4673878 | -2,4093054 | 0,45577344 | 1,9039E-08 | 9,7134E-07 |
| NANOZOOG1014 | 15,6823519 | -2,4009251 | 0,65431718 | 2,5503E-05 | 0,00050228 |
| NANOZOOG6880 | 25,5543312 | 2,39029976 | 1,12390786 | 0,00329992 | 0,028838 |
| NANOZOOG9154 | 41,6005009 | -2,3896706 | 0,73780035 | 0,00011089 | 0,00183918 |
| NANOZOOG8643 | 15,7212462 | -2,3893647 | 0,57320586 | 3,7225E-06 | 9,6112E-05 |
| NANOZOOG1895 | 154,815101 | -2,3860249 | 0,42117296 | 1,5557E-09 | 1,0288E-07 |
| NANOZOOG61 | 10,7380493 | -2,3829475 | 1,02546084 | 0,00152728 | 0,01552496 |
| NANOZOOG1149 | 420,336594 | -2,3730736 | 0,38421884 | 6,8561E-11 | 5,9239E-09 |
| NANOZOOG5007 | 15,9697811 | 2,37084751 | 1,07247997 | 0,00621343 | 0,04510208 |
| NANOZOOG8333 | 32,1066768 | -2,3690065 | 0,37716128 | 4,5998E-11 | 4,1959E-09 |
| NANOZOOG542 | 12,8748462 | -2,3652741 | 0,5027763 | 5,1898E-07 | 1,8171E-05 |
| NANOZOOG1872 | 10,8556682 | -2,3640048 | 0,6604062 | 4,9634E-05 | 0,00091058 |
| NANOZOOG584 | 7,73347534 | -2,358594 | 0,9004506 | 0,00134129 | 0,01411647 |
| NANOZOOG2297 | 25,1954911 | 2,35732045 | 1,1174044 | 0,00636412 | 0,04562802 |
| NANOZOOG4086 | 6,84513299 | 2,35445126 | 1,09067897 | 0,00626982 | 0,04526607 |
| NANOZOOG9017 | 32,2806043 | -2,3543563 | 0,36273626 | 1,1876E-11 | 1,2235E-09 |
| NANOZOOG7736 | 7,7140137 | -2,3513255 | 0,6543319 | 6,301E-05 | 0,00112891 |
| NANOZOOG1519 | 16,9943121 | -2,3508838 | 0,5392606 | 1,8645E-06 | 5,2848E-05 |
| NANOZOOG8667 | 30,7308764 | -2,3463093 | 0,46037199 | 4,3745E-08 | 2,0556E-06 |
| NANOZOOG3114 | 7,50495214 | 2,34536466 | 1,27568928 | 0,00733341 | 0,04995894 |
| NANOZOOG4361 | 5,18947235 | -2,3390299 | 0,90725233 | 0,00095165 | 0,0108699 |
| NANOZOOG2860 | 14,3470338 | -2,335179 | 0,57921857 | 8,4794E-06 | 0,00020011 |
| NANOZOOG7117 | 17,4982969 | -2,332051 | 0,44806553 | 2,8687E-08 | 1,4099E-06 |
| NANOZOOG4298 | 26,3973025 | 2,32750246 | 0,601257 | 2,5935E-05 | 0,00050891 |
| NANOZOOG1377 | 10,2512016 | -2,3204857 | 0,68613357 | 0,00011795 | 0,00193823 |
| NANOZOOG9124 | 23,3447121 | -2,3111802 | 0,45409534 | 6,2845E-08 | 2,7595E-06 |
| NANOZOOG5092 | 14,5829782 | -2,3111654 | 0,60024978 | 1,4055E-05 | 0,00030238 |
| NANOZOOG407 | 11,9520836 | -2,3083838 | 0,50164079 | 7,9815E-07 | 2,5913E-05 |
| NANOZOOG6315 | 11,4534504 | 2,30606323 | 0,64984198 | 8,2279E-05 | 0,00142184 |
| NANOZOOG7967 | 4,97943119 | -2,3050225 | 0,88202816 | 0,00148917 | 0,01525272 |
| NANOZOOG6224 | 28,3655625 | -2,3033383 | 0,45363564 | 5,2355E-08 | 2,3372E-06 |
| NANOZOOG1991 | 20,5466405 | -2,3001812 | 0,56065518 | 4,7932E-06 | 0,00011943 |
| NANOZOOG8982 | 14,3149071 | 2,29902903 | 0,8069654 | 0,00071602 | 0,00854693 |
| NANOZOOG3041 | 9,77639713 | -2,2959305 | 0,74964991 | 0,00028051 | 0,00394412 |
| NANOZOOG6651 | 6,1184093 | -2,2847585 | 0,98779407 | 0,00337588 | 0,02921583 |
| NANOZOOG8439 | 6,23992571 | -2,2821488 | 0,85593047 | 0,00146669 | 0,01505185 |
| NANOZOOG6597 | 9,82082784 | -2,2807202 | 0,69152355 | 0,00014168 | 0,00225616 |
| NANOZOOG2148 | 30,3270722 | 2,27903997 | 0,89719585 | 0,00244439 | 0,02261593 |
| NANOZOOG8772 | 43,433399 | -2,2777067 | 0,32779005 | 5,4852E-13 | 7,1669E-11 |
| NANOZOOG2726 | 19,2943875 | -2,2755745 | 0,46029984 | 1,2267E-07 | 4,9784E-06 |
| NANOZOOG7411 | 4,72587782 | -2,2731765 | 1,05714431 | 0,00457376 | 0,03635254 |
| NANOZOOG5022 | 3,56226191 | -2,2726412 | 0,86478969 | 0,00097953 | 0,01107032 |
| NANOZOOG7276 | 19,5350545 | -2,270559 | 0,49780008 | 7,3791E-07 | 2,4401E-05 |
| NANOZOOG4733 | 12,7030084 | 2,26829902 | 0,85001836 | 0,00210605 | 0,02001741 |
| NANOZOOG2835 | 34,1463742 | 2,2673433 | 1,00097298 | 0,00235217 | 0,02195223 |
| NANOZOOG6667 | 6,97930741 | -2,2671908 | 0,84309938 | 0,0010036 | 0,01129476 |
| NANOZOOG5301 | 12,7098966 | 2,26675867 | 0,76303765 | 0,00059071 | 0,0073082 |
| NANOZOOG4155 | 6,04275426 | -2,2640282 | 0,75372372 | 0,00039922 | 0,00540053 |
| NANOZOOG6308 | 9,54202025 | -2,2632691 | 0,58877903 | 1,9997E-05 | 0,00040322 |
| NANOZOOG4291 | 7,9476483 | -2,2625314 | 0,74290672 | 0,00031289 | 0,00433121 |
| NANOZOOG7449 | 59,4936326 | -2,2595156 | 0,61029863 | 2,1557E-05 | 0,0004309 |
| NANOZOOG8516 | 8,48964468 | 2,25744931 | 1,06597293 | 0,00712624 | 0,04925841 |
| NANOZOOG3634 | 9,17524668 | -2,2557594 | 0,56700758 | 1,2159E-05 | 0,00026694 |
| NANOZOOG5541 | 39,396568 | -2,2552268 | 0,3455205 | 9,9768E-12 | 1,048E-09 |
| NANOZOOG7148 | 4,69916403 | -2,2521936 | 0,96166674 | 0,00334805 | 0,02908768 |
| NANOZOOG2045 | 10,1578296 | -2,2508651 | 0,57737256 | 1,5247E-05 | 0,00032157 |
| NANOZOOG8739 | 21,3710524 | -2,2506318 | 0,43897972 | 5,0304E-08 | 2,2837E-06 |
| NANOZOOG2610 | 6,53734727 | -2,2478799 | 0,99821018 | 0,00334217 | 0,02908768 |
| NANOZOOG30 | 28,0404458 | -2,2361632 | 0,34673247 | 1,8728E-11 | 1,8929E-09 |
| NANOZOOG1271 | 13,9944934 | -2,230802 | 0,45265886 | 1,3772E-07 | 5,5059E-06 |
| NANOZOOG7564 | 9,14080424 | -2,2299511 | 0,86634621 | 0,00117861 | 0,01285914 |
| NANOZOOG6886 | 11,6288179 | 2,22989145 | 0,88247932 | 0,00140291 | 0,01465632 |
| NANOZOOG2383 | 15,2610811 | -2,2288739 | 0,46017994 | 2,0882E-07 | 8,1062E-06 |
| NANOZOOG6311 | 4,37274483 | -2,2274423 | 0,81587282 | 0,00091848 | 0,01051341 |
| NANOZOOG3441 | 9,34312796 | 2,21874505 | 0,89223956 | 0,00301278 | 0,02680971 |
| NANOZOOG1732 | 7,47676535 | 2,21261142 | 1,12911591 | 0,00632405 | 0,04553489 |
| NANOZOOG8636 | 19,5225892 | -2,2078754 | 0,47418779 | 4,0732E-07 | 1,4843E-05 |
| NANOZOOG5261 | 9,30657193 | -2,2070481 | 0,77785234 | 0,00057954 | 0,00720328 |
| NANOZOOG7381 | 10,9054695 | -2,2066428 | 0,54278796 | 7,1803E-06 | 0,00017095 |
| NANOZOOG1182 | 24,2154792 | -2,2044461 | 0,40587373 | 6,7613E-09 | 3,8947E-07 |
| NANOZOOG3348 | 13,9991194 | 2,20264322 | 0,61502526 | 5,3382E-05 | 0,00096611 |
| NANOZOOG6874 | 31,584261 | 2,20161987 | 1,25491896 | 0,00508287 | 0,03934818 |
| NANOZOOG8218 | 7,91078152 | -2,1770569 | 0,63512551 | 0,00010201 | 0,001713 |
| NANOZOOG9248 | 35,7151791 | 2,17624231 | 0,60912015 | 8,279E-05 | 0,00142606 |
| NANOZOOG9071 | 17,0074573 | 2,17548741 | 0,86067714 | 0,00295662 | 0,02644176 |
| NANOZOOG7567 | 18,1959514 | -2,1740511 | 0,53478287 | 6,0166E-06 | 0,00014584 |
| NANOZOOG7812 | 19,028802 | -2,1718921 | 0,52668675 | 4,3036E-06 | 0,00010866 |
| NANOZOOG8823 | 16,2512312 | 2,17184194 | 0,66610315 | 0,00020908 | 0,00309409 |
| NANOZOOG8418 | 143,574957 | -2,1695203 | 0,42379431 | 3,5765E-08 | 1,7106E-06 |
| NANOZOOG5999 | 21,6560344 | -2,1672379 | 0,40514955 | 1,3332E-08 | 6,934E-07 |
| NANOZOOG6202 | 14,3806575 | -2,1620134 | 0,45332018 | 2,4054E-07 | 9,2041E-06 |
| NANOZOOG4188 | 16,3004164 | -2,1578946 | 0,65765837 | 0,00014573 | 0,00230962 |
| NANOZOOG4368 | 179,243697 | -2,1552167 | 0,49686879 | 1,6611E-06 | 4,7841E-05 |
| NANOZOOG1460 | 31,8335811 | -2,1533794 | 0,36052217 | 2,6215E-10 | 2,0353E-08 |
| NANOZOOG2324 | 8,13830373 | -2,1504061 | 0,65380988 | 0,00013882 | 0,00222652 |
| NANOZOOG639 | 8,71051667 | -2,1445435 | 0,55580467 | 1,6487E-05 | 0,00034501 |
| NANOZOOG3540 | 3,76018662 | -2,144223 | 0,99497775 | 0,00536816 | 0,04067503 |
| NANOZOOG6844 | 13,6118857 | -2,1413077 | 0,47630728 | 1,1106E-06 | 3,3998E-05 |
| NANOZOOG8101 | 32,699018 | -2,1401649 | 0,31410617 | 1,3643E-12 | 1,6996E-10 |
| NANOZOOG3444 | 16,0913381 | 2,13787562 | 0,9037886 | 0,00403362 | 0,03319219 |
| NANOZOOG474 | 15,4976493 | 2,13717416 | 0,63042131 | 0,00012178 | 0,00199501 |
| NANOZOOG9083 | 64,5352375 | 2,13685001 | 0,44518294 | 2,2702E-07 | 8,7493E-06 |
| NANOZOOG1378 | 28,4906706 | -2,1361056 | 0,47883665 | 1,0474E-06 | 3,2621E-05 |
| NANOZOOG5454 | 4,54837841 | -2,1344918 | 1,00939186 | 0,00564392 | 0,04199236 |
| NANOZOOG5924 | 50,7284989 | 2,12400818 | 0,58020356 | 5,8935E-05 | 0,00106301 |
| NANOZOOG4625 | 11,3826807 | -2,1237837 | 0,67669204 | 0,00016671 | 0,00258116 |
| NANOZOOG2872 | 14,724585 | -2,1189264 | 0,53481404 | 1,2717E-05 | 0,00027694 |
| NANOZOOG5177 | 8,31812342 | -2,1153739 | 0,71478279 | 0,00059486 | 0,00733013 |
| NANOZOOG2533 | 15,5562625 | -2,1140064 | 0,52350106 | 8,6409E-06 | 0,00020302 |
| NANOZOOG4779 | 54,0184453 | 2,11298859 | 0,49655011 | 3,2372E-06 | 8,585E-05 |
| NANOZOOG5923 | 86,5906967 | -2,1127055 | 0,50462072 | 3,2558E-06 | 8,5919E-05 |
| NANOZOOG678 | 24,5419671 | -2,1060674 | 0,43507503 | 1,9437E-07 | 7,6003E-06 |
| NANOZOOG260 | 7,13899142 | 2,10543465 | 0,90729395 | 0,00368804 | 0,03101543 |
| NANOZOOG1320 | 16,3360673 | -2,1052632 | 0,37906814 | 5,673E-09 | 3,3033E-07 |
| NANOZOOG1608 | 12,6410831 | -2,1031141 | 0,58090072 | 4,4922E-05 | 0,00083268 |
| NANOZOOG3284 | 36,9930721 | 2,10292282 | 0,52355782 | 1,2135E-05 | 0,00026694 |
| NANOZOOG5987 | 4,9233613 | -2,1017119 | 0,87316422 | 0,00188324 | 0,01846541 |
| NANOZOOG9353 | 416,265554 | -2,0973095 | 0,33433064 | 4,6212E-11 | 4,1959E-09 |
| NANOZOOG8906 | 6,11128581 | -2,066581 | 0,83342784 | 0,00206037 | 0,01970966 |
| NANOZOOG8104 | 6,19286135 | 2,06255746 | 0,99665899 | 0,00706626 | 0,0490432 |
| NANOZOOG3241 | 33,0702194 | -2,0606833 | 0,40245215 | 4,6602E-08 | 2,139E-06 |
| NANOZOOG2819 | 12,1463768 | -2,0590081 | 0,51043119 | 9,6237E-06 | 0,00022032 |
| NANOZOOG7725 | 29,1925028 | 2,05814413 | 0,51027803 | 1,1366E-05 | 0,00025371 |
| NANOZOOG7030 | 21,5246938 | -2,0569016 | 0,41030503 | 8,3517E-08 | 3,5792E-06 |
| NANOZOOG5683 | 28,6303938 | -2,0565022 | 0,45580356 | 9,3355E-07 | 2,9768E-05 |
| NANOZOOG7038 | 23,8969797 | -2,0560176 | 0,39695001 | 3,5331E-08 | 1,7051E-06 |
| NANOZOOG4038 | 13,7239334 | -2,0521084 | 0,66800816 | 0,00029816 | 0,00414871 |
| NANOZOOG6987 | 12,1107146 | -2,0515224 | 0,58348237 | 7,514E-05 | 0,00131544 |
| NANOZOOG2663 | 11,1773885 | -2,0412867 | 0,71108428 | 0,0005392 | 0,00686105 |
| NANOZOOG2305 | 27,6098171 | 2,03676167 | 0,80203915 | 0,00145581 | 0,01501419 |
| NANOZOOG4848 | 41,0205573 | -2,0299346 | 0,40633666 | 8,2437E-08 | 3,5614E-06 |
| NANOZOOG6227 | 14,4996003 | -2,0285034 | 0,50397199 | 1,0262E-05 | 0,00023394 |
| NANOZOOG704 | 6,92999393 | 2,02838161 | 0,95874909 | 0,00655643 | 0,04652025 |
| NANOZOOG6400 | 25,1299151 | -2,0263318 | 0,39711936 | 5,2024E-08 | 2,3372E-06 |
| NANOZOOG8281 | 39,7672653 | -2,0224762 | 0,35947198 | 2,7805E-09 | 1,7732E-07 |
| NANOZOOG189 | 13,1517645 | -2,0156891 | 0,96218486 | 0,00317082 | 0,02793764 |
| NANOZOOG5027 | 6,40972693 | -2,0148226 | 0,80705285 | 0,00199757 | 0,01936858 |
| NANOZOOG8096 | 40,085298 | -2,0132289 | 0,5212828 | 1,4576E-05 | 0,00031233 |
| NANOZOOG3334 | 8,49503103 | 2,00476353 | 0,90290687 | 0,00610968 | 0,04465148 |
| NANOZOOG6423 | 30,3310807 | -2,0025763 | 0,3550755 | 2,85E-09 | 1,7962E-07 |
| NANOZOOG7673 | 10,2636721 | -2,0015727 | 0,68694147 | 0,00044766 | 0,00590672 |
| NANOZOOG5845 | 30,8392388 | -1,9988838 | 0,35201812 | 2,4368E-09 | 1,5727E-07 |
| NANOZOOG3811 | 22,0128419 | -1,998612 | 0,5294235 | 2,3057E-05 | 0,00045747 |
| NANOZOOG6790 | 27,2594694 | -1,9982863 | 0,35933813 | 4,3137E-09 | 2,6259E-07 |
| NANOZOOG4377 | 27,8739357 | -1,996798 | 0,40201604 | 9,6267E-08 | 4,0606E-06 |
| NANOZOOG7236 | 10,7594988 | 1,99585467 | 0,65602257 | 0,00037783 | 0,0051371 |
| NANOZOOG4670 | 16,8289723 | 1,98740907 | 0,90856245 | 0,00596418 | 0,0438273 |
| NANOZOOG5838 | 4,99100354 | -1,9854188 | 0,7288168 | 0,00097202 | 0,01105546 |
| NANOZOOG996 | 5,44503087 | -1,9852372 | 0,82315102 | 0,00272285 | 0,02468071 |
| NANOZOOG5656 | 6,81342101 | -1,984822 | 0,66310698 | 0,00047917 | 0,00621529 |
| NANOZOOG314 | 8,93996536 | -1,9832532 | 0,89372271 | 0,00394922 | 0,03263642 |
| NANOZOOG972 | 8,80954723 | -1,9828644 | 0,61893371 | 0,00024861 | 0,00357054 |
| NANOZOOG41 | 15,1906121 | -1,9782809 | 0,48987126 | 8,0311E-06 | 0,00019037 |
| NANOZOOG2234 | 10,8163923 | 1,97819449 | 0,7893259 | 0,00286926 | 0,02578965 |
| NANOZOOG8244 | 8,54812763 | 1,97404922 | 0,76995389 | 0,00180407 | 0,01793028 |
| NANOZOOG7978 | 41,921181 | -1,9709449 | 0,34266251 | 1,4343E-09 | 9,6045E-08 |
| NANOZOOG8533 | 5,00449001 | -1,9571225 | 0,88263895 | 0,00445068 | 0,03563911 |
| NANOZOOG6772 | 42,0164785 | -1,9570668 | 0,46114493 | 2,9619E-06 | 7,9334E-05 |
| NANOZOOG7121 | 32,8478384 | -1,9485927 | 0,64467055 | 0,0003165 | 0,0043698 |
| NANOZOOG5604 | 22,514905 | -1,9458539 | 0,4521342 | 2,4979E-06 | 6,9334E-05 |
| NANOZOOG5508 | 13,4016071 | -1,9453589 | 0,53122846 | 4,2885E-05 | 0,00080047 |
| NANOZOOG1990 | 23,7330418 | -1,9436373 | 0,41414328 | 4,6979E-07 | 1,6667E-05 |
| NANOZOOG8749 | 33,3567475 | 1,94334973 | 0,67433544 | 0,00051634 | 0,00661728 |
| NANOZOOG7166 | 29,6826763 | -1,9412056 | 0,41401517 | 3,9028E-07 | 1,432E-05 |
| NANOZOOG3176 | 20,9260153 | -1,9406454 | 0,43282876 | 1,1819E-06 | 3,5744E-05 |
| NANOZOOG9169 | 28,5313041 | -1,9397708 | 0,43463981 | 1,224E-06 | 3,658E-05 |
| NANOZOOG4061 | 26,2855273 | -1,9390421 | 0,58768108 | 0,00012998 | 0,00211008 |
| NANOZOOG419 | 8,69027621 | -1,9387937 | 0,72170526 | 0,00127491 | 0,0135714 |
| NANOZOOG8734 | 9,05712619 | -1,9387326 | 0,69027372 | 0,00104864 | 0,01169942 |
| NANOZOOG2290 | 12,473745 | -1,9361202 | 0,53802551 | 5,0927E-05 | 0,00093112 |
| NANOZOOG2972 | 18,2977989 | -1,9355498 | 0,45046217 | 2,8018E-06 | 7,619E-05 |
| NANOZOOG4813 | 39,6228853 | -1,9343334 | 0,76353167 | 0,00141489 | 0,01471759 |
| NANOZOOG8291 | 21,9078428 | -1,9329044 | 0,48114598 | 8,8771E-06 | 0,00020586 |
| NANOZOOG6110 | 128,220148 | -1,9306905 | 0,47581708 | 6,6152E-06 | 0,00015891 |
| NANOZOOG3173 | 68,1794329 | 1,9305322 | 0,42036548 | 8,5911E-07 | 2,7725E-05 |
| NANOZOOG7214 | 2522,45957 | -1,9302856 | 0,21679678 | 8,5071E-20 | 5,0636E-17 |
| NANOZOOG7171 | 10,2032743 | 1,92967205 | 0,78742915 | 0,00335021 | 0,02908768 |
| NANOZOOG2247 | 3282,50365 | -1,9290299 | 0,35838816 | 1,1955E-08 | 6,4042E-07 |
| NANOZOOG5283 | 22,9951546 | 1,92789231 | 0,56304883 | 0,00010866 | 0,00180776 |
| NANOZOOG497 | 82,8808621 | -1,926562 | 0,3878528 | 1,0131E-07 | 4,2401E-06 |
| NANOZOOG3327 | 107,000692 | -1,9252429 | 0,30353953 | 3,3248E-11 | 3,1805E-09 |
| NANOZOOG7893 | 30,5064173 | -1,9245491 | 0,42964898 | 1,236E-06 | 3,658E-05 |
| NANOZOOG8721 | 81,8155264 | -1,9238445 | 0,66805653 | 0,00045065 | 0,00593159 |
| NANOZOOG8412 | 5,51683701 | -1,9192697 | 0,87087025 | 0,00512526 | 0,03958072 |
| NANOZOOG7756 | 9,209962 | -1,9162916 | 0,60570516 | 0,00026213 | 0,00372656 |
| NANOZOOG2661 | 4,89798048 | -1,9112085 | 0,92650031 | 0,00548392 | 0,0410872 |
| NANOZOOG6137 | 5,61077259 | -1,910594 | 0,71305422 | 0,00109337 | 0,01207667 |
| NANOZOOG1570 | 27,6216752 | -1,9017409 | 0,32577001 | 8,6788E-10 | 6,0379E-08 |
| NANOZOOG738 | 41,0503112 | -1,897387 | 0,35511458 | 1,3268E-08 | 6,934E-07 |
| NANOZOOG2269 | 5,2723466 | -1,895639 | 0,74043801 | 0,00166307 | 0,01671495 |
| NANOZOOG7992 | 8,63867948 | -1,8941873 | 0,70802667 | 0,00133023 | 0,01405532 |
| NANOZOOG6069 | 10,6274481 | 1,89413003 | 0,73652229 | 0,00224819 | 0,02104297 |
| NANOZOOG6803 | 9,42774652 | -1,8937365 | 0,71456697 | 0,00108978 | 0,01206189 |
| NANOZOOG3437 | 6,82919287 | -1,8927977 | 0,73613112 | 0,00176422 | 0,01759947 |
| NANOZOOG5726 | 18,2155609 | -1,8915153 | 0,53754894 | 7,2249E-05 | 0,00127316 |
| NANOZOOG8335 | 6,01591881 | -1,8806857 | 0,78544297 | 0,00272256 | 0,02468071 |
| NANOZOOG8839 | 10,5692042 | -1,8787562 | 0,56782965 | 0,00017821 | 0,00271984 |
| NANOZOOG3392 | 17,866845 | -1,8787054 | 0,43389209 | 2,6872E-06 | 7,3446E-05 |
| NANOZOOG6600 | 17,6465462 | -1,8773049 | 0,44069825 | 3,7318E-06 | 9,6112E-05 |
| NANOZOOG8147 | 16,6463734 | 1,87399377 | 0,74886926 | 0,00264429 | 0,02417313 |
| NANOZOOG4089 | 20,8383215 | -1,8690002 | 0,50410789 | 4,2048E-05 | 0,00078759 |
| NANOZOOG3703 | 12,6229702 | 1,86794095 | 0,85449428 | 0,00420513 | 0,03424827 |
| NANOZOOG9235 | 7,86131647 | 1,86780426 | 0,79619958 | 0,003158 | 0,02787056 |
| NANOZOOG7015 | 12,0850088 | -1,8677823 | 0,62678934 | 0,00043323 | 0,00574462 |
| NANOZOOG6271 | 7,76217492 | 1,86620632 | 0,85454576 | 0,00573631 | 0,04262054 |
| NANOZOOG2869 | 19,3434112 | 1,86392206 | 0,60346933 | 0,00045933 | 0,00603101 |
| NANOZOOG6206 | 5,14474379 | -1,8573673 | 0,93325193 | 0,00721054 | 0,04952164 |
| NANOZOOG888 | 81,634109 | 1,85698926 | 0,79588845 | 0,00326394 | 0,02857012 |
| NANOZOOG585 | 11,0971319 | -1,8557218 | 0,64221149 | 0,00064551 | 0,00785906 |
| NANOZOOG5711 | 14,260105 | -1,8548763 | 0,45915957 | 1,2338E-05 | 0,00026978 |
| NANOZOOG7231 | 8,83930738 | 1,85267099 | 0,86013626 | 0,00657327 | 0,04654766 |
| NANOZOOG8934 | 24,8239014 | -1,8510668 | 0,39097498 | 4,1298E-07 | 1,4948E-05 |
| NANOZOOG4306 | 22,4435291 | -1,8493332 | 0,3982898 | 6,4736E-07 | 2,1949E-05 |
| NANOZOOG6506 | 21,0966104 | -1,8488002 | 0,95033077 | 0,00578115 | 0,04286098 |
| NANOZOOG1080 | 42,8041903 | -1,8483102 | 0,27078134 | 1,6979E-12 | 2,0213E-10 |
| NANOZOOG7217 | 6717,66455 | -1,8461475 | 0,2276837 | 8,5616E-17 | 2,8665E-14 |
| NANOZOOG7743 | 40,8630416 | -1,8382164 | 0,47236886 | 1,519E-05 | 0,00032157 |
| NANOZOOG9148 | 34,4266517 | -1,8382035 | 0,4078602 | 1,1877E-06 | 3,5744E-05 |
| NANOZOOG4491 | 6,8298273 | -1,8340797 | 0,78340727 | 0,00404911 | 0,03321761 |
| NANOZOOG6135 | 13,7776098 | -1,8325677 | 0,48835583 | 3,132E-05 | 0,00060353 |
| NANOZOOG4281 | 15,3802095 | 1,82913489 | 0,77528015 | 0,00449749 | 0,03586818 |
| NANOZOOG9420 | 6,54697443 | -1,8284594 | 0,88284055 | 0,0071972 | 0,04949349 |
| NANOZOOG4272 | 37,3738042 | 1,82541539 | 0,72052502 | 0,0018105 | 0,01793609 |
| NANOZOOG4154 | 20,0602045 | -1,8244879 | 0,37884227 | 3,3921E-07 | 1,2532E-05 |
| NANOZOOG231 | 61,3815248 | -1,8238559 | 0,29587068 | 1,1905E-10 | 9,6631E-09 |
| NANOZOOG1785 | 17,940385 | -1,8230818 | 0,56911291 | 0,00019518 | 0,00292067 |
| NANOZOOG8687 | 15,1700787 | 1,82072569 | 0,68858219 | 0,00181908 | 0,0179794 |
| NANOZOOG1264 | 27,8543982 | -1,8185649 | 0,43236896 | 4,4577E-06 | 0,00011159 |
| NANOZOOG8188 | 556,979637 | -1,8171334 | 0,33449582 | 9,5349E-09 | 5,2658E-07 |
| NANOZOOG4134 | 39,6497043 | 1,81470853 | 0,51390336 | 7,1586E-05 | 0,00126563 |
| NANOZOOG6730 | 15,0650947 | 1,81250031 | 0,84139656 | 0,00492976 | 0,03832905 |
| NANOZOOG8369 | 40,8808978 | 1,80964507 | 0,5703281 | 0,00026183 | 0,00372656 |
| NANOZOOG1495 | 30,5766311 | -1,8094044 | 0,34947305 | 4,6716E-08 | 2,139E-06 |
| NANOZOOG7904 | 1697,36501 | -1,8078651 | 0,29749973 | 2,1349E-10 | 1,707E-08 |
| NANOZOOG1566 | 74,9339159 | -1,8046248 | 0,60844926 | 0,00041419 | 0,00557007 |
| NANOZOOG1612 | 12,2079046 | 1,80266508 | 0,72811963 | 0,00283714 | 0,0255438 |
| NANOZOOG4838 | 160,612682 | -1,8007577 | 0,57084366 | 0,00023878 | 0,00347592 |
| NANOZOOG3376 | 11,8799176 | 1,79933094 | 0,63887034 | 0,00112308 | 0,01232854 |
| NANOZOOG2089 | 29,938812 | 1,79889177 | 0,59574577 | 0,0005297 | 0,00677227 |
| NANOZOOG8767 | 25,3503334 | 1,79816864 | 0,55531295 | 0,00026525 | 0,00375913 |
| NANOZOOG3281 | 22,2129465 | -1,7864868 | 0,48012083 | 3,9918E-05 | 0,00075032 |
| NANOZOOG6056 | 14,6358101 | 1,78425768 | 0,79143637 | 0,00464946 | 0,03668215 |
| NANOZOOG7119 | 17,901421 | -1,7829617 | 0,87928552 | 0,00544944 | 0,04097368 |
| NANOZOOG8407 | 11,9997841 | -1,7814451 | 0,52809633 | 0,00012592 | 0,00205649 |
| NANOZOOG3047 | 10,4517908 | 1,77548844 | 0,72122809 | 0,00273864 | 0,0247731 |
| NANOZOOG3067 | 11,6119017 | -1,7722986 | 0,59987517 | 0,0005614 | 0,00709294 |
| NANOZOOG5504 | 5,62018357 | -1,7654597 | 0,77817489 | 0,00440727 | 0,03550337 |
| NANOZOOG3158 | 8,86379525 | -1,7642217 | 0,6565319 | 0,0014468 | 0,01496234 |
| NANOZOOG653 | 129,104847 | 1,76386494 | 0,75839164 | 0,00254559 | 0,02339064 |
| NANOZOOG8339 | 14,0287731 | -1,7596845 | 0,45366868 | 2,0022E-05 | 0,00040322 |
| NANOZOOG3499 | 15,5742528 | 1,75884404 | 0,6850447 | 0,00205032 | 0,01968382 |
| NANOZOOG9202 | 95,8964907 | 1,75605105 | 0,39015523 | 1,1703E-06 | 3,562E-05 |
| NANOZOOG8933 | 22,1465828 | -1,755669 | 0,45138552 | 1,6927E-05 | 0,00035283 |
| NANOZOOG3127 | 5,81033838 | -1,7511805 | 0,87881585 | 0,00727283 | 0,04975801 |
| NANOZOOG9414 | 11,3932484 | 1,75042141 | 0,87448138 | 0,00731101 | 0,0498918 |
| NANOZOOG3544 | 13,2784558 | -1,7481734 | 0,54080648 | 0,00021743 | 0,00320874 |
| NANOZOOG3573 | 17,2280131 | 1,74761556 | 0,75698419 | 0,00356672 | 0,03050566 |
| NANOZOOG2514 | 94,1041735 | -1,7462807 | 0,36707262 | 3,3151E-07 | 1,2419E-05 |
| NANOZOOG2077 | 9,93847516 | -1,7462793 | 0,73781233 | 0,00274229 | 0,0247731 |
| NANOZOOG5522 | 29,8190569 | -1,7394024 | 0,31346465 | 5,2332E-09 | 3,1499E-07 |
| NANOZOOG6794 | 96,4683969 | -1,7391896 | 0,52039386 | 0,00012764 | 0,00207838 |
| NANOZOOG897 | 15,7356212 | 1,73065437 | 0,58639156 | 0,00085105 | 0,00986815 |
| NANOZOOG1809 | 10,2701722 | -1,7273654 | 0,60031524 | 0,00079658 | 0,00935801 |
| NANOZOOG1933 | 35,4749831 | 1,72722682 | 0,50952681 | 0,000161 | 0,0025145 |
| NANOZOOG7619 | 10,7206614 | -1,7217735 | 0,5568943 | 0,00046631 | 0,00607789 |
| NANOZOOG8484 | 58,6979507 | -1,718612 | 0,28299697 | 2,4083E-10 | 1,8972E-08 |
| NANOZOOG4873 | 17,5527432 | 1,71643178 | 0,63489565 | 0,00133778 | 0,01410729 |
| NANOZOOG6128 | 17,4834849 | -1,7157235 | 0,42407366 | 1,0584E-05 | 0,0002389 |
| NANOZOOG867 | 8,68952028 | -1,7123915 | 0,63920558 | 0,00137197 | 0,01441105 |
| NANOZOOG3388 | 10,4063587 | -1,7093898 | 0,51954372 | 0,00021974 | 0,00322512 |
| NANOZOOG3932 | 5,22812259 | -1,7087835 | 0,82873796 | 0,00598709 | 0,04393538 |
| NANOZOOG2816 | 8,79704459 | -1,7078996 | 0,79266509 | 0,00413602 | 0,03382697 |
| NANOZOOG6860 | 28,4555031 | 1,70588788 | 0,63769915 | 0,00123624 | 0,01329828 |
| NANOZOOG3227 | 20,9403826 | -1,704358 | 0,49026036 | 8,396E-05 | 0,00144158 |
| NANOZOOG2556 | 35,2035694 | -1,7039171 | 0,37985068 | 1,4207E-06 | 4,1588E-05 |
| NANOZOOG3229 | 8,92015241 | -1,7037267 | 0,66253208 | 0,00175395 | 0,01752969 |
| NANOZOOG199 | 14,7988756 | -1,7036914 | 0,53974184 | 0,00028387 | 0,00398092 |
| NANOZOOG6298 | 30,0925843 | -1,7027664 | 0,33244839 | 5,7929E-08 | 2,5647E-06 |
| NANOZOOG6241 | 25,0388457 | -1,6983499 | 0,486742 | 9,3739E-05 | 0,00159923 |
| NANOZOOG6449 | 26,5563875 | 1,69771716 | 0,73078934 | 0,00481575 | 0,03760638 |
| NANOZOOG4152 | 19,5058208 | -1,6966851 | 0,62693442 | 0,00122892 | 0,0132799 |
| NANOZOOG7488 | 8,24318984 | -1,6937609 | 0,65851451 | 0,00205574 | 0,01970055 |
| NANOZOOG5798 | 17,1122699 | -1,6936574 | 0,75637878 | 0,00359773 | 0,03056549 |
| NANOZOOG7070 | 26,9278197 | 1,69361363 | 0,59501574 | 0,00108098 | 0,01198926 |
| NANOZOOG8459 | 7,79578632 | -1,6908615 | 0,58168758 | 0,00075811 | 0,00898497 |
| NANOZOOG7560 | 61,3881227 | -1,6898105 | 0,38983026 | 2,528E-06 | 6,9806E-05 |
| NANOZOOG3141 | 33,1889291 | -1,6885152 | 0,75816383 | 0,00361172 | 0,03056549 |
| NANOZOOG8551 | 12,0196233 | 1,68623838 | 0,79006263 | 0,00558477 | 0,04161002 |
| NANOZOOG2406 | 20,2775287 | -1,6852429 | 0,51121806 | 0,00016752 | 0,00258621 |
| NANOZOOG8419 | 23,8777227 | -1,6823514 | 0,85174165 | 0,00650866 | 0,04636453 |
| NANOZOOG7168 | 6,11641181 | -1,6801989 | 0,72987745 | 0,00364641 | 0,03071353 |
| NANOZOOG340 | 10,9411601 | -1,6756786 | 0,81383581 | 0,00539223 | 0,04074211 |
| NANOZOOG5959 | 10,7815363 | -1,673901 | 0,66046596 | 0,00217459 | 0,02047326 |
| NANOZOOG6309 | 10,6120411 | -1,6662821 | 0,58396433 | 0,00078778 | 0,00927506 |
| NANOZOOG5824 | 11,2873541 | -1,6648887 | 0,56674713 | 0,00058487 | 0,00725267 |
| NANOZOOG6354 | 697,014764 | -1,6643701 | 0,28190409 | 6,7804E-10 | 4,7793E-08 |
| NANOZOOG5131 | 17,6186263 | 1,64643804 | 0,77420816 | 0,00545418 | 0,04097368 |
| NANOZOOG1220 | 952,831026 | -1,6424682 | 0,42833342 | 2,4108E-05 | 0,00047656 |
| NANOZOOG4354 | 13,1724708 | -1,6295555 | 0,46854533 | 0,00011255 | 0,00186095 |
| NANOZOOG2035 | 38,3197079 | -1,6291617 | 0,38338121 | 4,2365E-06 | 0,00010756 |
| NANOZOOG1071 | 67,4776107 | -1,6251889 | 0,32780378 | 1,391E-07 | 5,5199E-06 |
| NANOZOOG8987 | 7,12209856 | -1,6251097 | 0,68471669 | 0,00364014 | 0,03070903 |
| NANOZOOG5277 | 18,4399035 | 1,6238372 | 0,61923998 | 0,00201025 | 0,01936858 |
| NANOZOOG2242 | 8,0054592 | -1,623728 | 0,5689366 | 0,0009046 | 0,01039902 |
| NANOZOOG7566 | 8,35098806 | 1,62318826 | 0,75167612 | 0,0054556 | 0,04097368 |
| NANOZOOG7462 | 13,0534301 | -1,622493 | 0,42211674 | 2,7194E-05 | 0,00052974 |
| NANOZOOG3214 | 7,49075519 | -1,6204653 | 0,7108119 | 0,00532303 | 0,04056251 |
| NANOZOOG5339 | 1084,53264 | -1,6158237 | 0,35432231 | 1,0146E-06 | 3,1785E-05 |
| NANOZOOG7793 | 15,2521778 | 1,61174206 | 0,57140468 | 0,00121398 | 0,0131646 |
| NANOZOOG4969 | 23,5299895 | 1,61054976 | 0,52005921 | 0,0004694 | 0,00610334 |
| NANOZOOG5625 | 5,8822461 | -1,6103133 | 0,65731864 | 0,00269643 | 0,02456594 |
| NANOZOOG2002 | 10,8453915 | -1,6039291 | 0,68464132 | 0,00413008 | 0,03382697 |
| NANOZOOG4964 | 13,3607597 | 1,59871805 | 0,60531303 | 0,00213067 | 0,02015776 |
| NANOZOOG6773 | 104,535517 | -1,5961333 | 0,34402882 | 6,7463E-07 | 2,2587E-05 |
| NANOZOOG8814 | 18,9604719 | 1,59503645 | 0,72625566 | 0,00472078 | 0,03708094 |
| NANOZOOG7885 | 20,5649146 | -1,5946603 | 0,64138521 | 0,00213355 | 0,02015776 |
| NANOZOOG9406 | 37,3888683 | -1,5943964 | 0,43799428 | 4,9468E-05 | 0,00091058 |
| NANOZOOG2058 | 40,4164751 | -1,5920751 | 0,54083523 | 0,00065847 | 0,00796373 |
| NANOZOOG7220 | 3551,93305 | -1,5865068 | 0,26156349 | 2,7632E-10 | 2,1146E-08 |
| NANOZOOG328 | 23,0091935 | -1,5858218 | 0,35935327 | 2,3464E-06 | 6,5467E-05 |
| NANOZOOG1498 | 18,0751642 | -1,5839361 | 0,48259823 | 0,00024827 | 0,00357054 |
| NANOZOOG8159 | 12,4164237 | 1,58152751 | 0,64394593 | 0,00346083 | 0,02980658 |
| NANOZOOG9428 | 44,7839882 | -1,5802843 | 0,2885632 | 8,4034E-09 | 4,7837E-07 |
| NANOZOOG3269 | 9,05470187 | 1,57262865 | 0,71882743 | 0,00652412 | 0,04636453 |
| NANOZOOG1808 | 173,562425 | -1,5699018 | 0,29300732 | 1,8684E-08 | 9,6241E-07 |
| NANOZOOG4043 | 42,7746408 | 1,5688519 | 0,47231081 | 0,00019058 | 0,00285972 |
| NANOZOOG7290 | 17,8595793 | -1,5644672 | 0,46664942 | 0,00018046 | 0,00274211 |
| NANOZOOG5140 | 211,804977 | -1,5637493 | 0,45278387 | 0,00010628 | 0,00177362 |
| NANOZOOG8535 | 91,850603 | -1,562744 | 0,31128228 | 1,1213E-07 | 4,5852E-06 |
| NANOZOOG8872 | 21,5257687 | 1,56177723 | 0,53295802 | 0,00071637 | 0,00854693 |
| NANOZOOG8356 | 15,4036336 | -1,5571095 | 0,53095482 | 0,00083877 | 0,00976803 |
| NANOZOOG8743 | 39,2513031 | -1,5539701 | 0,46414548 | 0,00015771 | 0,00247751 |
| NANOZOOG1795 | 11,2887092 | -1,5515498 | 0,61421124 | 0,00237083 | 0,02202311 |
| NANOZOOG8317 | 17,5067277 | 1,55093944 | 0,59977303 | 0,00223532 | 0,02097134 |
| NANOZOOG7223 | 25,938494 | -1,5475272 | 0,48726377 | 0,00029593 | 0,00412843 |
| NANOZOOG74 | 19,4526118 | -1,5411184 | 0,53183704 | 0,00074512 | 0,0088506 |
| NANOZOOG8709 | 8,46521919 | -1,5395477 | 0,66431302 | 0,0036989 | 0,03105122 |
| NANOZOOG667 | 45,2717667 | -1,5370573 | 0,29357189 | 3,0758E-08 | 1,4979E-06 |
| NANOZOOG7965 | 17,8892651 | -1,5352798 | 0,51758996 | 0,00065224 | 0,007923 |
| NANOZOOG2542 | 9,46642461 | 1,53283296 | 0,70673142 | 0,00726209 | 0,04975801 |
| NANOZOOG4537 | 17,2333276 | -1,5318846 | 0,58711373 | 0,00162159 | 0,01635943 |
| NANOZOOG3908 | 17,0895579 | 1,53025823 | 0,45187123 | 0,00018069 | 0,00274211 |
| NANOZOOG9001 | 14,8622587 | -1,528663 | 0,50322176 | 0,0005488 | 0,00696665 |
| NANOZOOG7068 | 9,3243468 | -1,5285672 | 0,72255549 | 0,00643435 | 0,04595842 |
| NANOZOOG1702 | 22,7420905 | 1,52175032 | 0,49714921 | 0,00060282 | 0,0074067 |
| NANOZOOG2591 | 10,3288342 | -1,5217431 | 0,72701179 | 0,00652583 | 0,04636453 |
| NANOZOOG4003 | 53,484035 | 1,52091086 | 0,42806292 | 0,00010329 | 0,0017291 |
| NANOZOOG3325 | 14,7071672 | -1,5193605 | 0,47807215 | 0,00035213 | 0,00482443 |
| NANOZOOG4856 | 10,1735512 | -1,5183902 | 0,49198499 | 0,00048654 | 0,00626531 |
| NANOZOOG3280 | 11,6176433 | -1,5129862 | 0,54784181 | 0,0014147 | 0,01471759 |
| NANOZOOG783 | 23,9250202 | -1,5128262 | 0,67203983 | 0,00418973 | 0,03421396 |
| NANOZOOG7044 | 19,3313513 | -1,5121852 | 0,44684249 | 0,00015962 | 0,00250023 |
| NANOZOOG5583 | 24,0544357 | -1,5117898 | 0,35714075 | 5,7375E-06 | 0,00014099 |
| NANOZOOG4270 | 10,1405501 | 1,51176857 | 0,64596519 | 0,00473227 | 0,03711676 |
| NANOZOOG2335 | 19,2077367 | 1,51076176 | 0,53221781 | 0,00116576 | 0,01274482 |
| NANOZOOG4066 | 46,0855194 | 1,50909952 | 0,38115404 | 1,9034E-05 | 0,00038917 |
| NANOZOOG7687 | 12,6137499 | -1,5087441 | 0,55517169 | 0,00146022 | 0,01501419 |
| NANOZOOG6661 | 5,61793969 | -1,50612 | 0,68754878 | 0,00642504 | 0,04595322 |
| NANOZOOG1095 | 7,70344296 | -1,5052498 | 0,62250513 | 0,00341638 | 0,02951863 |
| NANOZOOG1603 | 27,6085539 | -1,5030177 | 0,32771851 | 1,0636E-06 | 3,2936E-05 |
| NANOZOOG5856 | 12,5335393 | -1,5025378 | 0,57054375 | 0,00189758 | 0,01851612 |
| NANOZOOG6693 | 472,154199 | -1,5023199 | 0,50059905 | 0,00057151 | 0,00713655 |
| NANOZOOG131 | 36,2054342 | -1,5008286 | 0,3280676 | 1,1028E-06 | 3,3952E-05 |
| NANOZOOG7123 | 157,869722 | -1,5001894 | 0,52603506 | 0,00084487 | 0,00981777 |
| NANOZOOG7614 | 17,4472351 | 1,50012036 | 0,57535534 | 0,00200791 | 0,01936858 |
| NANOZOOG3520 | 12,3635957 | -1,4962425 | 0,56124931 | 0,00181135 | 0,01793609 |
| NANOZOOG7014 | 11,9653541 | -1,4960335 | 0,66668291 | 0,00449361 | 0,03586818 |
| NANOZOOG5527 | 99,2323211 | -1,4945167 | 0,31403363 | 4,2647E-07 | 1,5333E-05 |
| NANOZOOG9099 | 10,1439239 | -1,4882348 | 0,59093589 | 0,0027887 | 0,02514998 |
| NANOZOOG568 | 412,600457 | -1,4876742 | 0,27867179 | 2,1921E-08 | 1,0975E-06 |
| NANOZOOG792 | 11,5575396 | -1,4855923 | 0,53942344 | 0,00140353 | 0,01465632 |
| NANOZOOG3819 | 28,2253116 | 1,48205944 | 0,4701155 | 0,00043064 | 0,00572441 |
| NANOZOOG2009 | 8,96555094 | -1,4819209 | 0,56454701 | 0,00185429 | 0,01826 |
| NANOZOOG6923 | 10,0371674 | 1,47720496 | 0,65581112 | 0,00630967 | 0,04549249 |
| NANOZOOG55 | 13,3103671 | -1,4769892 | 0,47053236 | 0,00041487 | 0,00557007 |
| NANOZOOG3671 | 20,2388803 | 1,47690215 | 0,55172912 | 0,00195351 | 0,01902722 |
| NANOZOOG7605 | 20,8236264 | 1,47590926 | 0,70899272 | 0,00719357 | 0,04949349 |
| NANOZOOG4831 | 15,2872271 | 1,47416565 | 0,63478202 | 0,00521201 | 0,03988681 |
| NANOZOOG4977 | 9,47251228 | -1,4717788 | 0,61902323 | 0,00401456 | 0,03308615 |
| NANOZOOG7482 | 19,8094844 | -1,4623791 | 0,40618103 | 7,7396E-05 | 0,00134614 |
| NANOZOOG2181 | 8,94846455 | -1,4590857 | 0,61590415 | 0,00420671 | 0,03424827 |
| NANOZOOG3028 | 17,1841346 | -1,4589549 | 0,44508444 | 0,00023847 | 0,00347592 |
| NANOZOOG299 | 21,1897823 | -1,4581955 | 0,3645823 | 1,7038E-05 | 0,00035377 |
| NANOZOOG3113 | 7,62300205 | -1,4536584 | 0,69754587 | 0,00681659 | 0,04792187 |
| NANOZOOG1695 | 19,5958836 | -1,4533371 | 0,44208997 | 0,00027474 | 0,00387307 |
| NANOZOOG2031 | 5,64221924 | -1,4529733 | 0,66606327 | 0,00657767 | 0,04654766 |
| NANOZOOG6723 | 12,0472718 | -1,452965 | 0,62786847 | 0,00404207 | 0,03321069 |
| NANOZOOG7909 | 10,1145384 | 1,45104185 | 0,65659868 | 0,00727136 | 0,04975801 |
| NANOZOOG606 | 24,1804678 | -1,4504875 | 0,5106456 | 0,00098566 | 0,01111612 |
| NANOZOOG6715 | 10,3600504 | 1,45041471 | 0,66556012 | 0,00729402 | 0,04983938 |
| NANOZOOG7096 | 19,7154562 | -1,4450224 | 0,46103633 | 0,00043886 | 0,00580487 |
| NANOZOOG9174 | 6,88462928 | -1,4449485 | 0,5779153 | 0,00305055 | 0,02706707 |
| NANOZOOG8347 | 12,939484 | -1,4430812 | 0,45729955 | 0,00038897 | 0,00527527 |
| NANOZOOG4485 | 38,5847573 | 1,44306388 | 0,49405089 | 0,00105048 | 0,01169942 |
| NANOZOOG6166 | 6,80032749 | -1,4394804 | 0,62248929 | 0,00458647 | 0,03639958 |
| NANOZOOG389 | 1754,182 | -1,432354 | 0,19193185 | 2,3799E-14 | 4,3998E-12 |
| NANOZOOG9329 | 10,7494404 | -1,4269621 | 0,60094616 | 0,00377344 | 0,03145691 |
| NANOZOOG1586 | 37,183595 | -1,4248525 | 0,64363971 | 0,0053633 | 0,04067503 |
| NANOZOOG7984 | 10,8594604 | -1,4247762 | 0,62581959 | 0,00586151 | 0,04331048 |
| NANOZOOG5947 | 24,2760321 | -1,4245631 | 0,38531315 | 5,156E-05 | 0,00093629 |
| NANOZOOG9085 | 41,6038065 | -1,4241864 | 0,3474946 | 9,2538E-06 | 0,00021368 |
| NANOZOOG4180 | 30,5618396 | -1,4234289 | 0,35278174 | 1,4748E-05 | 0,00031475 |
| NANOZOOG7078 | 8,43210382 | -1,416554 | 0,56343008 | 0,00263714 | 0,02414901 |
| NANOZOOG6000 | 181,06188 | -1,4060799 | 0,46457071 | 0,00057741 | 0,00719344 |
| NANOZOOG6428 | 33,3878016 | -1,40247 | 0,55243381 | 0,0023721 | 0,02202311 |
| NANOZOOG8603 | 22,4299428 | -1,3970795 | 0,42893809 | 0,00027371 | 0,00386872 |
| NANOZOOG8398 | 13,8016654 | -1,3948608 | 0,51448688 | 0,00159059 | 0,01607697 |
| NANOZOOG1188 | 12,5243284 | -1,3842089 | 0,53753997 | 0,00225082 | 0,02104297 |
| NANOZOOG803 | 36,1151097 | -1,375496 | 0,59275683 | 0,00436187 | 0,03524366 |
| NANOZOOG509 | 17,6501712 | 1,37271023 | 0,58909558 | 0,00557979 | 0,04161002 |
| NANOZOOG7353 | 22,206953 | -1,3649881 | 0,43220685 | 0,00042126 | 0,00562406 |
| NANOZOOG7226 | 5750,32271 | -1,3641054 | 0,21843242 | 1,1607E-10 | 9,5657E-09 |
| NANOZOOG3046 | 31,2916822 | -1,3640406 | 0,38566077 | 9,9077E-05 | 0,00166904 |
| NANOZOOG2117 | 11,0425799 | -1,3588613 | 0,5383841 | 0,00298068 | 0,02661254 |
| NANOZOOG6467 | 18,9985646 | -1,3573529 | 0,43965307 | 0,0004836 | 0,00624248 |
| NANOZOOG3822 | 10,2914716 | -1,3471601 | 0,50558478 | 0,00210749 | 0,02001741 |
| NANOZOOG498 | 478,853077 | -1,3465187 | 0,37713375 | 9,1614E-05 | 0,00156798 |
| NANOZOOG8669 | 28,8254612 | -1,346467 | 0,38833717 | 0,00013246 | 0,00213877 |
| NANOZOOG1224 | 37,7245723 | -1,3439857 | 0,33698371 | 1,7401E-05 | 0,00035854 |
| NANOZOOG3754 | 21,4665871 | 1,34249903 | 0,57068243 | 0,0052283 | 0,03993236 |
| NANOZOOG7298 | 11,7504044 | -1,3328168 | 0,47427066 | 0,00131523 | 0,01392425 |
| NANOZOOG4319 | 105,386085 | -1,3296342 | 0,3652125 | 7,4259E-05 | 0,00130428 |
| NANOZOOG8187 | 614,960848 | -1,3241181 | 0,34550922 | 3,4559E-05 | 0,0006612 |
| NANOZOOG5723 | 20,437289 | 1,31980601 | 0,50634785 | 0,00265602 | 0,024239 |
| NANOZOOG2736 | 14,5584839 | -1,3184981 | 0,53453201 | 0,00354709 | 0,0304515 |
| NANOZOOG2524 | 15,9766271 | -1,3080917 | 0,44938213 | 0,00097551 | 0,01105685 |
| NANOZOOG3211 | 21,8019284 | -1,3069982 | 0,38668835 | 0,00020835 | 0,00309178 |
| NANOZOOG3248 | 36,8813606 | 1,30298463 | 0,47731746 | 0,00186852 | 0,01836632 |
| NANOZOOG7065 | 41,5898197 | -1,301404 | 0,3029897 | 4,8773E-06 | 0,00012096 |
| NANOZOOG2577 | 10,762965 | -1,2913877 | 0,55809305 | 0,00521009 | 0,03988681 |
| NANOZOOG2162 | 10,9211327 | -1,2906598 | 0,47237296 | 0,00184344 | 0,01818658 |
| NANOZOOG8634 | 15,7716973 | -1,2906201 | 0,46511685 | 0,00163038 | 0,01641723 |
| NANOZOOG9380 | 14,1436182 | 1,28445181 | 0,56594429 | 0,00705974 | 0,0490432 |
| NANOZOOG8936 | 10,8899475 | -1,2803261 | 0,51553148 | 0,00370389 | 0,03105122 |
| NANOZOOG2906 | 21,0818128 | -1,2801619 | 0,46064595 | 0,00154475 | 0,01567278 |
| NANOZOOG1509 | 32,7813829 | -1,2745615 | 0,46663813 | 0,00171993 | 0,01725408 |
| NANOZOOG6108 | 187,461267 | -1,2715145 | 0,43460085 | 0,00089426 | 0,01030221 |
| NANOZOOG5186 | 23,8971687 | -1,2678404 | 0,3716369 | 0,00019052 | 0,00285972 |
| NANOZOOG3895 | 17,4709292 | -1,2673062 | 0,40512449 | 0,00057077 | 0,00713655 |
| NANOZOOG6641 | 589,742908 | -1,2663168 | 0,3060063 | 1,0614E-05 | 0,0002389 |
| NANOZOOG2953 | 17,2232804 | -1,2662798 | 0,42375075 | 0,00088746 | 0,0102459 |
| NANOZOOG6504 | 11,0367063 | -1,2641587 | 0,56588261 | 0,00673661 | 0,04754679 |
| NANOZOOG323 | 13,2893999 | -1,2609803 | 0,55210047 | 0,00622629 | 0,04513429 |
| NANOZOOG3920 | 16,9204746 | 1,2590758 | 0,53421376 | 0,00578467 | 0,04286098 |
| NANOZOOG99 | 20,5461316 | -1,252796 | 0,44317336 | 0,00149641 | 0,01526906 |
| NANOZOOG3452 | 22,0677997 | 1,24863307 | 0,4674437 | 0,00253823 | 0,02336304 |
| NANOZOOG7218 | 1279,94332 | -1,2453078 | 0,1939911 | 4,405E-11 | 4,1399E-09 |
| NANOZOOG3149 | 22,3078066 | -1,2378524 | 0,48818826 | 0,0033672 | 0,02918782 |
| NANOZOOG2685 | 13,9770084 | 1,23725476 | 0,54673577 | 0,00718035 | 0,04949349 |
| NANOZOOG6728 | 10,8129728 | -1,23229 | 0,52462407 | 0,00520943 | 0,03988681 |
| NANOZOOG2503 | 29,3830145 | 1,23201205 | 0,53800652 | 0,00714317 | 0,04931179 |
| NANOZOOG8243 | 63,5639123 | 1,23189676 | 0,33284621 | 6,9083E-05 | 0,00122543 |
| NANOZOOG2534 | 11,8949264 | -1,2301698 | 0,54969279 | 0,00677706 | 0,04773613 |
| NANOZOOG1468 | 33,8904056 | 1,22778387 | 0,42049811 | 0,00127277 | 0,0135714 |
| NANOZOOG3592 | 28,0029299 | -1,2174312 | 0,32907244 | 6,785E-05 | 0,00120756 |
| NANOZOOG4034 | 25,0063972 | 1,20397059 | 0,48420293 | 0,00445073 | 0,03563911 |
| NANOZOOG8654 | 11,0243472 | -1,1980873 | 0,50012073 | 0,00533779 | 0,04061727 |
| NANOZOOG8109 | 14,7780065 | -1,197783 | 0,49999356 | 0,00523288 | 0,03993236 |
| NANOZOOG4645 | 39,3667043 | -1,1925361 | 0,28952853 | 1,3298E-05 | 0,00028841 |
| NANOZOOG5278 | 10,2701675 | -1,1835381 | 0,50456876 | 0,00620953 | 0,04510208 |
| NANOZOOG5544 | 19,8172307 | -1,1829278 | 0,46831675 | 0,00374665 | 0,03136011 |
| NANOZOOG5378 | 20,9608867 | -1,1713399 | 0,39493853 | 0,00101294 | 0,01137597 |
| NANOZOOG1950 | 32,5731134 | 1,17032476 | 0,39817503 | 0,00122979 | 0,0132799 |
| NANOZOOG1171 | 14,1333079 | -1,1625568 | 0,45675007 | 0,00357048 | 0,03050566 |
| NANOZOOG3773 | 25,4016655 | 1,15492086 | 0,43222405 | 0,00261796 | 0,02401443 |
| NANOZOOG3531 | 19,07399 | -1,1530379 | 0,43236431 | 0,00271082 | 0,02465513 |
| NANOZOOG4559 | 22,4697759 | 1,14795438 | 0,43651314 | 0,00295629 | 0,02644176 |
| NANOZOOG7222 | 20001,2538 | -1,1454007 | 0,15322855 | 2,8583E-14 | 4,9394E-12 |
| NANOZOOG8737 | 20,1857249 | -1,1421602 | 0,4691487 | 0,00489529 | 0,03811639 |
| NANOZOOG9067 | 25,5302232 | -1,138941 | 0,35898136 | 0,00055891 | 0,00707824 |
| NANOZOOG3995 | 12,7690948 | -1,137565 | 0,47776526 | 0,00587695 | 0,04336479 |
| NANOZOOG4973 | 32,2866847 | -1,1299267 | 0,3836484 | 0,00120916 | 0,01313889 |
| NANOZOOG2229 | 72,6663268 | 1,12335032 | 0,33595699 | 0,00031075 | 0,00431261 |
| NANOZOOG8610 | 15,5815947 | 1,12260021 | 0,46666784 | 0,00603804 | 0,04418822 |
| NANOZOOG7219 | 250825,212 | -1,1217626 | 0,20085037 | 8,7001E-09 | 4,8548E-07 |
| NANOZOOG9274 | 23,8387545 | -1,1188345 | 0,48381728 | 0,00691011 | 0,04838883 |
| NANOZOOG8674 | 26,5356347 | -1,1135962 | 0,3531777 | 0,00059522 | 0,00733013 |
| NANOZOOG7932 | 18,471066 | -1,0870835 | 0,43661227 | 0,00463189 | 0,03659736 |
| NANOZOOG7931 | 29,1579173 | -1,0810371 | 0,32586456 | 0,0003535 | 0,00483086 |
| NANOZOOG4098 | 24,7161536 | 1,07948916 | 0,44861308 | 0,00634221 | 0,04560435 |
| NANOZOOG8979 | 17,5696216 | -1,0679485 | 0,41342706 | 0,00377577 | 0,03145691 |
| NANOZOOG6762 | 386,406821 | -1,0661986 | 0,30882616 | 0,00020687 | 0,00307838 |
| NANOZOOG4885 | 709,774022 | -1,0615467 | 0,3020554 | 0,00017129 | 0,00262922 |
| NANOZOOG8653 | 18,4342099 | -1,0610562 | 0,41436638 | 0,00389854 | 0,03236372 |
| NANOZOOG9100 | 2011,99447 | 1,05925815 | 0,30429765 | 0,00019841 | 0,0029607 |
| NANOZOOG3010 | 286,595942 | -1,0547826 | 0,37849429 | 0,00200384 | 0,01936858 |
| NANOZOOG871 | 29,3835862 | 1,04589484 | 0,40688657 | 0,00431313 | 0,03495371 |
| NANOZOOG3727 | 14,268174 | -1,0430538 | 0,40616845 | 0,00381857 | 0,03176407 |
| NANOZOOG7301 | 40,0240404 | 1,03732514 | 0,40454469 | 0,0039539 | 0,03263642 |
| NANOZOOG2163 | 24,2316025 | -1,0336083 | 0,34228458 | 0,00102584 | 0,01149668 |
| NANOZOOG1138 | 36,1716457 | 1,01392178 | 0,3536037 | 0,00177487 | 0,0176728 |
| NANOZOOG8138 | 40,7971545 | 0,9984595 | 0,3630228 | 0,00250048 | 0,02309496 |
| NANOZOOG1794 | 19,0725323 | -0,9962815 | 0,38788673 | 0,00427387 | 0,03474222 |
| NANOZOOG5329 | 21,314081 | -0,9910554 | 0,41196206 | 0,00678126 | 0,04773613 |
| NANOZOOG8057 | 1143,09276 | -0,9807437 | 0,34771987 | 0,00200837 | 0,01936858 |
| NANOZOOG7238 | 24,9546703 | -0,968914 | 0,3644682 | 0,00333919 | 0,02908768 |
| NANOZOOG4005 | 47,0384078 | 0,95316768 | 0,36462196 | 0,00429051 | 0,03482466 |
| NANOZOOG801 | 15,1880003 | -0,9373075 | 0,37279526 | 0,00520051 | 0,03988681 |
| NANOZOOG3039 | 30,7026147 | -0,9217355 | 0,38134361 | 0,00709721 | 0,04912116 |
| NANOZOOG7009 | 25,7465831 | -0,8805556 | 0,33707613 | 0,00449942 | 0,03586818 |
| NANOZOOG7568 | 43,8143966 | 0,8684325 | 0,35081588 | 0,00696688 | 0,04859581 |
| NANOZOOG122 | 1831,64942 | -0,8409305 | 0,23387667 | 0,0001696 | 0,00261071 |
| NANOZOOG7607 | 56,4046615 | -0,8311544 | 0,32585698 | 0,00546112 | 0,04097368 |
| NANOZOOG4318 | 421,699662 | 0,80368444 | 0,25346553 | 0,00086872 | 0,01005124 |
| NANOZOOG766 | 40,6115685 | 0,78718313 | 0,27111123 | 0,00212699 | 0,02015776 |
| NANOZOOG3257 | 90,1680387 | 0,77604648 | 0,30991898 | 0,00707435 | 0,0490432 |
| NANOZOOG1312 | 37,2121983 | -0,7726349 | 0,29517201 | 0,00512769 | 0,03958072 |
| NANOZOOG4167 | 45,1953936 | -0,7621062 | 0,29189816 | 0,00520935 | 0,03988681 |
| NANOZOOG9044 | 1228,06035 | -0,7535852 | 0,296271 | 0,00637106 | 0,04562802 |
| NANOZOOG2596 | 35,8727935 | -0,7510011 | 0,29599774 | 0,00661984 | 0,04678425 |
| NANOZOOG5476 | 287,222275 | -0,6981216 | 0,25747941 | 0,00431946 | 0,03495371 |

Mean of normalized counts, fold-change, fold-change standard error, and p-values in genes that were differentially expressed between the samples CGHH 10 h and CG 30 h. CGHH 10 h and CG 30 h samples belong to three independent cultivations in culture medium containing as carbon source either CGHH (a mixture of hemicellulose hydrolysate and crude glycerol) or CG (crude glycerol), respectively. The samples were collected at 10 h and 30 h cultivation, respectively. Data was obtained using RNA-Seq.

Table S3: Differentially expressed genes between growth on different media when glycerol concentration is lower

| **Gene ID** | **baseMean** | **log2FoldChange** | **lfcSE** | **pvalue** | **padj** |
| --- | --- | --- | --- | --- | --- |
| NANOZOOG7221 | 31,1033687 | 7,59776522 | 3,36324049 | 0,00014453 | 0,00336492 |
| NANOZOOG6644 | 5,81187966 | 5,87848361 | 2,62410278 | 0,00016742 | 0,00365949 |
| NANOZOOG6579 | 3,81261933 | -3,508259 | 1,75067056 | 0,00166771 | 0,01789663 |
| NANOZOOG4321 | 7,29022995 | -3,4971355 | 1,39874718 | 0,00045629 | 0,00696683 |
| NANOZOOG4823 | 18,5821301 | -3,3929374 | 1,22329166 | 0,00018455 | 0,00386419 |
| NANOZOOG4748 | 3,79980533 | -3,3704473 | 1,71973211 | 0,00223447 | 0,02180179 |
| NANOZOOG6723 | 12,0472718 | -3,2765398 | 0,8058362 | 6,2052E-06 | 0,00035301 |
| NANOZOOG188 | 71,2275968 | -3,1930122 | 0,53853353 | 2,2187E-10 | 2,8741E-07 |
| NANOZOOG1700 | 4,26629481 | -3,0835322 | 1,43382755 | 0,0021276 | 0,02103793 |
| NANOZOOG4049 | 4,63877153 | -2,9237862 | 1,20942434 | 0,00195535 | 0,02000142 |
| NANOZOOG2663 | 11,1773885 | -2,885371 | 0,90049367 | 0,00018827 | 0,00387077 |
| NANOZOOG6151 | 51,5811901 | -2,881272 | 0,70602341 | 2,1862E-06 | 0,00015389 |
| NANOZOOG4706 | 28,7168902 | -2,8237977 | 0,94088785 | 9,957E-05 | 0,00253691 |
| NANOZOOG2969 | 23,2029585 | -2,6790474 | 0,80549579 | 4,1366E-05 | 0,00155686 |
| NANOZOOG6898 | 31,4554182 | -2,6646907 | 0,83271295 | 5,5409E-05 | 0,00177465 |
| NANOZOOG6816 | 20,6290017 | -2,6353005 | 0,92335756 | 0,00018167 | 0,00386419 |
| NANOZOOG4625 | 11,3826807 | -2,5902677 | 0,75341223 | 4,1611E-05 | 0,00155686 |
| NANOZOOG1932 | 17,9773575 | -2,5808683 | 0,65894285 | 1,0735E-05 | 0,00056983 |
| NANOZOOG4549 | 10,5651319 | -2,5106705 | 1,45583948 | 0,00274491 | 0,0250541 |
| NANOZOOG1567 | 149,708367 | -2,5084022 | 0,49353001 | 2,1719E-08 | 5,0111E-06 |
| NANOZOOG4366 | 6,0826476 | -2,4988271 | 1,45440998 | 0,00234734 | 0,02251386 |
| NANOZOOG2795 | 35,6566274 | -2,4965787 | 0,5809785 | 1,5034E-06 | 0,00011562 |
| NANOZOOG1795 | 11,2887092 | -2,475661 | 0,59756204 | 2,7738E-06 | 0,00018179 |
| NANOZOOG6182 | 9,64211249 | 2,46334907 | 0,6718885 | 2,2418E-05 | 0,0009598 |
| NANOZOOG9426 | 25,771583 | -2,4552104 | 1,39850032 | 0,00203461 | 0,02050911 |
| NANOZOOG4637 | 18,4596069 | -2,4039692 | 0,57888852 | 2,8015E-06 | 0,00018179 |
| NANOZOOG3566 | 6,84146771 | -2,4004198 | 0,80329405 | 0,0003295 | 0,00572561 |
| NANOZOOG3810 | 4,79821872 | -2,3946497 | 1,29992197 | 0,00416592 | 0,03326236 |
| NANOZOOG3481 | 29,0458468 | 2,35546076 | 0,38078407 | 4,6996E-11 | 9,7586E-08 |
| NANOZOOG6772 | 42,0164785 | -2,3512779 | 0,49444156 | 1,5207E-07 | 1,9736E-05 |
| NANOZOOG4803 | 8,90158941 | 2,33985143 | 0,48214533 | 1,6842E-07 | 2,0572E-05 |
| NANOZOOG4059 | 5,49717691 | -2,3301375 | 1,45912647 | 0,00647312 | 0,04450805 |
| NANOZOOG3179 | 104,649965 | -2,3174465 | 1,0212449 | 0,00075524 | 0,0102301 |
| NANOZOOG6164 | 4,21969435 | -2,308416 | 1,11802683 | 0,00239126 | 0,02277725 |
| NANOZOOG2089 | 29,938812 | -2,3036953 | 0,42781975 | 6,244E-09 | 1,6207E-06 |
| NANOZOOG7897 | 18,8863237 | 2,29580242 | 0,47269836 | 9,3342E-08 | 1,3367E-05 |
| NANOZOOG5333 | 11,0686791 | -2,2852017 | 0,78715612 | 0,00024587 | 0,00464134 |
| NANOZOOG1241 | 40,2623149 | -2,2672095 | 0,6082399 | 1,0839E-05 | 0,00056983 |
| NANOZOOG1826 | 5,68693779 | -2,2470136 | 1,32824536 | 0,00739651 | 0,04899157 |
| NANOZOOG4713 | 31,1778052 | -2,2419621 | 0,86955073 | 0,00043195 | 0,00671875 |
| NANOZOOG6693 | 472,154199 | -2,2416244 | 0,50058329 | 4,3832E-07 | 4,2334E-05 |
| NANOZOOG9219 | 8,45529913 | -2,2135458 | 0,90142849 | 0,00072313 | 0,00997722 |
| NANOZOOG6597 | 9,82082784 | -2,1988055 | 0,71997134 | 0,00019728 | 0,00403604 |
| NANOZOOG606 | 24,1804678 | -2,1922187 | 0,48212123 | 4,0791E-07 | 4,0835E-05 |
| NANOZOOG7751 | 5,69349129 | -2,1887427 | 0,73160434 | 0,00033138 | 0,00573432 |
| NANOZOOG5287 | 21,7280131 | 2,18200936 | 0,7614208 | 0,00021376 | 0,00426797 |
| NANOZOOG4707 | 14,7122943 | 2,17568393 | 1,59877994 | 0,00434476 | 0,0341092 |
| NANOZOOG6733 | 6,93348615 | -2,1664413 | 1,02929121 | 0,00175615 | 0,01854601 |
| NANOZOOG8743 | 39,2513031 | -2,1528407 | 0,46572408 | 3,1648E-07 | 3,3701E-05 |
| NANOZOOG3929 | 157,426197 | -2,1306485 | 0,39577578 | 5,107E-09 | 1,5149E-06 |
| NANOZOOG189 | 13,1517645 | -2,1269062 | 0,979408 | 0,00134038 | 0,01517697 |
| NANOZOOG3737 | 14,856422 | 2,1162472 | 0,46821837 | 5,657E-07 | 5,1072E-05 |
| NANOZOOG7772 | 19,1539824 | 2,11143692 | 0,37102955 | 1,0761E-09 | 4,9654E-07 |
| NANOZOOG7015 | 12,0850088 | -2,0797 | 0,63916407 | 9,3818E-05 | 0,00243517 |
| NANOZOOG3156 | 6,59915134 | -2,0553341 | 0,88814427 | 0,00164259 | 0,01776477 |
| NANOZOOG6201 | 8,21467673 | -2,0533144 | 0,76402151 | 0,00052635 | 0,00777911 |
| NANOZOOG1674 | 8,20348682 | -2,0508609 | 1,11955509 | 0,00243344 | 0,02307324 |
| NANOZOOG6922 | 7,59714324 | -2,0494819 | 1,135109 | 0,00396497 | 0,03222412 |
| NANOZOOG4678 | 10,4935775 | -2,0318707 | 0,94386264 | 0,00140822 | 0,01572134 |
| NANOZOOG6763 | 20,4783982 | -2,0318312 | 0,79946572 | 0,0005701 | 0,00827848 |
| NANOZOOG4705 | 18,5218936 | -2,0257909 | 0,91489424 | 0,00107262 | 0,01291187 |
| NANOZOOG3215 | 14,6573566 | -2,024382 | 0,62378854 | 8,1122E-05 | 0,00217594 |
| NANOZOOG4472 | 37,413628 | -2,0152335 | 0,42972572 | 1,8565E-07 | 2,1416E-05 |
| NANOZOOG9223 | 20,0723074 | -2,0054864 | 0,61378729 | 6,9935E-05 | 0,00197577 |
| NANOZOOG6337 | 7,53634566 | -2,0052492 | 0,87553646 | 0,00152269 | 0,01677387 |
| NANOZOOG4152 | 19,5058208 | -2,003745 | 0,58213849 | 4,6526E-05 | 0,00161019 |
| NANOZOOG5313 | 16,0954071 | 2,00236928 | 0,43774051 | 4,067E-07 | 4,0835E-05 |
| NANOZOOG6908 | 5,38206568 | 1,98419517 | 0,63430761 | 0,00014924 | 0,00342425 |
| NANOZOOG3028 | 17,1841346 | -1,9792403 | 0,56365273 | 4,6285E-05 | 0,00161019 |
| NANOZOOG9153 | 72,7839524 | -1,9781298 | 0,80068748 | 0,00061881 | 0,0088313 |
| NANOZOOG2043 | 5,34561019 | -1,9721695 | 0,86888789 | 0,00201401 | 0,02041375 |
| NANOZOOG5843 | 17,3996826 | -1,968123 | 0,46567778 | 2,4137E-06 | 0,00016168 |
| NANOZOOG9017 | 32,2806043 | -1,9667596 | 0,34283278 | 1,0302E-09 | 4,9654E-07 |
| NANOZOOG7863 | 81,1573194 | -1,9634538 | 0,6158659 | 8,1735E-05 | 0,00217594 |
| NANOZOOG2610 | 6,53734727 | 1,9538421 | 0,86924269 | 0,00149395 | 0,01654495 |
| NANOZOOG9071 | 17,0074573 | 1,93949123 | 0,3283516 | 3,4592E-10 | 2,8741E-07 |
| NANOZOOG7621 | 35,7543463 | -1,9333858 | 0,38104811 | 3,3087E-08 | 6,5433E-06 |
| NANOZOOG4984 | 8,4806398 | -1,9305052 | 1,0583149 | 0,00327405 | 0,02862551 |
| NANOZOOG3112 | 14,6311159 | -1,9301676 | 0,80266196 | 0,0011509 | 0,01357868 |
| NANOZOOG5092 | 14,5829782 | -1,9243671 | 0,73248744 | 0,00069905 | 0,00967722 |
| NANOZOOG6392 | 7,35954835 | 1,92356372 | 0,56998662 | 6,9717E-05 | 0,00197577 |
| NANOZOOG5403 | 14,6529964 | 1,91939619 | 0,56621749 | 5,8286E-05 | 0,0018338 |
| NANOZOOG9051 | 9,98308463 | -1,911357 | 0,71541651 | 0,00050154 | 0,00746551 |
| NANOZOOG8043 | 8,56642485 | -1,9062242 | 1,97152912 | 0,00561309 | 0,04079042 |
| NANOZOOG2150 | 206,475206 | -1,8960575 | 0,40198253 | 1,7378E-07 | 2,0621E-05 |
| NANOZOOG1635 | 5,66991972 | -1,8860937 | 1,08333412 | 0,00469403 | 0,03603387 |
| NANOZOOG3041 | 9,77639713 | 1,87386523 | 0,73274283 | 0,00067988 | 0,00950685 |
| NANOZOOG7654 | 19,6567899 | -1,8726 | 0,52343715 | 3,1706E-05 | 0,00125403 |
| NANOZOOG9336 | 5,80266689 | 1,87203279 | 0,77595752 | 0,00118693 | 0,01392468 |
| NANOZOOG3640 | 12,7056686 | 1,87001536 | 0,48596085 | 1,004E-05 | 0,00054861 |
| NANOZOOG6934 | 13,148244 | 1,84701803 | 0,45282889 | 3,5912E-06 | 0,00021615 |
| NANOZOOG6906 | 6,17492077 | 1,84607861 | 0,91918406 | 0,0026423 | 0,02443978 |
| NANOZOOG8224 | 6,90999857 | -1,8451013 | 0,58715614 | 0,000152 | 0,00344951 |
| NANOZOOG7251 | 13,8342354 | 1,84208435 | 0,44689298 | 3,5147E-06 | 0,00021466 |
| NANOZOOG5209 | 24,1999875 | -1,8412393 | 1,31051581 | 0,00453181 | 0,0352446 |
| NANOZOOG2507 | 23,2162728 | -1,8303965 | 1,63192285 | 0,00519379 | 0,0383859 |
| NANOZOOG4780 | 40,926465 | -1,8254675 | 0,32555777 | 1,9849E-09 | 7,8137E-07 |
| NANOZOOG2724 | 75,0943071 | -1,8237838 | 1,24211128 | 0,00407691 | 0,03287655 |
| NANOZOOG2648 | 7,73600854 | -1,8220585 | 0,61085226 | 0,00026336 | 0,0048366 |
| NANOZOOG3735 | 4,27285917 | -1,82145 | 1,22547765 | 0,0073109 | 0,04850189 |
| NANOZOOG740 | 32,2520998 | -1,8196866 | 0,51594786 | 3,765E-05 | 0,0014613 |
| NANOZOOG4247 | 8,90049846 | -1,8151116 | 0,95516377 | 0,00399178 | 0,03237862 |
| NANOZOOG4464 | 19,2651512 | 1,79918128 | 0,3371974 | 7,4373E-09 | 1,8169E-06 |
| NANOZOOG1377 | 10,2512016 | -1,7965132 | 0,6427632 | 0,00049165 | 0,00737125 |
| NANOZOOG8515 | 5,37346491 | -1,7964347 | 1,45873756 | 0,00697931 | 0,04673178 |
| NANOZOOG8507 | 21,44485 | 1,79583438 | 0,36815328 | 9,0571E-08 | 1,3367E-05 |
| NANOZOOG7914 | 9,69763874 | 1,79302122 | 0,52170756 | 4,3013E-05 | 0,00157064 |
| NANOZOOG9154 | 41,6005009 | -1,7896578 | 0,7932461 | 0,00128225 | 0,01475118 |
| NANOZOOG5138 | 26,0706973 | 1,78909086 | 0,31908964 | 2,0696E-09 | 7,8137E-07 |
| NANOZOOG4061 | 26,2855273 | -1,7825863 | 0,60938574 | 0,00026317 | 0,0048366 |
| NANOZOOG5629 | 6,58536152 | 1,77720794 | 1,99718322 | 0,00679813 | 0,04590673 |
| NANOZOOG435 | 9,33617492 | -1,7769743 | 0,97428919 | 0,00330315 | 0,0287144 |
| NANOZOOG7143 | 4,29384416 | -1,773697 | 1,1349329 | 0,00746387 | 0,04920228 |
| NANOZOOG4346 | 3,79840287 | -1,7678061 | 1,02764315 | 0,00606192 | 0,0428149 |
| NANOZOOG6005 | 5,37801627 | -1,7676805 | 0,82457355 | 0,00278168 | 0,0252786 |
| NANOZOOG7137 | 7,3315722 | -1,7644303 | 0,89149387 | 0,0032315 | 0,02841278 |
| NANOZOOG9246 | 5,58339478 | -1,7624924 | 0,7139082 | 0,0009838 | 0,01207874 |
| NANOZOOG4572 | 4,80194971 | -1,7529578 | 0,71850694 | 0,00117926 | 0,01387384 |
| NANOZOOG4664 | 12,8881063 | -1,7452471 | 0,55667571 | 0,0001508 | 0,00344099 |
| NANOZOOG8990 | 5,72069668 | -1,7433907 | 1,52499845 | 0,00677604 | 0,04586385 |
| NANOZOOG2579 | 7,57870551 | -1,7413022 | 0,91989382 | 0,00428565 | 0,03379957 |
| NANOZOOG3548 | 121,478873 | -1,741301 | 0,39374699 | 8,7893E-07 | 7,4494E-05 |
| NANOZOOG8704 | 25,3296908 | -1,739277 | 1,02596643 | 0,00357783 | 0,03038596 |
| NANOZOOG7252 | 14,0752447 | 1,7354554 | 1,09269637 | 0,00694857 | 0,04661939 |
| NANOZOOG2372 | 57,0028491 | -1,73308 | 0,44827916 | 8,6048E-06 | 0,00048291 |
| NANOZOOG3200 | 4,89663256 | -1,7288878 | 0,84636917 | 0,00307383 | 0,02727635 |
| NANOZOOG4737 | 11,5623122 | 1,72608354 | 0,50711769 | 6,0199E-05 | 0,00186437 |
| NANOZOOG5110 | 42,2354385 | -1,7245105 | 0,48952408 | 3,336E-05 | 0,00130701 |
| NANOZOOG4086 | 6,84513299 | -1,7158399 | 0,83318254 | 0,00344136 | 0,02946794 |
| NANOZOOG3834 | 6,13010234 | -1,709376 | 0,76779362 | 0,00246453 | 0,02331477 |
| NANOZOOG6242 | 15,6022472 | 1,70705599 | 0,48015542 | 3,0509E-05 | 0,00123388 |
| NANOZOOG4213 | 6,45657904 | -1,7067754 | 1,03275544 | 0,00566935 | 0,04094746 |
| NANOZOOG5923 | 86,5906967 | -1,705923 | 0,51792246 | 6,9252E-05 | 0,00197577 |
| NANOZOOG3505 | 6,9297119 | -1,7056005 | 0,85114544 | 0,00359097 | 0,03043532 |
| NANOZOOG3198 | 54,2336566 | 1,70476388 | 0,33464713 | 2,6119E-08 | 5,4236E-06 |
| NANOZOOG3925 | 6,42259336 | -1,7046002 | 0,87329724 | 0,00329403 | 0,0287144 |
| NANOZOOG9218 | 3,85509157 | -1,7035536 | 0,99918713 | 0,00644047 | 0,04435703 |
| NANOZOOG738 | 41,0503112 | -1,6988729 | 0,33435084 | 3,6891E-08 | 6,9641E-06 |
| NANOZOOG1066 | 15,2990982 | 1,69495105 | 0,45611311 | 1,9673E-05 | 0,00086916 |
| NANOZOOG7885 | 20,5649146 | -1,6915094 | 0,65595155 | 0,00065506 | 0,00928607 |
| NANOZOOG3210 | 30,4776656 | 1,69004448 | 0,412499 | 3,0645E-06 | 0,00019283 |
| NANOZOOG7240 | 127,611873 | -1,6889746 | 0,44558163 | 1,1596E-05 | 0,00059182 |
| NANOZOOG7347 | 6,63208747 | -1,6885142 | 1,38245277 | 0,00649251 | 0,04455927 |
| NANOZOOG1173 | 5,9564204 | 1,68553388 | 0,76866738 | 0,00192707 | 0,01980969 |
| NANOZOOG4241 | 3,79100374 | -1,6823908 | 0,71922618 | 0,00195377 | 0,02000142 |
| NANOZOOG1378 | 28,4906706 | -1,681248 | 0,48000682 | 4,2471E-05 | 0,00157064 |
| NANOZOOG9367 | 16,5625846 | 1,67879267 | 0,44372728 | 1,1925E-05 | 0,00059358 |
| NANOZOOG3057 | 16,0389307 | -1,6748933 | 0,97688559 | 0,0033746 | 0,02919732 |
| NANOZOOG1785 | 17,940385 | -1,6670251 | 0,61089202 | 0,00053872 | 0,00790962 |
| NANOZOOG4038 | 13,7239334 | -1,6669521 | 0,65980688 | 0,00088018 | 0,01117861 |
| NANOZOOG6122 | 12,8848845 | 1,66680179 | 0,48292845 | 4,8306E-05 | 0,00164518 |
| NANOZOOG1067 | 119,474499 | -1,6662048 | 0,40762923 | 3,4846E-06 | 0,00021466 |
| NANOZOOG7121 | 32,8478384 | -1,6652583 | 0,6640581 | 0,0007664 | 0,01030051 |
| NANOZOOG1991 | 20,5466405 | -1,6647851 | 0,59083492 | 0,00038872 | 0,00637245 |
| NANOZOOG1080 | 42,8041903 | -1,6645936 | 0,28849819 | 8,536E-10 | 4,9654E-07 |
| NANOZOOG4336 | 27,3291038 | 1,66348546 | 0,32319346 | 2,4327E-08 | 5,3173E-06 |
| NANOZOOG5926 | 5,36357943 | -1,6631366 | 0,65913292 | 0,00113483 | 0,01346554 |
| NANOZOOG2433 | 6,43961302 | -1,6543339 | 0,69759753 | 0,00161103 | 0,01756066 |
| NANOZOOG1480 | 9,77859047 | -1,651728 | 0,53472733 | 0,00022205 | 0,00430925 |
| NANOZOOG2233 | 30,7881909 | 1,64694234 | 0,29810897 | 2,929E-09 | 1,0137E-06 |
| NANOZOOG5690 | 16,0600813 | -1,6462196 | 0,57980786 | 0,00039304 | 0,00637245 |
| NANOZOOG2181 | 8,94846455 | -1,6456285 | 0,60259069 | 0,00066802 | 0,00940429 |
| NANOZOOG7068 | 9,3243468 | -1,643689 | 0,65073657 | 0,00097504 | 0,01205165 |
| NANOZOOG4481 | 4,02477424 | -1,6428367 | 1,05107904 | 0,00497964 | 0,03753257 |
| NANOZOOG7990 | 42,865065 | -1,642755 | 1,25692713 | 0,00584493 | 0,04188805 |
| NANOZOOG3229 | 8,92015241 | -1,6413936 | 0,71531319 | 0,00175948 | 0,01854601 |
| NANOZOOG7299 | 96,8537927 | -1,6357584 | 0,33069577 | 7,4995E-08 | 1,2013E-05 |
| NANOZOOG2378 | 20,160664 | -1,6298664 | 0,46937573 | 4,5938E-05 | 0,00161019 |
| NANOZOOG5717 | 6,29514564 | -1,6293772 | 0,63598459 | 0,00100689 | 0,01226276 |
| NANOZOOG7725 | 29,1925028 | 1,62275687 | 0,29927097 | 5,9556E-09 | 1,6207E-06 |
| NANOZOOG6428 | 33,3878016 | -1,622221 | 0,58872122 | 0,00045036 | 0,00690168 |
| NANOZOOG3882 | 17,2239549 | 1,61747882 | 0,53724102 | 0,00020946 | 0,00420494 |
| NANOZOOG650 | 10,0519092 | -1,6165733 | 0,77940334 | 0,00238254 | 0,02274645 |
| NANOZOOG2306 | 7,35543389 | -1,6143901 | 0,6555107 | 0,00131261 | 0,01501616 |
| NANOZOOG4285 | 20,2392175 | 1,6098075 | 0,59783962 | 0,0004699 | 0,00712229 |
| NANOZOOG5976 | 15,4183667 | 1,60560778 | 0,59770691 | 0,00047852 | 0,0072265 |
| NANOZOOG2623 | 13,1754107 | -1,6003964 | 0,69749938 | 0,00135724 | 0,01531688 |
| NANOZOOG7965 | 17,8892651 | -1,6003718 | 0,47760934 | 7,3681E-05 | 0,0020381 |
| NANOZOOG7988 | 9,78794233 | -1,596083 | 0,64598718 | 0,00109054 | 0,01308967 |
| NANOZOOG1037 | 10,7204227 | -1,590992 | 0,68797921 | 0,00185674 | 0,01926413 |
| NANOZOOG3298 | 26,0788635 | 1,59097415 | 0,36385352 | 1,1945E-06 | 9,5403E-05 |
| NANOZOOG6916 | 20,6179429 | -1,5903012 | 0,42741086 | 1,8121E-05 | 0,00082699 |
| NANOZOOG4192 | 13,7347415 | 1,58911547 | 0,50361739 | 0,00013245 | 0,00317949 |
| NANOZOOG5018 | 13,1607688 | 1,58295614 | 0,40453382 | 8,8508E-06 | 0,0004901 |
| NANOZOOG803 | 36,1151097 | -1,5828283 | 0,62310314 | 0,00083434 | 0,01079447 |
| NANOZOOG8569 | 59,9520979 | -1,5827772 | 0,7261668 | 0,00174229 | 0,01850574 |
| NANOZOOG7824 | 6,55783263 | -1,5807654 | 0,8342696 | 0,00361045 | 0,03053811 |
| NANOZOOG194 | 28,0466938 | 1,5754842 | 0,37427958 | 2,1286E-06 | 0,00015389 |
| NANOZOOG6116 | 5,95042581 | 1,57097911 | 0,71694417 | 0,00218814 | 0,02153399 |
| NANOZOOG973 | 641,375708 | 1,570617 | 0,27005063 | 6,0936E-10 | 4,2178E-07 |
| NANOZOOG694 | 29,3016347 | -1,570608 | 0,41252009 | 1,5948E-05 | 0,00074418 |
| NANOZOOG4121 | 32,4773694 | 1,56815463 | 0,37014068 | 2,3101E-06 | 0,00015727 |
| NANOZOOG4087 | 5,45383625 | -1,5643887 | 0,95965824 | 0,0075273 | 0,0494634 |
| NANOZOOG7632 | 16,6582547 | 1,56140195 | 0,4525591 | 4,8726E-05 | 0,00164518 |
| NANOZOOG5922 | 7,00134917 | -1,5595245 | 0,69576023 | 0,00250784 | 0,02349976 |
| NANOZOOG1586 | 37,183595 | -1,5544662 | 0,73264231 | 0,0021346 | 0,02105697 |
| NANOZOOG2464 | 38,2779578 | 1,55431949 | 0,33877351 | 4,1297E-07 | 4,0835E-05 |
| NANOZOOG7488 | 8,24318984 | -1,5470401 | 0,60435645 | 0,00097247 | 0,01205165 |
| NANOZOOG7096 | 19,7154562 | -1,5463834 | 0,42212658 | 2,5903E-05 | 0,00107577 |
| NANOZOOG2683 | 20,2728066 | 1,5422825 | 0,70305884 | 0,00182353 | 0,01902791 |
| NANOZOOG5270 | 5,03699721 | -1,5410217 | 0,70509282 | 0,00263807 | 0,02443978 |
| NANOZOOG6037 | 32,958775 | 1,52901863 | 0,35839586 | 1,999E-06 | 0,00014825 |
| NANOZOOG6289 | 14,703732 | -1,5223546 | 1,09115331 | 0,00698782 | 0,04673178 |
| NANOZOOG609 | 33,5477408 | 1,5206011 | 0,34199328 | 9,4597E-07 | 7,8572E-05 |
| NANOZOOG8367 | 12,9725809 | 1,51744527 | 0,45573429 | 8,8447E-05 | 0,00231019 |
| NANOZOOG6324 | 19,7135082 | 1,51692738 | 0,42621954 | 3,0602E-05 | 0,00123388 |
| NANOZOOG500 | 15,2481707 | 1,51409197 | 0,58701347 | 0,00072972 | 0,01000174 |
| NANOZOOG7831 | 10,9422793 | 1,51398973 | 0,66885925 | 0,00163901 | 0,01776477 |
| NANOZOOG6199 | 18,0383189 | -1,5124495 | 0,53250464 | 0,00039151 | 0,00637245 |
| NANOZOOG1451 | 22,8738492 | 1,51041495 | 0,3048867 | 8,4158E-08 | 1,2945E-05 |
| NANOZOOG8096 | 40,085298 | -1,5077912 | 0,54078248 | 0,00045972 | 0,00699344 |
| NANOZOOG5238 | 65,4679197 | -1,5062328 | 0,34810146 | 1,5887E-06 | 0,00011996 |
| NANOZOOG2766 | 23,971511 | -1,5022822 | 0,96755237 | 0,00578398 | 0,0415586 |
| NANOZOOG6538 | 6,04609347 | 1,50064464 | 0,81480886 | 0,00412233 | 0,03310929 |
| NANOZOOG7123 | 157,869722 | -1,4988763 | 0,54455787 | 0,00044279 | 0,00683608 |
| NANOZOOG8223 | 26,8106476 | -1,4948115 | 0,7920244 | 0,00323603 | 0,02841278 |
| NANOZOOG1168 | 8,93847749 | 1,49215504 | 0,52283827 | 0,00039801 | 0,00640668 |
| NANOZOOG1740 | 24,1993551 | -1,4920383 | 0,43787197 | 6,6121E-05 | 0,00197282 |
| NANOZOOG7978 | 41,921181 | -1,4918549 | 0,32441539 | 4,8342E-07 | 4,4911E-05 |
| NANOZOOG4778 | 24,9590806 | 1,47729299 | 0,30230414 | 1,1236E-07 | 1,5554E-05 |
| NANOZOOG3519 | 7,37490052 | 1,47304712 | 0,48789561 | 0,00026187 | 0,0048366 |
| NANOZOOG2180 | 8,92477418 | 1,46739049 | 0,51795396 | 0,00042852 | 0,00669039 |
| NANOZOOG1039 | 5,09084901 | -1,4660257 | 0,86764085 | 0,00749805 | 0,04934927 |
| NANOZOOG6676 | 6,16749424 | 1,46486459 | 0,65533274 | 0,00202857 | 0,02049789 |
| NANOZOOG4188 | 16,3004164 | -1,4643864 | 0,59072482 | 0,00114792 | 0,01357868 |
| NANOZOOG5159 | 11,5223845 | -1,4622867 | 4,68190107 | 0,0070863 | 0,04731414 |
| NANOZOOG5086 | 28,9377396 | 1,46208318 | 0,31979367 | 4,8663E-07 | 4,4911E-05 |
| NANOZOOG5058 | 154,16404 | -1,4599119 | 0,41411323 | 4,3114E-05 | 0,00157064 |
| NANOZOOG5723 | 20,437289 | 1,4597827 | 0,37517783 | 1,1518E-05 | 0,00059182 |
| NANOZOOG6809 | 26,9593259 | 1,45887676 | 0,29302424 | 7,5206E-08 | 1,2013E-05 |
| NANOZOOG1024 | 9,83477282 | -1,458604 | 0,76174178 | 0,00389056 | 0,03207882 |
| NANOZOOG3276 | 54,1606763 | -1,4583295 | 0,83179002 | 0,00439554 | 0,03444282 |
| NANOZOOG948 | 5,72711198 | 1,45800215 | 0,83626952 | 0,00519453 | 0,0383859 |
| NANOZOOG4364 | 17,2145877 | 1,45368284 | 0,42697687 | 6,8414E-05 | 0,00197577 |
| NANOZOOG4509 | 81,5214626 | 1,4496315 | 0,20116197 | 8,1034E-14 | 3,3654E-10 |
| NANOZOOG3260 | 28,4804106 | 1,44944332 | 0,35288401 | 4,2956E-06 | 0,00025485 |
| NANOZOOG3056 | 28,2180744 | 1,44871729 | 0,79887195 | 0,00414306 | 0,03315243 |
| NANOZOOG19 | 4,30468164 | -1,448537 | 0,82121517 | 0,00511377 | 0,03799191 |
| NANOZOOG7853 | 20,1755122 | -1,4480463 | 0,4871775 | 0,0002801 | 0,00505757 |
| NANOZOOG7446 | 15,5086983 | 1,44373261 | 0,39591613 | 2,8225E-05 | 0,00116056 |
| NANOZOOG8287 | 12,2182212 | 1,43972723 | 0,49686413 | 0,00040041 | 0,00642041 |
| NANOZOOG980 | 11,3371773 | 1,43531332 | 0,41614889 | 6,2482E-05 | 0,00189408 |
| NANOZOOG6713 | 7,86686843 | 1,43402172 | 0,93734186 | 0,00711591 | 0,04743558 |
| NANOZOOG4779 | 54,0184453 | -1,4331451 | 0,35343787 | 4,8231E-06 | 0,00028212 |
| NANOZOOG1744 | 18,2764723 | -1,4322724 | 0,77438357 | 0,00428742 | 0,03379957 |
| NANOZOOG2657 | 8,88414802 | 1,43178328 | 0,67506644 | 0,00270629 | 0,02486554 |
| NANOZOOG8681 | 12,1296796 | -1,4311686 | 0,74931519 | 0,00365867 | 0,03069588 |
| NANOZOOG352 | 42,3142094 | 1,42772498 | 0,37098307 | 1,3072E-05 | 0,00063866 |
| NANOZOOG7793 | 15,2521778 | 1,42483915 | 0,39630785 | 3,1388E-05 | 0,00125342 |
| NANOZOOG3620 | 7,2932912 | -1,424134 | 0,75857136 | 0,00565652 | 0,04092604 |
| NANOZOOG7310 | 9,4072229 | -1,4209067 | 0,80564916 | 0,00501516 | 0,03773178 |
| NANOZOOG1933 | 35,4749831 | 1,42018375 | 0,29305378 | 1,398E-07 | 1,8729E-05 |
| NANOZOOG5795 | 21,5994113 | 1,41905546 | 0,29559372 | 1,952E-07 | 2,191E-05 |
| NANOZOOG4170 | 26,2515752 | 1,41811335 | 0,46835467 | 0,00022974 | 0,00441718 |
| NANOZOOG7893 | 30,5064173 | -1,4100954 | 0,41480835 | 6,9831E-05 | 0,00197577 |
| NANOZOOG2692 | 16,9423008 | 1,4068725 | 0,64881282 | 0,00225936 | 0,02187209 |
| NANOZOOG5878 | 12,6821472 | 1,40605526 | 0,43747837 | 0,00013442 | 0,00320832 |
| NANOZOOG944 | 19,7258083 | 1,40498307 | 0,39378136 | 3,9484E-05 | 0,00150436 |
| NANOZOOG111 | 19,8810081 | 1,40492764 | 0,36494358 | 1,2006E-05 | 0,00059358 |
| NANOZOOG140 | 11,956456 | 1,40270362 | 0,43808365 | 0,00014781 | 0,00341029 |
| NANOZOOG6031 | 19,7941626 | 1,4025321 | 0,48268478 | 0,00036481 | 0,00610909 |
| NANOZOOG5140 | 211,804977 | -1,3995876 | 0,47740123 | 0,00027661 | 0,00503848 |
| NANOZOOG1692 | 50,4930618 | -1,3986557 | 0,53629977 | 0,00074506 | 0,01014501 |
| NANOZOOG6396 | 19,1678493 | 1,395626 | 0,46224233 | 0,00024381 | 0,00464134 |
| NANOZOOG5632 | 14,5431109 | 1,39236051 | 0,36252978 | 1,4728E-05 | 0,00070307 |
| NANOZOOG4907 | 8,01604105 | 1,39072099 | 0,56575284 | 0,00136648 | 0,0153794 |
| NANOZOOG330 | 35,3831241 | -1,3891877 | 0,31580435 | 1,2237E-06 | 9,5891E-05 |
| NANOZOOG8584 | 13,4009641 | 1,38732362 | 0,40707667 | 7,4104E-05 | 0,0020381 |
| NANOZOOG2598 | 10,2384527 | 1,37885651 | 0,53735664 | 0,0010066 | 0,01226276 |
| NANOZOOG1192 | 19,0849628 | 1,37732171 | 0,5221715 | 0,00074266 | 0,01014501 |
| NANOZOOG2988 | 8,10474228 | 1,37436058 | 0,581706 | 0,00166192 | 0,01788073 |
| NANOZOOG4185 | 19,1298819 | -1,3733925 | 0,77555492 | 0,00518534 | 0,0383859 |
| NANOZOOG682 | 10,7351832 | 1,37149036 | 0,44913522 | 0,00024821 | 0,00466441 |
| NANOZOOG3744 | 99,9214103 | -1,3710693 | 0,2717494 | 6,3268E-08 | 1,0948E-05 |
| NANOZOOG6981 | 13,8675248 | 1,36989153 | 0,44471618 | 0,00019895 | 0,00405022 |
| NANOZOOG8835 | 11,8839696 | 1,36736201 | 0,44018717 | 0,00021891 | 0,00430925 |
| NANOZOOG88 | 17,1502149 | 1,3659911 | 0,38993627 | 4,9935E-05 | 0,00165061 |
| NANOZOOG1643 | 8,41776554 | 1,36316675 | 0,62726686 | 0,00230493 | 0,02226132 |
| NANOZOOG5339 | 1084,53264 | -1,362745 | 0,36008205 | 1,6155E-05 | 0,00074546 |
| NANOZOOG5671 | 11,0448569 | 1,36049461 | 0,43364753 | 0,00018721 | 0,00386802 |
| NANOZOOG7290 | 17,8595793 | -1,3600603 | 0,50146248 | 0,00077352 | 0,01036271 |
| NANOZOOG1261 | 7,38294406 | 1,35983637 | 0,60562817 | 0,00237571 | 0,02273349 |
| NANOZOOG5967 | 22,7526873 | 1,35749016 | 0,35048205 | 1,1685E-05 | 0,00059182 |
| NANOZOOG2171 | 11,8558736 | 1,35563349 | 0,44681426 | 0,00027813 | 0,00504391 |
| NANOZOOG4338 | 8,40505067 | 1,3550143 | 0,50940409 | 0,00080843 | 0,01065846 |
| NANOZOOG1950 | 32,5731134 | 1,35415961 | 0,24423442 | 3,3608E-09 | 1,0737E-06 |
| NANOZOOG7884 | 15,5591625 | -1,3531616 | 0,60825044 | 0,00254129 | 0,02371676 |
| NANOZOOG6265 | 9,81073691 | 1,35101772 | 0,51085094 | 0,00086695 | 0,01104431 |
| NANOZOOG1541 | 20,2764937 | 1,34750487 | 0,40239462 | 9,7904E-05 | 0,00251128 |
| NANOZOOG8407 | 11,9997841 | -1,3466096 | 0,52071313 | 0,00111932 | 0,01335782 |
| NANOZOOG349 | 16,5078988 | 1,34274186 | 0,435297 | 0,00020729 | 0,00419945 |
| NANOZOOG5079 | 14,0874049 | 1,33999487 | 0,34945907 | 1,5712E-05 | 0,00074148 |
| NANOZOOG7166 | 29,6826763 | -1,3398623 | 0,38471593 | 5,957E-05 | 0,0018601 |
| NANOZOOG8209 | 9,44115425 | 1,33917438 | 0,51695316 | 0,00097438 | 0,01205165 |
| NANOZOOG4562 | 29,9400609 | -1,3355525 | 0,46867583 | 0,00042329 | 0,00664855 |
| NANOZOOG1078 | 9,34134063 | 1,33434027 | 0,52300616 | 0,00094172 | 0,01181565 |
| NANOZOOG797 | 18,5021964 | 1,33265406 | 0,40809506 | 0,0001219 | 0,00303152 |
| NANOZOOG8463 | 22,4777775 | 1,33242837 | 0,42973097 | 0,00018606 | 0,00386419 |
| NANOZOOG2713 | 42,2113882 | -1,3312684 | 0,52443684 | 0,00112264 | 0,01335907 |
| NANOZOOG678 | 24,5419671 | -1,330699 | 0,45306831 | 0,00041102 | 0,00654005 |
| NANOZOOG6540 | 11,3628275 | 1,33045575 | 0,4339737 | 0,00026116 | 0,0048366 |
| NANOZOOG3161 | 7,2546732 | -1,3303865 | 0,62987529 | 0,0035343 | 0,03007777 |
| NANOZOOG7586 | 7,27078619 | -1,3288682 | 0,68484467 | 0,00502492 | 0,03773684 |
| NANOZOOG1566 | 74,9339159 | -1,3274996 | 0,66450903 | 0,00344128 | 0,02946794 |
| NANOZOOG871 | 29,3835862 | 1,32713848 | 0,27995023 | 2,7274E-07 | 2,9808E-05 |
| NANOZOOG6790 | 27,2594694 | -1,3260302 | 0,34799624 | 1,8596E-05 | 0,00083601 |
| NANOZOOG4485 | 38,5847573 | 1,3251775 | 0,25908681 | 4,2475E-08 | 7,6695E-06 |
| NANOZOOG5931 | 11,4984672 | 1,3241723 | 0,44287562 | 0,00029922 | 0,00531057 |
| NANOZOOG214 | 7,58640769 | 1,32412902 | 0,65485744 | 0,00377274 | 0,03133637 |
| NANOZOOG4645 | 39,3667043 | -1,3224306 | 0,29067258 | 8,0987E-07 | 7,007E-05 |
| NANOZOOG9274 | 23,8387545 | -1,3203731 | 0,41852938 | 0,00018008 | 0,00386297 |
| NANOZOOG1781 | 13,9124233 | 1,31997091 | 0,42018727 | 0,00018586 | 0,00386419 |
| NANOZOOG7167 | 13,5986413 | -1,3194358 | 0,70878609 | 0,00454799 | 0,03530432 |
| NANOZOOG1858 | 6,44246789 | -1,3176269 | 0,74783863 | 0,00626502 | 0,04365543 |
| NANOZOOG8435 | 10,377095 | 1,31445067 | 0,43821414 | 0,00031823 | 0,00561031 |
| NANOZOOG2077 | 9,93847516 | -1,3122811 | 0,72440765 | 0,00536094 | 0,03947514 |
| NANOZOOG5236 | 308,655894 | -1,311991 | 0,31204933 | 2,9941E-06 | 0,0001913 |
| NANOZOOG6492 | 15,4737877 | 1,31137668 | 0,39159065 | 8,5987E-05 | 0,00226014 |
| NANOZOOG9035 | 20,3293022 | 1,31069305 | 0,37528023 | 5,5598E-05 | 0,00177465 |
| NANOZOOG9344 | 10,3703066 | -1,3046696 | 0,69248984 | 0,00428904 | 0,03379957 |
| NANOZOOG7075 | 13,469198 | 1,30296293 | 0,40657639 | 0,00016135 | 0,00358325 |
| NANOZOOG2032 | 19,9240556 | 1,3017658 | 0,38134162 | 7,0565E-05 | 0,00198011 |
| NANOZOOG7214 | 2522,45957 | -1,3010444 | 0,21867833 | 3,4603E-10 | 2,8741E-07 |
| NANOZOOG2886 | 26,9146377 | 1,30096126 | 0,30164949 | 2,1852E-06 | 0,00015389 |
| NANOZOOG6961 | 14,4047214 | -1,2994352 | 0,68112885 | 0,00457145 | 0,03542024 |
| NANOZOOG7030 | 21,5246938 | -1,298189 | 0,39937912 | 0,00016695 | 0,00365949 |
| NANOZOOG2267 | 5,14465096 | 1,29754877 | 0,68149224 | 0,00466454 | 0,03594034 |
| NANOZOOG5359 | 36,0088705 | 1,29546062 | 0,40973147 | 0,00015619 | 0,00350632 |
| NANOZOOG5043 | 16,0286117 | -1,2950288 | 0,5590575 | 0,00225375 | 0,02186873 |
| NANOZOOG8833 | 53,4867909 | -1,2932421 | 0,71897901 | 0,0049778 | 0,03753257 |
| NANOZOOG6754 | 28,5169009 | 1,29293553 | 0,26668149 | 1,631E-07 | 2,0526E-05 |
| NANOZOOG5410 | 10,7273528 | 1,29203165 | 0,52215799 | 0,00130914 | 0,01501616 |
| NANOZOOG51 | 6,80758665 | -1,2882237 | 0,69323632 | 0,00570798 | 0,04115493 |
| NANOZOOG2990 | 33,822494 | -1,2857232 | 0,60780504 | 0,0029223 | 0,02617478 |
| NANOZOOG3512 | 13,5045079 | 1,28530768 | 0,38876899 | 0,00011193 | 0,00280022 |
| NANOZOOG7023 | 13,5799338 | 1,28492598 | 0,37828631 | 8,1478E-05 | 0,00217594 |
| NANOZOOG9326 | 19,689179 | -1,2846002 | 0,36705289 | 6,0604E-05 | 0,00186437 |
| NANOZOOG5512 | 33,0293769 | 1,28216962 | 0,27909566 | 5,84E-07 | 5,1603E-05 |
| NANOZOOG2914 | 15,8781956 | 1,28172105 | 0,40273567 | 0,00018609 | 0,00386419 |
| NANOZOOG6078 | 15,7059958 | 1,2800613 | 0,40474453 | 0,00018482 | 0,00386419 |
| NANOZOOG5634 | 13,6211355 | 1,27906805 | 0,46131376 | 0,00060701 | 0,00872287 |
| NANOZOOG2515 | 4,96310018 | -1,2784786 | 0,71599373 | 0,00741832 | 0,04901115 |
| NANOZOOG5523 | 17,9031373 | 1,27788023 | 0,38959852 | 0,00013038 | 0,00316646 |
| NANOZOOG2447 | 10,823816 | -1,2774481 | 0,55990172 | 0,00272065 | 0,02494228 |
| NANOZOOG206 | 88,4882664 | -1,2679205 | 0,44123438 | 0,00043963 | 0,00681256 |
| NANOZOOG5094 | 20,7139753 | 1,26558908 | 0,35645153 | 4,6487E-05 | 0,00161019 |
| NANOZOOG7114 | 14,7355033 | 1,26010601 | 0,36492323 | 6,9615E-05 | 0,00197577 |
| NANOZOOG7736 | 7,7140137 | -1,2587116 | 0,58629378 | 0,00401308 | 0,03248795 |
| NANOZOOG3861 | 42,2750459 | -1,2581483 | 0,38687016 | 0,00014503 | 0,00336492 |
| NANOZOOG1224 | 37,7245723 | -1,2541268 | 0,32933948 | 1,8721E-05 | 0,00083601 |
| NANOZOOG6087 | 28,2289301 | 1,25327581 | 0,33563569 | 2,3789E-05 | 0,00100812 |
| NANOZOOG2923 | 19,9575495 | -1,2475316 | 0,4981753 | 0,00150406 | 0,01661268 |
| NANOZOOG3420 | 12,2539018 | 1,24594827 | 0,42600027 | 0,00039435 | 0,00637245 |
| NANOZOOG2142 | 6,22993027 | -1,2445308 | 0,6179874 | 0,00492618 | 0,03740118 |
| NANOZOOG3329 | 64,4655368 | -1,2437871 | 0,34476593 | 4,6308E-05 | 0,00161019 |
| NANOZOOG6101 | 22,5799359 | 1,24209888 | 0,42671415 | 0,00039168 | 0,00637245 |
| NANOZOOG42 | 13,8290104 | 1,23826623 | 0,4542235 | 0,00077655 | 0,01036976 |
| NANOZOOG3176 | 20,9260153 | -1,2372423 | 0,4340416 | 0,00057347 | 0,00829837 |
| NANOZOOG7443 | 20,8187836 | -1,2364008 | 0,48734349 | 0,00134119 | 0,01517697 |
| NANOZOOG8476 | 15,9556605 | 1,23088709 | 0,36903102 | 0,00010685 | 0,00270584 |
| NANOZOOG9151 | 24,6448209 | -1,2280736 | 0,68118538 | 0,00585343 | 0,04188805 |
| NANOZOOG2000 | 25,825969 | -1,2262063 | 0,47843205 | 0,00131613 | 0,01501616 |
| NANOZOOG3509 | 35,5787406 | -1,2253025 | 0,39997156 | 0,00029181 | 0,00522369 |
| NANOZOOG1497 | 15,0282588 | 1,22502049 | 0,38058021 | 0,00015735 | 0,00351327 |
| NANOZOOG6769 | 12,6416303 | 1,22432931 | 0,52801203 | 0,00208907 | 0,02078036 |
| NANOZOOG9304 | 21,7170717 | 1,22294973 | 0,37967457 | 0,00015451 | 0,00348738 |
| NANOZOOG2793 | 33,532351 | 1,22110722 | 0,31626598 | 1,457E-05 | 0,00070307 |
| NANOZOOG2408 | 17,0090627 | 1,22088201 | 0,40646121 | 0,00032082 | 0,00561031 |
| NANOZOOG7860 | 28,3103535 | 1,21860247 | 0,28367926 | 2,2833E-06 | 0,00015727 |
| NANOZOOG9058 | 10,7662841 | -1,2165877 | 0,58981854 | 0,00379152 | 0,0314295 |
| NANOZOOG4345 | 13,24143 | -1,2164255 | 0,36727513 | 0,00013121 | 0,00316812 |
| NANOZOOG2592 | 17,5096109 | 1,21313129 | 0,51612598 | 0,00201532 | 0,02041375 |
| NANOZOOG6263 | 6,61532048 | 1,21263856 | 0,57561073 | 0,00377186 | 0,03133637 |
| NANOZOOG6438 | 15,7934889 | 1,21103039 | 0,33868836 | 4,9833E-05 | 0,00165061 |
| NANOZOOG7798 | 27,2872069 | 1,20877194 | 0,31928011 | 2,1285E-05 | 0,0009208 |
| NANOZOOG5376 | 9,91700145 | 1,20565259 | 0,60248901 | 0,0045261 | 0,0352446 |
| NANOZOOG7317 | 36,1394657 | -1,2051941 | 0,50144965 | 0,00177241 | 0,01863077 |
| NANOZOOG5006 | 20,3511365 | 1,20317779 | 0,33652674 | 5,0079E-05 | 0,00165061 |
| NANOZOOG7646 | 15,7830202 | 1,20178636 | 0,37182487 | 0,0001444 | 0,00336492 |
| NANOZOOG2810 | 12,409676 | -1,2000273 | 0,56183397 | 0,00324844 | 0,02846159 |
| NANOZOOG7567 | 18,1959514 | -1,1984185 | 0,57923963 | 0,00412969 | 0,03310929 |
| NANOZOOG4062 | 10,2735839 | -1,1976332 | 0,50538602 | 0,00208278 | 0,02078036 |
| NANOZOOG9204 | 16,76218 | 1,19589035 | 0,36006836 | 0,00012386 | 0,00306177 |
| NANOZOOG2937 | 93,0977843 | -1,1946677 | 0,40220078 | 0,00036877 | 0,00615053 |
| NANOZOOG1658 | 12,9207217 | 1,18638257 | 0,41182157 | 0,0005461 | 0,00798574 |
| NANOZOOG7641 | 21,2907329 | 1,18619425 | 0,45905231 | 0,00106951 | 0,01291185 |
| NANOZOOG2148 | 30,3270722 | 1,18425814 | 0,393166 | 0,00032092 | 0,00561031 |
| NANOZOOG416 | 15,2851156 | 1,18413461 | 0,48142941 | 0,00157568 | 0,01726594 |
| NANOZOOG3843 | 24,304072 | -1,1823876 | 0,43987794 | 0,00096418 | 0,0120247 |
| NANOZOOG7457 | 18,3541831 | 1,18217261 | 0,62937815 | 0,00559348 | 0,0407539 |
| NANOZOOG8027 | 7,69231927 | 1,18055224 | 0,56116141 | 0,00364208 | 0,03065996 |
| NANOZOOG3273 | 18,3403404 | 1,18041256 | 0,39996868 | 0,0003902 | 0,00637245 |
| NANOZOOG3736 | 9,95615956 | 1,17992366 | 0,42371201 | 0,00068746 | 0,00954854 |
| NANOZOOG6400 | 25,1299151 | -1,1778391 | 0,37213741 | 0,00022164 | 0,00430925 |
| NANOZOOG2070 | 14,499067 | 1,17596405 | 0,36354603 | 0,00017102 | 0,00371866 |
| NANOZOOG4544 | 15,9566362 | 1,17493578 | 0,34423877 | 9,796E-05 | 0,00251128 |
| NANOZOOG5397 | 8,50182962 | -1,1675953 | 0,60681488 | 0,00601904 | 0,0426246 |
| NANOZOOG1527 | 10,586291 | 1,16621212 | 0,53647665 | 0,00308054 | 0,02727635 |
| NANOZOOG3284 | 36,9930721 | 1,16579268 | 0,31102464 | 2,4525E-05 | 0,00102882 |
| NANOZOOG7886 | 7,58854481 | -1,1657104 | 0,60480173 | 0,00602471 | 0,0426246 |
| NANOZOOG7933 | 10,8363518 | 1,16040922 | 0,46858616 | 0,0016419 | 0,01776477 |
| NANOZOOG3546 | 17,6210319 | 1,15949998 | 0,32190172 | 4,4718E-05 | 0,00161019 |
| NANOZOOG344 | 23,6435812 | 1,15928713 | 0,39353876 | 0,00042424 | 0,00664855 |
| NANOZOOG2625 | 6,50684602 | 1,15683455 | 0,59626742 | 0,00544395 | 0,03994472 |
| NANOZOOG3333 | 12,5076137 | 1,15657821 | 0,42300258 | 0,00081788 | 0,01068047 |
| NANOZOOG5857 | 95,1336634 | -1,1563292 | 0,63140418 | 0,00587734 | 0,04188805 |
| NANOZOOG4831 | 15,2872271 | 1,15327758 | 0,35893038 | 0,0001791 | 0,00386297 |
| NANOZOOG2170 | 9,80444094 | 1,15177473 | 0,52942969 | 0,00339699 | 0,0292691 |
| NANOZOOG4749 | 13,5155972 | 1,15031339 | 0,41365533 | 0,00072569 | 0,00997945 |
| NANOZOOG5598 | 18,1463456 | 1,14755863 | 0,36238807 | 0,00020959 | 0,00420494 |
| NANOZOOG4063 | 10,374626 | -1,1464872 | 0,47231012 | 0,00207612 | 0,02078036 |
| NANOZOOG6225 | 12,2587265 | -1,145062 | 0,49657426 | 0,00269378 | 0,02480543 |
| NANOZOOG1657 | 34,3389656 | -1,1449185 | 0,38394861 | 0,00041349 | 0,00655425 |
| NANOZOOG5541 | 39,396568 | -1,1409703 | 0,32906817 | 8,253E-05 | 0,00218311 |
| NANOZOOG5583 | 24,0544357 | -1,1398608 | 0,35850259 | 0,00022166 | 0,00430925 |
| NANOZOOG76 | 43,9178476 | 1,13692213 | 0,40783363 | 0,00067306 | 0,00944328 |
| NANOZOOG6722 | 25,1003389 | -1,1352758 | 0,33923427 | 0,0001262 | 0,00310132 |
| NANOZOOG6604 | 16,5735653 | 1,13247833 | 0,41465049 | 0,00084138 | 0,01081806 |
| NANOZOOG7700 | 10,6602499 | 1,13230618 | 0,50598193 | 0,00285468 | 0,02577279 |
| NANOZOOG7771 | 36,0430477 | 1,12997641 | 0,25011436 | 1,1108E-06 | 9,0451E-05 |
| NANOZOOG2875 | 16,1045019 | 1,12936531 | 0,36053943 | 0,0002428 | 0,00464134 |
| NANOZOOG5188 | 11,2682227 | 1,12689231 | 0,40869814 | 0,00085708 | 0,0109521 |
| NANOZOOG8429 | 9,53423079 | 1,12683137 | 0,53413627 | 0,00430325 | 0,03384734 |
| NANOZOOG180 | 15,3716865 | 1,1236318 | 0,35901849 | 0,00026437 | 0,0048366 |
| NANOZOOG8824 | 12,4881758 | 1,12361867 | 0,42938653 | 0,00120354 | 0,01404015 |
| NANOZOOG5875 | 19,9730013 | 1,12208182 | 0,38712628 | 0,00049392 | 0,00737856 |
| NANOZOOG4977 | 9,47251228 | -1,1214327 | 0,5643768 | 0,00574496 | 0,04134976 |
| NANOZOOG6068 | 13,9198446 | 1,1212124 | 0,38281889 | 0,00048323 | 0,00727126 |
| NANOZOOG56 | 12,6994121 | -1,1181608 | 0,49154811 | 0,00286525 | 0,02581211 |
| NANOZOOG2426 | 13,1798643 | 1,11757842 | 0,50985126 | 0,00336887 | 0,02919732 |
| NANOZOOG3544 | 13,2784558 | -1,1167605 | 0,47471898 | 0,0024835 | 0,0233877 |
| NANOZOOG1537 | 14,3519569 | -1,1125428 | 0,47555043 | 0,00251238 | 0,02349976 |
| NANOZOOG8708 | 26,6102316 | 1,11112343 | 0,33852806 | 0,00014468 | 0,00336492 |
| NANOZOOG6048 | 15,2614072 | 1,11016125 | 0,36028923 | 0,00029634 | 0,00528206 |
| NANOZOOG7135 | 7,15960992 | 1,10738741 | 0,52979691 | 0,00460262 | 0,03559533 |
| NANOZOOG7637 | 15,9676488 | 1,10700692 | 0,39599833 | 0,00078458 | 0,01042925 |
| NANOZOOG6885 | 14,8654606 | 1,10673087 | 0,4927012 | 0,00284493 | 0,02574075 |
| NANOZOOG3697 | 22,3578392 | 1,1059164 | 0,35444473 | 0,00025651 | 0,00479854 |
| NANOZOOG946 | 23,3247261 | 1,10508513 | 0,30829753 | 5,5334E-05 | 0,00177465 |
| NANOZOOG5521 | 15,7030792 | 1,1007785 | 0,47368289 | 0,00258631 | 0,02408284 |
| NANOZOOG4031 | 39,2449135 | 1,10047757 | 0,53151082 | 0,00421695 | 0,03348562 |
| NANOZOOG2808 | 7,21173339 | 1,09795033 | 0,56971583 | 0,00630237 | 0,0436957 |
| NANOZOOG4848 | 41,0205573 | -1,0977683 | 0,39567011 | 0,00083127 | 0,01078831 |
| NANOZOOG1679 | 10,4141791 | 1,09228443 | 0,44196689 | 0,00188048 | 0,01942693 |
| NANOZOOG7044 | 19,3313513 | -1,0919189 | 0,44409964 | 0,00209155 | 0,02078036 |
| NANOZOOG7909 | 10,1145384 | 1,09112788 | 0,44869705 | 0,00208762 | 0,02078036 |
| NANOZOOG8819 | 15,2090709 | 1,08918599 | 0,33198942 | 0,00016342 | 0,00360997 |
| NANOZOOG7597 | 16,461477 | 1,08885778 | 0,39244928 | 0,00080252 | 0,01061427 |
| NANOZOOG6582 | 14,5094706 | 1,08696122 | 0,4504362 | 0,00211781 | 0,02099106 |
| NANOZOOG1668 | 19,0581611 | 1,08434773 | 0,39987037 | 0,00093468 | 0,01176283 |
| NANOZOOG9284 | 13,2267324 | 1,08426052 | 0,42759401 | 0,00144486 | 0,01604413 |
| NANOZOOG6157 | 10,8103035 | 1,08153219 | 0,43123855 | 0,00171802 | 0,01834176 |
| NANOZOOG2274 | 29,2329365 | 1,08124495 | 0,374118 | 0,00055043 | 0,00802078 |
| NANOZOOG3551 | 10,6018145 | 1,08058624 | 0,40501629 | 0,00111464 | 0,01334037 |
| NANOZOOG8965 | 12,0813439 | 1,07909525 | 0,46812054 | 0,00292442 | 0,02617478 |
| NANOZOOG6108 | 187,461267 | -1,0790672 | 0,45826293 | 0,00234392 | 0,02251386 |
| NANOZOOG5604 | 22,514905 | -1,0752003 | 0,40970985 | 0,00119214 | 0,01394637 |
| NANOZOOG994 | 62,3213066 | -1,0727034 | 0,35505334 | 0,00040553 | 0,0064776 |
| NANOZOOG4415 | 27,8989852 | 1,06943856 | 0,30263319 | 6,6505E-05 | 0,00197282 |
| NANOZOOG4949 | 49,6504217 | -1,0679045 | 0,29219235 | 4,8657E-05 | 0,00164518 |
| NANOZOOG2410 | 28,3412612 | 1,06563106 | 0,43183776 | 0,00174787 | 0,01851756 |
| NANOZOOG5222 | 20,2039168 | 1,06537031 | 0,31759521 | 0,0001281 | 0,00312947 |
| NANOZOOG1442 | 17,881952 | 1,06406405 | 0,34615158 | 0,00033333 | 0,0057441 |
| NANOZOOG3186 | 31,9387033 | 1,06398902 | 0,31281374 | 0,0001115 | 0,00280022 |
| NANOZOOG7904 | 1697,36501 | -1,0624669 | 0,30059401 | 6,171E-05 | 0,00188442 |
| NANOZOOG3524 | 19,4441193 | -1,0605043 | 0,33061618 | 0,00022204 | 0,00430925 |
| NANOZOOG8253 | 25,9803008 | 1,06046557 | 0,30029677 | 6,4494E-05 | 0,00194088 |
| NANOZOOG8920 | 26,5641306 | 1,06006717 | 0,33429202 | 0,00024506 | 0,00464134 |
| NANOZOOG5093 | 27,507704 | -1,0576105 | 0,29781581 | 6,7044E-05 | 0,00197471 |
| NANOZOOG3910 | 12,3573523 | 1,05727206 | 0,38481287 | 0,00091147 | 0,01150558 |
| NANOZOOG554 | 11,2026226 | 1,05527451 | 0,53805997 | 0,00586677 | 0,04188805 |
| NANOZOOG2454 | 26,2667229 | 1,05422054 | 0,38497925 | 0,00090858 | 0,01150401 |
| NANOZOOG1231 | 34,3714776 | 1,05185564 | 0,34514604 | 0,00034344 | 0,00583278 |
| NANOZOOG2705 | 7,12177545 | 1,04789274 | 0,51682577 | 0,00565175 | 0,04092604 |
| NANOZOOG2526 | 25,8779991 | 1,04615126 | 0,34647218 | 0,00041984 | 0,0066296 |
| NANOZOOG1584 | 18,1184628 | 1,04535968 | 0,3445451 | 0,0003835 | 0,00634535 |
| NANOZOOG8849 | 9,26372239 | 1,04424037 | 0,45800617 | 0,00317957 | 0,02803559 |
| NANOZOOG2503 | 29,3830145 | 1,04187387 | 0,28945679 | 5,3592E-05 | 0,00175249 |
| NANOZOOG7361 | 23,1221892 | -1,0401277 | 0,33590062 | 0,00034448 | 0,00583278 |
| NANOZOOG1384 | 16,5243994 | -1,0396647 | 0,47747889 | 0,00390122 | 0,03207882 |
| NANOZOOG5711 | 14,260105 | -1,0389451 | 0,37504678 | 0,00098596 | 0,01207874 |
| NANOZOOG8243 | 63,5639123 | 1,02972187 | 0,27926033 | 3,9099E-05 | 0,00150348 |
| NANOZOOG3118 | 39,5225439 | 1,02816573 | 0,33601287 | 0,0003455 | 0,00583278 |
| NANOZOOG319 | 10,9611787 | 1,02808465 | 0,39823913 | 0,00159514 | 0,01743317 |
| NANOZOOG3174 | 21,5430635 | 1,02668357 | 0,31992654 | 0,00022546 | 0,00435501 |
| NANOZOOG3811 | 22,0128419 | -1,0246534 | 0,51060539 | 0,00594691 | 0,04221799 |
| NANOZOOG2522 | 10,116942 | 1,02437121 | 0,45412763 | 0,00330496 | 0,0287144 |
| NANOZOOG8484 | 58,6979507 | -1,0204985 | 0,28880309 | 7,1787E-05 | 0,00200088 |
| NANOZOOG7220 | 3551,93305 | -1,0203984 | 0,26490606 | 2,0498E-05 | 0,00089608 |
| NANOZOOG1570 | 27,6216752 | -1,0186423 | 0,32409567 | 0,00028638 | 0,00514862 |
| NANOZOOG3520 | 12,3635957 | -1,0163182 | 0,51574592 | 0,00678074 | 0,04586385 |
| NANOZOOG2524 | 15,9766271 | -1,0120912 | 0,50024661 | 0,00613218 | 0,04301852 |
| NANOZOOG6990 | 50,2147725 | -1,0119453 | 0,37697696 | 0,00121649 | 0,0141515 |
| NANOZOOG9310 | 12,9757932 | 1,01175735 | 0,35906567 | 0,00082039 | 0,01068047 |
| NANOZOOG2860 | 14,3470338 | -1,0083798 | 0,4639672 | 0,00468263 | 0,0360129 |
| NANOZOOG7356 | 12,0199041 | 1,00643899 | 0,39679311 | 0,00171489 | 0,01834176 |
| NANOZOOG4343 | 11,7510895 | 1,00557267 | 0,40580257 | 0,00206916 | 0,02078036 |
| NANOZOOG7272 | 20,5353342 | -1,0030559 | 0,37406466 | 0,00124927 | 0,014453 |
| NANOZOOG2972 | 18,2977989 | -1,0016518 | 0,46618153 | 0,00495173 | 0,03752529 |
| NANOZOOG8322 | 11,4168764 | 0,99916089 | 0,47996113 | 0,00538079 | 0,0395512 |
| NANOZOOG6131 | 14,3167288 | -0,9976866 | 0,48862976 | 0,00650203 | 0,04455927 |
| NANOZOOG2751 | 25,6099248 | -0,9959362 | 0,36156806 | 0,00095234 | 0,01191288 |
| NANOZOOG2334 | 46,0146735 | -0,9951415 | 0,35328546 | 0,00081161 | 0,0106665 |
| NANOZOOG6835 | 28,1398287 | 0,99332859 | 0,27381372 | 5,5979E-05 | 0,00177465 |
| NANOZOOG1702 | 22,7420905 | 0,99225964 | 0,29357274 | 0,00014052 | 0,00333485 |
| NANOZOOG2991 | 1180,24534 | -0,9898258 | 0,3295502 | 0,00044711 | 0,00687722 |
| NANOZOOG5019 | 24,8251059 | 0,98947509 | 0,33832878 | 0,0005758 | 0,00830316 |
| NANOZOOG8823 | 16,2512312 | 0,9884093 | 0,41739173 | 0,00339508 | 0,0292691 |
| NANOZOOG4103 | 19,6187156 | -0,986793 | 0,38153675 | 0,00173296 | 0,0184538 |
| NANOZOOG7509 | 20,6334864 | 0,98499019 | 0,31912902 | 0,00034541 | 0,00583278 |
| NANOZOOG866 | 17,2266254 | 0,98236655 | 0,39608218 | 0,00221729 | 0,02171789 |
| NANOZOOG1619 | 19,0929665 | 0,97927514 | 0,32802912 | 0,00052111 | 0,00772913 |
| NANOZOOG5262 | 14,7998519 | 0,97891737 | 0,38871707 | 0,001831 | 0,01905799 |
| NANOZOOG3139 | 14,7074182 | 0,97848009 | 0,42600768 | 0,00363368 | 0,03065996 |
| NANOZOOG7811 | 19,0225576 | -0,9784015 | 0,46832748 | 0,00554686 | 0,04055655 |
| NANOZOOG8014 | 18,6241582 | 0,97815419 | 0,45557551 | 0,00489934 | 0,03726552 |
| NANOZOOG2608 | 18,6843089 | 0,97741992 | 0,29632018 | 0,00018045 | 0,00386297 |
| NANOZOOG8281 | 39,7672653 | -0,9727305 | 0,3100987 | 0,00032152 | 0,00561031 |
| NANOZOOG1886 | 8,7466158 | 0,9696593 | 0,42210249 | 0,00371022 | 0,0310031 |
| NANOZOOG5627 | 75,8123685 | -0,9689993 | 0,39820843 | 0,00247227 | 0,02333483 |
| NANOZOOG6586 | 17,2424796 | 0,96748142 | 0,47981177 | 0,00659044 | 0,04503287 |
| NANOZOOG299 | 21,1897823 | -0,966701 | 0,35564738 | 0,00125285 | 0,014453 |
| NANOZOOG4854 | 11,7198013 | 0,96478671 | 0,41105333 | 0,0030869 | 0,02727635 |
| NANOZOOG7895 | 13,0451286 | 0,96375186 | 0,35783486 | 0,00138769 | 0,01557589 |
| NANOZOOG2953 | 17,2232804 | -0,9614583 | 0,37390707 | 0,00190439 | 0,01962515 |
| NANOZOOG9312 | 19,9292919 | 0,95754073 | 0,39382691 | 0,00249403 | 0,02343368 |
| NANOZOOG6298 | 30,0925843 | -0,9546335 | 0,33128372 | 0,00076266 | 0,01028355 |
| NANOZOOG9001 | 14,8622587 | -0,9486212 | 0,42822724 | 0,00484536 | 0,03693942 |
| NANOZOOG7306 | 31,5417813 | 0,94729364 | 0,2672374 | 7,8708E-05 | 0,00213643 |
| NANOZOOG3589 | 8,65990845 | 0,93978096 | 0,45428839 | 0,00639165 | 0,04409388 |
| NANOZOOG7832 | 23,9036815 | -0,93476 | 0,41334503 | 0,00419063 | 0,03334038 |
| NANOZOOG4787 | 342,78408 | -0,9332557 | 0,35785484 | 0,00157218 | 0,01726594 |
| NANOZOOG7760 | 15,7436501 | -0,9215907 | 0,37051131 | 0,00261576 | 0,02430259 |
| NANOZOOG1895 | 154,815101 | -0,9197324 | 0,42855227 | 0,00557092 | 0,04066089 |
| NANOZOOG1495 | 30,5766311 | -0,9196549 | 0,29070195 | 0,00034209 | 0,00583278 |
| NANOZOOG2903 | 12,40865 | -0,9144157 | 0,42597738 | 0,00561814 | 0,04079042 |
| NANOZOOG8739 | 21,3710524 | -0,9108718 | 0,41044342 | 0,00509684 | 0,03793399 |
| NANOZOOG2702 | 10,2813408 | 0,91060609 | 0,44552694 | 0,00742307 | 0,04901115 |
| NANOZOOG1223 | 11,7627324 | -0,9080308 | 0,40870784 | 0,0049606 | 0,03752529 |
| NANOZOOG3327 | 107,000692 | -0,9054824 | 0,31128584 | 0,00078602 | 0,01042925 |
| NANOZOOG3400 | 29,1425395 | 0,90291835 | 0,31928509 | 0,00098462 | 0,01207874 |
| NANOZOOG7038 | 23,8969797 | -0,8989593 | 0,40069394 | 0,00484758 | 0,03693942 |
| NANOZOOG97 | 9,26019934 | 0,89616847 | 0,42978523 | 0,00665378 | 0,04522611 |
| NANOZOOG8930 | 34,2414414 | 0,89419799 | 0,39891183 | 0,00442939 | 0,03464265 |
| NANOZOOG8424 | 10,9323171 | 0,89215974 | 0,41255919 | 0,00588026 | 0,04188805 |
| NANOZOOG5113 | 30,0981007 | 0,88946166 | 0,38791721 | 0,00410158 | 0,03301135 |
| NANOZOOG754 | 29,5688779 | 0,88714014 | 0,32791281 | 0,00140099 | 0,01568275 |
| NANOZOOG2771 | 25,3735156 | 0,88678164 | 0,29748957 | 0,00061517 | 0,00880967 |
| NANOZOOG236 | 12,6939253 | 0,88369225 | 0,419966 | 0,00637409 | 0,04404592 |
| NANOZOOG6327 | 27,5378976 | -0,8825925 | 0,36175118 | 0,00301422 | 0,02680526 |
| NANOZOOG8020 | 17,3363489 | 0,88019379 | 0,39382782 | 0,00461458 | 0,03562151 |
| NANOZOOG5389 | 12,3180692 | 0,87923098 | 0,41686745 | 0,00625437 | 0,04365543 |
| NANOZOOG4989 | 17,780838 | 0,87866592 | 0,39776472 | 0,00508823 | 0,03793399 |
| NANOZOOG5367 | 28,1234114 | 0,87649769 | 0,33469621 | 0,00180635 | 0,01889615 |
| NANOZOOG7339 | 24,0663982 | 0,8761575 | 0,3354906 | 0,00186008 | 0,01926413 |
| NANOZOOG3877 | 29,4374549 | 0,87548018 | 0,26672223 | 0,00021595 | 0,00429115 |
| NANOZOOG1953 | 25,595749 | 0,87518534 | 0,30385664 | 0,00085432 | 0,01095055 |
| NANOZOOG2864 | 23,5713578 | 0,87498463 | 0,39156366 | 0,00483435 | 0,03693942 |
| NANOZOOG3754 | 21,4665871 | 0,87426894 | 0,33794746 | 0,00196264 | 0,02002665 |
| NANOZOOG568 | 412,600457 | -0,8731508 | 0,2776593 | 0,00035816 | 0,00602201 |
| NANOZOOG5818 | 17,7091509 | 0,87216145 | 0,38441155 | 0,00475309 | 0,03641989 |
| NANOZOOG6806 | 19,5132213 | 0,86904797 | 0,3544543 | 0,00289631 | 0,0260354 |
| NANOZOOG30 | 28,0404458 | -0,8688123 | 0,34693031 | 0,00273839 | 0,02504963 |
| NANOZOOG5076 | 11,3926077 | 0,86729181 | 0,41095568 | 0,00659282 | 0,04503287 |
| NANOZOOG198 | 22,2150782 | -0,8603515 | 0,31029445 | 0,00125152 | 0,014453 |
| NANOZOOG6153 | 17,9138544 | 0,85775562 | 0,3347854 | 0,00224862 | 0,02186873 |
| NANOZOOG3588 | 20,7110462 | 0,8548977 | 0,28750102 | 0,00068603 | 0,00954854 |
| NANOZOOG6889 | 34,0307984 | 0,85453798 | 0,28680548 | 0,00065515 | 0,00928607 |
| NANOZOOG5850 | 18,8280775 | -0,8527265 | 0,39268072 | 0,00607258 | 0,04281734 |
| NANOZOOG5767 | 19,96915 | 0,85193514 | 0,34302739 | 0,00282787 | 0,02564223 |
| NANOZOOG3029 | 16,6146525 | 0,85106137 | 0,36112471 | 0,00393212 | 0,03218793 |
| NANOZOOG3460 | 77,9679154 | -0,8446051 | 0,39724427 | 0,00661856 | 0,0450746 |
| NANOZOOG4318 | 421,699662 | -0,844016 | 0,2339764 | 7,6326E-05 | 0,00208542 |
| NANOZOOG6124 | 13,5021223 | 0,84059989 | 0,35359577 | 0,00396129 | 0,03222412 |
| NANOZOOG6449 | 26,5563875 | 0,83963425 | 0,32545844 | 0,00223635 | 0,02180179 |
| NANOZOOG235 | 18,6248422 | 0,83702525 | 0,38470401 | 0,00610225 | 0,0429288 |
| NANOZOOG5905 | 14,1373029 | 0,83531013 | 0,33334061 | 0,00277072 | 0,02523419 |
| NANOZOOG7218 | 1279,94332 | -0,8342768 | 0,19478999 | 4,8935E-06 | 0,00028226 |
| NANOZOOG8690 | 19,1372377 | 0,83383269 | 0,35560695 | 0,00423251 | 0,03354505 |
| NANOZOOG7560 | 61,3881227 | -0,8303923 | 0,39386918 | 0,00721965 | 0,04805002 |
| NANOZOOG1675 | 26,0433752 | 0,82811351 | 0,28158354 | 0,00081945 | 0,01068047 |
| NANOZOOG5644 | 21,9018519 | -0,8231613 | 0,34453759 | 0,00390147 | 0,03207882 |
| NANOZOOG2481 | 27284,5577 | -0,8177106 | 0,27710854 | 0,00075623 | 0,0102301 |
| NANOZOOG2950 | 18,315533 | 0,81672761 | 0,36008527 | 0,00503438 | 0,03773969 |
| NANOZOOG2817 | 17,2342287 | 0,81302202 | 0,35560085 | 0,00509024 | 0,03793399 |
| NANOZOOG6079 | 47,9067247 | 0,81300311 | 0,27520694 | 0,00084074 | 0,01081806 |
| NANOZOOG5242 | 18,3540647 | 0,80637263 | 0,30065962 | 0,00177649 | 0,01863077 |
| NANOZOOG7973 | 49,5229523 | -0,8052169 | 0,29665813 | 0,00166018 | 0,01788073 |
| NANOZOOG9252 | 25,4456035 | -0,801727 | 0,34833011 | 0,00533657 | 0,03936551 |
| NANOZOOG8260 | 21,5469474 | 0,80152766 | 0,32777449 | 0,0034998 | 0,02984531 |
| NANOZOOG8025 | 14,9178639 | 0,797943 | 0,34506187 | 0,00505698 | 0,03784082 |
| NANOZOOG2005 | 16,4018312 | 0,79501882 | 0,34726042 | 0,00545521 | 0,0399568 |
| NANOZOOG3819 | 28,2253116 | 0,79129677 | 0,28425348 | 0,00133143 | 0,01514909 |
| NANOZOOG4665 | 35,4751727 | 0,78756689 | 0,32235522 | 0,00364701 | 0,03065996 |
| NANOZOOG4993 | 15,0028236 | 0,78485675 | 0,35082768 | 0,00625593 | 0,04365543 |
| NANOZOOG2247 | 3282,50365 | -0,7825987 | 0,35918427 | 0,00672921 | 0,04566407 |
| NANOZOOG3950 | 19,6091246 | 0,77883166 | 0,32230143 | 0,00390847 | 0,03207882 |
| NANOZOOG6146 | 28,411867 | 0,77786098 | 0,27001057 | 0,00105275 | 0,0127466 |
| NANOZOOG8772 | 43,433399 | -0,776213 | 0,30558555 | 0,00296743 | 0,02650269 |
| NANOZOOG9463 | 26,9650327 | 0,77292984 | 0,25460636 | 0,00066328 | 0,00936939 |
| NANOZOOG1580 | 23,1763267 | 0,77090878 | 0,3001384 | 0,00269029 | 0,02480543 |
| NANOZOOG7065 | 41,5898197 | -0,769398 | 0,30179925 | 0,00297529 | 0,02651585 |
| NANOZOOG3920 | 16,9204746 | 0,76524073 | 0,32182252 | 0,00449796 | 0,03511284 |
| NANOZOOG5428 | 17,4927189 | 0,76401883 | 0,34239162 | 0,00629384 | 0,0436957 |
| NANOZOOG3936 | 19,1885874 | -0,7535357 | 0,33620244 | 0,00662064 | 0,0450746 |
| NANOZOOG122 | 1831,64942 | -0,7510118 | 0,23530012 | 0,00038344 | 0,00634535 |
| NANOZOOG176 | 20,4146948 | 0,75091229 | 0,30908683 | 0,00417281 | 0,03326236 |
| NANOZOOG8610 | 15,5815947 | 0,74695957 | 0,32880306 | 0,00622174 | 0,04357319 |
| NANOZOOG6423 | 30,3310807 | -0,742106 | 0,32245505 | 0,00610906 | 0,0429288 |
| NANOZOOG4098 | 24,7161536 | 0,73731121 | 0,26156624 | 0,0014284 | 0,01590389 |
| NANOZOOG8188 | 556,979637 | -0,7316875 | 0,32933716 | 0,00628625 | 0,0436957 |
| NANOZOOG7783 | 45,2494296 | 0,72909541 | 0,27050178 | 0,00201287 | 0,02041375 |
| NANOZOOG231 | 61,3815248 | -0,7285477 | 0,29432305 | 0,00395762 | 0,03222412 |
| NANOZOOG6420 | 22,5761621 | 0,72785763 | 0,32453349 | 0,0068128 | 0,04593112 |
| NANOZOOG6861 | 16,6850536 | 0,72277143 | 0,32070382 | 0,00690911 | 0,04643268 |
| NANOZOOG3295 | 19,2813084 | 0,72195327 | 0,31390802 | 0,00632056 | 0,04374882 |
| NANOZOOG7217 | 6717,66455 | -0,7115637 | 0,22684052 | 0,00053899 | 0,00790962 |
| NANOZOOG3144 | 29,4107246 | 0,70357897 | 0,27744194 | 0,00340721 | 0,02929639 |
| NANOZOOG1468 | 33,8904056 | 0,68817212 | 0,27050387 | 0,00347351 | 0,0296821 |
| NANOZOOG3079 | 27,6836782 | 0,68599178 | 0,30307884 | 0,00728864 | 0,04843156 |
| NANOZOOG4005 | 47,0384078 | 0,67712264 | 0,22830748 | 0,00101236 | 0,01229336 |
| NANOZOOG9047 | 21,9744332 | 0,66978207 | 0,26487661 | 0,00380833 | 0,03150598 |
| NANOZOOG4605 | 39,4867873 | 0,65937589 | 0,25949408 | 0,00377177 | 0,03133637 |
| NANOZOOG8689 | 32,0867617 | 0,65653182 | 0,27593888 | 0,00590495 | 0,04199188 |
| NANOZOOG7222 | 20001,2538 | -0,6430934 | 0,15287694 | 1,0191E-05 | 0,00054964 |
| NANOZOOG5223 | 24,3997245 | 0,62747377 | 0,24194366 | 0,00370356 | 0,0310031 |
| NANOZOOG6681 | 52,7199331 | -0,6197435 | 0,22358299 | 0,00220655 | 0,02166386 |
| NANOZOOG3230 | 30,8605481 | 0,59314495 | 0,22877137 | 0,00404962 | 0,03272 |
| NANOZOOG7226 | 5750,32271 | -0,5631557 | 0,21537973 | 0,00393727 | 0,03218793 |
| NANOZOOG7219 | 250825,212 | -0,556619 | 0,19875486 | 0,00231101 | 0,02226827 |
| NANOZOOG3132 | 4,06062439 | 0,46127113 | 0,87350348 | 0,00241067 | 0,02290967 |
| NANOZOOG6065 | 4,68587238 | -0,4581336 | 0,86056506 | 0,00690956 | 0,04643268 |

Mean of normalized counts, fold-change, fold-change standard error, and p-values in genes that were differentially expressed between the samples CGHH 36 h and CG 60 h. CGHH 36 h and CG 60 h samples belong to three independent cultivations in culture medium containing as carbon source either CGHH (a mixture of hemicellulose hydrolysate and crude glycerol) or CG (crude glycerol), respectively. The samples were collected at 36 h and 60 h cultivation, respectively. Data was obtained using RNA-Seq.

Table S4: Gene expression within central metabolic pathways

| **Gene name** | **Gene ID** | **CGHH-10h** | **CGHH-36h** | **CGHH-60h** | **CG-10h** | **CG-30h** | **CG-60h** |
| --- | --- | --- | --- | --- | --- | --- | --- |
| *lbsA* | NANOZOOG1991 | 347 | 132 | 76 | 141 | 108 | 56 |
| *NdufS7* | NANOZOOG6298 | 283 | 127 | 55 | 134 | 147 | 95 |
| *NdufA8* | NANOZOOG6990 | 766 | 199 | 121 | 327 | 191 | 144 |
| *nuo21.3c* | NANOZOOG7015 | 84 | 55 | 27 | 39 | 34 | 19 |
| *NDUFA1* | NANOZOOG7030 | 290 | 137 | 72 | 223 | 117 | 74 |
| *PRR36* | NANOZOOG2057 | 18 | 29 | 4 | 0 | 14 | 28 |
| *NDUFA6* | NANOZOOG4848 | 542 | 287 | 164 | 209 | 222 | 191 |
| *RIP1* | NANOZOOG1224 | 173 | 121 | 44 | 87 | 114 | 77 |
| *QCR7* | NANOZOOG3327 | 1845 | 539 | 390 | 1204 | 830 | 424 |
| *COX12* | NANOZOOG6045 | 1606 | 301 | 218 | 682 | 491 | 300 |
| *ATP16* | NANOZOOG7299 | 1775 | 481 | 169 | 561 | 546 | 237 |
| *ATP4* | NANOZOOG8484 | 500 | 209 | 99 | 288 | 262 | 149 |
| *ATP5* | NANOZOOG7743 | 376 | 152 | 82 | 223 | 168 | 123 |
| *ATP7* | NANOZOOG5110 | 540 | 133 | 54 | 149 | 93 | 61 |
| *ATP14* | NANOZOOG7884 | 326 | 98 | 29 | 167 | 84 | 50 |
| *PHB1* | NANOZOOG8333 | 243 | 84 | 64 | 92 | 78 | 83 |
| *PHB2* | NANOZOOG7096 | 69 | 69 | 36 | 44 | 40 | 35 |
| *PET9* | NANOZOOG1808 | 511 | 373 | 250 | 337 | 294 | 429 |
| *RIB3* | NANOZOOG9252 | 151 | 78 | 38 | 97 | 14 | 17 |
| *RIB4* | NANOZOOG2556 | 293 | 127 | 73 | 127 | 151 | 175 |
| *RIB5* | NANOZOOG2045 | 85 | 31 | 24 | 40 | 28 | 26 |
| *THI80* | NANOZOOG9040 | 98 | 12 | 2 | 15 | 2 | 8 |
| *LIP5* | NANOZOOG5711 | 63 | 52 | 39 | 31 | 26 | 35 |
| *CPR6* | NANOZOOG5522 | 72 | 18 | 15 | 23 | 38 | 47 |
| *IDP1* | NANOZOOG5845 | 46 | 19 | 13 | 19 | 19 | 30 |
| *IDH1* | NANOZOOG435 | 16 | 34 | 12 | 29 | 16 | 10 |
| *LSC2* | NANOZOOG7009 | 65 | 57 | 34 | 42 | 60 | 68 |
| *LSC1* | NANOZOOG528 | 11 | 14 | 8 | 11 | 11 | 8,86 |
| *AAEL001134* | NANOZOOG3041 | 36 | 5 | 12 | 1 | 10 | 39 |
| *ETR1* | NANOZOOG8822 | 76 | 16 | 23 | 32 | 22 | 19 |
| *GCDH* | NANOZOOG7323 | 114 | 4 | 8 | 7 | 8 | 11 |
| *YAT1* | NANOZOOG1185 | 60 | 3 | 4 | 18 | 3 | 8 |
| *MZM1* | NANOZOOG3643 | 111 | 47 | 18 | 34 | 25 | 31 |
| *Acadsb* | NANOZOOG5684 | 76 | 5 | 3 | 7 | 0 | 8 |
| *RHTO_00476* | NANOZOOG3149 | 20 | 17 | 22 | 8 | 13 | 31 |
| *SPAC806.04c* | NANOZOOG8109 | 71 | 41 | 34 | 38 | 47 | 46 |
| *AAT1* | NANOZOOG6123 | 27 | 10 | 4 | 41 | 18 | 2,80 |
| *PDB1* | NANOZOOG206 | 83 | 561 | 297 | 171 | 253 | 312 |
| *lat1* | NANOZOOG6289 | 6 | 41 | 17 | 25 | 43 | 11,59 |
| *PDA1* | NANOZOOG1243 | 10 | 86 | 42 | 54 | 65 | 48 |
| *ACL1* | NANOZOOG7175 | 2 | 170 | 64 | 44 | 81 | 42 |
| *ACS1* | NANOZOOG8257 | 103 | 9 | 10 | 56 | 24 | 4 |
| *ACC1* | NANOZOOG8968 | 8 | 88 | 30 | 28 | 48 | 38 |
| *OAR1* | NANOZOOG8056 | 363 | 9 | 5 | 11 | 6 | 12 |
| *etr1* | NANOZOOG8822 | 76 | 16 | 23 | 32 | 22 | 19 |
| *FAS1* | NANOZOOG8939 | 3 | 58 | 26 | 12 | 39 | 20 |
| *FAS2* | NANOZOOG6383 | 6 | 80 | 32 | 18 | 48 | 36 |
| *FAS21* | NANOZOOG6384 | 0 | 0 | 0 | 0 | 0 | 1 |
| *ACAD10* | NANOZOOG4755 | 153 | 8 | 15 | 29 | 23 | 11 |
| *Acadsb* | NANOZOOG5684 | 76 | 5 | 3 | 7 | 0 | 8 |
| *GCDH* | NANOZOOG7323 | 114 | 4 | 8 | 7 | 8 | 11 |
| *POT1* | NANOZOOG52 | 169 | 15 | 13 | 17 | 9 | 49 |
| *POT1* | NANOZOOG3816 | 38 | 3 | 8 | 6 | 5 | 5 |
| *Acaa2* | NANOZOOG3149 | 20 | 17 | 22 | 8 | 13 | 31 |
| *ACADM* | NANOZOOG1965 | 66 | 10 | 12 | 21 | 17 | 47 |
| *ACAD11* | NANOZOOG8232 | 176 | 4 | 7 | 10 | 6 | 16 |
| *ECI1* | NANOZOOG4877 | 700 | 32 | 20 | 39 | 27 | 31 |
| *FOX2* | NANOZOOG4031 | 67 | 19 | 15 | 20 | 18 | 87 |
| *HSD17B4* | NANOZOOG901 | 598 | 9 | 11 | 21 | 18 | 15 |
| *Acaa1a* | NANOZOOG2943 | 18 | 5 | 3 | 1 | 2 | 7,77 |
| *fadJ* | NANOZOOG5824 | 73 | 21 | 20 | 16 | 35 | 32,31 |
| *FAA1* | NANOZOOG2650 | 6 | 46 | 26 | 31 | 30 | 50 |
| *ZWF1* | NANOZOOG6681 | 79 | 134 | 79 | 115 | 139 | 126 |
| *GND1* | NANOZOOG3886 | 26 | 140 | 28 | 88 | 101 | 71 |
| *ME3* | NANOZOOG4381 | 8 | 10 | 4 | 5 | 12 | 11 |
| *MAE1* | NANOZOOG166 | 3 | 13 | 6 | 3 | 11 | 5 |
| *MAE1* | NANOZOOG167 | 4 | 6 | 9 | 0 | 3 | 8 |
| *STL1* | NANOZOOG7915 | 8 | 48 | 8 | 51 | 22 | 69 |
| *GUP1* | NANOZOOG4353 | 0 | 4 | 4 | 6 | 0 | 6 |
| *GUT1* | NANOZOOG4714 | 38 | 38 | 17 | 57 | 71 | 50 |
| *GUT2* | NANOZOOG5741 | 9 | 12 | 7 | 9 | 11 | 20 |
| *GCY1* | NANOZOOG4361 | 90 | 12 | 7 | 52 | 23 | 18 |
| *GCY1* | NANOZOOG370 | 32 | 15 | 2 | 0 | 4 | 17 |
| *DAK2* | NANOZOOG2321 | 3 | 3 | 1 | 4 | 12 | 10 |
| *RTG_01831* | NANOZOOG6637 | 14 | 13 | 13 | 6 | 12 | 6 |
| *TPI1* | NANOZOOG1080 | 205 | 98 | 46 | 70 | 97 | 47 |
| *TPIP1* | NANOZOOG7548 | 37 | 32 | 31 | 55 | 31 | 65 |
| *GDP1* | NANOZOOG1674 | 4 | 50 | 19 | 12 | 8 | 9,72 |
| *glkA* | NANOZOOG4916 | 17 | 60 | 45 | 46 | 45 | 64 |
| *HXK2* | NANOZOOG7316 | 12 | 27 | 16 | 44 | 15 | 27 |
| *yeaD* | NANOZOOG9194 | 61 | 26 | 10 | 34 | 6 | 20,74 |
| *PGI1* | NANOZOOG771 | 20 | 47 | 14 | 54 | 38 | 32 |
| *PFK2* | NANOZOOG3173 | 6 | 40 | 41 | 41 | 46 | 71 |
| *FBA1* | NANOZOOG3590 | 70 | 69 | 39 | 204 | 78 | 40 |
| *TPI1* | NANOZOOG1080 | 205 | 98 | 46 | 70 | 97 | 47 |
| *GPD* | NANOZOOG7990 | 58 | 215 | 52 | 222 | 118 | 54 |
| *Gapdhs* | NANOZOOG1838 | 1 | 2 | 1 | 3 | 3 | 3 |
| *Gapdhs* | NANOZOOG4829 | 6 | 10 | 5 | 1 | 13 | 15 |
| *aldA* | NANOZOOG8569 | 101 | 113 | 31 | 413 | 169 | 41,83 |
| *PGK1* | NANOZOOG2622 | 18 | 32 | 9 | 50 | 38 | 15 |
| *GPM1* | NANOZOOG6772 | 400 | 191 | 104 | 182 | 166 | 50 |
| *GPM1* | NANOZOOG2179 | 4 | 30 | 13 | 30 | 6 | 25 |
| *ENO1* | NANOZOOG2969 | 34 | 135 | 19 | 56 | 62 | 25 |
| *PYK1* | NANOZOOG4157 | 4 | 94 | 15 | 35 | 27 | 46 |
| *PCK1* | NANOZOOG4519 | 14 | 2 | 1 | 8 | 2 | 10,38 |
| *PYC1* | NANOZOOG9202 | 11 | 105 | 66 | 51 | 72 | 126,10 |
| *TRX3* | NANOZOOG3685 | 147 | 52 | 60 | 45 | 23 | 39 |
| *TRX1* | NANOZOOG7078 | 93 | 35 | 17 | 45 | 54 | 29 |
| *txl1* | NANOZOOG3176 | 143 | 69 | 57 | 57 | 63 | 41 |
| *CPR6* | NANOZOOG5522 | 72 | 18 | 15 | 23 | 38 | 47 |
| *GST* | NANOZOOG3574 | 62 | 24 | 7 | 20 | 13 | 11 |
| *gst2* | NANOZOOG4618 | 57 | 0 | 1 | 2 | 0 | 0 |
| *GSTU8* | NANOZOOG9349 | 33 | 8 | 3 | 40 | 0 | 12 |
| *GPX2* | NANOZOOG2851 | 24 | 20 | 9 | 12 | 5 | 8 |
| *SFA1* | NANOZOOG5726 | 86 | 16 | 30 | 50 | 35 | 47 |
| *cat-1* | NANOZOOG1031 | 0 | 3 | 0 | 29 | 2 | 16 |
| *npcC* | NANOZOOG2281 | 217 | 4 | 9 | 12 | 10 | 6 |
| *xylB* | NANOZOOG7746 | 50 | 11 | 11 | 9 | 2 | 15 |
| *nahG* | NANOZOOG4610 | 84 | 5 | 9 | 9 | 19 | 20 |
| *pho2* | NANOZOOG8643 | 104 | 37 | 51 | 37 | 32 | 47 |
| *GCDH* | NANOZOOG7323 | 114 | 4 | 8 | 7 | 8 | 11 |
| *Rhto_03963* | NANOZOOG7125 | 137 | 28 | 5 | 57 | 18 | 16,88 |
| *gar1* | NANOZOOG8868 | 126 | 2 | 5 | 25 | 5 | 5,43 |
| *SPAC2F3.05c* | NANOZOOG6189 | 4 | 6 | 2 | 4 | 0 | 1,47 |
| *SPAC2F3.05c* | NANOZOOG6194 | 0 | 1 | 1 | 0 | 0 | 2,14 |
| *SPAC2F3.05c* | NANOZOOG3299 | 18 | 3 | 0 | 25 | 0 | 0,00 |
| *XYL2* | NANOZOOG9007 | 105 | 4 | 7 | 16 | 19 | 12 |
| *RHTO_04556* | NANOZOOG9455 | 1 | 0 | 0 | 0 | 0 | 0,00 |
| *SPBC24C6.09c* | NANOZOOG7705 | 4 | 11 | 5 | 37 | 18 | 17 |
| *RPE1* | NANOZOOG6580 | 67 | 45 | 25 | 91 | 74 | 39,24 |
| *LAD1* | NANOZOOG4154 | 45 | 20 | 11 | 35 | 20 | 32 |
| *ARD1* | NANOZOOG6692 | 10 | 3 | 2 | 8 | 0 | 6,54 |
| *YDR109C* | NANOZOOG5356 | 15 | 7 | 8 | 8 | 11 | 14 |

Gene expression in TPM values from each of the samples CGHH 10 h, CGHH 36 h and CGHH 60 h belongs to the mean values between three independent cultivations in culture medium containing CGHH as carbon source, at 10 h, 36 h and 60 h, respectively. Gene expression from each of the samples CG 10 h, CG 30 h and CG 60 h belongs to mean TPM values between three independent cultivations in culture medium containing CG as carbon source, taken at 10 h, 30 h and 60 h, respectively.

Figure S1: Principal Component Analysis based on expression level from the 500 highest expressed genes in each of the cultivation media and RNA sampling points. CG, crude glycerol; CGHH, mixture of CG and hemicellulose hydrolysate


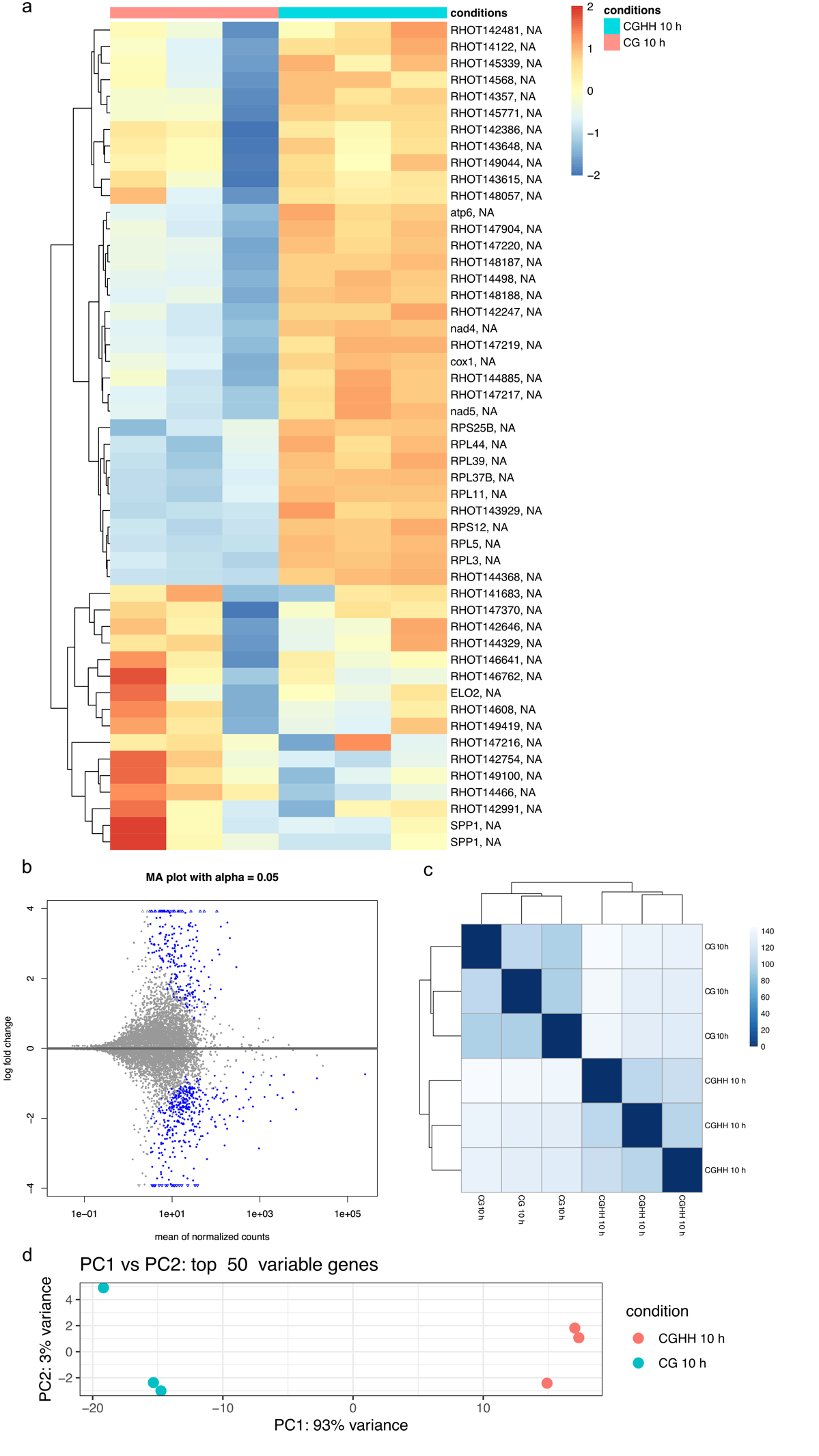


Figure S2: Overview of differential expression analysis between *Rhodotorula toruloides* CBS 14 cultivated on crude glycerol and a mixture of crude glycerol and hemicellulose hydrolysate, at 10 h cultivation. (A) Heatmap of the count matrix from the 50 genes whose expression varies the most, using variance stabilizing transformation and being row scaled. (B) MA-plot. Blue dots indicate significantly (adjusted p-value <0.05) differentially expressed genes, while gray dots indicate non-differentially expressed genes. (C) Heatmap of sample-to-sample distances on count matrix, which was variance-stabilized. Darker blue indicates a more similar expression level. (D) Principal Component Analysis (PCA) from the 50 genes whose expression varies the most, using variance stabilizing transformation. The samples CG 10 h belong to the three independent cultivations in culture medium containing CG as carbon source, respectively at 10 h. The samples CGHH 10 h belong to the three independent cultivations in culture medium containing CGHH as carbon source, respectively at 10 h.


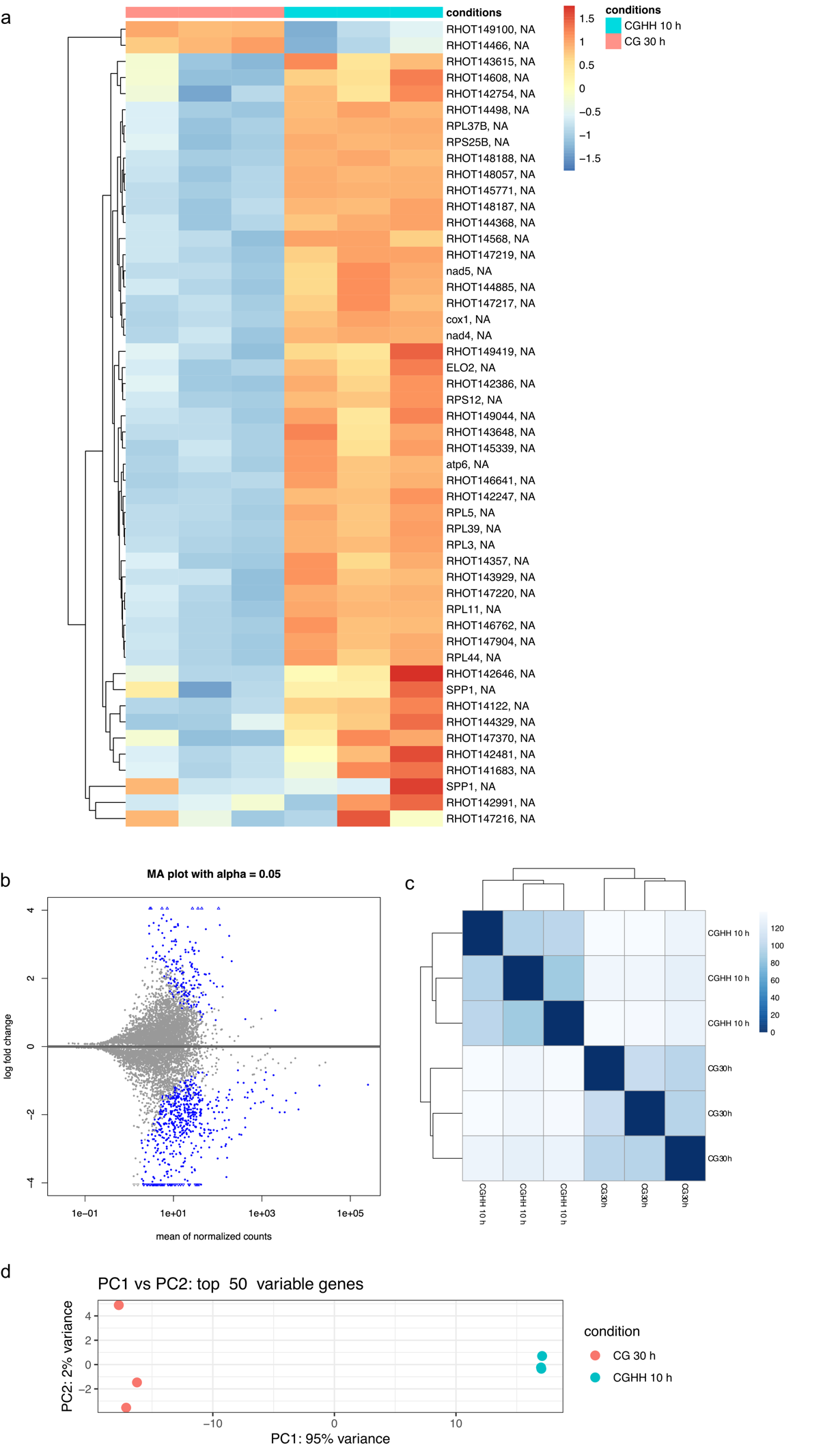


Figure S3: Overview of differential expression analysis between *Rhodotorula toruloides* CBS 14 grown on different media when glycerol consumption became visible: CG 30 h and CGHH 10 h cultivations. (A) Heatmap of the count matrix from the 50 genes whose expression varies the most, using variance stabilizing transformation and being row scaled. (B) MA-plot. Blue dots indicate significantly (adjusted p-value <0.05) differentially expressed genes, while gray dots indicate non-differentially expressed genes. (C) Heatmap of sample-to-sample distances on count matrix, which was variance-stabilized. Darker blue indicates a more similar expression level. (D) Principal Component Analysis (PCA) from the 50 genes whose expression varies the most, using variance stabilizing transformation. The samples CG 30 h belong to the three independent cultivations in culture medium containing CG as carbon source, respectively at 30 h. The samples CGHH 10 h belong to the three independent cultivations in culture medium containing CGHH as carbon source, respectively at 10 h.


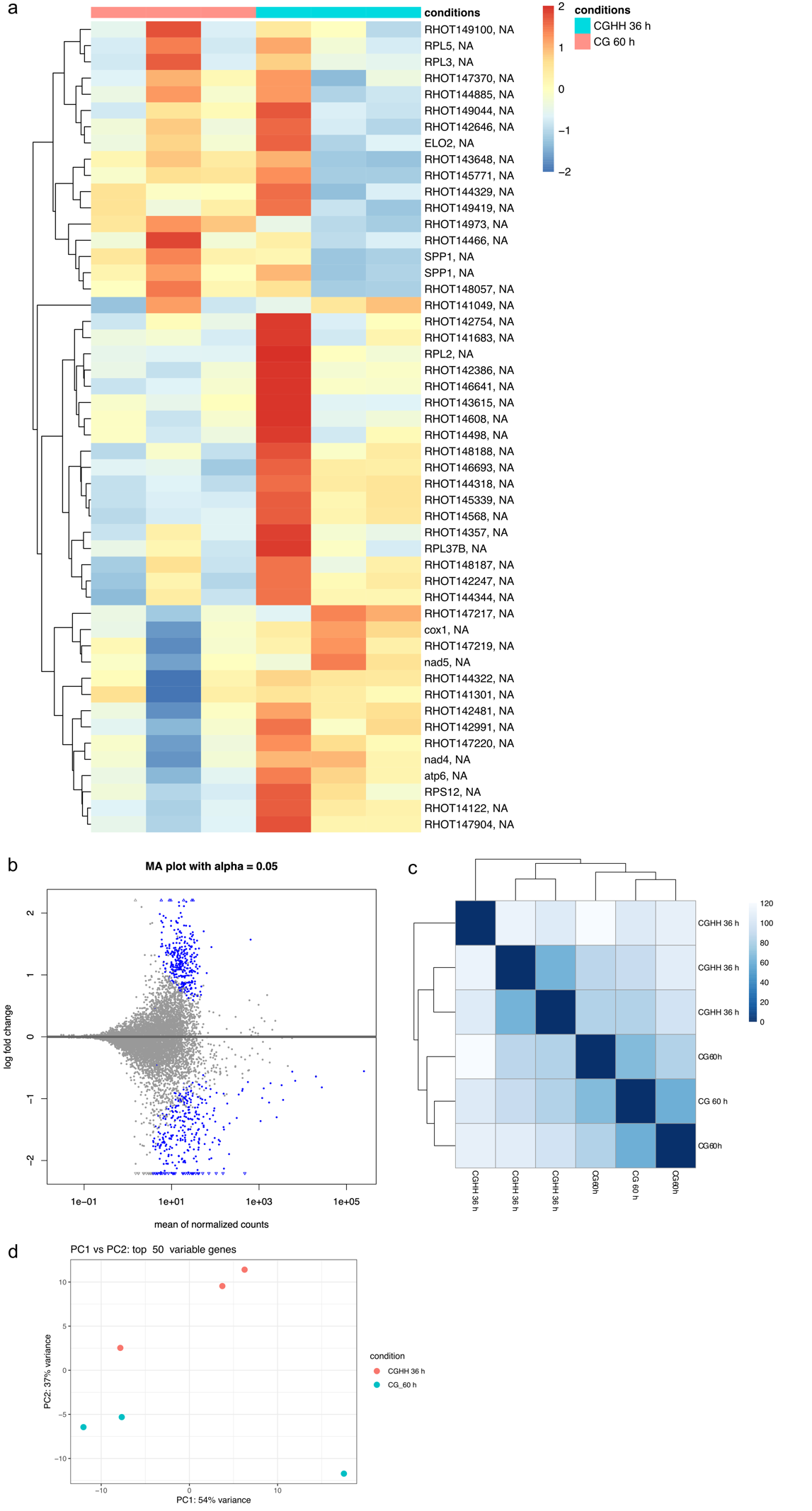


Figure S4: Overview of differential expression analysis between *Rhodotorula toruloides* CBS 14 grown on different media when about 20 g/l of glycerol were left: CG 60 h and CGHH 36 h cultivations. (A) Heatmap of the count matrix from the 50 genes whose expression varies the most, using variance stabilizing transformation and being row scaled. (B) MA-plot. Blue dots indicate significantly (adjusted p-value <0.05) differentially expressed genes, while gray dots indicate non-differentially expressed genes. (C) Heatmap of sample-to-sample distances on count matrix, which was variance-stabilized. Darker blue indicates a more similar expression level. (D) Principal Component Analysis from the 50 genes whose expression varies the most, using variance stabilizing transformation. The samples CG 60 h belong to the three independent cultivations in culture medium containing CG as carbon source, respectively at 60 h. The samples CGHH 36 h belong to the three independent cultivations in culture medium containing CGHH as carbon source, respectively at 36 h.

A)

B)

Figure S5: Differentially expressed genes in *Rhodotorula toruloides* CBS 14 within sampling time points in each of the growth media. Barplot representation of the number of genes that are differentially expressed within each of the KEGG pathways and cellular processes for cells grown on media containing (A) CGHH as main carbon source or (B) CG. The acounted genes showed significat (p < 0.05) upregulation (+y axis) or downregulation (-y axis) compared to the 10 h sampling point, with a log_2_Fold Change higher than 1.5. Bar colors indicate KEGG pathways. CG, crude glycerol; CGHH, mixture of CG and hemicellulose hydrolysate.


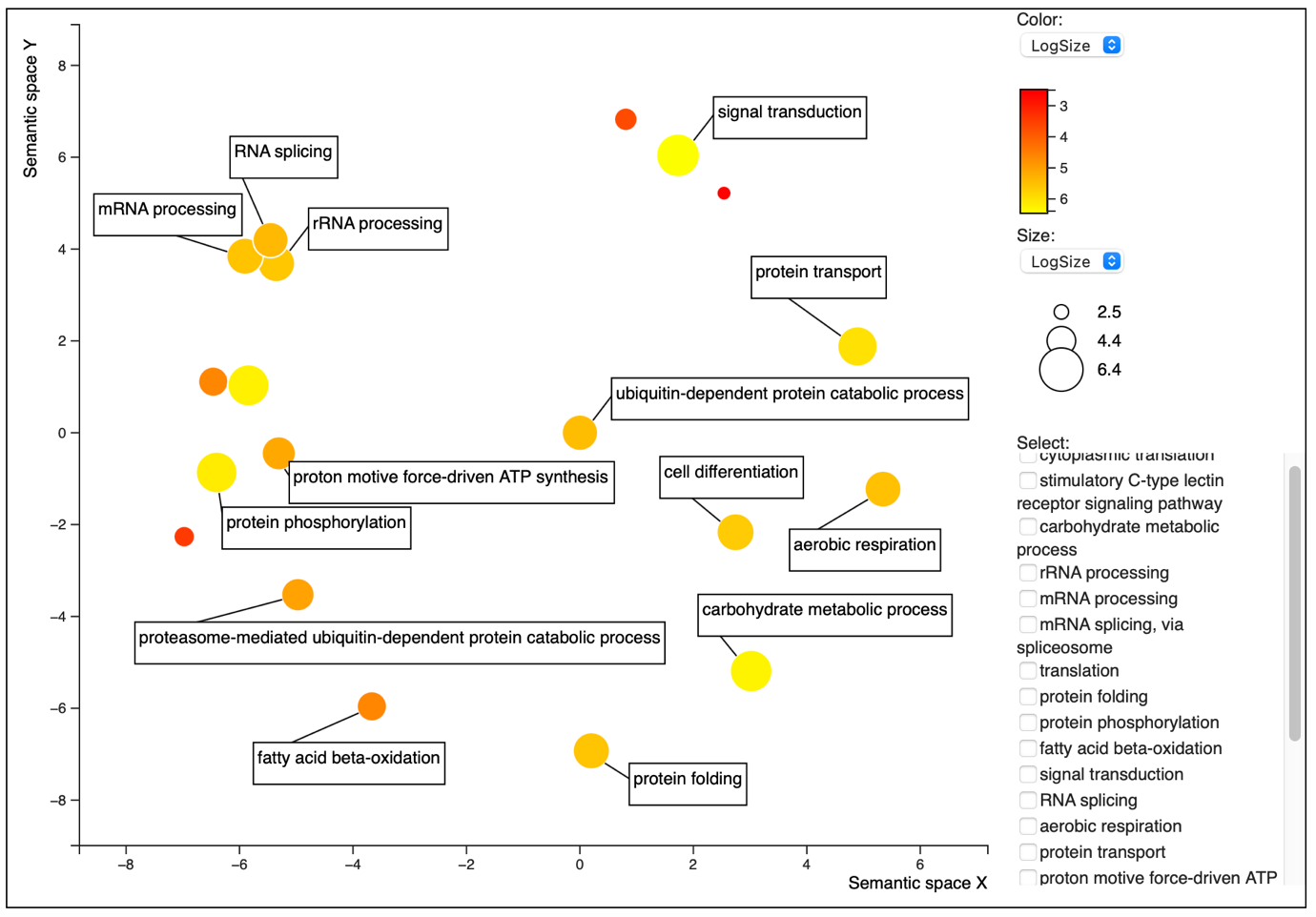


A)


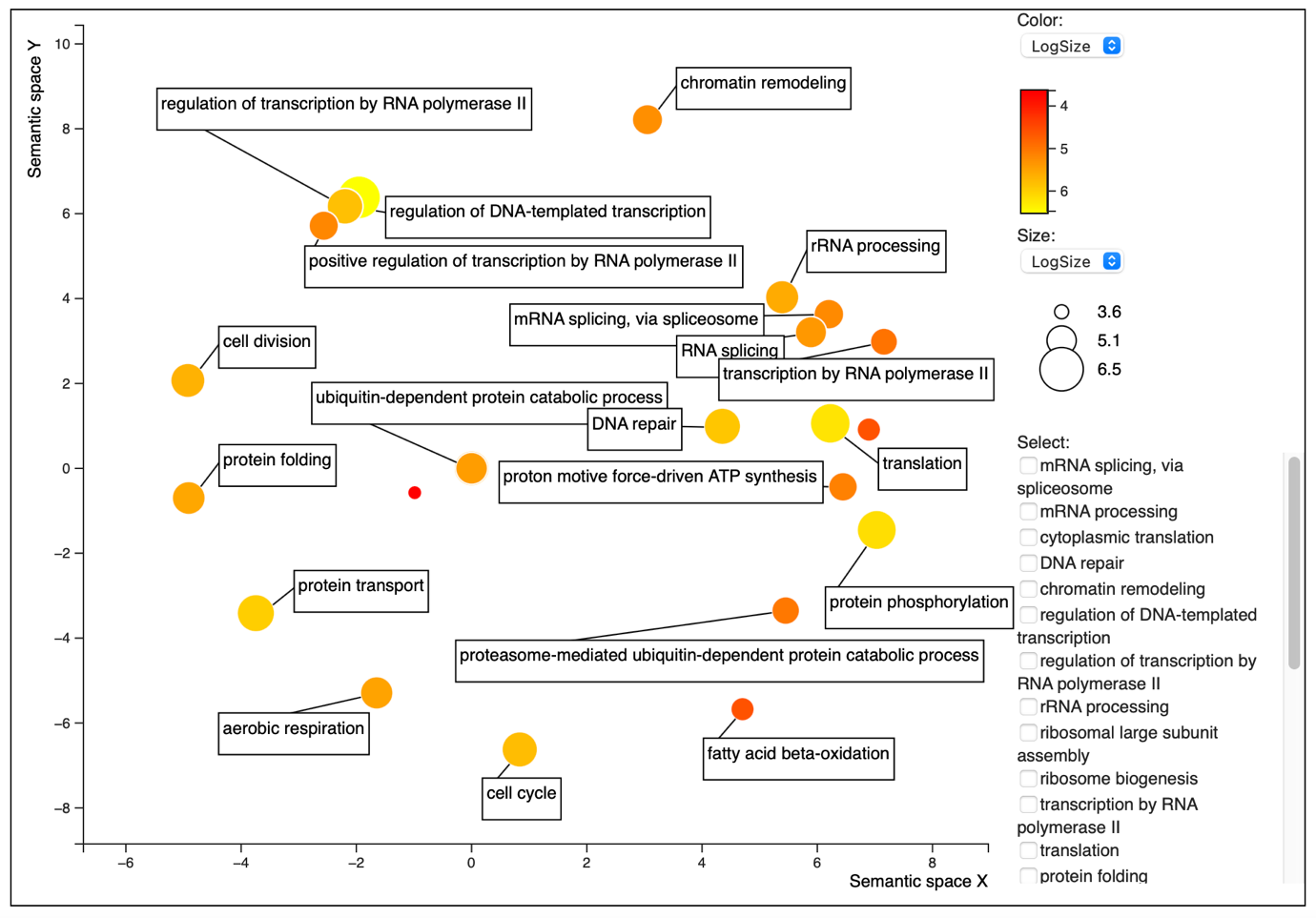


B)


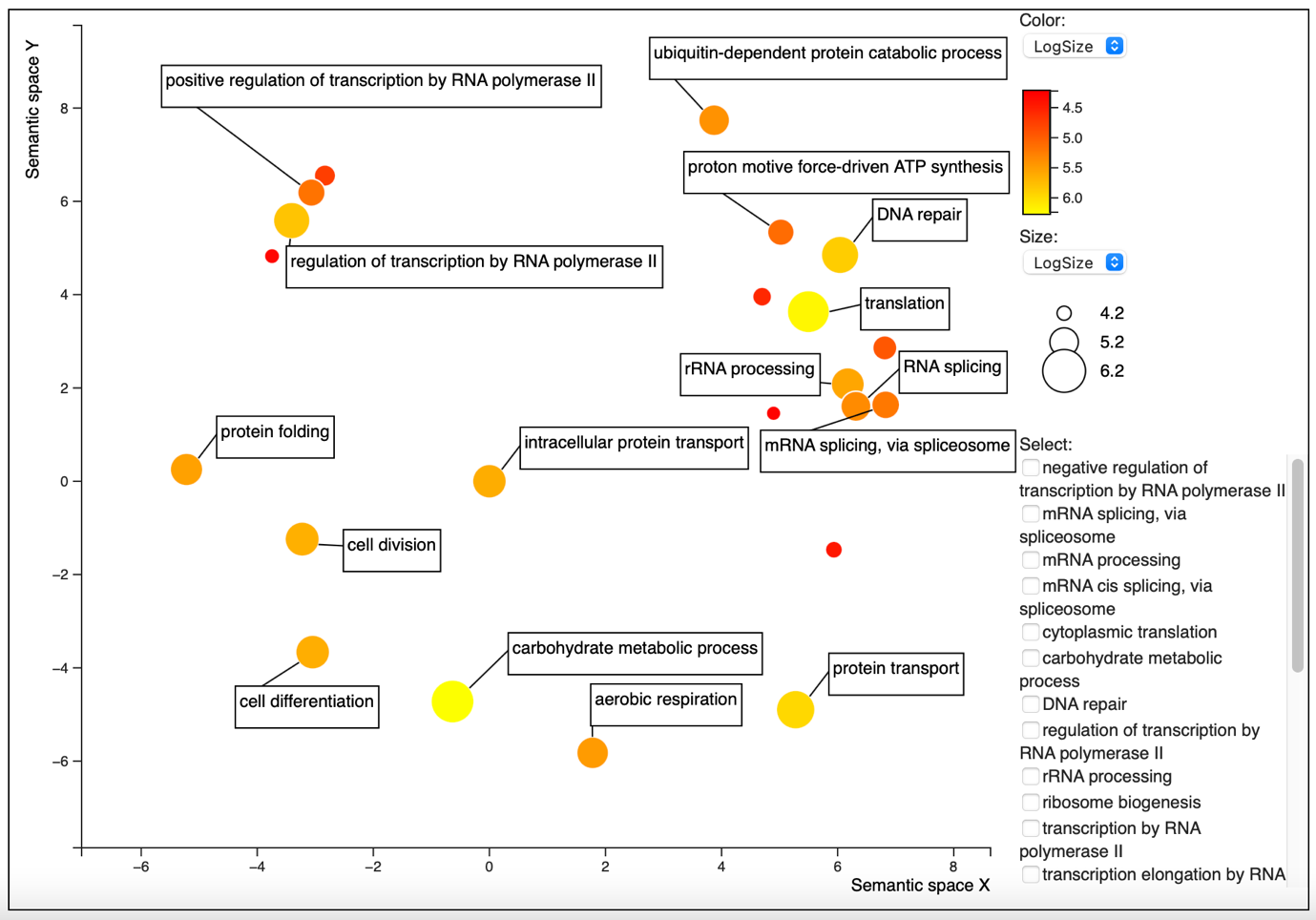


C)

Figure S6: Gene Ontology term summaries belonging to the GO topic: biological processes. Significantly differentially expressed genes (adjusted p-value <0.05) from similar physiological situations (For detailed explanation see section “RNA sampling points were selected according to the dynamics of glycerol consumption in CG or CGHH”): (a) CGHH 10 h versus CG 10 h; (b) CGHH 10 h versus CG 30 h; (c) CGHH 36 h versus CG 60 h.

­­­
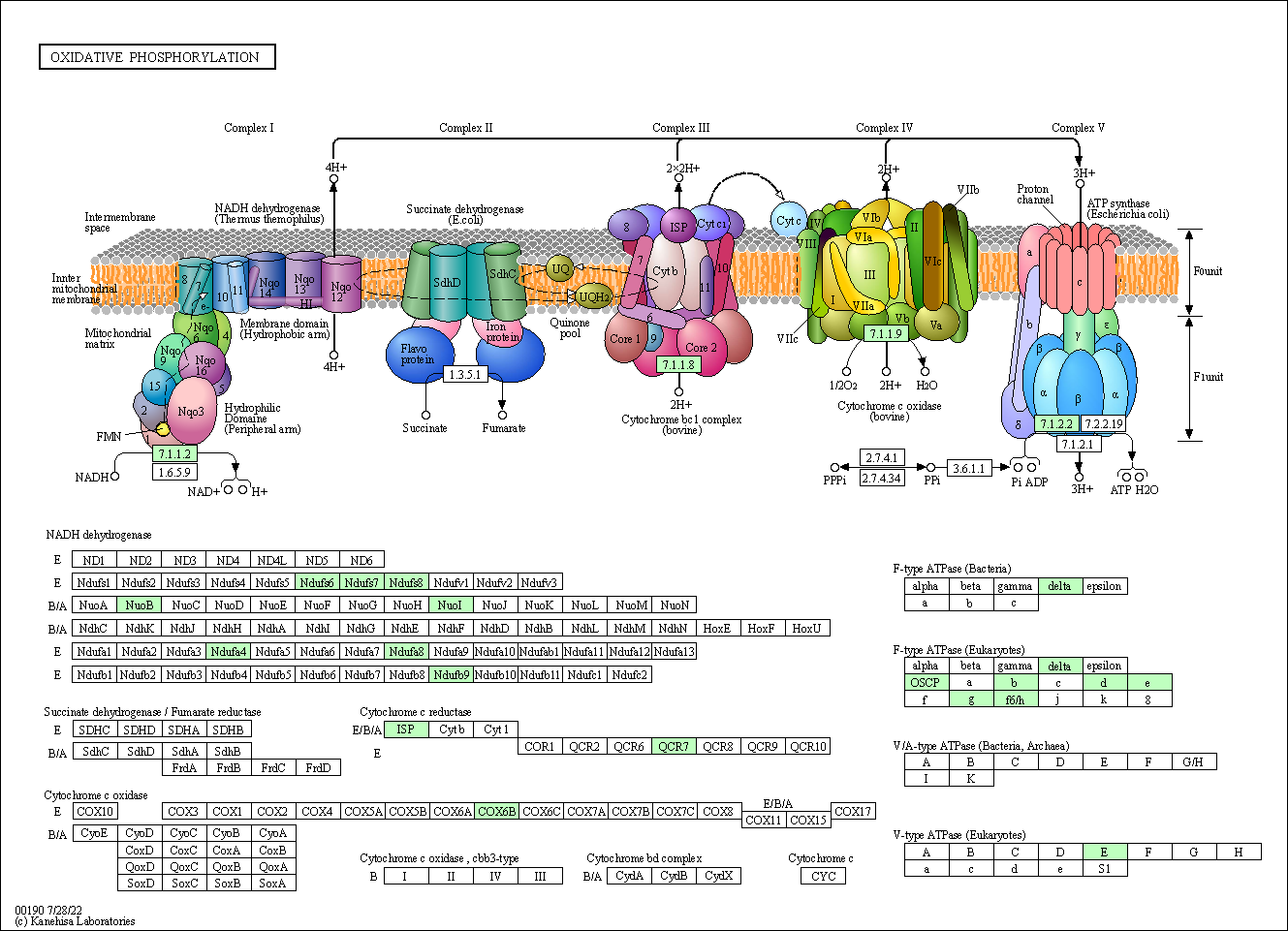


A)


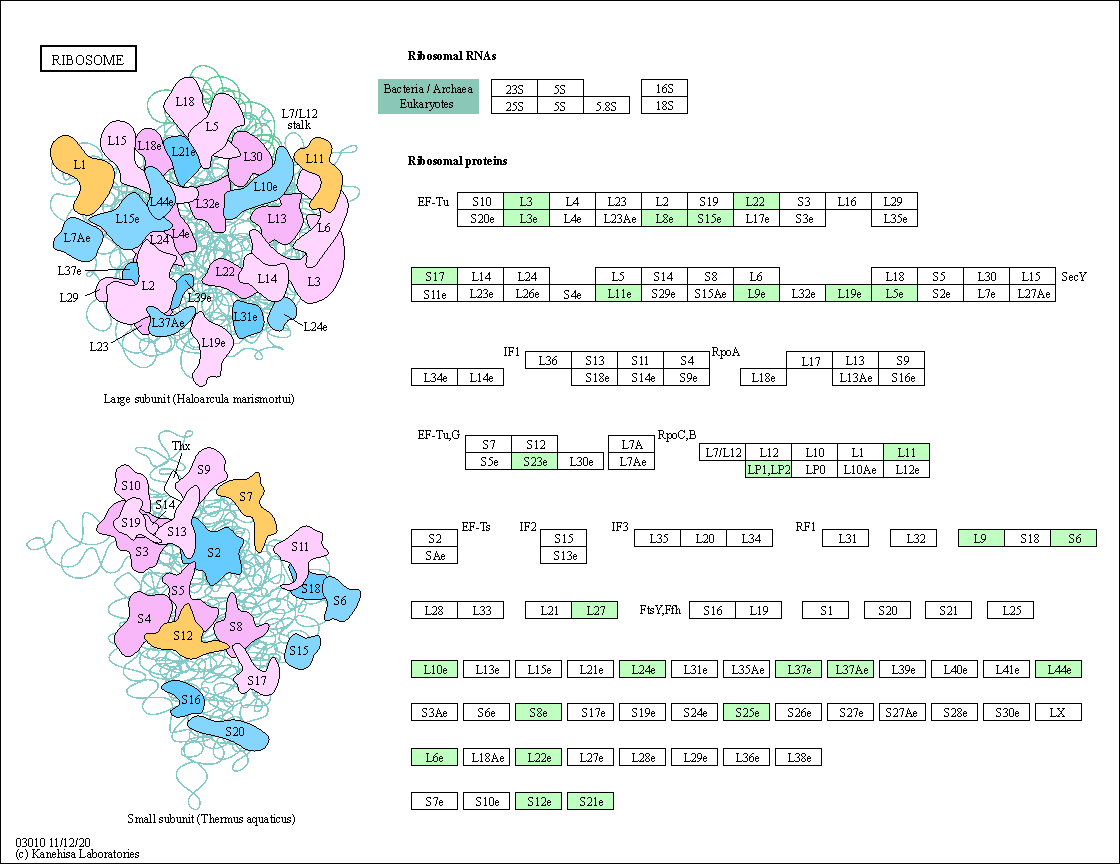


B)


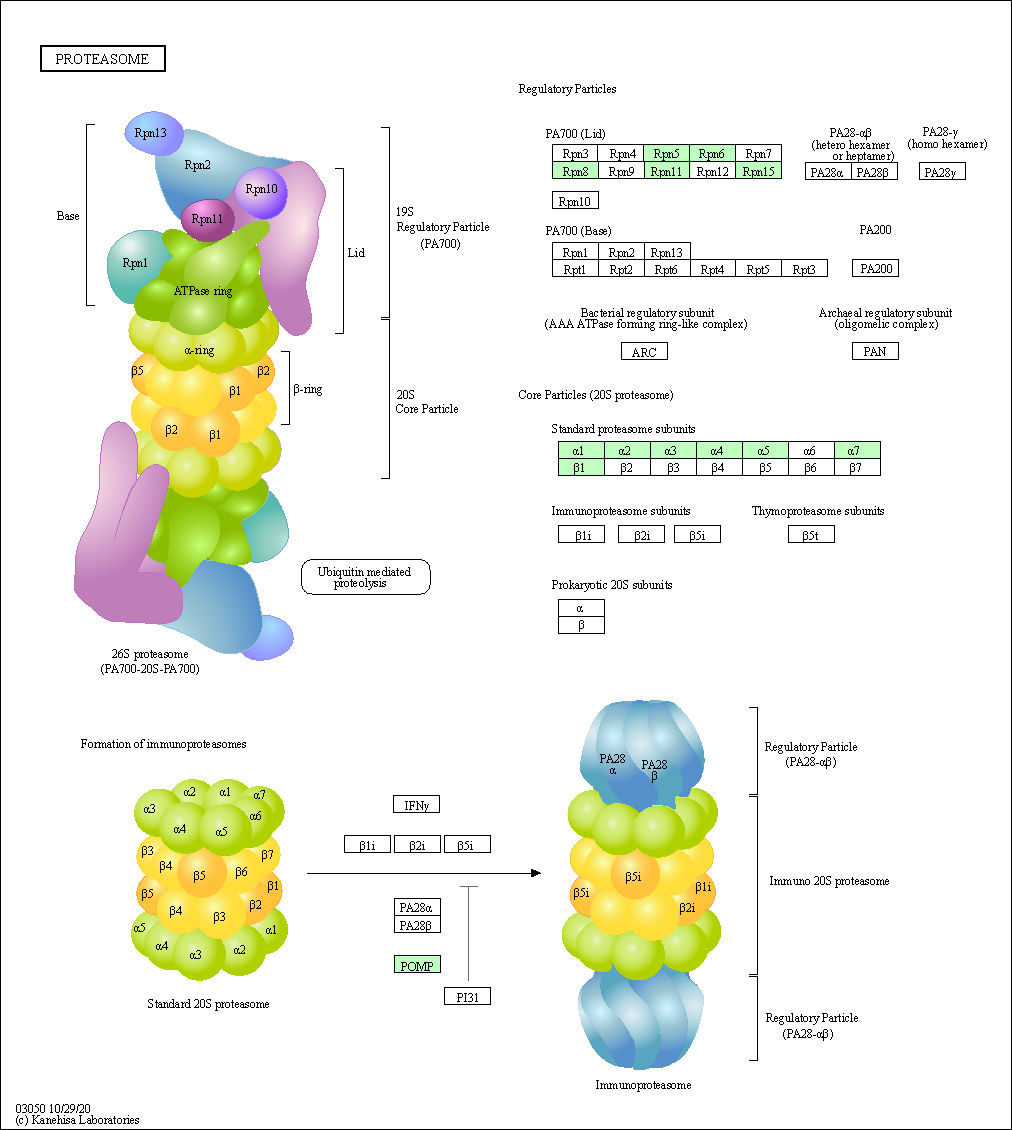


C)

Figure S7: Examples of upregulated genes in *Rhodotorula toruloides* CBS 14 grown on a mixture of crude glycerol (CG) and hemicellulose hydrolysate (CGHH) for 10 h of cultivation, whose expression is higher than CG 10 h. Genes encoding enzymes involved in (A) the respiratory chain, (B) ribosome biogenesis and (C) building of proteasome. Affected enzymes are highlighted in green. The figures were generated using the KEGG mapper.


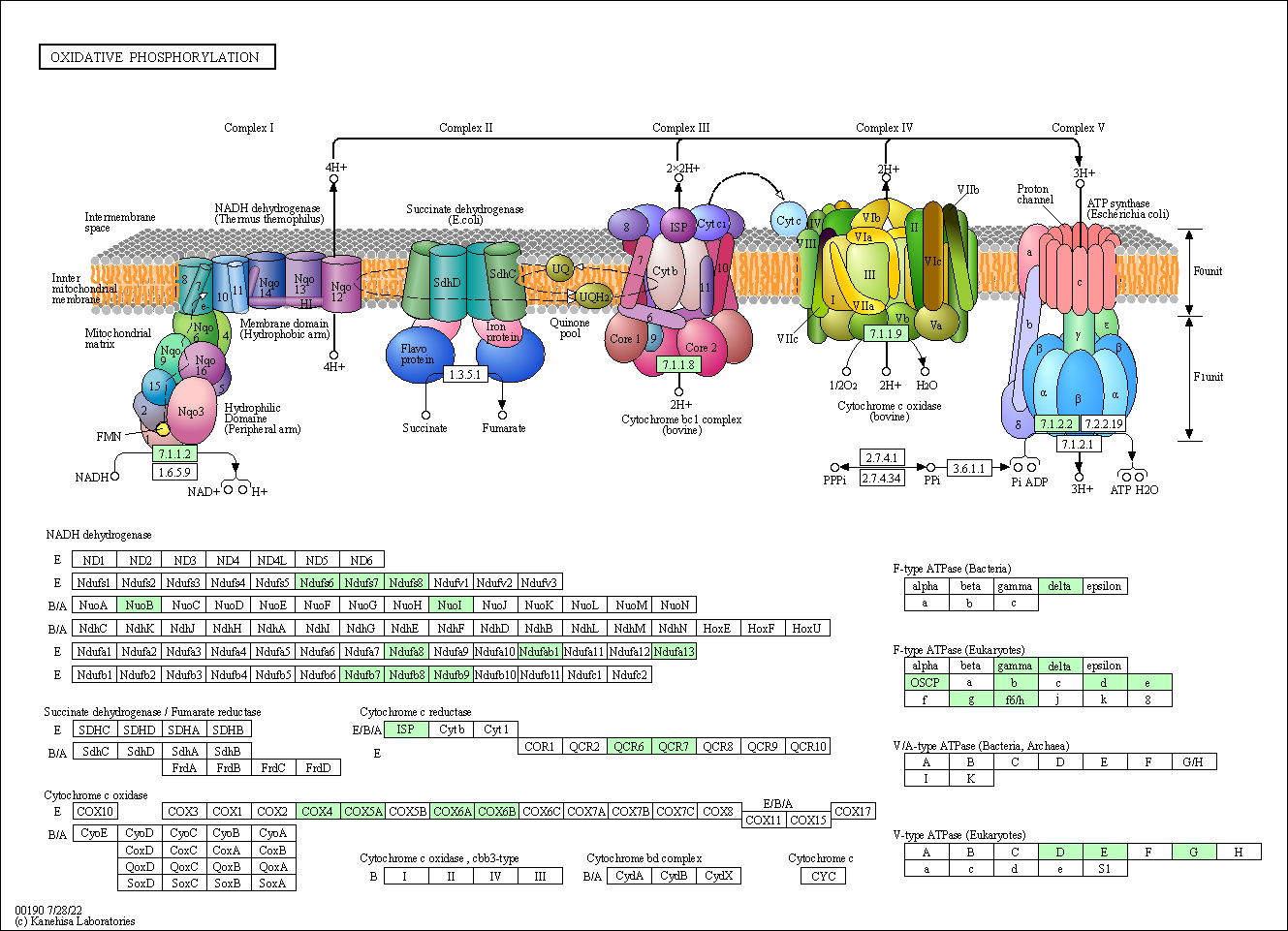


A)


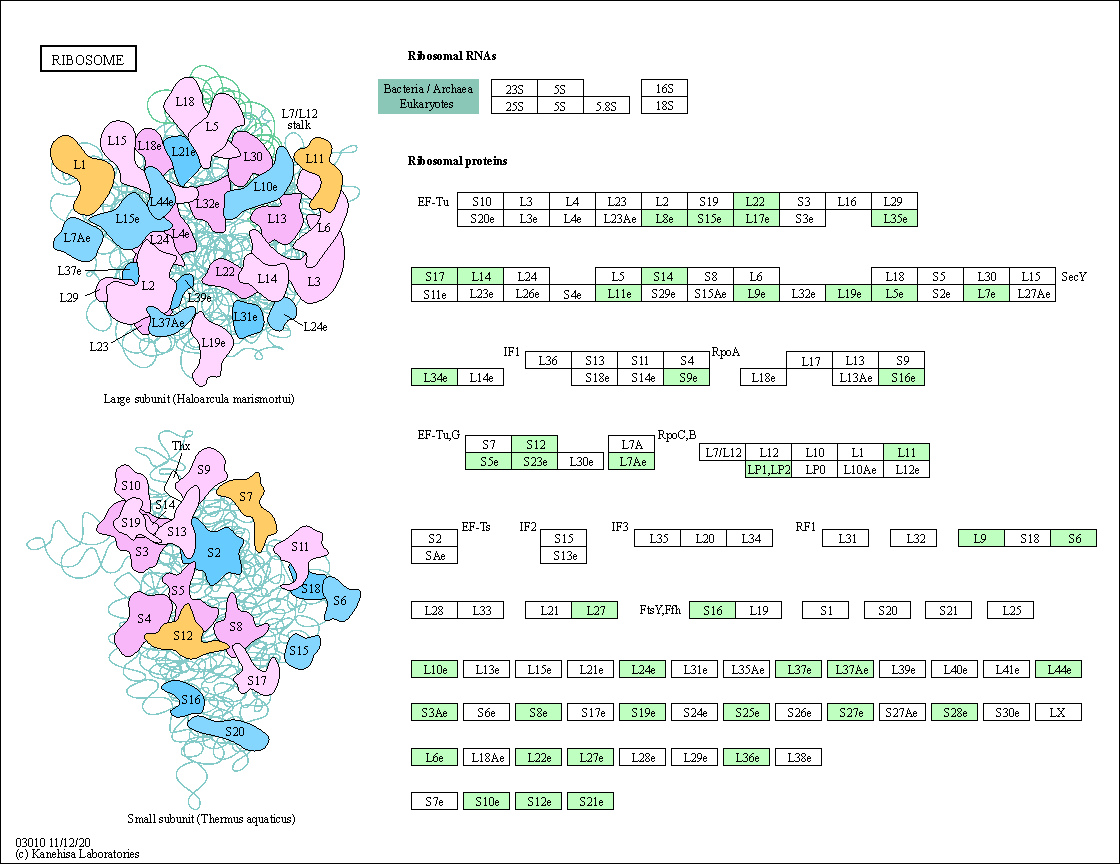


B)


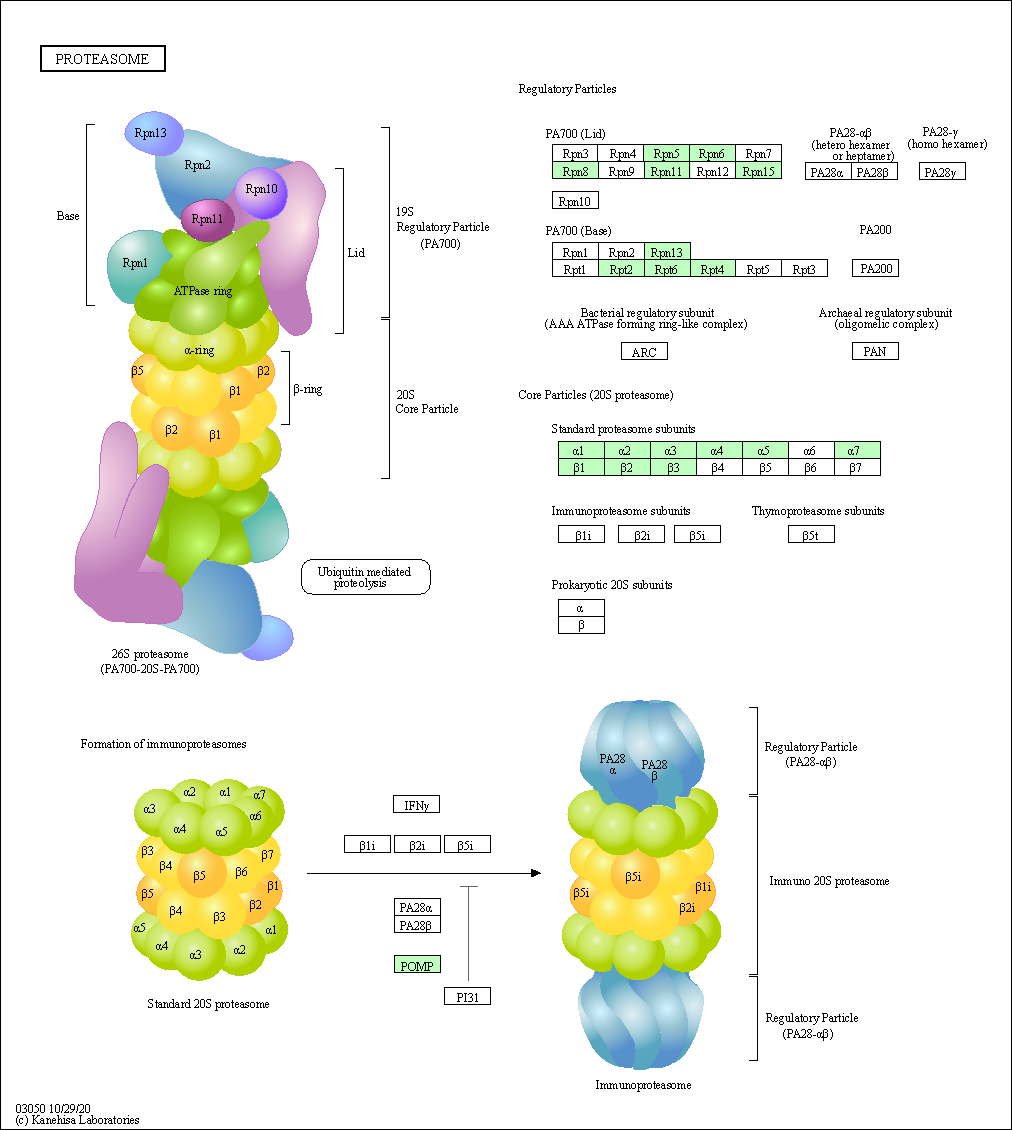


C)

Figure S8: Examples of upregulated genes in *Rhodotorula toruloides* CBS 14 grown on a mixture of crude glycerol (CG) and hemicellulose hydrolysate (CGHH) for 10 h of cultivation, whose expression is higher than CG 30 h. Genes encoding enzymes involved in (A) the respiratory chain, (B) ribosome biogenesis and (C) building of proteasome. Affected enzymes are highlighted in green. The figures were generated using the KEGG mapper.


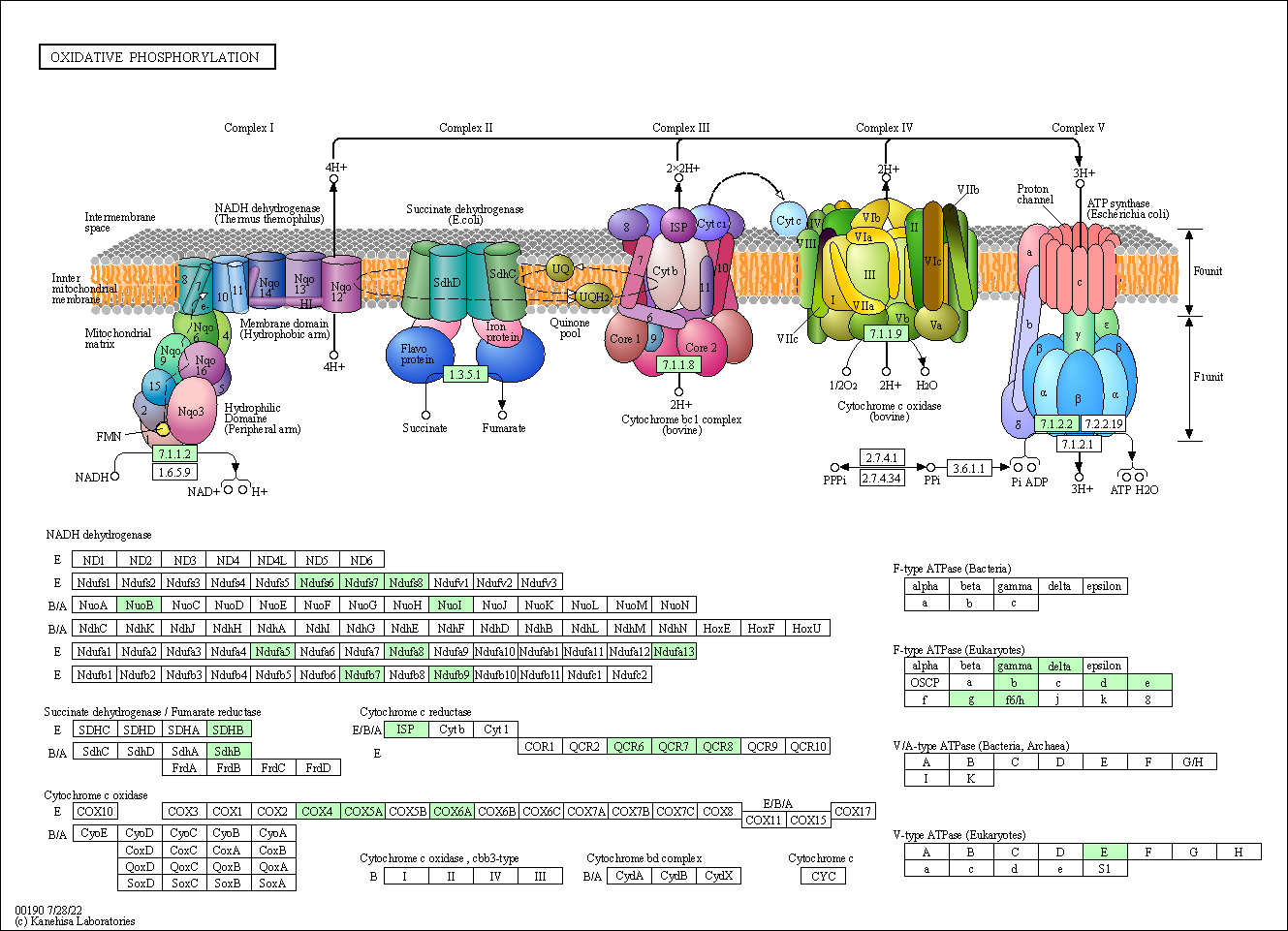


A)


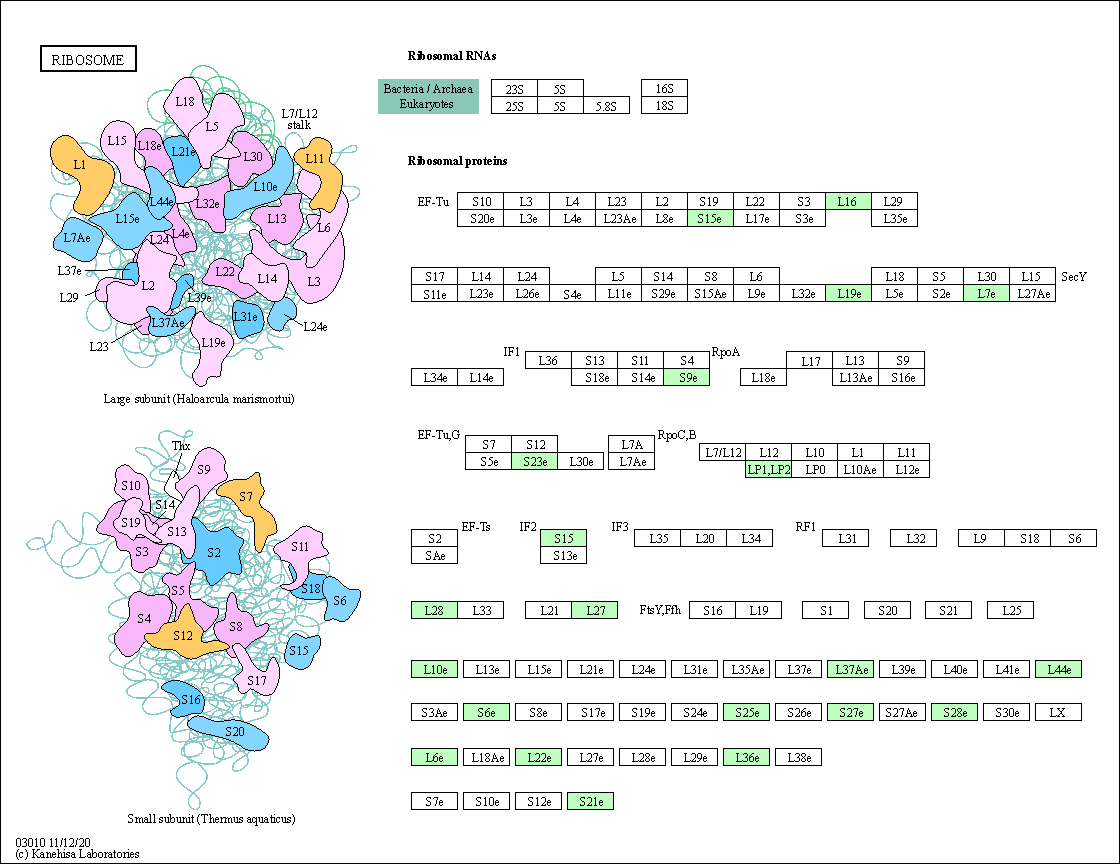


B)


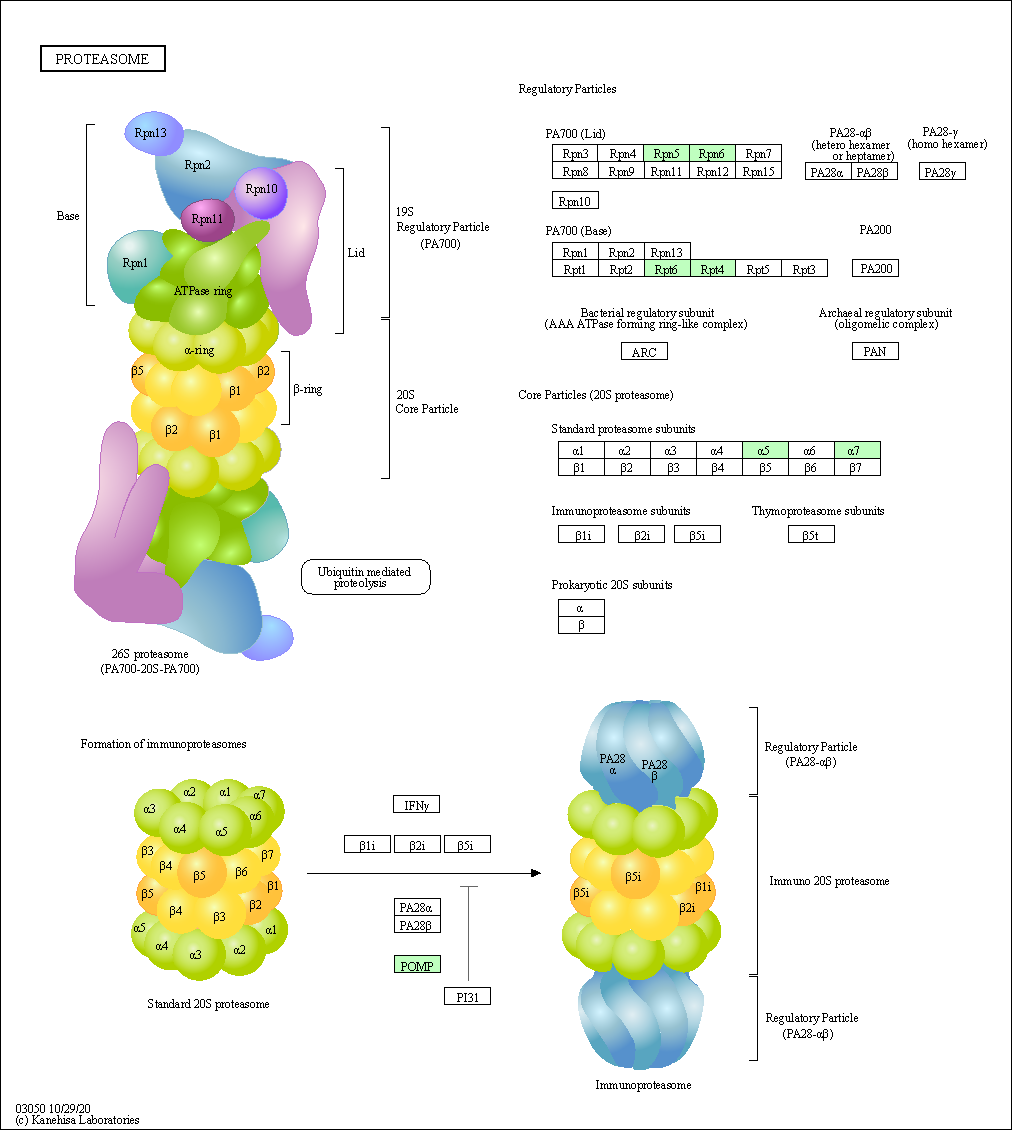


C)

Figure S9: Examples of upregulated genes in *Rhodotorula toruloides* CBS 14 grown on a mixture of crude glycerol (CG) and hemicellulose hydrolysate (CGHH) for 36 h of cultivation, whose expression is higher than CG 60 h. Genes encoding enzymes involved in (A) the respiratory chain, (B) ribosome biogenesis and (C) building of proteasome. Affected enzymes are highlighted in green. The figures were generated using the KEGG mapper.
